# Electrochemical Dehydrogenative sp^2^-Coupling Reaction of Naphthols Accessing a Polycyclic Naphthalenone Motif

**DOI:** 10.1021/acs.orglett.4c03518

**Published:** 2024-12-10

**Authors:** Julian Buchholz, Elisabeth K. Oehl, Maximilian M. Hielscher, Simone L. Kuhn, Dieter Schollmeyer, Siegfried R. Waldvogel

**Affiliations:** † Max-Planck-Institute for Chemical Energy Conversion, Stiftstraße 34−36, 45470 Mülheim an der Ruhr, Germany; ‡ Department of Chemistry, Johannes Gutenberg University, Duesbergweg 10−14, 55128 Mainz, Germany; § Karlsruhe Institute of Technology, Institute of Biological and Chemical, Systems − Functional Molecular Systems (IBCS − FMS), Kaiserstraße 12, 76131 Karlsruhe, Germany

## Abstract

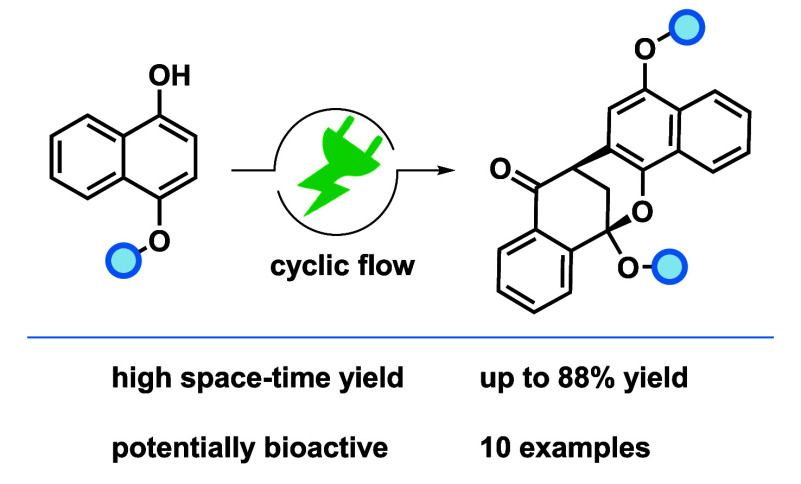

A novel polycyclic naphthalenone motif was obtained by electrochemical synthesis starting from naphthols. The process is solvent controlled, and the highly diastereoselective cyclization is due to a solvent cage. The direct, anodic dehydrogenative sp^2^-coupling was carried out by flow electrolysis. Ten derivatives containing this motif were synthesized in yields up to 88%, resulting in novel polycycles structurally similar to bioactive compounds like Daldionin, potentially exploring the bioactive profile.

Oxidative coupling is a versatile method with excellent functional group compatibility to create new phenolic compounds. Unlike classic coupling methods with transition metal catalysis,^1,2^ prefunctionalization of arene groups are not necessary. However, controlling multiple reactive sites within the molecules poses a challenge and can result in undesired coupling products. Oxidative phenol coupling has been explored for various metal systems such as iron^3^ and chromium.^4^ Factors such as oxidation potentials and nucleophilicities are employed to control the selectivity between homo- and cross-coupling of phenols and related compounds.^5^ In terms of the oxidation of 4-methoxy-1-naphthol (**1)** many different products can be obtained by changing the reaction conditions. For example, the formation of the homocoupling product **3** as well as a dinaphthofuran and the dehydrodimeric naphthoquinone is achieved with SnCl_4_ at prolonged reaction times in the presence of molecular oxygen.^6,7^ Notably, oxidation with the single electron oxidizer Ag_2_O leads to the conjugated diketone.^7^ A common issue related to using overstoichiometric amounts of oxidizing agents is too many byproducts and reagent waste. This can be prevented with anodic cross-coupling reactions. Electrochemical oxidative dehydrogenative coupling reactions have recently greatly expanded the toolbox of synthetic chemists. Here too, the otherwise necessary prefunctionalization of the coupling partners can be omitted, which leads to a minimized amount of reagent waste, thus simplifing the workup and costs.^8−15^ In the dehydrogenative coupling, only hydrogen is generated as a byproduct, requiring a cathode material with an adequate low overpotential for the hydrogen evolution reaction.^16−20^

We established a novel, unexpected polycyclic product (**2**) by applying conditions of phenol coupling reactions^17^ in 1,1,1,3,3,3-hexafluoro-2-propanol (HFIP) to 4-methoxy-1-naphthol (**1**). Thereby HFIP was identified as the key parameter for the formation of this motif. The combination of HFIP and quaternary ammonium salts has been demonstrated to provide an effective and stable electrolyte in coupling reactions between phenols and/or arenes, leading to high selectivity of the electrolysis process.^22^ The addition of protic additives, such as water or methanol, can be utilized to control selectivity when there is a mismatch in the oxidation potentials and nucleophilicity of the coupling partners.^23^ Without this solvent control in electrosynthesis,^24^ phenols tend to form polycyclic architectures which have similarities to naturally occurring compounds.^25−28^ In addition, only the homocoupling product **3** and the overoxidized dehydrodimeric naphthoquinone **4** were reported by electrochemical anodic oxidation of **1** in acetonitrile (Figure 1). Thus, the choice of HFIP as a solvent appears to play a decisive role in the electrochemical formation of the polycyclic motif.^21^ The obtained polycycle shares the same carbon skeleton with natural compound Daldionin (**5**) (Figure 1). **5** is a bioactive natural product isolated from the fungal orchid endophyte *Daldinia eschscholtzii*. It exhibits structural similarities to that of our novel polycyclic naphthaleonone. Daldionin has interesting antiproliferative features against immortalized leukemia cells (HUVEC and K-562 cell lines). **5** also shows antimicrobial properties against several bacteria such as *B. subtilis* 6633 B1; *S. aureus* 134/93 R9; *E. faecalis* 1528 R10 (partial).^29^

**Figure 1:**
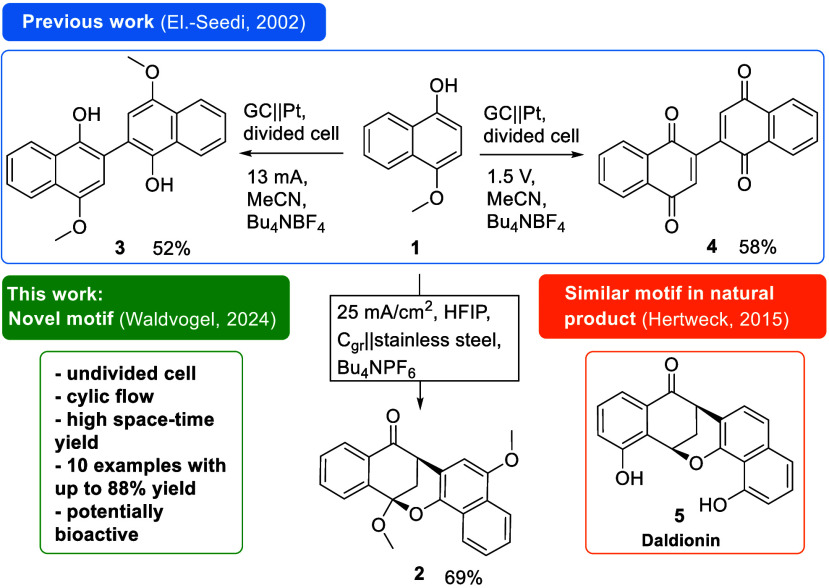
Electrochemical approaches on the oxidation of 4-methoxy-1-naphthol (**1**).^6,7,21^

Optimizing combined classical and electro-organic synthesis is challenging due to intricate interactions between reaction parameters, rendering traditional sequential optimization methods, in which one parameter is optimized after the other, ineffective. Design of Experiment (DoE) provides a remedy by examining all the reaction parameters under consideration simultaneously and in a balanced manner.^30,31^ The electrolysis itself was carried out in a flow. In comparison to batch-type electrolysis, flow electrolysis exhibits a higher surface-to-volume ratio and a reduced contact time of the electrolyte at the electrodes.^32,33^ Due to the latter, it is particularly suitable for substrates being prone to overoxidation which forms polymeric byproducts and a deposit on the anode (see Supporting Information (SI)). In our experiments the electrolyte is pumped through the cell multiple times from a reservoir. We present a sustainable electro-organic synthesis for this novel naphthalenone polycyclic motif which might have similar bioactivity.^34,35^

Our initial goal was to develop a direct oxidative synthesis of binaphthols such as 4,4′-dimethoxy-(2,2′-binaphthalene)-1,1′-diol starting from α-naphthol derivatives like **1** in an undivided cell using HFIP based electrolytes.^21^

To our surprise, additionally to the formation of the homocoupling product **3**, a methylene-bridged oxocin-8-one species was found as the major product. The structure of **2** was elucidated by spectrometric and spectroscopic means. X-ray analysis of a single crystal unequivocally confirmed the molecular structure (CCDC 2377041).

For systematical optimization of the synthesis of **2**, an initial electro synthetic screening and a two additive screening series with water and methanol were carried out. Followed by a 2^5–3^ fractional factorial design to investigate the temperature (*T*), current density (*j*), applied amount of charge (*Q*), concentration of the supporting electrolyte (*c*(NEt_4_PF_6_)), and concentration of the starting material (*c*(**1**)) (*R*^2^ = 95% and α = 0.1).^30,36^ Based on this, a steepest ascent screening and a full factorial design (DoE parameters: *T*, *j*, and *Q*; *R*^2^ = 33% and α = 0.1) were carried out. The optimized conditions are displayed in Figure 2, and the isolated yield of **2** was increased from 37% to 69%. A final screening of *j* (with 5 mA/cm^2^ steps) did not increase the yield any further. The productivity as well as the space–time yield were doubled during the optimization steps (Table 1). The 10-fold scale-up of the electrochemical synthesis of **2** resulted in a 52% yield. The productivity increased, while the space–time yield decreased (Table 1). The optimized conditions (Figure 2) were applied to a collection of naphthols with a diverse substitution pattern in position 4.The limiting factors appear to be both sterically demanding starting materials such as for **7** compared with **8** as well as electron-withdrawing groups like **16**. Limitations also arose due to low solubility, especially for example **9** and overoxidation which is described in detail in the SI. The alkyl chain length of the substituent seems to have a positive impact on the polycycle formation leading to good yields of 76% and 72% comparing **6** and **7**, respectively. A stepwise decrease in yields can be observed for more hydrophobic molecules (**9**, **10**, and **11**) due to poor solubility. **15**, **16**, and **17** contain moieties which could also be leaving groups. This could explain the low conversion toward the desired polycyclic system, like **1**, allowing the enrichment of this species during the electrochemical reaction.

**Table 1:** Overview of the Productivity and Space–Time Yield after the Optimization and in Scale-up of **2**

Electrolyzer	Productivity [g/h]	Space–time yield [g/(h·mL)]
2 × 6 cm^2^ flow cell (initial conditions)	0.684	2.281
2 × 6 cm^2^ flow cell (optimized conditions)	1.343	4.476
4 × 12 cm^2^ flow cell (optimized conditions)	3.922	3.628

**Figure 2:**
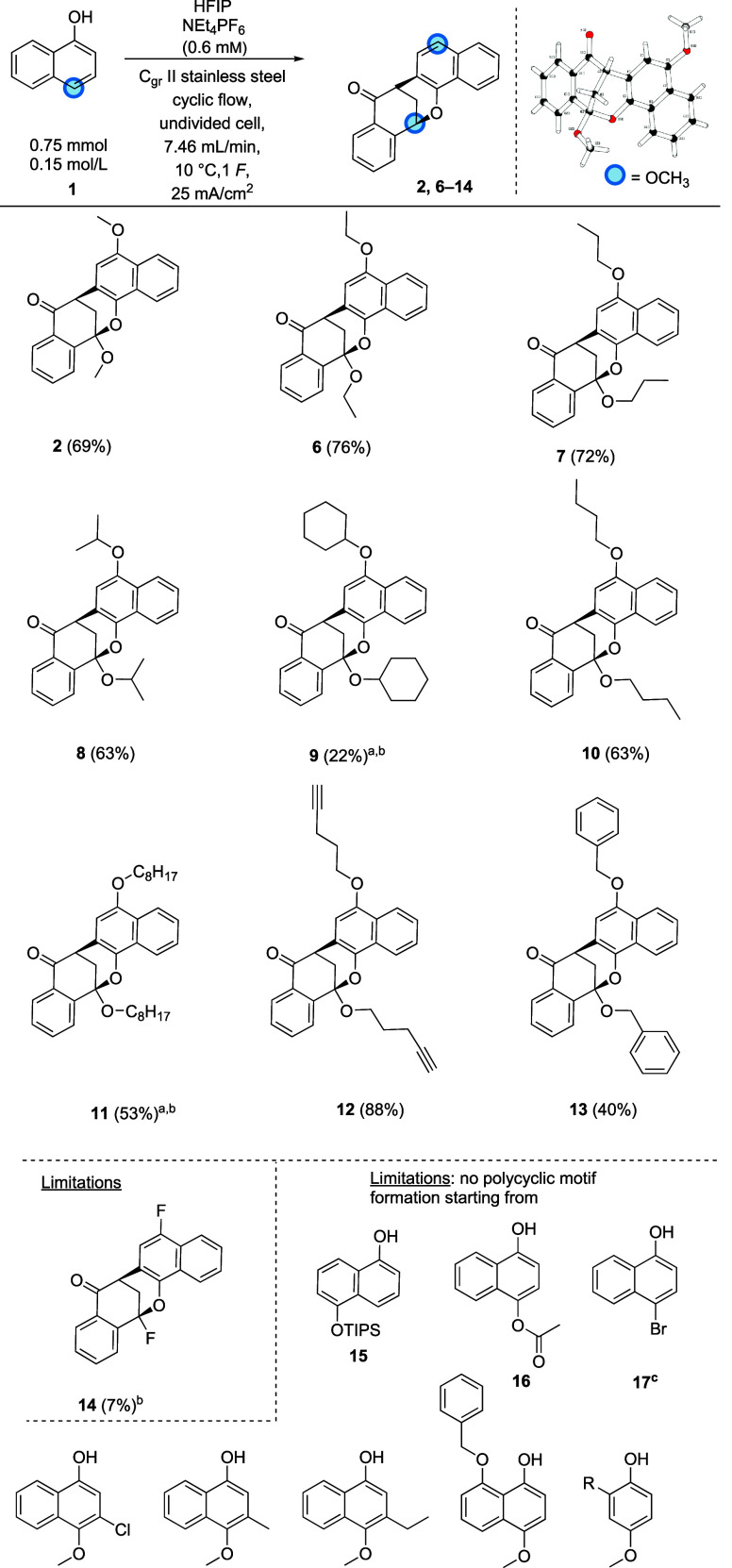
Scope (^a^*T* = 30 °C, ^b^one-fifth of 5 mL HFIP was equally substituted by chlorobenzene or DCM, ^c^performed a batch-type cell; all to enhance solubility of the respective starting material).

Furthermore, different 4-methoxyphenols (**22**–**25**) were tested under the optimized conditions. Since no fused arene blocks the other side of the phenol, mainly polymerization was observed as fouling at the anode. The *tert-*butyl derivative **25** was investigated since one side is covered and *tert*-butyl mimics the lipophilicity of the arene, helping to form the HFIP cage around it. This results in traces of coupling products that can be observed via GC-MS and NMR, while polymerization dominates the reaction also in this example.

Especially interesting is **12**, which was obtained in 88% yield and could be further functionalized via the click reaction and potentially tuned for the fit in possible enzymatic targets. We conducted several cyclic voltammetry (CV) studies (see SI). First, 4-methoxynaphthol (**1**) and 4-methoxynaphthalene-1-yl acetate (**16**) were compared to clarify the significant difference in polycycle formation of **16** compared to **2**. As expected, **16** showed a significantly higher oxidation potential compared to our model substrate **1**. Second, the CV data of **14** have similar characteristics to **1** (see SI). Third, the CV measurements of the polycyclic product **2** showed a higher oxidation potential than the studied naphthols. In the proposed mechanism (Figure 3), **1** is initially oxidized accompanied by loss of a proton. Due to the minimal reorganization energy, the nucleophilic attack of the second naphthol **1** toward the neutral, electrophilic^37^ radical species is strongly favored.^38^**28** is protonated by the adjacent hydroxyl group followed by the nucleophilic attack of the naphtholate subsequently onto the carbocation formed. The activation in this step is provided by HFIP’s strong hydrogen bonding (visually indicated for **28** and **29**). It is known in literature that HFIP can establish a solvent cage.^39^ The second oxidation in combination with extrusion of a proton leads to rearomatization of the naphthol moiety within the polycycle. Two strategies were used for mechanistic clarification: One strategy was to block specific positions, and the other strategy was to run the electrolysis in deuterated solvent. The acidic proton of the hydroxyl group of 4-methoxy-1-naphthol (**1**) is essential for the formation of **2**. If this is altered by a methyl group (1,4-dimethoxynaphthalene (**31**)) no formation of the respective polycyclic motif was traced by GC/MS analysis–only unconverted starting material (Figure 3). In addition, the substitution of a hydrogen atom in position 3 by a chloro substituent (**18**), methyl (**19**) or ethyl group (**20**) did not lead to any polycyclic product (Figure 2), probably because of their steric requirements. Furthermore, it was investigated whether a quinone-ketal dimer could be a possible intermediate of the reaction. Therefore, the methanol ketal (**33**) was used in combination with **1** under optimized conditions. Due to the large drop in yield, a quinine-ketal was excluded in order to be an intermediate. To identify the origin of the proton at the methylene bridge the coupling was carried out in HFIP-*d*_2_. The ^2^H NMR spectra of the coupling product revealed that one deuterium atom is predominantly incorporated at the methylene bridge. One signal in the ^2^H NMR spectra is formed with the decrease of the integral of only one out of two *dd* in the ^1^H NMR, which correspond to the methylene bridge protons (see SI). Due to a deuteriation degree of only 34% (where a maximum of 50% is possible, as one proton is already present), the origin of the deuterium originated most likely intramolecularly from the neighboring hydroxyl group rather than intermolecularly by the acidic solvent HFIP. HFIP plays a crucial role because the novel structural motif of **2** was not claimed by any other literature known approach, classical or electrochemical, using acetonitrile or nitromethane as a solvent. We can confirm that, with our optimized reaction conditions in acetonitrile instead of HFIP, the homocoupling product (39% yield) is favored over the polycycle formation (14% yield) starting from **1**. HFIP forms a solvent cage and therefore promotes the conformation of the formed polycyclic system due to strong hydrogen bonding interaction with HFIP.^39^

**Figure 3:**
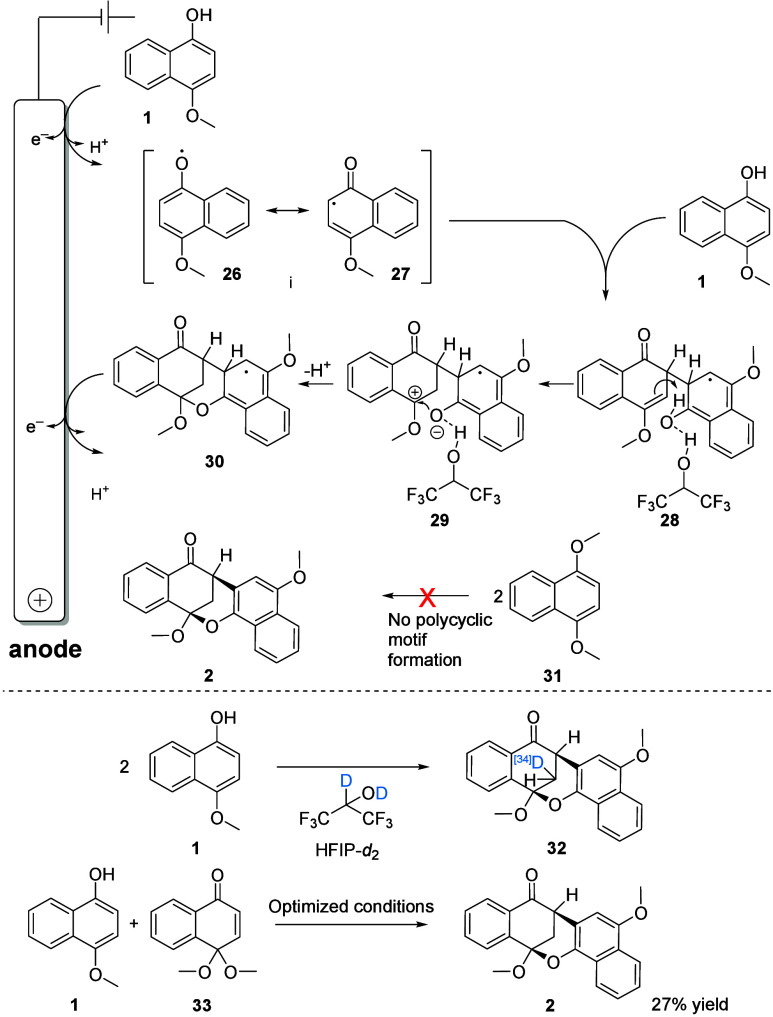
Proposed mechanism for polycycle formation.

In summary, the electrochemical dehydrogenative sp^2^-couplings of naphthols toward a novel polycyclic naphthalenone motif was demonstrated. The synthesis of **2** was optimized using DoE as an effective optimization tool, doubling the space–time yield of the initial reaction. We were able to obtain 10 diverse examples with an up to 88% yield by applying the optimized conditions. Mechanistic studies were conducted, indicating the high importance of the solvent cage formed by HFIP for activation of the key intermediate and the cyclization. The reaction design in a cyclic flow system shows advantages in the prevention of overoxidation and a strong indication of scalability for polycyclic naphthalenone motifs, which shares the same carbon skeleton with the natural compound Daldionin (**5**) showing unique bioactivity.

## Data Availability

The data underlying this study are available in the published article and its Supporting Information.

## Supporting Information Available

Experimental procedures and product characterization and single crystal X-ray data (PDF)
