# Supplementary material for: Electrochemical Dehydrogenative sp2-Coupling Reaction of Naphthols Accessing a Polycyclic Naphthalenone Motif
Source: Org Lett. 2024 Dec 10;27(1):25–9. doi: 10.1021/acs.orglett.4c03518 (PMC11731377; doi:10.1021/acs.orglett.4c03518)
Supplement: Supplementary file 1 — ol4c03518_si_001.pdf [file ol4c03518_si_001.pdf]

Supporting Information

Electrochemical Dehydrogenative sp^2- Coupling Reaction of Naphthols Accessing a Polycyclic Naphthalenone Motif

Julian Buchholz‡ [a], Elisabeth K. Oehl‡ [b], Maximilian M. Hielscher[b], Simone L. Kuhn[b], Dieter Schollmeyer[b], and Siegfried R. Waldvogel[a,b,c]

[a] Max-Planck-Institute for Chemical Energy Conversion, Stiftstraße 34–36, 45470 Mülheim an der Ruhr, Germany.

[b] Department of Chemistry, Johannes Gutenberg University, Duesbergweg 10–14, 55128 Mainz, Germany.

[c] Karlsruhe Institute of Technology, Institute of Biological and Chemical, Systems – Functional Molecular Systems (IBCS – FMS), Kaiserstraße 12, 76131 Karlsruhe, Germany.

E-mail: siegfried.waldvogel@cec.mpg.de

‡ The authors contributed equally to this work.

Table of Content

1. General information ... S3
2. Quantification ... S6
3. Mechanistic studies ... S7
3.1 Preliminary experiments ... S7
4. Optimization data ... S14
4.1 Initial experiments ... S14
4.2 Overview of the optimization process ... S16
4.3 Choice of parameter range ... S17
4.4 ANOVA and linear regression regarding yield of (**2**) ... S17
4.5 Steepest ascent screening ... S20
4.6 ANOVA and linear regression regarding the yield (**1**) ... S21
4.5. Screening of current density ... S24
4.6. Reusability of the electrode ... S25
4.7. Analysis of side products ... S26
4.8. Cross-coupling reactions ... S29
5. General protocols ... S31
6. Synthesis of the naphthol's and polycycles ... S32
6.1 Synthesis of naphthol's ... S32
6.1.1 4-Methoxy-1-naphthol (**1**) ... S32
6.1.2 4-Ethoxy-1-naphthol (**37**) ... S32
6.1.3 4-Propyloxy-1-naphthol (**36**) ... S33
6.1.4 4-(1-Methylethyl)oxy-1-naphthol (**38**) ... S34
6.1.5 4-Pentyloxy-1-naphthol (**39**) ... S34
6.1.6 4-Cyclohexyloxy-1-naphthol (**40**) ... S35
6.1.7 4-Octyloxy-1-naphthol (**41**) ... S36
6.1.8 Pent-4-yn-1-yl methanesulfonate (**42**) ... S36
6.1.9 4-(Pent-4-yn-1-yl)oxy-1-naphthol (**43**) ... S37
6.1.10 4-Benzyloxy-1-naphthol (**44**) ... S38
6.1.11 5-((Tri-(1-methylethyl)silyl)oxy)naphthalen-1-ol (**15**) ... S38
6.1.12 4-Acetoxy-1-naphthol (**16**) ... S39
6.1.13 4-Bromonaphthalen-1-ol (**17**) ... S40
6.1.14 2-Chloronaphthalene-1,4-diol (**46**) ... S41
6.1.15 2-Chloro-4-((tri-(1-methylethyl)silyl)oxy)naphthalen-1-ol (**47**) ... S41
6.1.16 ((3-Chloro-4-methoxynaphthalen-1-yl)oxy)tri-1-methylethylsilane (**48**) ... S42
6.1.17 3-Chloro-4-methoxynaphthalen-1-ol (**18**) ... S42
6.1.18 2-Ethylnaphthalene-1,4-dione (**50**) ... S44
6.1.19 2-Methylnaphthalene-1,4-diol (**51**) ... S44
6.1.20 2-Ethylnaphthalene-1,4-diol (**52**) ... S45

6.1.21 4-(2-Chloroethoxy)-2-methylnaphthalen-1-ol (**53**) S45
6.1.22 4-(2-Chloroethoxy)-2-ethylnaphthalen-1-ol (**54**)........ S46
6.1.23 4-(2-Chloroethoxy)-1-methoxy-2-methylnaphthalene (**55**) S46
6.1.24 4-(2-Chloroethoxy)-1-methoxy-2-ethylnaphthalene (**56**) S47
6.1.25 4-(2-Chloroethoxy)-1-methoxy-2-methylnaphthalene (**19**) S47
6.1.26 4-(2-Chloroethoxy)-1-methoxy-2-ethylnaphthalene (**20**) S48
6.1.27 5-(Benzyloxy)naphthalene-1,4-dione (**58**) S49
6.1.28 5-(Benzyloxy)naphthalene-1,4-diol (**59**)........ S50
6.1.29 8-(Benzyloxy)-4-methoxynaphth-1-ol (**21**) S51
6.1.30 4,4-Dimethoxynaphthalen-1(4*H*)-one (**33**) S51
6.2 Synthesis of polycycles S53
6.2.1 7*H*,13*H*-5,13-Dimethoxy-7,13-methanobenzo[*f*]naphtho[1,2-*b*]oxocin-8-one (**2**) S53
6.2.2. 7*H*,13*H*-5,13-Dimethoxy-7,13-ethanobenzo[*f*]naphtho[1,2-*b*]oxocin-8-one (**6**) S55
6.2.3. 7*H*,13*H*-5,13-Dimethoxy-7,13-ethanobenzo[*f*]naphtho[1,2-*b*]oxocin-8-one (**7**) S56
6.2.4. 7*H*,13*H*-5,13-Di(1-methyleth)oxy-7,13-methanobenzo[*f*]naphtho[1,2-*b*]oxocin-8-one (**8**) .. S57
6.2.5. 7*H*,13*H*-5,13-Di(1-cyclohexyl-7,13-methanobenzo[*f*]naphtho[1,2-*b*]oxocin-8-one (**9**)........ S57
6.2.6. 7*H*,13*H*-5,13-Dibutanoxy-7,13-methanobenzo[*f*]naphtho[1,2-*b*]oxocin-8-one (**10**) S58
6.2.7. 7*H*,13*H*-5,13-Dioctanoxy-7,13-methanobenzo[*f*]naphtho[1,2-*b*]oxocin-8-one (**11**)........ S59
6.2.8. 7*H*,13*H*-5,13-Di(pent-4-yn-1yloxy)-7,13-methanobenzo[*f*]naphtho[1,2-*b*]oxocin-8-one...... S60
6.2.9. 7*H*,13*H*-5,13-Di(benzyloxy)-7,13-methanobenzo[*f*]naphtho[1,2-*b*]oxocin-8-one S61
6.2.10. 7H,13H-5,13-Difluoro-7,13-methanobenzo[*f*]naphtho[1,2-b]oxocin-8-one (**14**) S62
6.2.11. Limitations in the electrolysis of 5-((tri-(1-methylethyl)silyl)oxy)naphthalen-1-ol (**15**) S63
6.2.12. Limitations in the electrolysis of 4-hydroxynaphthalen-1-yl (**16**)........ S64
6.2.13. Further investigated limitations........ S65
7 Crystallographic data........ S70
7.1 7*H*,13*H*-5,13-Dimethoxy-7,13-methanobenzo[*f*]naphtho[1,2-*b*]oxocin-8-one (**2**) S70
7.2 7*H*,13*H*-5,13-Dimethoxy-7,13-ethanobenzo[*f*]naphtho[1,2-*b*]oxocin-8-one (**6**)........ S73
7.3 7*H*,13*H*-5,13-Dimethoxy-(7,13- pent-4-yn-1yloxy)benzo[*f*]naphtho[1,2-*b*]oxocin-8-one (**12**)...... S76
8 NMR spectra........ S80
9 References S131

1. General information

All reagents were used in analytical grades and without further purification. Ethyl acetate and cyclohexane were purchased in technical grade and purified by distillation prior to use. Anhydrous solvents were purchased pre-dried and treated by a solvent purification system (SPS-5, M. Braun Incorporated, Stratham, USA). Milli-Q™ water was obtained using Simplicity™ System (UV) (Merck KGaA, Darmstadt, Germany) for chromatography purposes.

Chromatography

Crude reaction mixtures and purified products were analyzed with **TLC** (thin layer chromatography on Silica gel 60 F254, Merck KGaA, Darmstadt, Germany) and **HPLC** (high performance liquid chromatography). A modular system LC-20A Prominence, UV/VIS detector SPD-20A/AV (λ = 254 nm), and LCMS-2020 Single Quadrupole (all from Shimadzu Deutschland GmbH, Duisburg, Germany) was used. An Eurospher II 100-5 C-18-column (Knauer Wissenschaftliche Geräte GmbH, Berlin, Germany) column (length of 150 mm, diameter of 4 mm, pore size of 100 Å, particle size 5 μm) was used for separation. It operated with acetonitrile, and water with 5% (v/v) acetonitrile and formic acid (1 mL/L) as eluents.

Gas chromatographic analyses of product mixtures and pure substances were carried out using the GC-2030 gas chromatograph. A quartz capillary column HI-5MS (Avantor VWR, Radnor, USA) with 30 m, 0.25 mm inner diameter, a 0.25 μm thickness of the covalently bonded stationary phase with ((5% phenyl)dimethylsiloxane) using hydrogen as carrier gas at constant rate of 40 cm/s with a flame ionization detector (FID) was used. The injector temperature is set at 270 °C with detector temperature at 320 °C running a program with 50 °C start temperature for 1 min, heating rate at 17.5 °C/min and 300 °C as end temperature for 4.71 min.

Gas chromatographic mass spectra (**GCMS**): The analysis was performed on a GCMS-QP2010SE (Shimadzu, Kyoto, Japan) equipped with an electron ionisation (EI) source and a quadrupole mass analyser. HI-5MS quartz capillary column (Avantor VWR, Radnor, USA) with a length of 30 m, 0.25 mm inner diameter and a stationary phase ((5% phenyl)dimethylsiloxane) of 0.25 μm thickness was used. Helium is the carrier gas at a constant velocity of 30 cm/s. The temperature started at 50 °C (held for 1 min) and was heated to 300 °C (held for 4.71 min) with a temperature rise of 17.5 °C/min

The purification of the crude products was done by preparative column chromatography on silica gel 60 M (0.040 – 0.063 mm, Macherey-Nagel GmbH & Co, Düren, Germany) combined with a Sepacore™ system with a Büchi Control Unit C-620, Büchi Pump Modules C-605, a UV detector Büchi UV photometer C-635, and Büchi Fraction Collector C-660 (Büchi-Labortechnik GmbH, Essen, Germany) and two pump model C-605 (Büchi-Labortechnik GmbH, Essen,

Germany). The isolation of the derivatives was conducted with reversed phase column chromatography on Sepacore^{TM} C18 (HP-F0080, Büchi-Labortechnik GmbH, Essen, Germany) using MillQ^{TM} water and acetonitrile as eluents.

X-ray Crystallography

Crystal structures were measured on a STOE STADIVARI or STOE IPDS 2T (STOE & Cie GmbH, Darmstadt, Germany) using a Cu or Mo source with graphite tube monochromator. The respective compound was dissolved in $CDCl_3$ and slow evaporating at room temperature resulted in crystal formation.

Spectroscopy and spectrometry

^{1}H NMR and ^{13}C NMR spectra were recorded at 25 °C by using a Bruker Avance III HD 400 (^{1}H NMR (400 MHz), ^{13}C NMR (101 MHz) or Avance III 600 (^{1}H NMR (600 MHz), ^{13}C NMR (151 MHz)) (5 mm BBFO-SmartProbe with z gradient and ATM, SampleXPress 60 sample changer, Analytische Messtechnik, Karlsruhe, Germany). Chemical shifts (δ) are reported in parts per million (ppm) relative to TMS as internal standard. Deuterated solvents were used and reference according to Fulmer *et al*.[1] (e.g. deuterated chloroform: ^{1}H NMR: δ = 7.26 ppm, ^{13}C NMR: δ = 77.2 ppm). For further assignment of signal 2D methods such as ^{1}H,^{1}H COSY, ^{1}H,^{13}C HSQC, ^{1}H,^{13}C HMBC and ^{1}H,^{1}H NOESY were utilized. The signals were assigned the following abbreviations: s (singlet), d (doublet), t (triplet), dd (doublet of doublets), td (triplet of doublets), m (multiplet), q (quartet), hep (heptet). The spectra obtained were analyzed by MestReNova 14.2.0-26256 (Mestrelab Research S.L., Spain).

For mass spectra via electrospray-ionization (ESI+/ESI–) mass spectrometry using Quadrupole time-of-flight technology (QTOF) on an Agilent 6545 QTOF-MS (Agilent, Santa Clara (CA), USA) leading to mass-charge ratios (m/z) of the characterized substance. Mass calibration was carried out directly before analysis using an external standard mixture.

Cyclic voltammetry (CV)

The mechanism of the reaction was studied by cyclic voltammetry using an electrochemical glass cell equipped with a glassy carbon (with a diameter of 3 mm, Metrohm), a Pt wire and a leakless miniature Ag/AgCl (3.4 M KCl, EDAQ) as working, counter and reference electrode, respectively. The glassy carbon electrode was polished with alumina (1 µm particle size, Buehler), rinsed abundantly with Milli-Q water and dried with an Argon flow before every measurement. Cyclic voltammograms were measured using a potentiostat/galvanostat PGSTAT302N (Metrohm AG, Herisau, Switzerland) with a scan rate of 50 mV/s in 5 ml of 1,1,1,3,3,3-hexafluoro-2-propanol (HFIP) (99%, Carbolution Chemicals GmbH, St. Ingbert, Germany) containing 0.1 M of tetraethylammonium hexafluorophosphate (98%, Alfa aesar,

Haverhill, USA) and 5 mM of the corresponding molecule. Prior to the CV measurements, the electrolyte was degassed with an Argon flow for 25 min. An argon atmosphere was kept flowing over the electrolyte during the measurements.

The electrode potentials are reported with reference to the redox system ferrocene/ferrocenium ion (FcH/FcH^+).

Electrochemical setup

For the electrochemical reaction a flow cell developed by the Waldvogel group in cooperation with the mechanical workshop of the University of Mainz which are identical in design to IKA was used.[2] The flow cell is directly attached to a cryostat for temperature control via the cathodic part.[3] The setup is equipped with a peristaltic pump (REGLO Digital MS-2/12, ISMATEC). The distance between the electrodes is set using a corresponding Teflon™ spacer (d = 0.25 mm). Electrolysis is galvanostatic and follows a cyclic protocol. The anode area is 12 cm^2. Isostatic graphite is used as the anode material and stainless steel as the cathode material. Electrochemical reactions were carried out using a four-channel galvanostat HMP4040 (Rohde & Schwarz, München, Germany) as DC power source. In our experiments the electrolyte is pumped through the cell multiple times (cyclic flow rate: multiplier × single pass flow rate) from a single reservoir. After each electrolysis the flow cell was rinsed with 5 mL acetone and 5 mL dichloromethane (flow rate: 5 mL/min) and emptied and dried by air flow. After disassembling the cell both electrodes were cleaned with acetone and the anode was sanded (sandpaper 600 followed by 1000 grid).

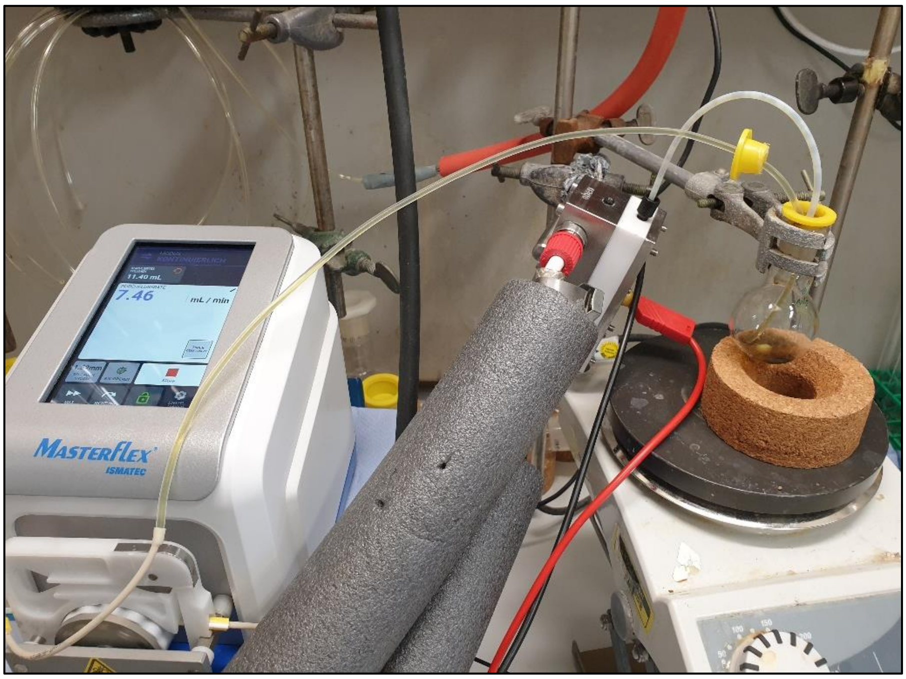

Figure S1: Set-up for the flow reaction.

2. Quantification

Quantification was performed using GC with external calibration of the starting material (**1**) and product (**2**) and each sample was taken twice to discover eventual errors during sampling. The yield was determined against the standard *n*-octylbenzene (20 µL). In the early stages of the project (chapter *4 Initial experiments*) the supporting electrolyte NBu_4PF_6 (tetrabutylammonium hexafluorophosphate) was used. We switched to NEt_4PF_6 (tetraethylammonium hexafluorophosphate) because it is more easily removed by liquid-liquid extraction and filtration on silica prior application to the GC column.

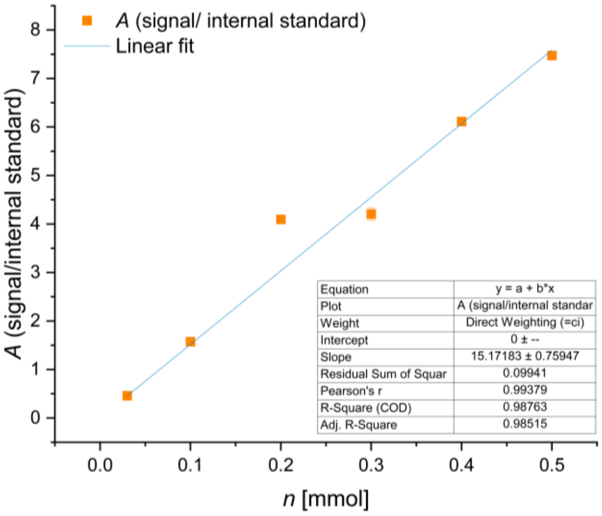

polycycle

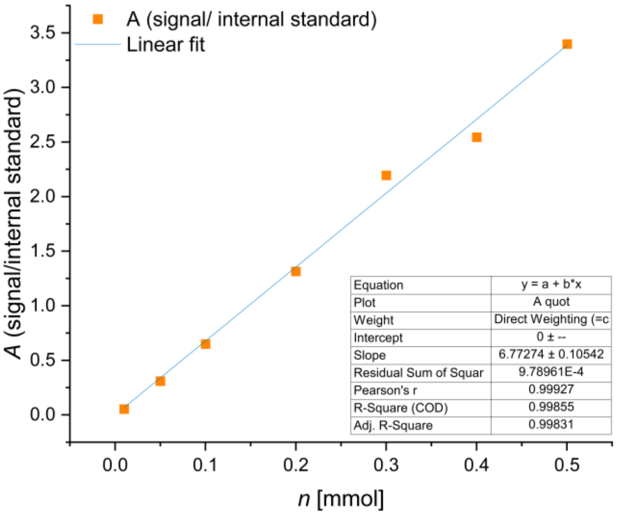

Starting material

Figure S2: Calibration curves used for GC calibration. Upper figure showes curve of the polycyclic product, the lower curve indicates the calibration for the starting material. *n*-Octylbenzene was used as an internal standard.

3. Mechanistic studies

3.1 Preliminary experiments

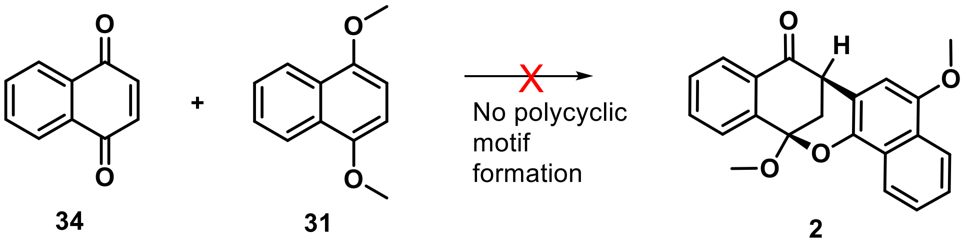

34 31 2

Figure S3: Mechanistic studies for the investigation of the necessity of a protected hydroxyl group using 1,4-naphthoquinone.

According to GP2 (chapter 5), naphthalene-1,4-dione (39.5 mg, 0.25 mmol, 1.00 equiv.), 1,4-dimethoxy-naphthalene (43.6 mg, 0.25 mmol, 1.00 equiv.) and tetraethylammonium hexafluorophosphate (17.2 mg, 0.63 mmol, 0.125 equiv.) were dissolved in 1,1,1,3,3,3-hexafluoropropan-2-ol (5.00 mL). Electrochemical parameter for this reaction: Q=60.3 C, j=5 mA/cm^2 (60 mA) and flow rate: 3.58 mL/min.

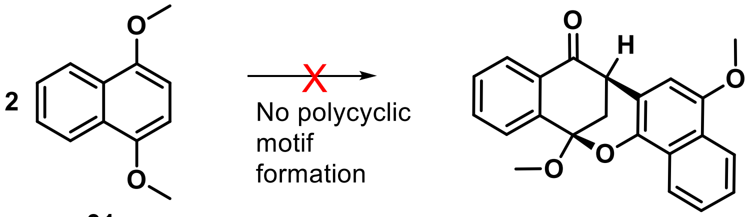

31 2

Figure S4: Mechanistic studies for the investigation of the necessity of a free hydroxyl group using 1,4-dimethoxynaphthalene.

According to GP2 (chapter 5), 1,4-dimethoxy-naphthalene (94.7 mg, 0.50 mmol, 1.00 equiv.) and tetraethylammonium hexafluorophosphate (17.2 mg, 0.63 mmol, 0.125 equiv.) were dissolved in 1,1,1,3,3,3-hexafluoropropan-2-ol (5.00 mL). Electrochemical parameter for this reaction: Q=60.3 C, j=5 mA/cm^2 (60 mA) and flow rate: 3.58 mL/min.

The crude products of each reaction were analysed with GCMS and the mass of **2** was not observed.

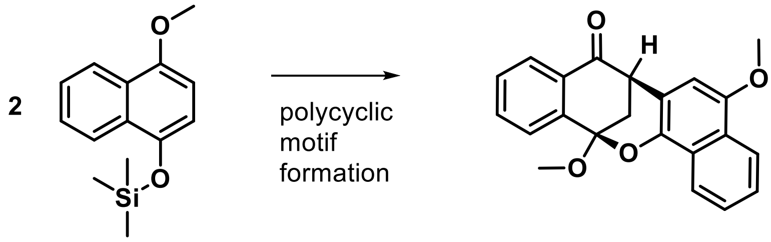

35 **2**

Figure S5: Mechanistic studies of the behavior of trimethylsilyl ethers in the formation of the polycyclic motif.

According to GP2 (chapter 5), 5-((tri-(1-methyl)silyl)oxy)naphthalen-1-ol (123.2 mg, 0.50 mmol, 1.00 equiv.) and tetraethylammonium hexafluorophosphate (17.2 mg, 0.63 mmol, 0.125 equiv.) were dissolved in 1,1,1,3,3,3-hexafluoropropan-2-ol (5.00 mL). Electrochemical parameter for this reaction: Q=60.3 C, j=5 mA/cm^2 (60 mA) and flow rate: 3.58 mL/min.

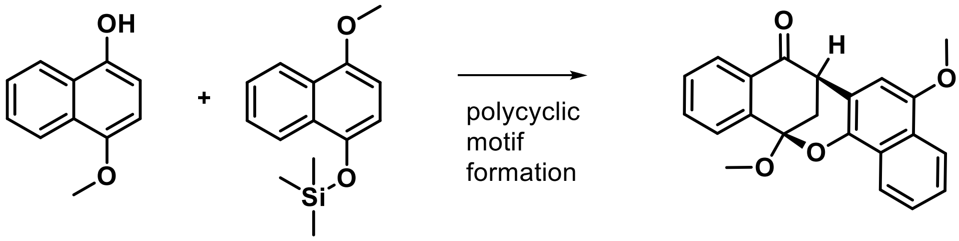

1 **35** **2**

Figure S6: Mechanistic studies of the behaviour of trimethylsilyl ethers in reaction with 1,4-dimethoxynaphthalene.

According to GP2 (chapter 5), 5-((tri-(1-methyl)silyl)oxy)naphthalen-1-ol (61.6 mg, 0.25 mmol, 1.00 equiv.) and 4-methoxy-1-naphthol (43.6 mg, 0.25 mmol, 1.00 equiv.) and tetraethylammonium hexafluorophosphate (17.2 mg, 0.63 mmol, 0.125 equiv.) were dissolved in 1,1,1,3,3,3-hexafluoropropan-2-ol (5.00 mL). Electrochemical parameter for this reaction: Q=60.3 C, j=5 mA/cm^2 (60 mA) and flow rate: 3.58 mL/min.

The crude products of each reaction were analyzed with GCMS and the mass of **2** was observed. The trimethylsilyl group (TMS) was removed in the electrolysis. For further experiments we proceeded with triisopropylsilyl (TIPS) instead of TMS as protecting group.

According to GP2 (chapter 5) the electrolysis starting from **1** was carried out in HFIP-d_2 instead of HFIP to identify the origin of the in proton in the methylene group.

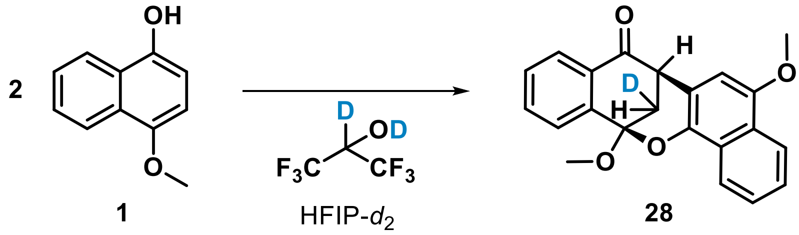

Figure S7: Mechanistic studies on the origin of the proton in the methylene bridge. Therefore, the optimized conditions towards **2** were carried out in HFIP-*d*2.

As a result, partial incorporation of deuterium into the methylene bridge was observed. The degree of deuteration was thereby determined at 66%, respective to the proton that is introduced during the reaction, via ^{1}H NMR and ^{2}H NMR. The deuteration degree was also determined to 68% using LC-MS (IsoPat[2] evaluation in excel[4]). We assume that the incomplete deuteration can be rationalized by an intramolecular protonation by the phenolic hydroxyl group. This result can be explained by the compact HFIP cage formed around the substrate due to strong hydrogen bonding interactions. Furthermore, the selective substitution at only one *dd* can be detected. Therefore, a stereoselective incorporation of the deuterium is assumed. This high selectivity is not likely due to sterically hinderance and more likely due to a rigid orientation in the solvent cage during an intramolecular attack. Both findings substantiate the proposed intramolecular protonation of the double bound of **28** (Figure 3; manuscript) by the phenolic hydroxyl group.

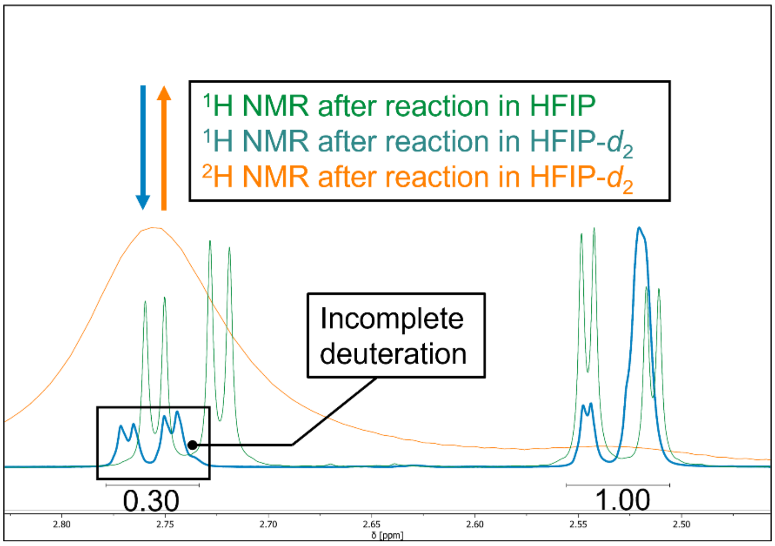

Figure S8: NMR spectra after the deuteration experiment in HFIP-d^{2} compared to the experiment in HFIP. Shown is the area of the *dd* corresponding with the two methylene bridge protons. The decrease of one of these signals can be detected in the deuteration experiment. The other signal at 2.52 ppm shows an integral of 1 compared to the other signals of the molecule which showed no change in integrals.

To investigate whether a quinone-ketal-dimer could be a possible intermediate of the reaction the corresponding quinone-ketal (**33**) has been synthesized and tested under optimized

conditions while just 0.5 *F* have been applied because of the higher oxidation state of the ketal (**29**). Because of the shorter reaction time the flow-rate was increased to remain the same flow-multiplicator.

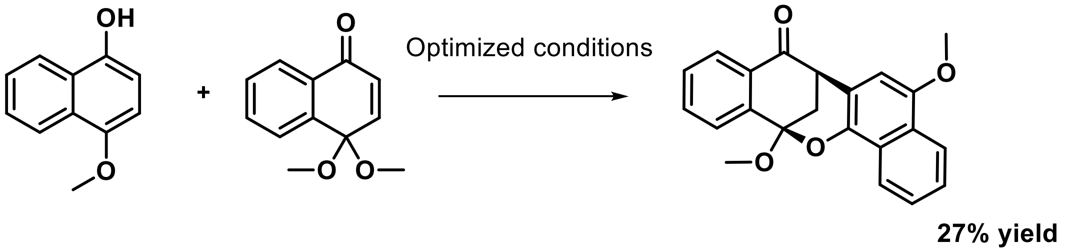

1 **33** **2**

Figure S9: Mechanistic studies of the behaviour of quinone-ketals (**29**) and 4-methoxy-naphth-1-ol (**1**) under optimized conditions.

According to GP2 (chapter 5), 4-methoxy-naphth-1-ol (65.3 mg, 0.375 mmol, 1.00 equiv.) and 4,4-dimethoxynaphthalen-1(4*H*)-one (76.6 mg, 0.375 mmol, 1.00 equiv.) and tetraethylammonium hexafluorophosphate (16.4 mg, 0.060 mmol, 0.15 equiv.) were dissolved in 1,1,1,3,3,3-hexafluoropropan-2-ol (5.00 mL). Electrochemical parameter for this reaction: Q=36.15 C, j=25 mA/cm^2 (300 mA), T = 10°C and flow rate: 14 mL/min.

The decrease of yield shows that the quinone-ketal is no intermediate of the reaction.

As a control, we conducted the same reaction without electrolysis and supporting electrolyte. Therefore, we mixed 4-methoxy-naphth-1-ol (13.6 mg, 0.187 mmol, 1.00 equiv.) and 4,4-dimethoxynaphthalen-1(4*H*)-one (15.32 mg, 0.187 mmol, 1.00 equiv.) in 1,1,1,3,3,3-hexafluoropropan-2-ol (1.00 mL) and stirred it for 12 h. No formation of the polycycle can be detected via GC-MS.

3.2 CV studies

3.2.1 CV of 4-methoxy-1-naphthol (**1**), 5-((tri-(1-methylethyl)silyl)oxy)naphthalen-1-ol (**15**), 4-acetoxy-1-naphthol (**16**) and 7H,13H-5,13-dimethoxy-7,13-methanobenzo[*f*]naphtho[1,2-b]oxocin-8-one (**2**).

The potential (E) was determined using ferrocene (FcH/FcH$^+$). The CVs were measured starting from the open circuit potential (approximately 0 V vs FcH/FcH+ or when the j = 0 mA/cm^2) to more cathodic potentials (up to +1 V vs FcH/FcH+) in the first scan, and the switching potential was +1 V vs FcH/FcH+. The reverse scan was measured from the switching potential to the starting potential. Three cycles were measured in every experiment. The base electrolyte is to be equated with blank. The CV plotting convention proceeded by IUPAC and all the CV were measured at room temperature. HFIP containing 0.1 M of tetraethylammonium hexafluorophosphate and 5 mM of the corresponding molecule was deoxygenated by argon bubbling for 25 min.

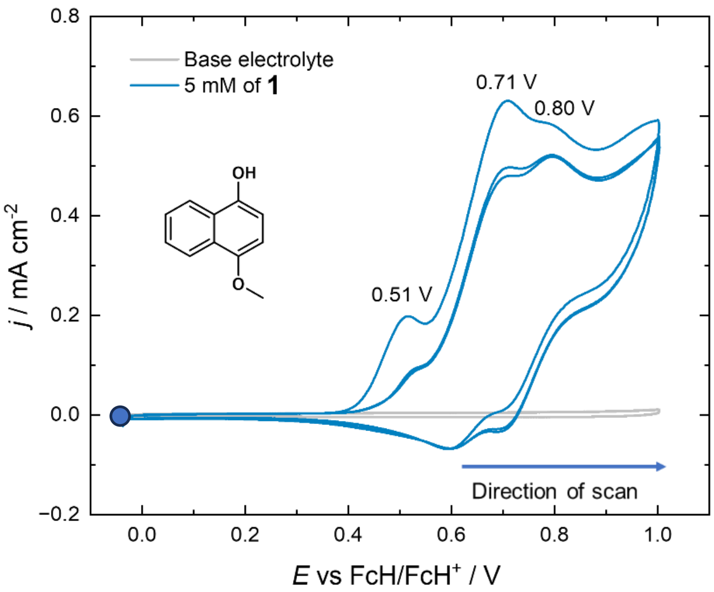

Figure S9: CV of 0.5 mM 4-methoxy-1-naphthol (**1**) and base electrolyte 0.1 M of NEt_4PF_6 both in HFIP at room temperature. The reverse scan was measured from the switching potential to the starting potential. The starting point of the scan is indicated by a blue dot.

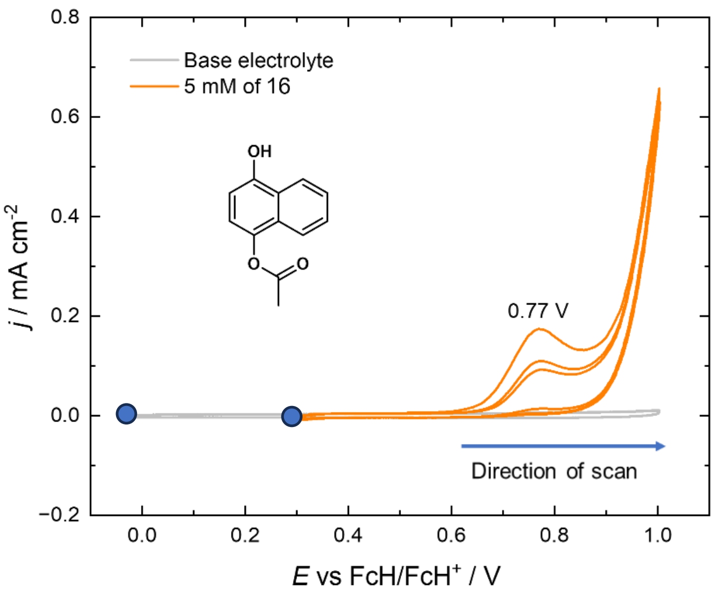

Figure S10: CV of 0.5 mM 5-((tri-(1-methylethyl)silyl)oxy)naphthalen-1-ol (**16**) and base electrolyte 0.1 M of NEt_4PF_6 both in HFIP at room temperature. The reverse scan was measured from the switching potential to the starting potential. The starting point of the scan is indicated by a blue dot.

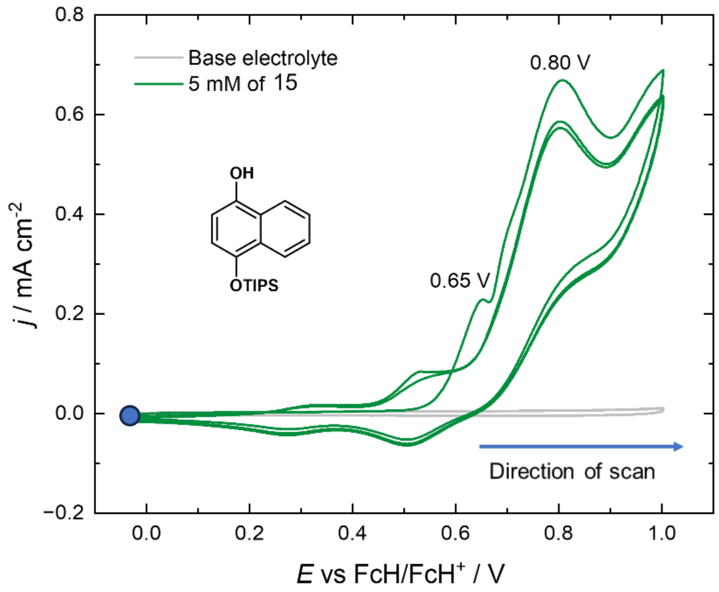

Figure S11: CV of 0.5 mM 4-acetoxy-1-naphthol (**15**) and base electrolyte 0.1 M of NEt_4PF_6 both in HFIP at room temperature. The reverse scan was measured from the switching potential to the starting potential. The starting point of the scan is indicated by a blue dot.

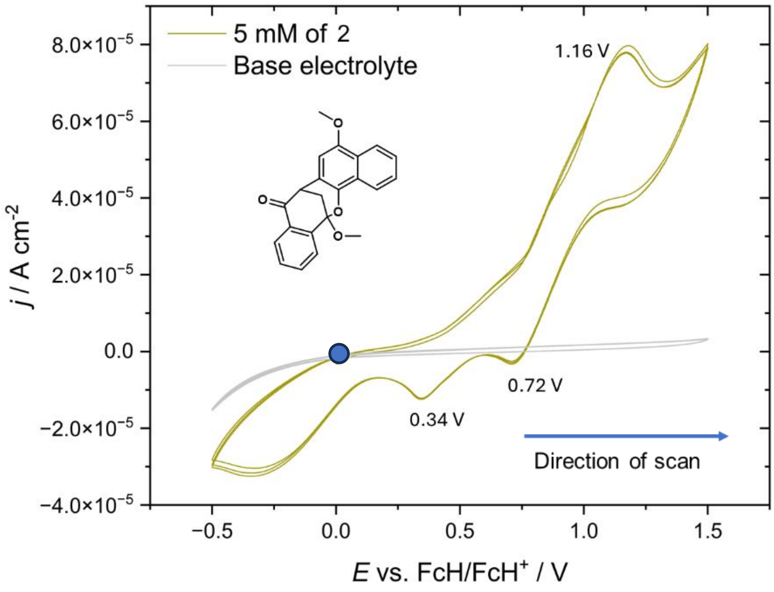

Figure S12: CV of 0.5 mM 7*H*,13*H*-5,13-dimethoxy-7,13-methanobenzo[*f*]naphtho[1,2-*b*]oxocin-8-one (**2**) and base electrolyte 0.1 M of NEt_4PF_6 in HFIP at room temperature. The reverse scan was measured from the switching potential to the starting potential. The starting point of the scan is indicated by a blue dot.

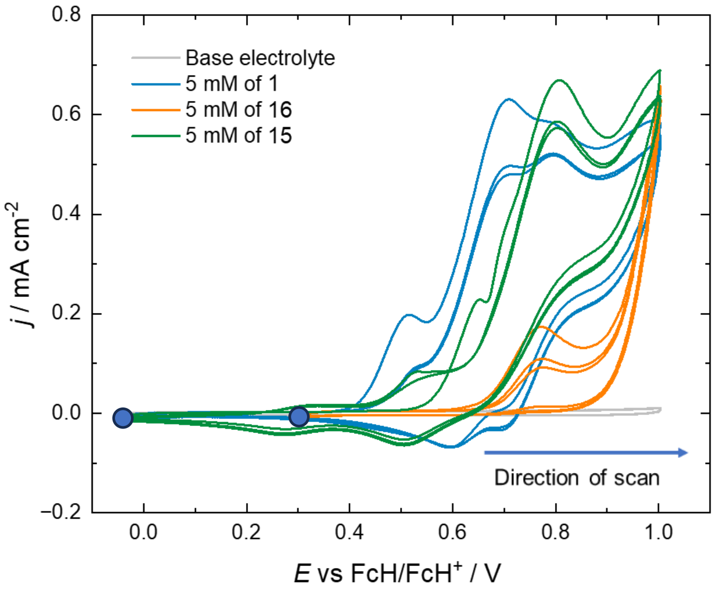

Figure S13: Stacked CV of 0.5 mM (**1**) highlighted in blue, 0.5 mM (**16**) highlighted in orange and 0.5 mM (**15**) highlighted in green and base electrolyte 0.1 M of NEt_4PF_6 all in HFIP at room temperature. The reverse scan was measured from the switching potential to the starting potential. The starting point of the scan is indicated by a blue dot.

The peak current density and the concentration are proportional in a cyclic voltammogram. The concentration of the three molecules (**1**), (**16**) and (**15**) was the same (5 mM). The molecule (**1**) (highlighted in blue) and (**15** in green) have very similar oxidation peak current density. However, the molecule (**16** in orange) has a higher oxidation potential (lower peak current density) compared to (**1**) and (**15**) and higher potential is necessary for oxidation.

4. Optimization data

4.1 Initial experiments

For these initial experiments the quotient of GC area of the naphthol (**1**) and the polycycle (**2**) were compared to an internal reference using equal aliquots from the reaction solution. The electrolysis was carried out in batch-type cells. Initial electrosynthetic screening was conducted to determine the optimal choice of electrode material and electrolyte system, in conjunction with the amount of applied charge. If the respective parameter was not varied Q=0.5 *F*, graphite was used as anode and 0.1 M tributyl methyl ammonium methyl sulfate (MTBS) in HFIP was used as supporting electrolyte.

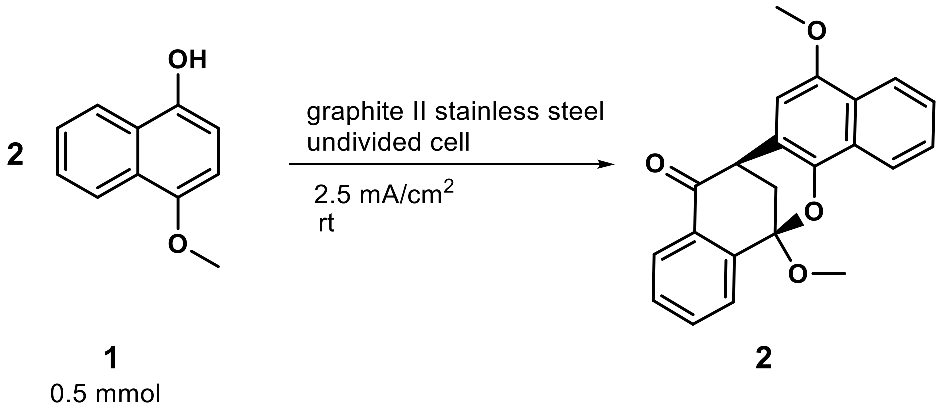

Scheme S1: Initial screening of anode material, *Q,* and supporting electrolytes.

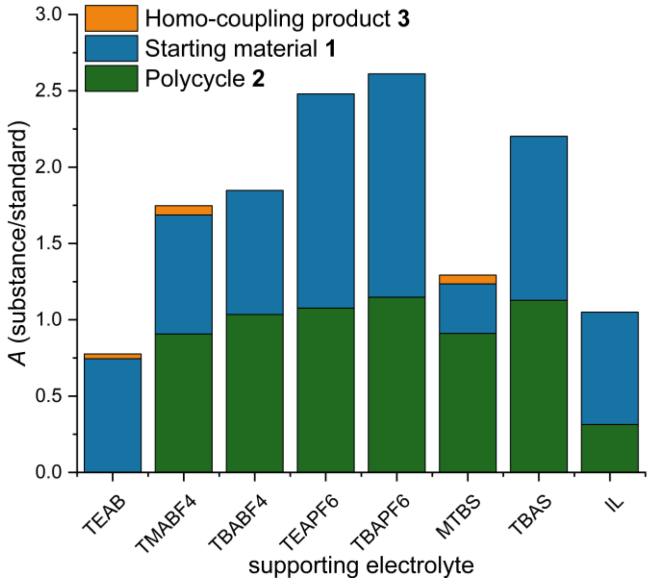

Figure S14: Results of the initial screening of supporting electrolytes. Quotient of GC integrals of homo-coupling product (**3**), starting material (**1**) and polycycle (**2**) against an internal standard is shown. IL = Butyl-3-methyl-imidazolium-hexafluorophosphate, TEAB=Tetraethylammonium bromide, TMA=Tetramethylammonium, TBA= Tetrabutylammonium, TEA=Tetraethylammonium, TBAS = Tetrabutylammonium sulfate.

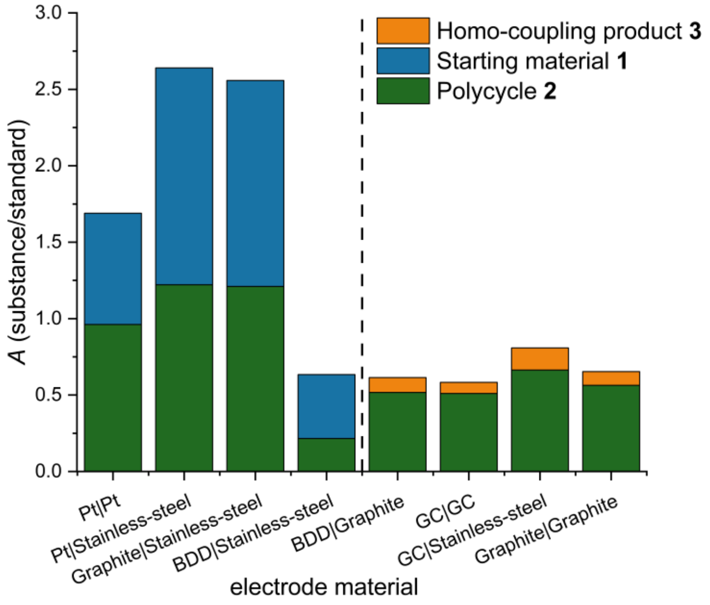

Figure S15: Results of the initial screening of electrodes. Quotient of GC integrals of homo-coupling product (**3**), starting material (**1**) and polycycle (**2**) against an internal standard is shown. Before the dashed line 1 mmol starting material was used for the screening after the line 0.5 mmol was used.

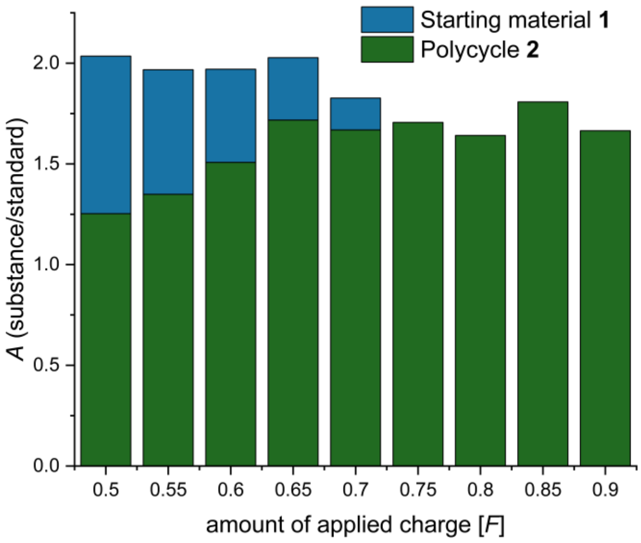

Figure S16: Results of the initial screening of applied charge. Quotient of GC integrals of starting material (**1**) and polycycle (**2**) against an internal standard is shown. The homo-coupling product (**3**) was not found in any reaction in this screening.

Platinum and graphite as anodic materials in combination with stainless steel as cathode led to an enhanced formation of **2**. Due to better availability and lower costs, we continued with graphite as anode. The best yield was achieved with NBu_4PF_6 as supporting electrolyte.

4.2 Overview of the optimization process

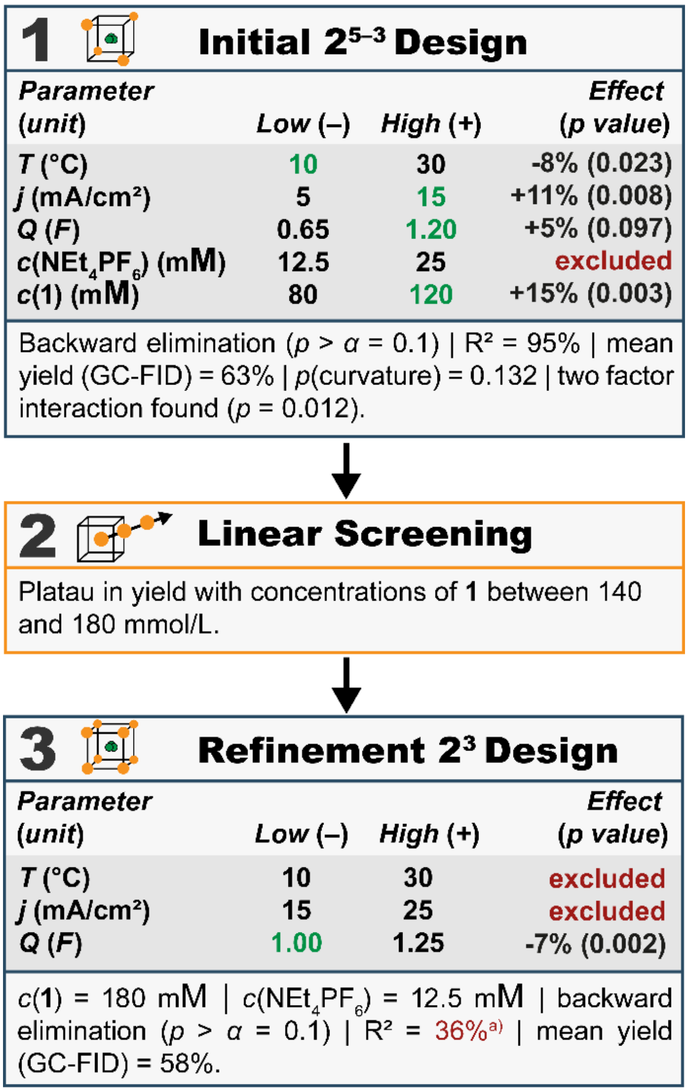

Figure S17: Optimization process and optimized condition of the polycycle (**2**) formation.

The starting point of the optimization was 37% isolated yield (conditions: NBu_4PF_6 (12.5 mM) in HFIP, 3.58 mL/min in cyclic flow with 12 as flow multiplicator, 5 mA/cm^2, 1 *F*, 10 °C at C_{iso}llstainless-steel). Two additive screening series with water (0.75vol%, 1.5vol%, 3vol%, 4.5vol%) and methanol (0.25vol%, 0.50vol%, 0.75vol% and 1vol%) decreased the yield of (**2**). For the optimization, a 2^{5-3} fractional factorial design investigated the following parameters: temperature, current density, amount of applied charge, concentration of supporting electrolyte, and concentration of the starting material (**1**). Preliminary studies have shown that the flow rate has no significant influence and can therefore be excluded for this design. The model with R^2=95% and α=0.1 issued the *c*(**1**), *j*, *T* and a two-factor interaction (*T* and *Q* or *c*(**1**) and *c*(NEt_4PF_6)) as significant parameters where low *T* and high *j*, *Q* and *c*(**1**) are presumably beneficial for the yield. Based on this, a steepest ascent, screening without the

two-factor interaction was carried out. The leading parameter which was increased stepwise was the *c*(**1**).

A full factorial DoE with the optimized *c*(**1**) of 0.15 mol/L investigated the same range of *T* and *Q* as in the first DoE but higher *j* (15–25 mA/cm^2 instead of 5–15 mA/cm^2) to enhance space-time yield. In the ANOVA (backward elimination, α=0.1), the temperature and the current density were identified as non-significant. The model (R^2=33%) shows a variance in the range of the cumulative measurement error (mean standard deviation 5%). We omitted further replication of the design and assumed the two main effects to be non-significant in the analyzed parameter range. The optimized conditions, based on the findings from the fractional factorial design, **1** was isolated with 69% isolated yield. A final current density screening (with 5 mA/cm^2 steps) did not increase the yield any further.

4.3 Choice of parameter range

Table S1: Parameter range for the DoE (2^{5-3} *fractional factorial design)*.

**Parameter**	**Low (−)**	**High (+)**
T/°C	10	30
j/mA·cm^{-2}	5	15
Q/*F*	1	1.25
c(NEt_4PF_6*)*/mmol/L	12.5	25
*c(**1**)*/mol/L	0.08	0.12

4.4 ANOVA and linear regression regarding yield of (**2**)

This is a 2^{5-2} fractional factorial design. The center point was repeated two times following a reduced model:

Table S2: Coded Coefficients (2^{5-3} *fractional factorial design)*.

Term	Effect	Coef	SE Coef	T-Value	P-Value	VIF
Constant		62.500	0.915	68.27	0.000	
T/°C	−7.25	−3.62	1.02	−3.54	0.024	1.00
j/mA/cm^2	8.1	4.10	1.02	4.01	0.016	1.00
Q/*F*	2.13	1.06	1.02	1.04	0.357	1.00
c(**1**)/mol/L	12.86	6.43	1.02	6.28	0.003	1.00
(T*Q)	6.35	3.17	1.02	3.10	0.036	1.00

Coef: Coefficient, **SE Coef**: Standard error of the coefficient.

Table S3: Model summary (2^{5-3} *fractional factorial design)*.

S	R²	R²(adjusted)	R-² (predicted)
2.89498	95.17%	89.13%	75.18%

Table S4: Analysis of Variance (2^{5-3} *fractional factorial design)*.

Source	DF	Adj SS	Adj MS	F-Value	P-Value
Model	5	660.213	132.043	15.76	0.010
Linear	4	579.688	144.922	17.29	0.009
T/°C	1	104.994	104.994	12.53	0.024
j/mA/cm^2	1	134.755	134.755	16.08	0.016
Q/*F*		9.060	9.060	1.08	0.357
c(**1**)/mol/L	1	330.878	330.878	39.48	0.003
2-factor interaction	1	80.525	80.525	9.61	0.036
(T*Q)	1	80.525	80.525	9.61	0.036
Error	4	33.524	8.381		
Curvature	1	11.929	11.929	1.66	0.288
Lack-of-Fit	2	8.522	4.261	0.33	0.778
Pure Error	1	13.072	13.072		
Total	9	693.736			

* linear fit was used for non-linear response curve

Table S5: Regression equation in uncoded units (2^{5-3} *fractional factorial design)*.

Yield (%)	=	76.9 – 3.218 *T*/°C + 0.821 *j*/mA/cm^2 – 42.2 *Q*/*F* +321.6 *c*(**1**)/mol/L + 2.538 (T*Q)

Graphical visualisation of the model

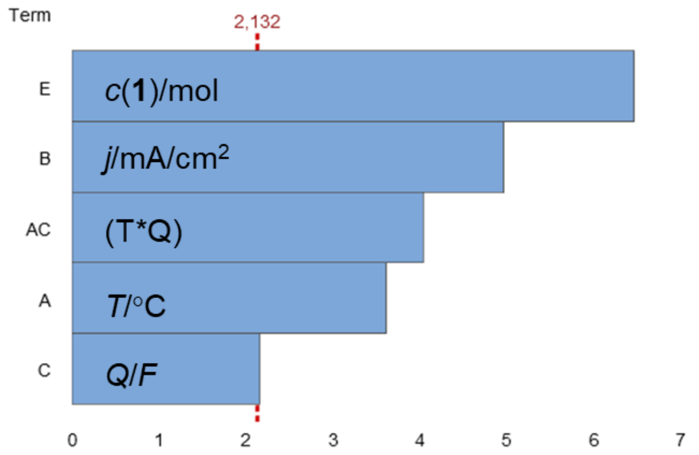

Figure S18: Pareto chart of the standardized effect of the factors A – C, E and (A · C) and residual plot regarding the yield (2^{5-3} *fractional factorial design)*.

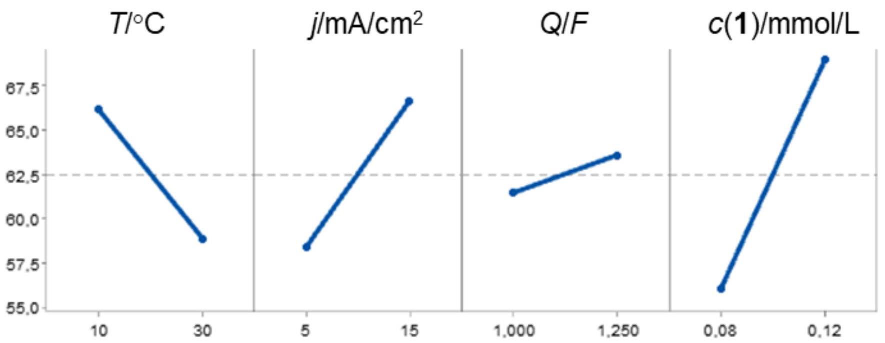

Figure S19: Main effects plot regarding the yield (2^{5-3} *fractional factorial design)*.

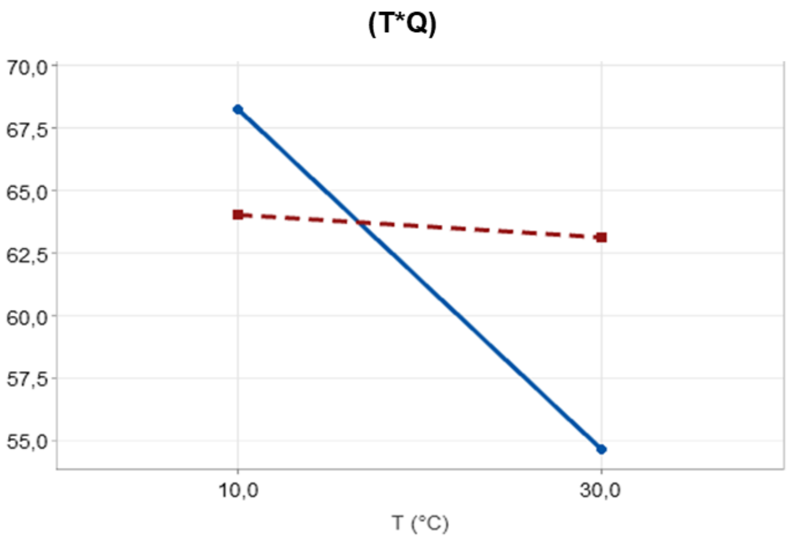

Figure S20: Two-factors interaction [AC] (2^{5-3} *fractional factorial design)*.

Table S6: Detailed information including center point, the five parameters and the yield and lost 3 (2^{5-3} *fractional factorial design)*.

**Default order**	**Pass order**	**Central point**	**Block**	***T*/°C**	***j*/mA/cm^2**	***Q*/*F***	***c*(NEt$_4$PF$_6$)/mol/L**	***c*(1)/mol/L**	**Yield**	**Yield FID1**	**Lost 1**
3	1	1	1	10	5	1.000	25.00	0,12	70	69.6	22.4
9	2	0	1	20	10	1.125	18.75	0,10	69	67.2	22.7
5	3	1	1	30	5	1.250	12.50	0,08	51	50.6	45.7
8	4	1	1	30	15	1.250	25.00	0,12	79	74.5	16.6
7	5	1	1	10	5	1.250	12.50	0,12	66	66.2	34.3
10	6	0	1	20	10	1.125	18.75	0,10	63	62.1	29.5
6	7	1	1	10	15	1.250	25.00	0,08	62	60.7	34.0
1	8	1	1	30	5	1.000	25.00	0,08	39	45.0	53.3
2	9	1	1	10	15	1.000	12.50	0,08	67	65.8	19.0
4	10	1	1	30	15	1.000	12.50	0,12	63	63.2	41.5

4.5 Steepest ascent screening

For the steepest ascent the concentration of **1** was chosen as leading parameter because it had the largest absolute regression coefficient $|\beta_j|$. The chosen step size $\Delta x_1$was 0.01. The step size in the other variables is $\Delta x_1 = \frac{\hat{\beta}_i}{\hat{\beta}_i/\Delta x_1} i = 1,2,\ldots,k, i \neq j$. Two factor interactions are excluded in this model.

Table S7: Parameter overview of steepest ascent screening.

	***T*/°C**	***j*/mA/cm^2**	***Q*/*F***	**[AC]**	***c*(1)/mol/L**
$|\beta_j|$.	–4.51	1.12	–53.4	3.647	364.7

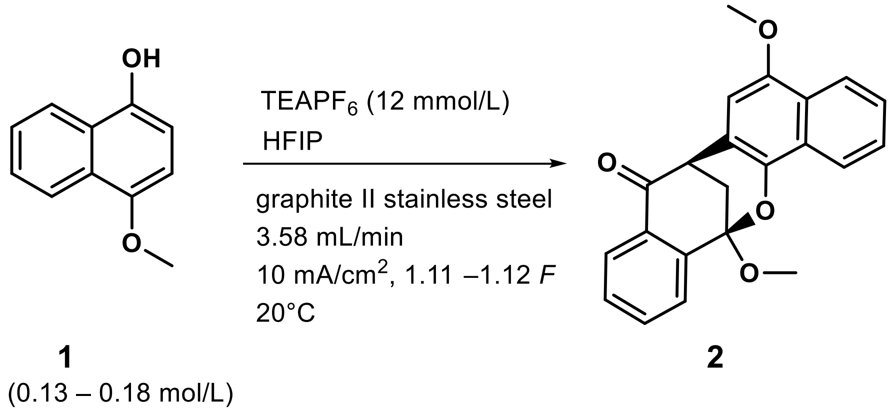

Figure S21: Reaction conditions for the steepest assent experiment.

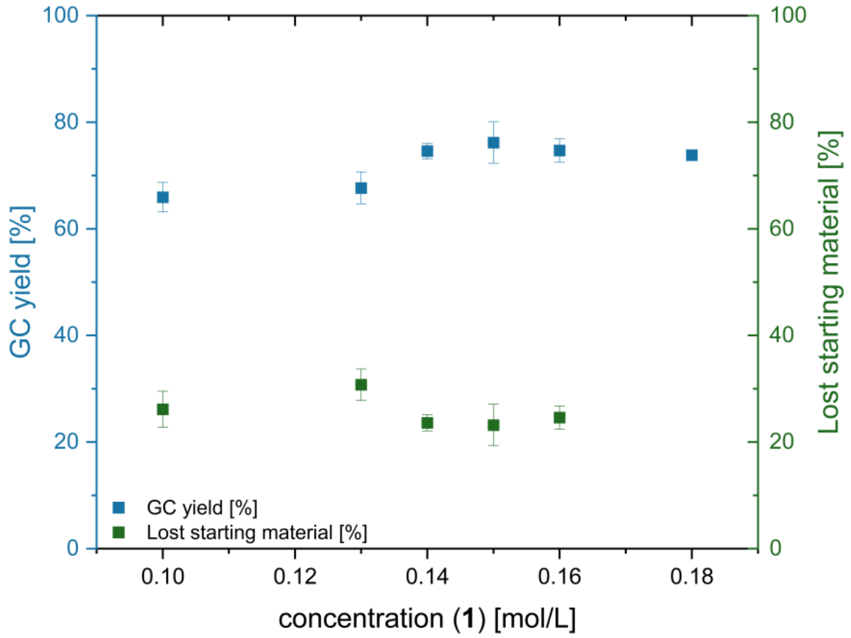

Figure S22: Results of the steepest assent. GC yield in blue, starting material which cannot be found in recovered starting material and product in green.

The highest yield was gained with a concentration of 0.15 mol/L of the starting material.

4.6 ANOVA and linear regression regarding the yield (**1**)

After the steepest ascent a second full factorial DoE 2^3 with the same parameter range regarding the temperature and the applied amount of charge but with higher current densities was carried out. The higher current density could possibly improve the time space yield. The concentration of 0.15 mol/L because of the steepest ascent screening was a fixed parameter. The concentration of the supporting electrolyte had no significant impact on the yield in the DoE before. Therefore, it was kept constant at 0.6 mmol respectively 0.12 mmol/L.

Table S8: Parameter range for the full factorial 2^3 DoE.

**Parameter**	**Low (–)**	**High (+)**
T/°C	10	30
j/mA·cm^{-2}	15	25
Q/F	1	1.25

The center point was repeated four times. A backward elimination ($\alpha = 0.1$) was carried out for the calculated regression model that derives the following reduced model:

Table S9: Coded Coefficients *(full factorial DoE 2^3).*

Term	Effect	Coef	SE Coef	T-Value	P-Value	VIF
Constant		58.521	0.860	68.04	0.000	
Q/*F*	–6.650	–3.325	0.942	–3.53	0.002	1.00

Coef: Coefficient, **SE Coef**: Standard error of the coefficient.

Table S10: Model summary *(full factorial DoE 2^3).*

S	R²	R²(adjusted)	R-² (predicted)
4.21376	36.14%	33.24%	24.44%

Table S11: Analysis of Variance *(full factorial DoE 2^3).*

Source	DF	Adj SS	Adj MS	F-Value	P-Value
Model	1	221.113	221.113	12.45	0.002
Linear	1	221.113	221.113	12.45	0.002
Q/*F*	1	221.113	221.113	12.45	0.002
Error	22	390.627	17.756		
Curvature	1	2.852	2.852	1.15	0.698
Lack-of-Fit	6	76.004	12.667	0.61	0.719
Pure Error	15	311.771	20.785		
Total	23	611.740			

* Linear fit was used for non-linear response curve

Table S12: Regression equation in uncoded units *(full factorial DoE 2^3).*

Yield (%)	=	88.45 – 26.60 *Q*/*F*

Table S13: Fits and diagnostics for unusual observations *(full factorial DoE 2^3).*

Observation	Yield [%]	Fit	Resid.	Std. Resid.
10	50.00	58.52	–8.52	–2.07 R
16	52.00	61.85	–9.85	–2.45 R

R: Large residual (Resid.: Residual, Std. Standard variation)

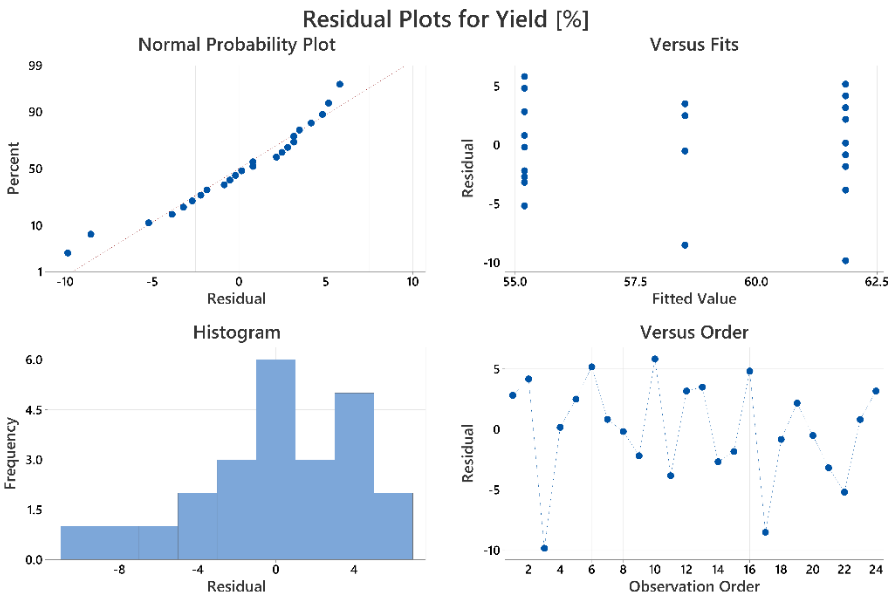

Figure S23*: Residual Plot for Yield of (***1***) (full factorial DoE* 2^3*).*

This design is not orthogonal. There was no decisive change in yield in the chosen parameter range. The chosen optimized conditions are: 10 °C, 1 *F* and 25 mA/cm^2 (corresponds to 300 mA). It aligns with the first DoE suggesting that low temperatures, high current densities, and high concentration of (**1**) are beneficial for the yield of (**2**). The high current densities and low amount of applied change leads to a very short reaction time of 4 minutes. The optimal conditions were repeated several times and led to 72% GC yield and 69% isolated yield.

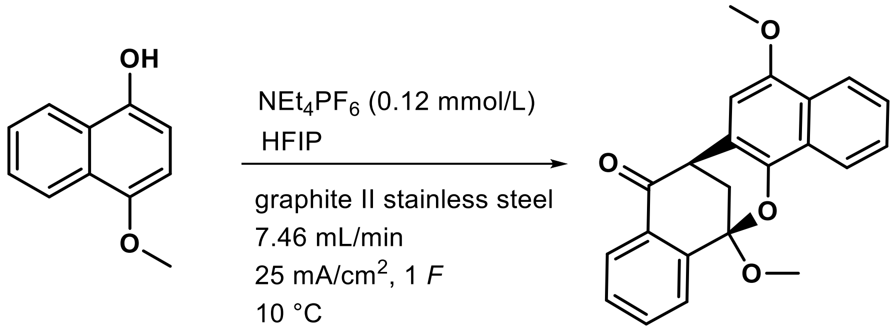

1
(0.15 mmol/L)

2

Figure S24: Optimized conditions for the polycycle synthesis of **2**.

4.5. Screening of current density

For the final screening of current density (

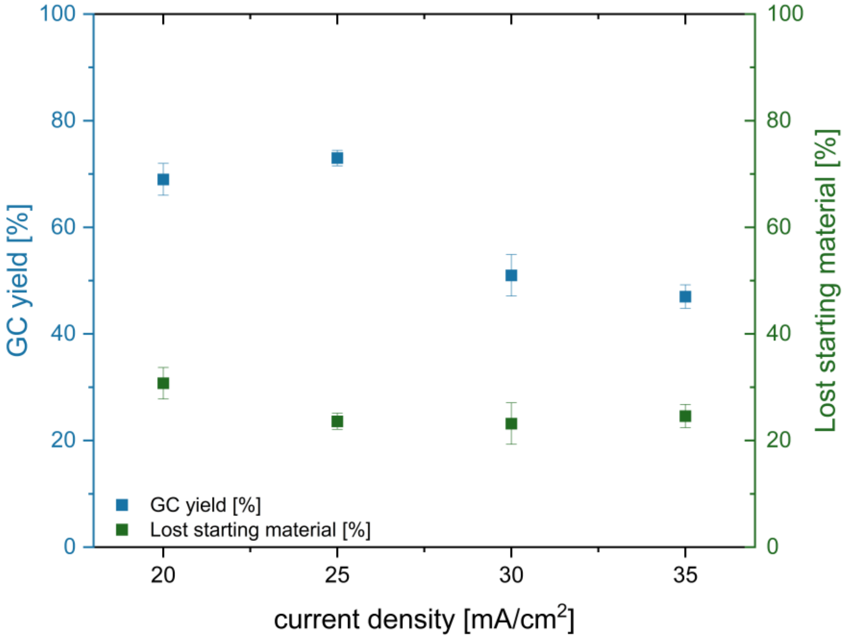

Figure S25), the current density was decreased by 5 mA/cm^2 to 20 mA/cm^2 (240 mA) and increase twice by 5 mA/cm^2 to 30 mA/cm^2 (360 mA) and 35 mA/cm^2 (420 mA) from the previous conditions.

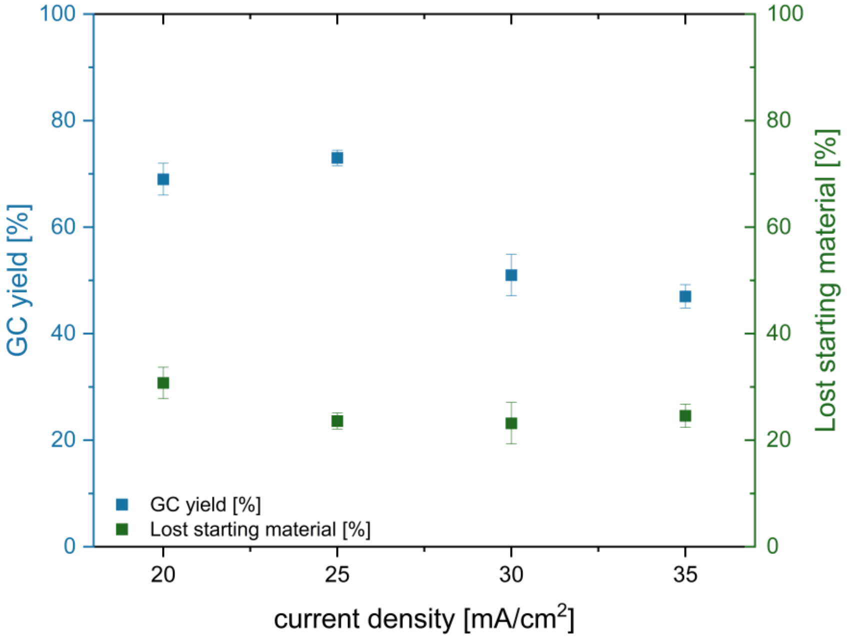

Figure S25: GC yields of **2** and lost **1** in the current density screening.

It was observed that the flow was not constant towards the end of the two reactions with the higher current densities. Presumably, the flow cell partly clogged over time due to the formation of deposits on the electrode surface (Figure S26).

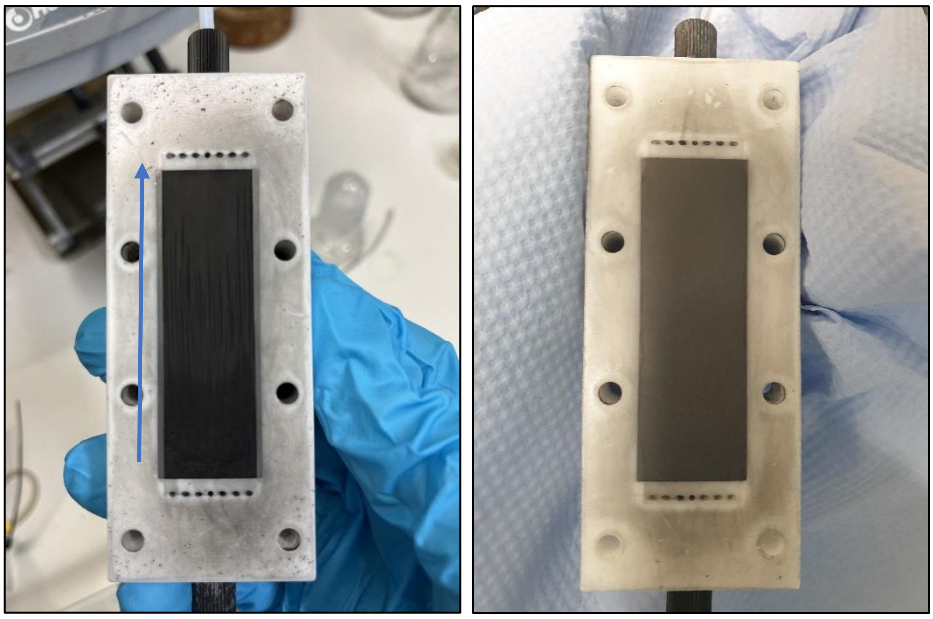

Figure S26: Graphite electrode after electrolysis with 35 mA/cm^2. Blue arrow indicates the flow direction (left-hand side) and electrode after cleaning (right-hand side).

4.6. Reusability of the electrode

The reusability of the graphite electrode was tested before the optimization. For these four experiments the electrode surface was not cleaned with solvent nor sanded after each electrolysis. The yields were determined with GC.

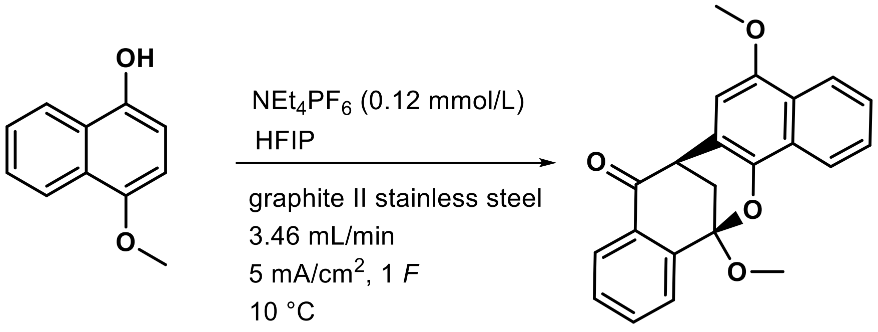

1 (0.5 mmol/L) **2**

Figure S27: Reaction conditions for the reusability experiment.

An increased yield was observed after the first and second time reusing the electrode without cleaning. This led to the conclusion that the deposit on the electrode can be beneficial until a certain point. After the third electrolysis a drop in the yield was observed. For the accountability

of each reaction the electrode was cleaned upon every electrolysis for the optimization and scope as indicated in the GP.

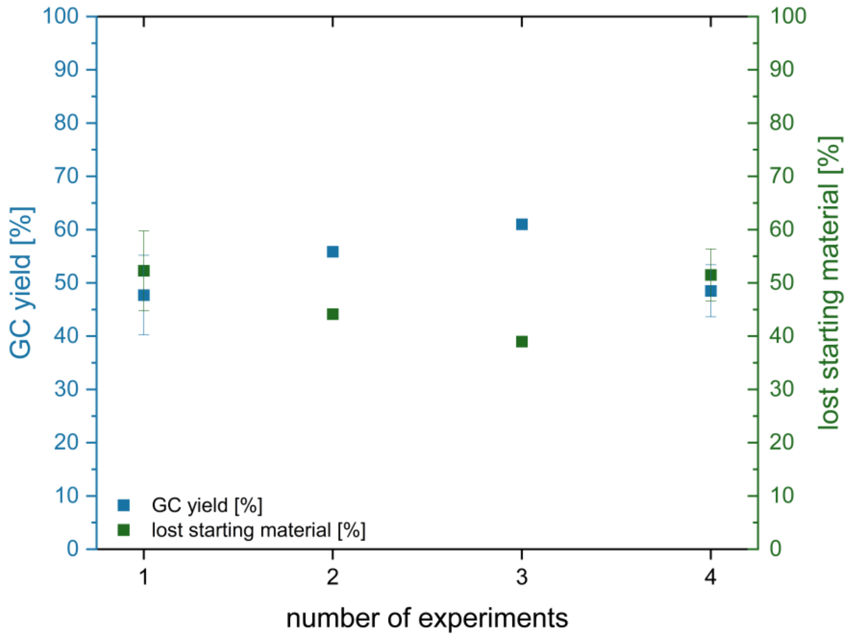

Figure S28: Change in yield and lost starting material when using the graphite electrode multiple times without cleaning. Every experiment was performed two times.

4.7. Analysis of side products

Before the systematic optimization of the reaction, side products (SP) of the reaction were analyzed to get a better insight into the reaction. Therefore, column chromatography was conducted after workup and every obtained fraction was analyzed using ^{1}H NMR and HRMS. The region of the *dd* corresponding to the methylene bridge protons and the methoxy groups were of special interest. Analysis of the data revealed that, in addition to the polycyclic product, the homo-coupling product and three oligomeric products containing the methylene bridge motif were observed. SP 1 shows the same shift of the *dd* as well as the methoxy group while SP2 and 3 have a sightly shift to low-field indicating a change in chemical environment next to this bridged motif. This could be explained by an oligomerization of the polycyclic product in proximity to the methylene bridge. We assume that the black deposit on the electrode (Figure S26) contains further overoxidation products which are insoluble to be analyzed by NMR or HRMS.

Table S14: Overview of products of the reaction before the first DoE screening. Molecular mass was determined by HRMS. For this reaction 0.5 mmol starting material (101 mg) was used.

	**Mass [mg] (Yield)**	**Shift *dd* [ppm]**	**Shift OC*H₃* [ppm]**	**Molecular mass [m/z]**
**Polycycle (2)**	32 mg (37%)	2.77–2.73 + 2.56–2.52	3.94 + 3.76	347.1277 ($M+H^+$)
**Homo-coupling product (3)**	14 mg (16%)	-	4.14	345.1132 ($M–H^-$)
**SP 1**	13 mg	2.77–2.73 + 2.56–2.52	3.94 + 3.76	-
**SP 2**	7 mg	2.92–2.91 + 2.77–2.73	4.32 + 3.82	1011.2557
**SP 3**	7 mg	2.92–2.91 + 2.77–2.73	4.32 + 3.82	1498.4058 + 1011.2562

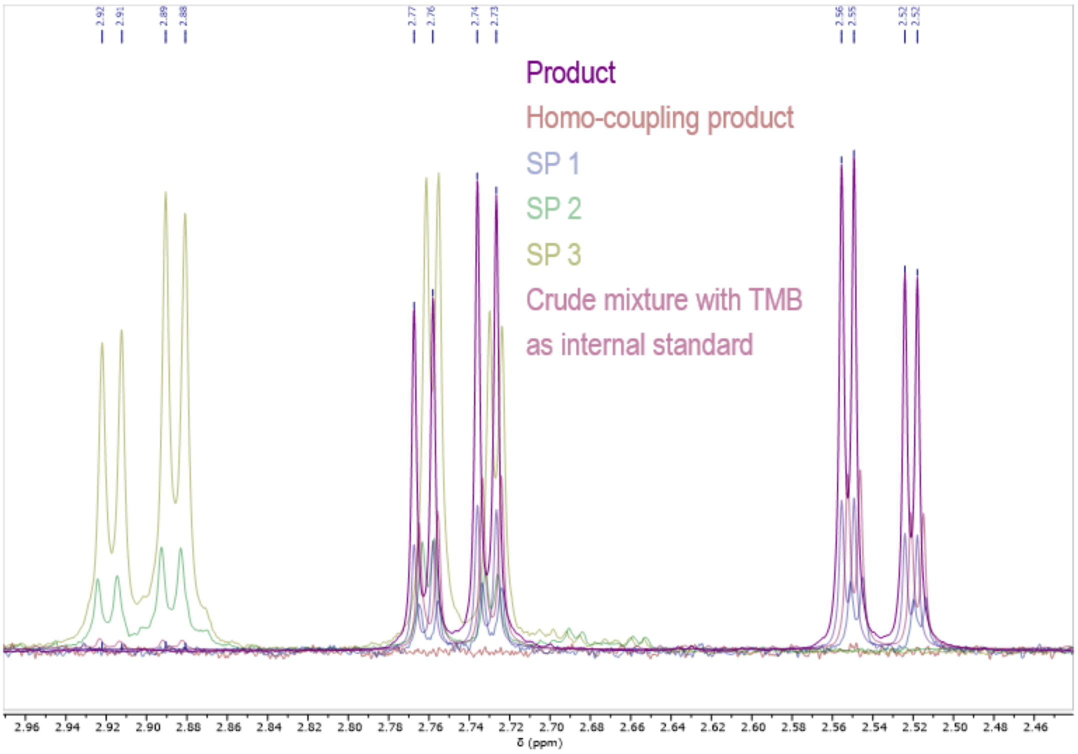

Figure S29: Comparison of the ^{1}H NMR (400 MHz, $CDCl_3$) spectra of the different isolated compounds in the region of the *dd*.

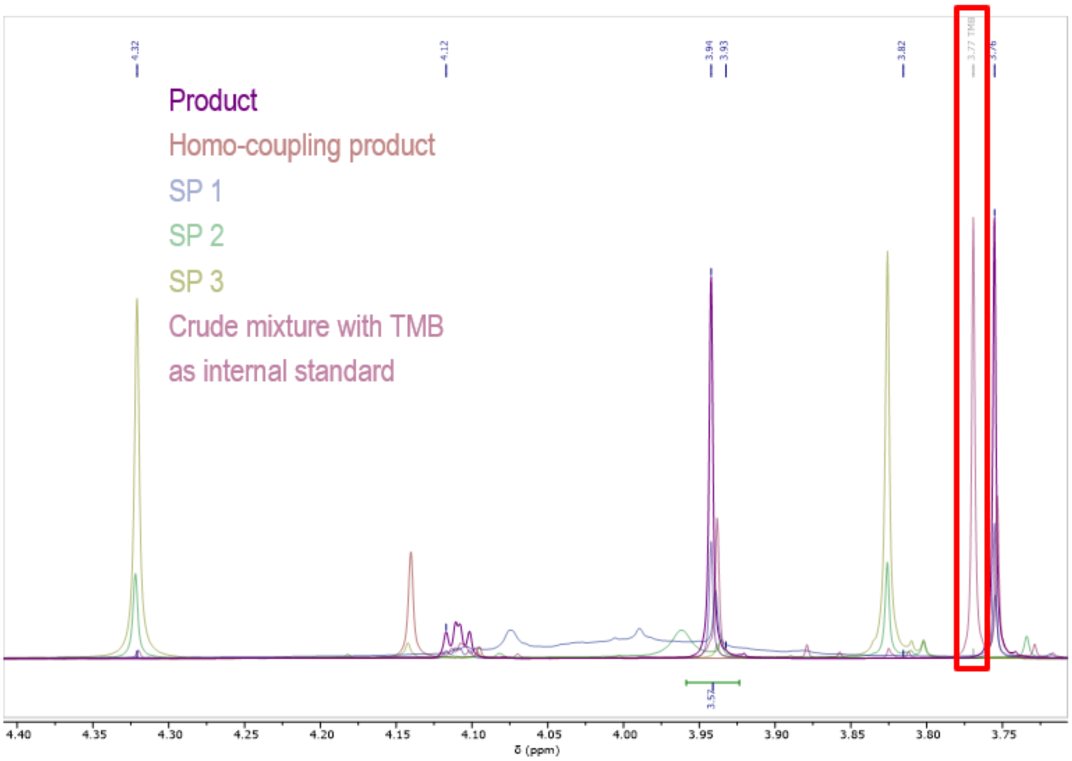

Figure S30: Comparison of the ^{1}H NMR (400 MHz, $CDCl_3$) spectra of the different isolated compounds in the typical region of methoxy groups. The signal of the methoxy group protons of 1,3,5-trimethoxybenzene (TMB), which was used as internal reference, is marked in red.

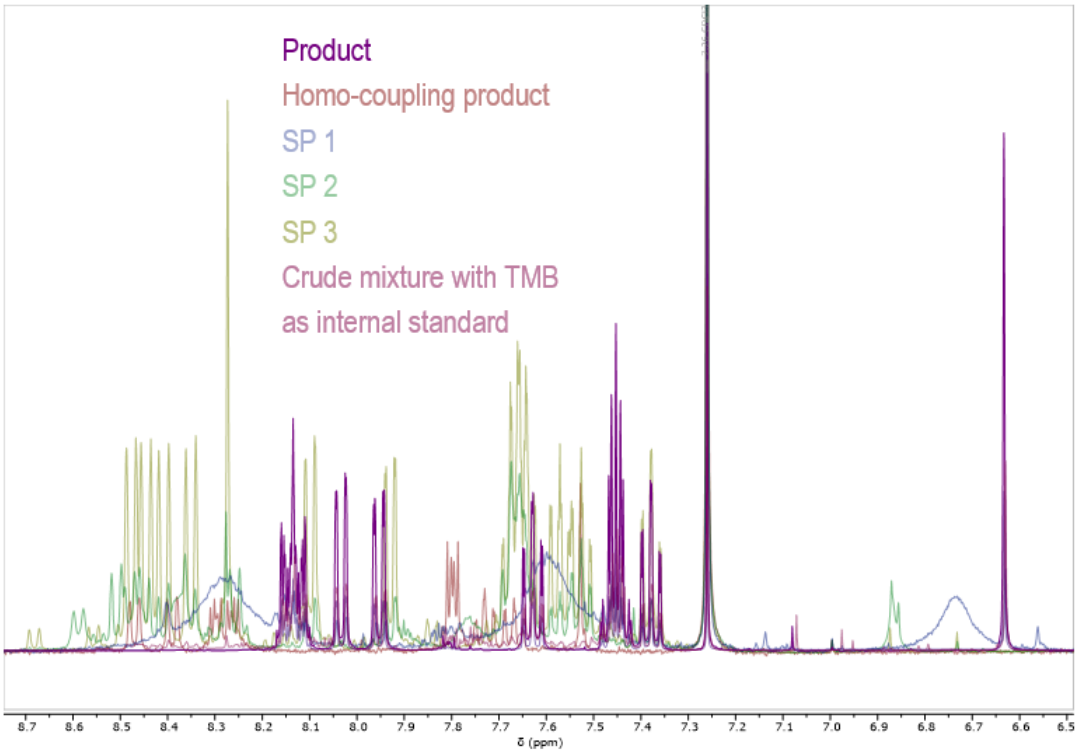

Figure S31: Comparison of the ^{1}H NMR (400 MHz, $CDCl_3$) spectra of the different isolated compounds in the aromatic region.

4.8. Cross-coupling reactions

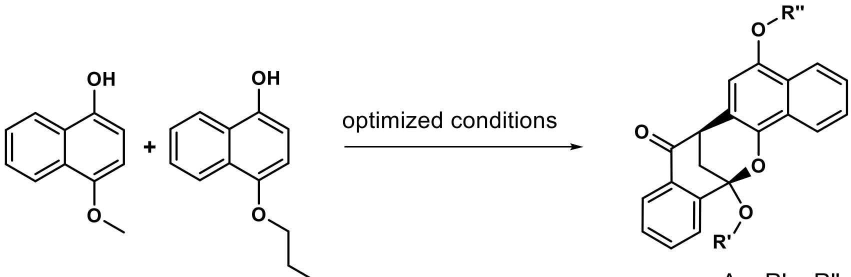

1 (0.375 mmol) **36** (0.375 mmol)

A = R' = R'' = Me
B = R' = R'' = C_3H_7
C = R' = Me, R'' = C_3H_7
D = R' = C_3H_7, R'' = Me

According to GP2 (chapter 5), 4-methoxy-naphth-1-ol (65.3 mg, 0.375 mmol, 1.00 equiv.) and 4-propoxy-naphth-1-ol (75.8 mg, 0.375 mmol, 1.00 equiv.) and tetraethylammonium hexafluorophosphate (16.4 mg, 0.060 mmol, 0.15 equiv.) were dissolved in 1,1,1,3,3,3-hexafluoropropan-2-ol (5.00 mL). Electrochemical parameter for this reaction: Q=72.3 C, j=25 mA/cm^2 (300 mA), T = 10°C and flow rate: 7.46 mL/min. The crude product was purified by column chromatography (silica: 96%→85% CH in 60 min). The A was obtained as a colorless amorphous solid (18 mg, 0.05 mmol, 15%), B was obtained as yellow amorphous solid (22 mg, 0.05 mmol, 14%), C and D were obtained as a greenish amorphous solid (45 mg, 0.12 mmol, 32%). Analysis of the GC-MS chromatogram shows that one isomer is favoured over the other. Separation of the isomers has not been achieved by normal phase column chromatography, so it can’t be determined which isomer is favoured.

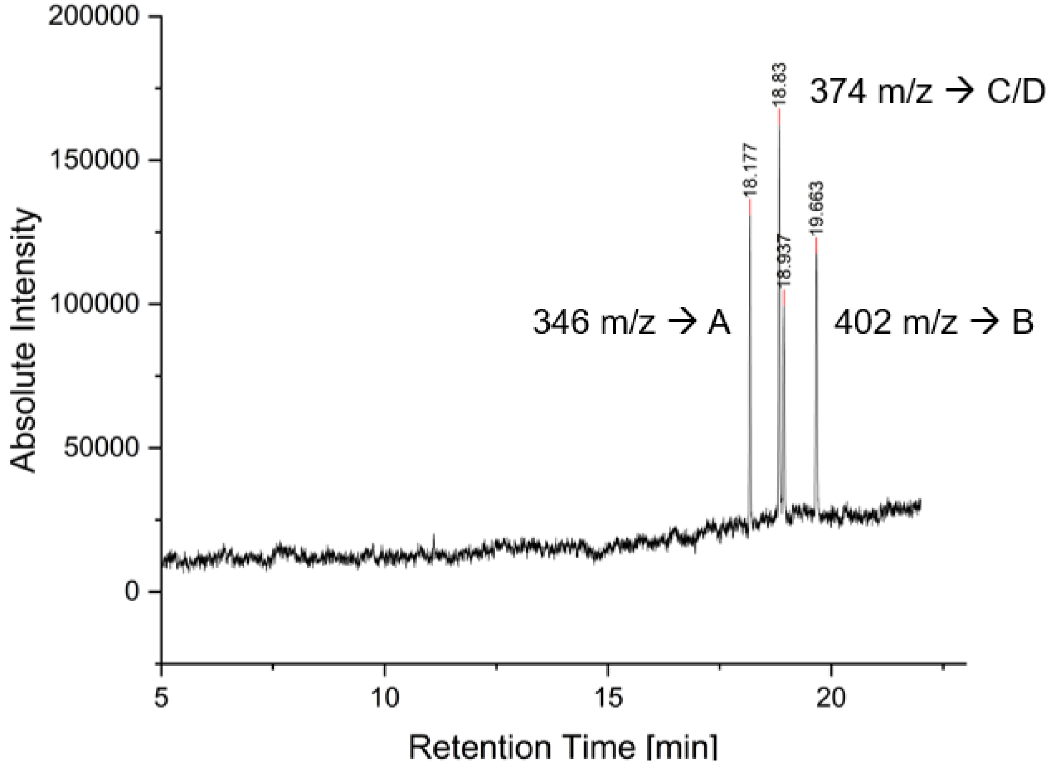

Figure S32: GC-MS chromatogram (TIC) of the crude reaction mixture of the cross-coupling experiment of 4-methoxy-naphth-1-ol (**1**) and 4-propoxy-naphth-1-ol (**36**). Detected base peaks were assigned to the corresponding products.

5. General protocols

GP1: Reduction and alkoxylation of 1,4-naphthoquinones

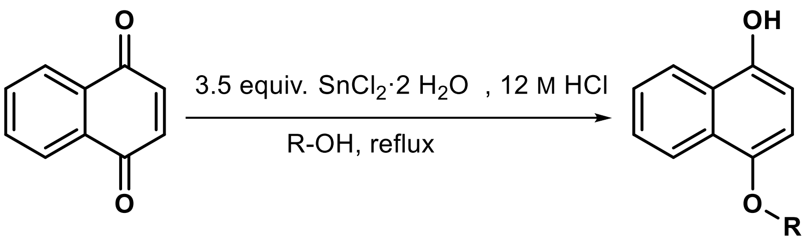

34

Figure S33: Synthesis of the alkoxylated 1,4-naphtholes described in GP1.

In a round-bottomed flask 1,4-naphthoquinone (**30**) (1 equiv., 5.00 – 20.00 mmol, 500 mM) was dissolved in the corresponding alcohol under argon atmosphere to prevent oxidation of the hydroquinone. A solution of $SnCl_2$·2 H_2O (3.5 equiv., 17.5 – 87.50 mmol, 1,75 M) in 12 M HCl (8.4 equiv.) was added over a period of 15 min. The reaction solution was stirred for 30 min at room temperature (rt.) and then heated under reflux using a heating mantle. After completion of the reaction (GC), the alcohol was removed under reduced pressure and the remaining reaction mixture was poured into 100 mL of cold water. The precipitated solid was filtered through a glass frit (pore size 4), washed with cold water (2 x 10 mL) and then dissolved in dichloromethane (2 x 20 mL). If no solids could be collected, the aqueous fraction was extracted using ethyl acetate (2 x 50 mL). The filtrate was dried over $MgSO_4$ and the solvent was removed under reduced pressure. The crude product was purified by recrystallization or collum chromatography.

This procedure was adapted from literature.[5]

GP2: Anodic polycycle synthesis

The starting material (0.75 mmol,1 equiv.) and tetraethylammonium hexafluorophosphate (16.6 mg, 0.06 mmol,0.125 equiv.) were dissolved in 5 mL HFIP. The electrolysis is started using 1 *F* and 25 mA/cm^2 (*I* = 300 mA, calculated $I = j \cdot A$, A = electrode area and j = current density). The flow rate was 7.46 mL/min. The distance between the electrodes was 0.25 mm. The temperature of the cell was 10°C. After the electrolysis the system is rinsed with 4 mL HFIP (1 mL/min) and the entire reaction solution is collected in the same flask and air is blown through the flow cell. After the cell was rinsed, the stirring bar was removed and washed with 1 mL HFIP. HFIP was recovered by distillation under reduced pressure (50 °C, 200 mbar). The crude product was purified by chromatography using silica. The color of the polycycles change depending on the solvent.

6. Synthesis of the naphthol's and polycycles

6.1 Synthesis of naphthol's

6.1.1 4-Methoxy-1-naphthol (**1**)

According to GP1 1,4-naphthoquinone (4.00 g, 25.00 mmol, 1 equiv.) was solved in methanol (50 mL). A solution of $SnCl_2{\cdot}2\ H_2O$ (18.17 g, 87.50 mmol, 3.5 equiv.) in 12 M HCl (17.5 mL) in a 250 mL round bottom flask and heated under reflux for 16 h. The crude product was crystallized from cyclohexane (45 mL) at 10 °C. The product was obtained as a colorless crystalline solid (2.423 g, 13.91 mmol, 56%).

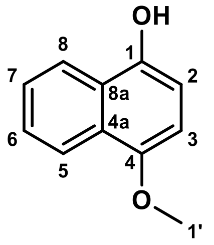

^{1}H NMR (400 MHz, $CDCl_3$) δ (ppm) = 8.25 – 8.21 (m, 1H, 5-*H*), 8.13 – 8.11 (m, 1H, 8-*H*), 7.55 – 7.49 (m, 2H, 6-*H*, 7-*H*), 6.74 (d, ^4J = 8.1 Hz, 1H, 3-*H*), 6.64 (d, ^4J = 8.1 Hz, 1H, 2-*H*), 4.92 (bs, 1H, 1-O*H*), 3.96 (s, 3H, 1'-*H*).

^{13}C NMR (101 MHz, $CDCl_3$) δ (ppm) = 149.9 (C-4), 145.1 (C-1), 126.4 (C-8a), 126.0 (C-7), 125.9 (C-6), 125.3 (C-4a), 122.1 (C-5), 121.5 (C-8), 108.0 (C-3), 103.5 (C-2), 55.9 (C-1').

Melting point (cyclohexane): decomposition starts at 123.1°C.

Spectral data correspond to literature. [5]

6.1.2 4-Ethoxy-1-naphthol (**37**)

According to GP1, 1,4-naphthoquinone (1.121 g, 7.00 mmol, 1 equiv.) and ethanol (0.81 mL, 14.12 mmol, 2 equiv.) were dissolved in acetonitrile (13 mL). A solution of $SnCl_2{\cdot}2\ H_2O$ (5.087 g, 24.5 mmol, 3.5 equiv.) in 12 M HCl (4.9 mL) was added in a 50 mL round bottom flask and heated under reflux for 28 h. The crude product was crystallized from cyclohexane (15 mL) at 10 °C. The product was obtained as a colorless crystalline solid (390 mg, 2.07 mmol, 30%). The procedure was adapted to prevent the formation of the double alkylated product, which forms as a main product, using unmodified conditions.

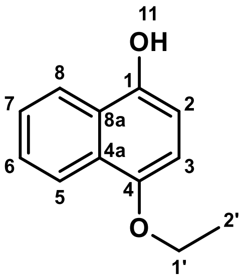

^{1}H NMR (400 MHz, $CDCl_3$) δ (ppm) = 8.28–8.23 (m, 1H, 5-*H*), 8.13–8.09 (m, 1H, 8-*H*), 7.54–7.48 (m, 2H, 6-*H*, 7-*H*), 6.72 (d, ^{3}J = 8.1 Hz, 1H, 3-*H*), 6.64 (d, ^{3}J = 8.1 Hz, 1H, 2-*H*), 4.88 (bs, 1H, 1-O*H*), 4.15 (q, ^{3}J = 7.0 Hz, 2*H*, 1‘-*H*), 1.53 (t, ^{3}J = 7.0 Hz, 3*H*, 2‘-*H*).

^{13}C NMR (101 MHz, $CDCl_3$) δ (ppm) = 149.3 (C-4), 145.0 (C-1), 126.7 (C-8a), 125.9 (C-7), 125.8 (C-6), 125.3 (C-4a), 122.3 (C-5), 121.4 (C-8), 108.1 (C-3), 104.7 (C-2), 64.3 (C-1’), 15.1 (C-2’).

Melting point (cyclohexane): decomposition starts at 92.5°C.

Spectral data correspond to literature.[6]

6.1.3 4-Propyloxy-1-naphthol (**36**)

According to GP1, 1,4-naphthoquinone (1.121 g, 7.00 mmol, 1 equiv.) was dissolved in propan-1-ol (14 mL). A solution of $SnCl_2{\cdot}2\ H_2O$ (5.087 g, 24.50 mmol, 3.5 equiv.) in 12 M HCl (4.9 mL) in a 100 mL round bottom flask and heated under reflux for 2 h. The crude product was purified by column chromatography (EA:CH 98% → 95% in 12 column volumes. The product was obtained as a colorless to slight violet amorphous solid (596 mg, 2.947 mmol, 42%).

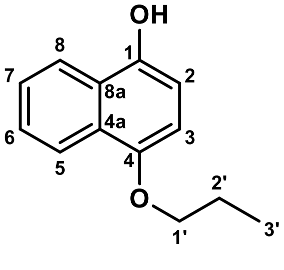

^{1}H NMR (500 MHz, $CDCl_3$) δ (ppm) = 8.29 – 8.25 (m, 1H, 5-*H*), 8.16 – 8.07 (m, 1H, 8-*H*), 7.55 – 7.48 (m, 2H, 6-*H*, 7-*H*), 6.72 (d, ^{3}J = 8.1 Hz, 1H, 2-*H*), 6.64 (d, ^{3}J = 8.1 Hz, 1H, 3-*H*), 4.91 (bs, 1H, 1-O*H*) 4.05 (t, ^{3}J = 6.4 Hz, 2H, 1‘-*H*), 1.93 (qt, ^{3}J = 7.4, 6.4 Hz, 2H, 2‘-*H*) 1.13 (t, ^{3}J = 7.4 Hz, 3H, 3‘-*H*).

^{13}C NMR (126 MHz, $CDCl_3$) δ (ppm) = 149.4 (C-4), 145.0 (C-1), 126.7 (C-4a), 126.0, 125.8 (C-6, C-7), 125.4 (C-8a), 122.3 (C-8), 121.5 (C-5), 108.2 (C-2), 104.6 (C-3), 70.2 (C-1’), 22.9 (C-2’), 11.0 (C-3’).

Spectral data correspond to literature.[7]

6.1.4 4-(1-Methylethyl)oxy-1-naphthol (**38**)

According to GP1, 1,4-naphthoquinone (800 mg, 5.00 mmol, 1 equiv.) was dissolved in isopropanol (10 mL). A solution of $SnCl_2 \cdot 2\,H_2O$ (3.633 g, 17.5 mmol, 3.5 equiv.) in 12 M HCl (3.5 mL) in a 50 mL round bottom flask and heated under reflux for 16 h. The crude product was purified by reverse column chromatography (MeCN:H_2O 30% → 70% in 60 min). The product was obtained as a colorless amorphous solid (382 mg, 1.90 mmol, 38%).

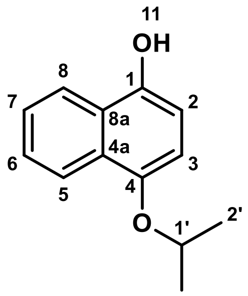

^{1}H NMR (400 MHz, $CDCl_3$) δ (ppm) = 8.25 – 8.21 (m, 1H, 5-*H*), 8.13 – 8.09 (m, 1H, 8-*H*), 7.53 – 7.47 (m, 2H, 6-*H*, 7-*H*), 6.74 – 6.70 (m, 2H, 2-*H*, 3-*H*) 4.96 (bs, 1H, 1-O*H*), 4.61 (hept, ^3J = 6.1 Hz, 1H, 1'-*H*), 1.42 (d, ^3J = 6.1 Hz, 6H, 2'-*H*).

^{13}C NMR (101 MHz, $CDCl_3$) δ (ppm) = 148.0 (C-4), 145.2 (C-1), 127.8 (C-8a), 125.8 (C-7), 125.8 (C-6), 125.5 (C-4a), 122.6 (C-5), 121.4 (C-8), 108.2 (C-3), 107.6 (C-2), 71.4 (C-1'), 22.3 (C-2').

Spectral data correspond to literature.[7]

6.1.5 4-Pentyloxy-1-naphthol (**39**)

According to GP1, 1,4-naphthoquinone (1.121 g, 7.00 mmol, 1 equiv.) was dissolved in pentan-1-ol (14 mL). A solution of $SnCl_2 \cdot 2\,H_2O$ (5.087 g, 24.50 mmol, 3.5 equiv.) in 12 M HCl (4.9 mL) in a 100 mL round bottom flask and heated under reflux for 1 h. The crude product was purified by column chromatography (EA:CH 98% → 95% in 12 column volumes). The product was obtained as a colorless to slight violet amorphous solid (726 mg, 3.59 mmol, 51%).

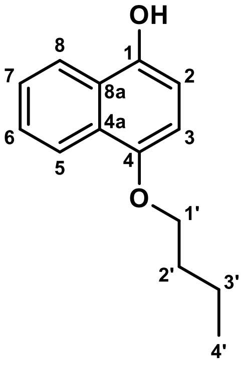

^{1}H NMR (500 MHz, $CDCl_3$) δ (ppm) = 8.30 – 8.23 (m, 1H, 5-*H*), 8.16 – 8.08 (m, 1H, 8-*H*), 7.56 – 7.47 (m, 2H, 6-*H*, 7-*H*), 6.72 (d, ^{3}J = 8.1 Hz, 1H, 2-*H*), 6.64 (d, ^{3}J = 8.1 Hz, 1H, 3-*H*), 4.98 (bs, 1H, 1-O*H*) 4.09 (t, ^{3}J = 6.4 Hz, 2H, 1'-*H*), 1.90 (tt, ^{3}J = 9.0, 6.4 Hz, 2H, 2'-*H*), 1.60 (qt, ^{3}J = 9.0, 7.4 Hz, 2H, 3'-*H*) 1.03 (t, ^{4}J = 7.4 Hz, 3H, 4'-*H*).

^{13}C NMR (126 MHz, $CDCl_3$) δ (ppm) = 149.4 (C-4), 145.0 (C-1), 126.7 (C-4a), 126.0, 125.8 (C-6, C-7), 125.4 (C-8a), 122.3 (C-8), 121.5 (C-5), 108.2 (C-2), 104.5 (C-3), 68.4 (C-1'), 31.6 (C-2'), 19.7 (C-3'), 14.1 (C-4').

Spectral data correspond to literature.[7]

6.1.6 4-Cyclohexyloxy-1-naphthol (**40**)

According to GP1, 1,4-naphthoquinone (1.121 g, 7.00 mmol, 1 equiv.) was dissolved in cyclohexanol (14 mL). A solution of $SnCl_2 \cdot 2\ H_2O$ (5.087 g, 24.5 mmol, 3.5 equiv.) in 12 M HCl (4.9 mL) in a 50 mL round bottom flask and heated under reflux for 26 h. The crude product was purified by column chromatography (CH:EA 99% → 95% in 60 min). The product was obtained as a violet amorphous solid (550 mg, 2.27 mmol, 32%).

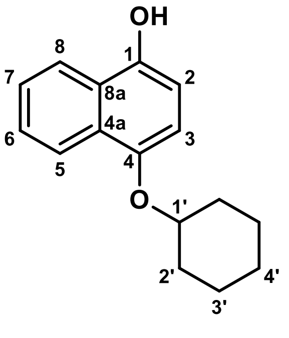

^{1}H NMR (400 MHz, $CDCl_3$) δ (ppm) = 8.28–8.24 (m, 1H, 5-*H*), 8.13–8.09 (m, 1H, 8-*H*), 7.53–7.48 (m, 2H, 6-*H*, 7-*H*), 6.72 (s, 2H, 3-*H*), 5.03 (bs, 1H, 1-O*H*), 4.37–4.31 (m, 1H, 1'-*H*), 2.05–2.00 (m, 2H, 2'-*H*), 1.87–1.83 (m, 2H, 3'-*H*) 1.71–1.64 (m, 2H, 2'-*H*), 1.61–1.55 (m, 1H, 4'-*H*), 1.43–1.37 (m, 3H, 3'-*H*, 4'-*H*).

^{13}C NMR (101 MHz, $CDCl_3$) δ (ppm) = 147.7 (C-4), 145.2 (C-1), 127.9 (C-8a), 125.8 + 125.7 (C-7 + C-6), 125.5 (C-4a), 122.6 (C-5), 121.5 (C-8), 108.2 (C-3), 107.8 (C-2), 76.6 (C-1'), 32.0 (C-2'), 25.9 (C-4'), 23.8 (C-3').

Spectral data correspond to literature.[7]

6.1.7 4-Octyloxy-1-naphthol (**41**)

According to GP1, 1,4-naphthoquinone (1.121 g, 7.00 mmol, 1 equiv.) and octan-1-ol (2.75 mL, 17.50 mmol, 2.5 equiv.) were dissolved in acetonitrile (13 mL). A solution of $SnCl_2 \cdot 2\ H_2O$ (5.087 g, 24.5 mmol, 3.5 equiv.) in 12 M HCl (4.9 mL) was added in a 50 mL round bottom flask and heated under reflux for 4 h. The crude product was purified by column chromatography (CH:EA 99% → 95% in 60 min). The product was obtained as a violet oil (496 mg, 1.82 mmol, 26%).

The procedure was adapted to allow the mixing of the reaction mixture with the aqueous HCl solution.

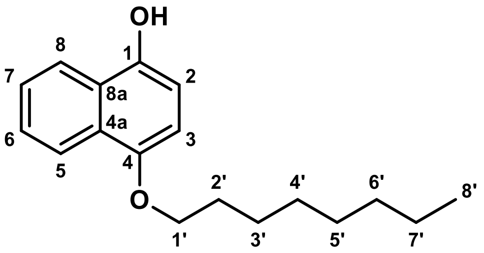

^{1}H NMR (400 MHz, $CDCl_3$) δ (ppm) = 8.31 – 8.21 (m, 1H, 5-*H*), 8.14 – 8.09 (m, 1H, 8-*H*), 7.57 – 7.46 (m, 2H, 6-*H*, 7-*H*), 6.72 (d, ^{3}J = 8.1 Hz, 1H, 3-*H*), 6.63 (d, ^{3}J = 8.1 Hz, 1H, 2-*H*) 4.85 (bs, 1H, 1-O*H*), 4.07 (t, ^{3}J = 6.4 Hz, 2H, 1'-*H*), 1.90 (q, ^{3}J = 6.4 Hz, 2H, 2'-*H*), 1.59 – 1.52 (m, 2H, 3'-*H*), 1.44 – 1.26 (m, 9H, 4'-*H*, 5'-*H*, 6'-*H*, 7'-*H*), 0.92 – 0.89 (m, 3H, 8'-*H*).

^{13}C NMR (101 MHz, $CDCl_3$) δ (ppm) = 149.4 (C-4), 145.0 (C-1), 126.7 (C-8a), 126.6, 125.8 (C-6, C-7), 125.4 (C-4a), 122.3 (C-5), 121.5 (C-8), 108.2 (C-3) 104.6 (C-2), 68.7 (C-1'), 32.0 (C-4', C-5', C-6' or C-7'), 29.6 (C-2' and C-4', C-5', C-6' or C-7'), 29.4 (C-4', C-5', C-6' or C-7'), 26.4 (C-3'), 22.8 (C-4', C-5', C-6' or C-7'), 14.3 (C-8').

HRMS for $C_{18}H_{24}O_2$ (ESI+) [M-H^+]: *m/z* calculated 271.1704, found 271.1710.

6.1.8 Pent-4-yn-1-yl methanesulfonate (**42**)

In a 50 mL dry round bottom flask, pent-4-yn-1-ol (1.262 g, 15.00 mmol, 1 equiv.) was dissolved in dry dichloromethane (15 mL) and triethylamine (4.16 mL, 30.00 mmol, 2 equiv.). The solution was cooled to 0°C in an ice bath. Metanesulfonyl chloride (1.74 mL, 22.50 mmol, 1.5 equiv.) was then added dropwise while stirring. Afterward the reaction mixture was stirred at rt. for 21 h. After the reaction was completed, water (30 mL) was added. The reaction

mixture was extracted with dichloromethane (3x40 mL), and the combined organic layers were extracted with saturated $NaHCO_3$ solution (2x40 mL) and brine (1x40 mL). The organic phase was dried over $MgSO_4$ and the solvent was removed under reduced pressure. The product was obtained as a yellow/brown liquid (2.415 g, 14.89 mmol, 99%).

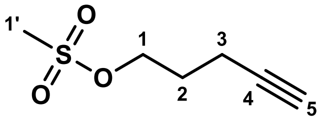

^{1}H NMR (400 MHz, $CDCl_3$) δ (ppm) = 4.36 (t, ^3J = 6.0 Hz, 2H, 1-*H*), 3.03 (s, 3H, 1'-*H*), 2.36 (td, ^3J = 6.8, ^4J = 2.6 Hz, 2H, 3-*H*), 2.01 (t, ^4J = 2.6 Hz, 1H, 5-*H*), 1.96 (tt, ^4J = 6.8, 6.0 Hz, 2H, 2-*H*).

^{13}C NMR (101 MHz, $CDCl_3$) δ (ppm) = 82.2 (C-4), 69.9 (C-5), 68.4 (C-1), 37.4 (C-1'), 27.9 (C-2), 14.8 (C-3).

Known compound, spectral data correspond to literature.[8]

6.1.9 4-(Pent-4-yn-1-yl)oxy-1-naphthol (**43**)

In a 100 mL round bottom flask K_2CO_3 (840 mg, 6.08 mmol, 1.2 equiv.), KI (84 mg, 0.51 mmol, 0.1 equiv.) and 1,4-naphthohydroquinone (812 mg, 5.07 mmol, 1 equiv.) were dispersed in acetone (50 mL). Pent-4-yn-1-yl-methanesulfonate (904 mg, 5.58 mmol, 1.1 equiv.) was added to the slurry and the reaction was heated under reflux using a heating mantle for 16 h. Water (50 mL) was pounded in the reaction mixture. The mixture was extracted with ethyl acetate (3x50 mL). The combined organic layers were washed with brine (1x100 mL) and dried over $MgSO_4$. The solvent was removed under reduced pressure and the crude product was purified by reverse column chromatography (MeCN:H_2O 65% → 95% in 100 min). The product was obtained as a slightly reddish highly viscous oil (230 mg, 1.01 mmol, 20%).

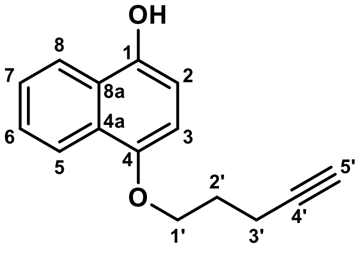

^{1}H NMR (400 MHz, $CDCl_3$) δ (ppm) = 8.26 – 8.19 (m, 1H, 5-*H*), 8.16 – 8.08 (m, 1H, 8-*H*), 7.57 – 7.46 (m, 2H, 6-*H*, 7-*H*), 6.72 (d, ^3J = 8.1 Hz, 1H, 3-*H*), 6.66 (d, ^3J = 8.1 Hz, 1H, 2-*H*) 4.99 (s, 1H, 1-O*H*), 4.19 (t, ^3J = 6.0 Hz, 2H, 1‘-*H*), 2.52 (td, ^3J = 7.0 Hz, ^4J = 2.7 Hz, 2H, 3‘-*H*), 2.13 (tt, ^3J = 7.0, 6.0 Hz, 2H, 2‘-*H*), 1.99 (t, ^4J = 2.7 Hz, 1H, 5‘-*H*).

^{13}C NMR (101 MHz, $CDCl_3$) δ (ppm) = 149.1 (C-4), 145.2 (C-1), 126.6 (C-8a), 126.0, 125.9 (C-6, C-7), 125.4 (C-4a), 122.2 (C-5), 121.6 (C-8), 108.1 (C-3), 104.7 (C-2), 83.8 (C-4'), 69.0 (C-5'), 66.9 (C-1'), 28.6 (C-2'), 15.6 (C-3').

HRMS for $C_{15}H_{14}O_2$ (ESI+) [M+H$^+$]: *m/z* calculated 227.1067, found 227.1066.

6.1.10 4-Benzyloxy-1-naphthol (**44**)

In a 250 mL round bottom flask K_2CO_3 (1.230 g, 8.90 mmol, 0.89 equiv.), KI (84 mg, 0.51 mmol, 0.05 equiv.) and 1,4-naphthohydroquinone (1.602 g, 10.00 mmol, 1 equiv.) were dispersed in acetone (100 mL). Benzyl bromide (1.05 mL, 8.85 mmol, 0.88 equiv.) was added to the slurry and the reaction was heated under reflux using a heating mantle for 16 h. Water (75 mL) was pounded in the reaction mixture. The mixture was extracted with ethyl acetate (3x50 mL). The combined organic fractions were washed with brine (1x100 mL) and dried over $MgSO_4$. The solvent was removed under reduced pressure and the crude product was purified by column chromatography (EA:CH 98% → 95% in 50 min). The product was obtained as a violet amorphous solid (853 mg, 3.41 mmol, 34%).

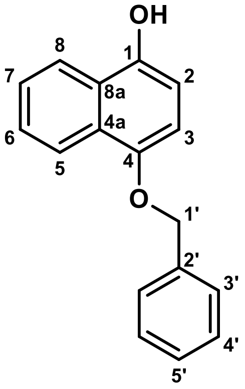

^{1}H NMR (400 MHz, $CDCl_3$) δ (ppm) = 8.34 – 8.29 (m, 1H, 5-*H*), 8.15 – 8.11 (m, 1H, 8-*H*), 7.56 – 7.48 (m, 4H, 3'-*H*, 4'-*H*), 7.46 – 7.39 (m, 2H, 5'-*H*, 7-*H*), 7.38 – 7.32 (m, 1H, 6-*H*), 6.72 (s, 2H, 2-*H,* 3-*H*), 5.20 (s, 2H, 1'-*H*), 4.98 (s, 1H, 1-O*H*).

^{13}C NMR (101 MHz, $CDCl_3$) δ (ppm) = 145.4, 137.6, 128.8, 128.7, 128.1, 128.0, 127.6, 127.51, 126.1, 126.0, 122.4, 121.6, 108.0, 105.3, 70.7.

Spectral data correspond to literature.[7]

6.1.11 5-((Tri-(1-methylethyl)silyl)oxy)naphthalen-1-ol (**15**)

In a 100 mL Schlenk flask, naphthalene-1,5-diol (640 mg, 4.00 mmol, 1.00 equiv.) and 1*H*-imidazole (680 mg, 10.00 mmol, 2.5 equiv.) were dissolved in dry dichloromethane (30.0 mL). After completion the flask was equipped with a septum and tri-(methylethyl)chlorosilane (1.02 mL, 4.80 mmol, 1.20 equiv.) was added dropwise into the solution. The reaction has been conducted in dry glass ware under argon atmosphere. The reaction was stirred for 5 h at rt. After completion, the reaction was quenched by the addition of water (50 mL). The reaction mixture was poured into a separation funnel and the aqueous layer was washed successively with dichloromethane (3x20 mL). The organic layers were

combined and were dried over $MgSO_4$. The solvent was evaporated under reduced pressure. The crude residue was purified via reverse phase column chromatography (MeCN:H_2O 65% → 95% in 60 min). The product was obtained as a light brownish solid (753 mg, 2.38 mmol, 59%).

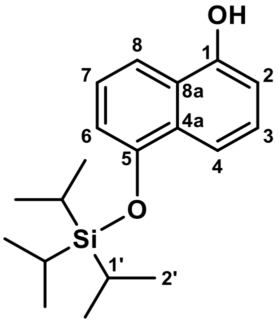

^{1}H NMR (500 MHz, $CDCl_3$) δ (ppm) = 7.87 (dt, ^3J = 8.5, ^4J = 1.0 Hz, 1H, 4-*H*), 7.74 (dt, ^3J = 8.5, ^4J = 1.0 Hz 1H, 8-*H*), 7.35 – 7.27 (m, 2H, 3/7-*H*), 6.90 (dd, ^3J = 7.6, ^4J = 1.0 Hz, 1H, 6-*H*), 6.83 (dd, ^3J = 7.6, ^4J = 1.0 Hz, 1H, 2-*H*), 5.22 (bs, 1H, 1-O*H*), 1.41 (hept, ^3J = 7.1 Hz, 3H, 1'-*H*), 1.41 (d, ^3J = 7.1 Hz, 18H, 2'-*H*).

^{13}C NMR (126 MHz, $CDCl_3$) δ (ppm) = 152.2 (C-5), 151.3 (C-1), 129.3 (C-4a), 126.0 (C-8a), 125.5 (C-7), 125.0 (C-3), 115.7 (C-8), 114.1 (C-4), 112.8 (C-6), 109.2 (C-2), 18.3 (C-2'), 13.2 (C-1').

HRMS for $C_{19}H_{28}O_2Si$ (ESI+) [M+H$^+$]: *m*/*z* calculated 317.1931, found 317.1924.

6.1.12 4-Acetoxy-1-naphthol (**16**)

In a 50 mL round bottom flask acetic anhydride (0.765 mL, 7.50 mmol, 1.5 equiv.) was added to a solution of 1,4-dihydroxynaphthalene (800 mg, 5.00 mmol, 1 equiv.) in pyridine (10 mL). The solution was stirred for 3 h at rt. Afterwards the reaction was quenched by the addition of water (100 mL). The mixture was extracted with ethyl acetate (3x 50 mL). The combined organic layers were washed with water (1x 50 mL), saturated aqueous $CuSO_4$ (4x 50 mL) and brine (1x 100 mL). The combined organic layers were dried over $MgSO_4$ and the solvent was evaporated under reduced pressure. The crude product was purified by column chromatography (EA:CH 95% → 90% in 50 min). The product was obtained as a yellow solid (58 mg, 0.2868 mmol, 6%) as well as the diacetylated product (905 mg, 3.71 mmol, 74%).

The diacetylated product was transferred to the monoacetylated product (887 mg, 3.632 mmol, 1 equiv.) by reduction. It was therefore transferred in a 100 mL round bottom flask with an argon atmosphere. The solid was suspended in ethanol (35 mL) and $NaBH_4$ (75 mg, 2.000 mmol, 0.55 equiv.) was added. The reaction was stirred for 3 h at rt. After completion the reaction was quenched by the addition of water (50 mL). The reaction mixture was acidified using 1 M aqueous HCl (pH =2–3) and extracted with dichloromethane (3x50 mL). The

combined organic layers were washed with $NaHCO_3$ (50 mL), brine (50 mL) and dried over $MgSO_4$. The solvent was removed under reduced pressure and the crude product was purified by column chromatography (EA:CH 95% → 90% in 50 min). The monoacetylated product (520 mg, 2.57 mmol, 71%) was obtained.[9]

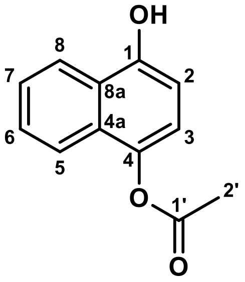

^1H NMR (400 MHz, $CDCl_3$) δ (ppm) = 8.10–8.08 (d, ^3J = 8.3 Hz 1H, 5-*H*), 7.77–7.75 (d, ^3J = 8.3, 1H, 8-*H*), 7.54–7.44 (m, 2H, 6-*H*, 7-*H*), 6.96–6.94 (d, ^3J = 8.1 Hz, 1H, 3-*H*), 6.51–6.49 (d, ^3J = 8.1 Hz 1H, 2-*H*), 6.06 (bs, 1H, 1-O*H*), 2.47 (s, 3H, 2'-*H*).

^{13}C NMR (101 MHz, $CDCl_3$) δ (ppm) = 171.0 (C-1'), 149.9 (C-4), 139.9 (C-1), 127.5 (C-8a), 127.1 (C-7), 125.7 (C-6), 125.3 (C-4a), 122.4 (C-5), 121.0 (C-8), 118.0 (C-3), 107.9 (C-2), 21.2 (C-2').

Spectral data correspond to literature.[9]

6.1.13 4-Bromonaphthalen-1-ol (**17**)

In a 500 mL round bottom flask, 1-naphthol (20.0 g, 138 mmol, 1.00 equiv.) was dissolved in acetonitrile (140 mL). *N*-Bromosuccinimide (24.7 g, 138 mmol, 1.00 equiv.) was added in portions during 1 h. After complete reaction occurred, the reaction was quenched by the addition of water (75 mL) and EA (75 mL). The reaction mixture was poured into a separation funnel and the aqueous layer was washed with ethyl acetate (2x75 mL). The organic fractions were combined and were dried over $MgSO_4$. The solvent was evaporated under reduced pressure. The product was used without further purification. The product was obtained as a colorless solid (22.9 g, 103 mmol, 74%).

Reaction was adapted from literature.[10]

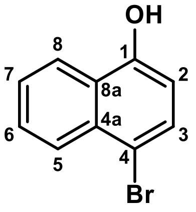

^{1}H NMR (400 MHz, DMSO-d_6) δ (ppm) = 10.52 (s, 1H, 1-OH), 8.24 – 8.17 (m, 1H, 8-H), 8.05 – 7.98 (m, 1H, 5-*H*), 7.69 – 7.60 (m, 1H, 7-*H*) 7.64 (d, ^{3}J = 8.2 1H, 3-*H*), 7.59 – 7.51 (m, 1H, 6-*H*), 6.84 (d, ^{3}J = 8.2 1H, 2-*H*).

^{13}C NMR (101 MHz, $CDCl_3$) δ (ppm) = 153.4 (C-4), 131.9 (C-8a), 130.2 (C-3), 127.9 (C-7), 126.1 (C-5), 126.0 (C-4a), 125.6 (C-6), 122.8 (C-8), 109.9 (C-1), 109.0 (C-2).

Spectral data correspond to literature.[10]

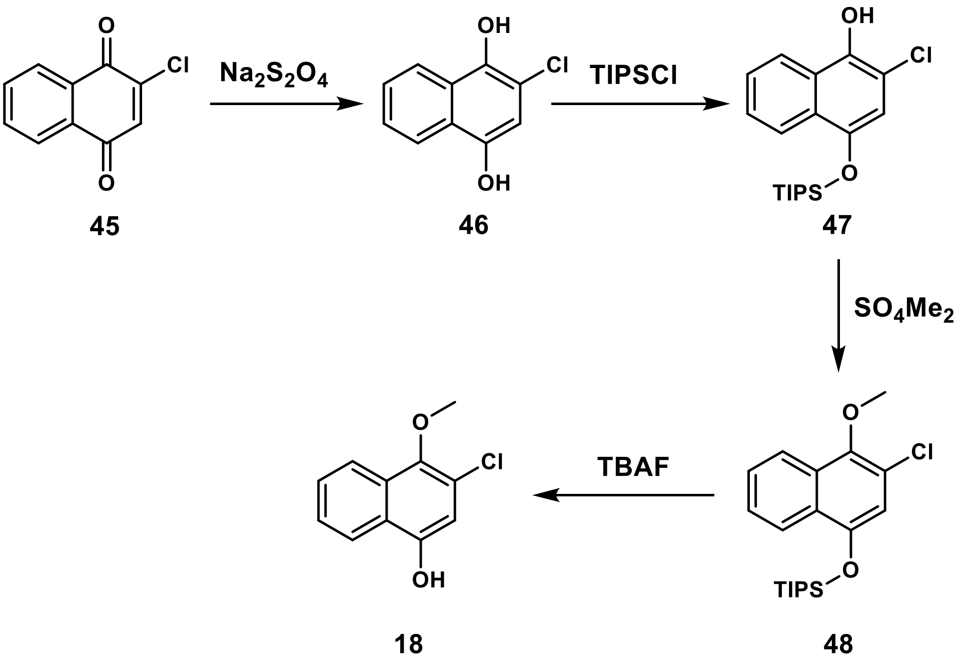

Figure S34: Overview of the synthetic route towards **18**.

6.1.14 2-Chloronaphthalene-1,4-diol (**46**)

In a 1 L separatory funnel 2-chloronaphthalene-1,4-dione (1.500 g, 7.788 mmol, 1 equiv.) was dissolved in ether (375 mL) and sodium dithionite (5.100 g, 29.12 mmol, 3.74 equiv.) was dissolved in water (250 ml). The biphasic mixture was shaken vigorously until the organic phase decolorized. After completion of the reaction the organic phase was collected, dried over $MgSO_4$ and the solvent was removed under reduced pressure. The flask was immediately flushed with argon. The product was obtained as a colorless amorphous solid (1.453 g, 7.466 mmol, 96%). The product was used without further purification.

6.1.15 2-Chloro-4-((tri-(1-methylethyl)silyl)oxy)naphthalen-1-ol (**47**)

In a 100 mL Schlenk flask, 2-chloronaphthalene-1,4-diol (1.453 g, 7.466 mmol, 1.00 equiv.) and 1*H*-imidazole (1.271 g, 18.670 mmol, 2.5 equiv.) were dissolved in dry dichloromethane (60 mL). After completion the flask was equipped with a septum and tri-(1-methylethyl)chlorosilane (1.90 mL, 8.959 mmol, 1.20 equiv.) was added dropwise to the solution. The reaction has been conducted in dry glass ware under argon atmosphere. The

reaction was stirred for 16 h at rt. After complete reaction occurred, the reaction was quenched by with water (50 mL). The reaction mixture was poured into a separation funnel and the aqueous layer was washed successively with dichloromethane (3x50 mL). The organic layers were combined and were dried over $MgSO_4$. The solvent was evaporated under reduced pressure.

The crude residue was purified via column chromatography (EA:CH 98% → 93% in 60 min). The product was obtained as a light brownish solid (1.214 g, 3.459 mmol, 46%).

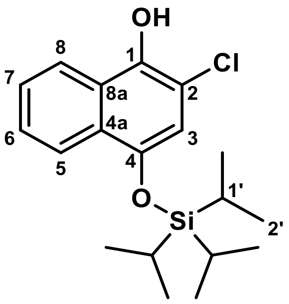

^{1}H NMR (400 MHz, $CDCl_3$) δ (ppm) = 8.22 – 8.11 (m, 2H, 5-*H*, 8-*H*), 7.59 – 7.40 (m, 2H, 6-*H*, 7-*H*), 6.76 (s, 1H, 3-*H*) 5.61 (s, 1H, 1-O*H*), 1.38 (q, ^3J = 7.3 Hz, 3H, 1'-*H*), 1.15 (d, ^3J = 7.3 Hz, 18H, 2'-*H*).

6.1.16 ((3-Chloro-4-methoxynaphthalen-1-yl)oxy)tri-1-methylethylsilane (**48**)

In a 250 mL round bottom flask 2-chloro-4-((tri-(1-methylethyl)silyl)oxy)naphthalen-1-ol (1.214 g, 3.459 mmol, 1 equiv.) was dissolved in acetone (70 mL) under an argon atmosphere. K_2CO_3 (1.446 g, 10.46 mmol, 3.025 equiv.) was suspended in the mixture and dimethylsulfate (0.85 mL, 8.994 mmol, 2.6 equiv.) was added. The mixture was heated under reflux using a heating mantle for 3 h. After full conversion, the reaction was quenched by the addition of water (50 mL) and was stirred for another 1.5 h to make sure that the dimethylsulfate completely reacted. Afterwards 1 M HCl was added to adjust the pH value to ~4. Acetone was removed under reduced pressure, forcing the product to precipitate out of solution. The precipitated solid was filtered through a glass frit (pore size 4). The product was obtained as reddish crystals (985 mg, 2.699 mmol, 78%). The remaining aqueous layers were extracted with ethyl acetate (3x 50 mL). The organic layers were combined, dried over $MgSO_4$ and the solvent was removed under reduced pressure yielding another fraction of product (215 mg, 0.589 mmol, 17%). Combined yield: 95%. The product was used directly in the next step without further purification.

6.1.17 3-Chloro-4-methoxynaphthalen-1-ol (**18**)

In a 100 mL round bottom flask ((3-chloro-4-methoxynaphthalen-1-yl)oxy)-tri-1-methylethylsilane (1.200 g, 3.28 mmol, 1 equiv.) was dissolved in tetrahydrofuran (40 mL). *N,N,N*-tributan-1-aminium fluoride trihydrate (1.578, 5.000 mmol, 1.52 equiv.),

dissolved in THF (10 mL) was added to the solution. The reaction was stirred for 5 h under argon atmosphere. After completion ethyl acetate (150 mL) was added to the solution and the organic layer was washed with brine (3x 75 mL). The organic layer was dried over $MgSO_4$ and the solvent was removed under reduced pressure. The crude product was purified via reverse phase column chromatography (MeCN:H_2O 60% → 95% in 60 min). The product was obtained as a light brownish solid (506 mg, 2.425 mmol, 74%).

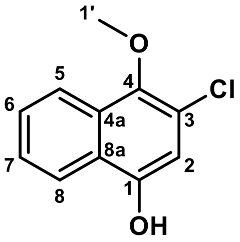

^{1}H NMR (400 MHz, $CDCl_3$) δ (ppm) = 8.13 (ddd, ^3J = 8.4, 1.4, 0.8 Hz, 1H, 8-*H*), 8.07 (ddd, ^3J = 8.4, 1.4, 0.8 Hz, 1H, 5-*H*), 7.57 (ddd, ^3J = 8.4, 6.9, 1.4 Hz, 1H, 6-*H*), 7.50 (ddd, ^3J = 8.4, 1.4, 0.8 Hz, 1H, 7-*H*), 6.81 (s, 1H, 2-*H*), 5.54 (s, 1H, 1-O*H*), 3.96 (s, 3H, 1'-*H*).

^{13}C NMR (101 MHz, $CDCl_3$) δ (ppm) = 148.4 (C-1), 145.8 (C-4) 129.4 (C-4a), 127.6 (C-6), 125.8 (C-7), 124.4 (C-3), 122.4 (C-8a), 122.3 (C-8), 122.0 (C-5), 110.0 (C-2), 61.6 (C-1').

Spectral data correspond to literature.[7]

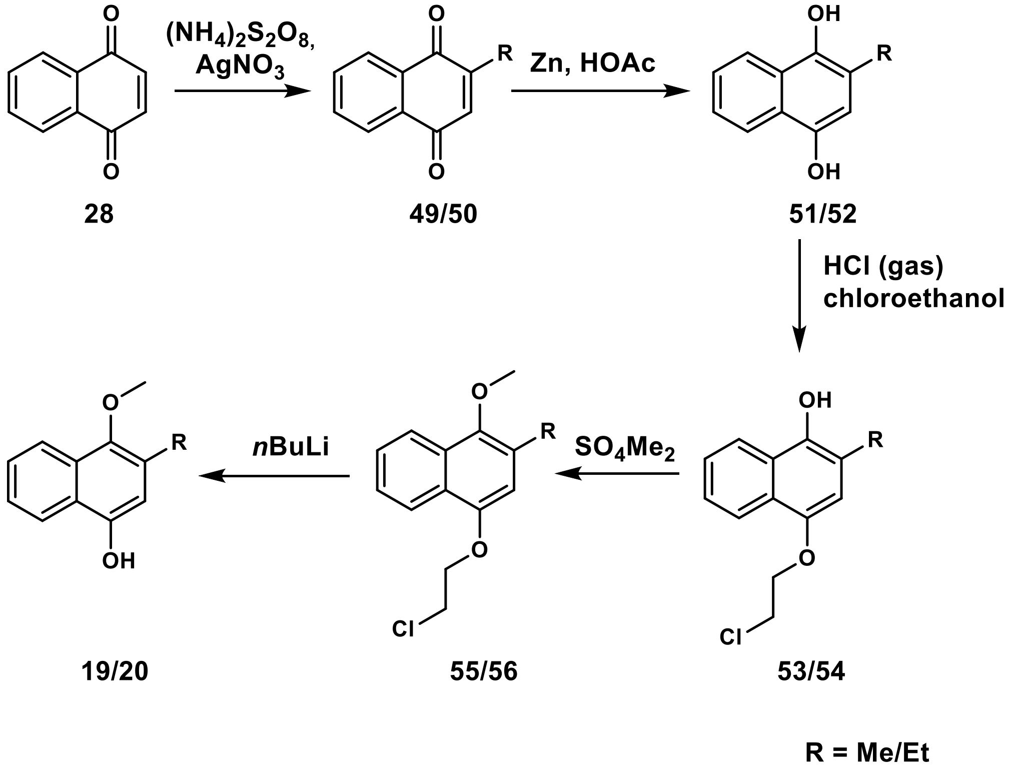

Figure S35: Overview of the synthetic route of **19** and **20**.

6.1.18 2-Ethylnaphthalene-1,4-dione (**50**)

In a 2 L round three-neck-round-bottom-flask, naphthoquinone (50.0 g, 0.32 mol, 1.0 equiv.), propionic acid (23.6 mL, 0.32 mol, 1.0 equiv.) and $AgNO_3$ (9.68 g, 0.06 mol, 0.2 equiv.) were added and suspended in a solvent mixture of acetonitrile, sulfolane and water (1:3:7, 790 mL). To gain complete dissolution of the quinone the mixture was heated to 60 °C using a heating mantle and acetonitrile (250 mL) was added. With vigorous stirring and continuous heating to 60 °C a solution of ammonium peroxodisulfate (86.5 g, 0.38 mol, 1.2 equiv.) in water (160 mL) was added via peristaltic pump at a flow rate of 0.5 mL/min. After the addition of 70 mL, the flow rate was lowered to 0.2 mL/min. The reaction mixture was heated to 60 °C for further 7 h after the addition was completed and then cooled in an ice bath. The reaction mixture was extracted with diethyl ether (4x100 mL) and the combined organic layers were washed with 10% sodium bicarbonate solution (3x100 mL), water (3x100 mL) and brine (2x100 mL), dried over $MgSO_4$ and the solvent was removed under reduced pressure. The aqueous layer was extracted with dichloromethane (3x300 mL), dried over $MgSO_4$, the solvent was removed under reduced pressure and combined to yield the crude product as a brown oil. The crude product was purified by collum chromatography (CH:EA 90:10 isocratic). The product was obtained as yellow needle-shaped crystals (20.3 g, 109 mmol, 35%).[11]

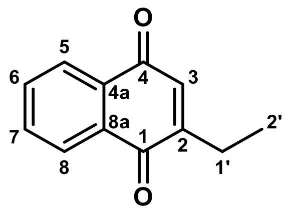

^{1}H NMR (400 MHz, $CDCl_3$) δ (ppm) = 8.11 – 8.01 (m, 2H, 5-*H*, 8-*H*), 7.73 – 7.68 (m, 2H, 6-*H*, 7-*H*), 6.77 (t, ^4J = 1.6, 1H, 3-*H*), 2.60 (qd, ^3J = 7.4, ^4J = 1.6, 2H, 1'-*H*), 1.19 (t, ^3J = 7.4, 3H, 2'-*H*).

^{13}C NMR (101 MHz, $CDCl_3$) δ (ppm) = 185.4 + 185.3 (C-1 + C-4), 153.2 (C-2), 134.1 (C-3), 133.7, 133.7 (C-6, C-7), 132.4 + 132.2 (C-4a + C-8a), 126.7 + 126.1 (C-5 + C-8), 22.7 (C-1'), 12.0 (C-2').

HRMS for $C_{12}H_{10}O_2$ (ESI+) [M+H$^+$]: *m/z* calculated 187.0754, found 187.0753.

Spectral data correspond to literature.[11]

6.1.19 2-Methylnaphthalene-1,4-diol (**51**)

In a 250 mL round-bottom-flask, commercially available 2-methylnaphthalene-1,4-dione (8.61 g, 50 mmol, 1.0 equiv.) was dissolved in glacial acetic acid (100 mL) and powdered zinc (13 g, 200 mmol, 4.0 equiv.) was added. The reaction mixture was heated to 60 °C and stirred under continuous heating for 18 h. After completion of the reduction, the reaction mixture was

filtered and the residue washed with ethyl acetate. The combined filtrates were concentrated in vacuo to obtain the product as grey-brown crystals (8.7 g, 50 mmol, quant.). The product is not stable at air and was used directly for the next step.[12]

6.1.20 2-Ethylnaphthalene-1,4-diol (**52**)

According to procedure 6.1.19 in a 500 mL round-bottom-flask, 2-ethylnaphthalene-1,4-dione (20.3 g, 109 mmol, 1.0 equiv.) was dissolved in glacial acetic acid (304 mL) and powdered zinc (28.5 g, 436 mmol, 4.0 equiv.) was added. The product was obtained as grey-brown crystals (20.5 g, 109 mmol, quant.). The product is not stable at air and was used directly for the next step.[12]

6.1.21 4-(2-Chloroethoxy)-2-methylnaphthalen-1-ol (**53**)

In a 250 mL two-neck-round-bottom-flask, 2-methylnaphthalene-1,4-diol (8.7 g, 37 mmol, 1.0 equiv.) was dissolved in 1,4-dioxane (25 mL). Then, 2-chloroethanol (45.0 g, 558 mmol, 15 equiv.) was added and the solution was purged with a vivid flow of HCl gas for five minutes without cooling. The reaction mixture was poured onto ice water (250 mL). The organic layer was separated, and the aqueous layer was extracted with diethyl ether, dichloromethane and ethyl acetate (150 mL each). The combined organic layers were dried over $MgSO_4$ and the solvent was removed under reduced pressure. The purified product was yielded after recrystallization from cyclohexane (75 mL), at 40 °C as red-brown needle-formed crystals (5.2 g, 22 mmol, 59%).[13]

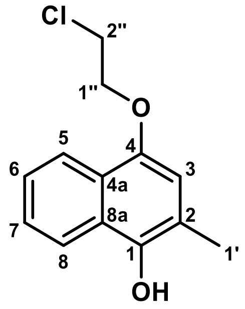

^{1}H NMR (400 MHz, $CDCl_3$) δ (ppm) = 8.25 – 8.20 (m, 1H, 8-*H*), 8.10 – 8.05 (m, 1H, 5-*H*), 7.54 – 7.42 (m, 2H, 6-*H*, 7-*H*), 6.61 (s, 1H, 3-*H*), 4.78 (bs, 1H, 1-O*H*), 4.35 (t, ^{3}J = 5.8, 2H, 1''-*H*), 3.93 (t, ^{3}J = 5.8, 2H, 2''-*H*), 2.39 (s, 3H, 1'-*H*).

^{13}C NMR (101 MHz, $CDCl_3$) δ (ppm) = 148.0 (C-4), 143.0 (C-1), 126.3 (C-6 or 7), 125.5 (C-8a), 125.3 (C-4a), 125.1 (C-6 or 7), 122.1 (C-8), 120.9 (C-5), 116.2 (C-2), 109.3 (C-3), 69.1 (C-1''), 42.3 (C-2''), 16.2 (C-1').

Melting range: 102 – 104 °C (cyclohexane)

Spectral data correspond to literature.[7]

6.1.22 4-(2-Chloroethoxy)-2-ethylnaphthalen-1-ol (**54**)

According to procedure 6.1.21 in a 250 mL two-neck-round-bottom-flask, 2-ethylnaphthalene-1,4-diol (20.5 g, 109 mmol, 1.0 equiv.) was dissolved in 1,4-dioxane (44 mL). Afterwards 2-chloroethanol (87.8 g, 1.09 mol, 10 equiv.) was added and the solution was purged with a vivid flow of HCl gas for five minutes without cooling. The purified product was yielded after recrystallization from cyclohexane (150 mL), at 40 °C as red-brown needle-formed crystals (3.84 g, 15.3 mmol, 14%).[13]

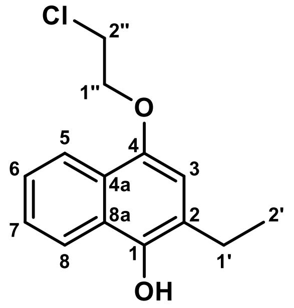

^{1}H NMR (400 MHz, $CDCl_3$) δ (ppm) = 8.25 – 8.20 (m, 1H, 8-*H*), 8.10 – 8.05 (m, 1H, 5-*H*), 7.55 – 7.43 (m, 2H, 6-*H*, 7-*H*), 6.64 (s, 1H, 3-*H*), 4.83 (bs, 1H, 1-O*H*), 4.37 (t, ^{3}J = 5.9 Hz, 2H, 1''-*H*), 3.93 (t, ^{3}J = 5.9 Hz, 2H, 2''-*H*), 2.76 (q, ^{3}J = 7.6 Hz, 2H, 1'-*H*), 4.37 (t, ^{3}J = 7.6 Hz, 3H, 2''-*H*).

^{13}C NMR (101 MHz, $CDCl_3$) δ (ppm) = 148.6 (C-1), 142.6 (C-4), 126.6 (C-6 o. C-7), 126.1 + 125.6 (C-4a + C-8a), 125.5 (C-6 o. C-7), 123.0 (C-2), 122.4 (C-8), 121.3 (C-5), 108.1 (C-3), 69.5 (C-1''), 42.7 (C-2''), 23.9 (C-1'), 15.0 (C-1'').

HRMS for $C_{14}H_{15}O_2Cl$ (ESI–) [M–H^{+}]: *m/z* calculated 249.0688, found 249.0684.

Melting point: 104 °C (cyclohexane).

6.1.23 4-(2-Chloroethoxy)-1-methoxy-2-methylnaphthalene (**55**)

In a 50 mL round-bottom-flask, 4-(2-chloroethoxy)-2-methylnaphthalen-1-ol (943 mg, 4 mmol, 1.0 equiv.) was dissolved in 1,4-dioxane (10 mL). Afterwards 40% aqueous sodium hydroxide solution (10 mL) was added under stirring and the reaction mixture was cooled in an ice bad. Dimethyl sulfate (0,42 mL, 4.4 mmol, 1.1 equiv.) was added via cannula and the reaction mixture was stirred for 1 h at rt., followed by another hour heated under reflux using a heating mantle. The reaction mixture was poured onto 10% aqueous ammonia solution (100 mL). The organic layer was separated, and the aqueous layer was extracted with dichloromethane (3 x 200 mL). The combined organic layers were dried over $MgSO_4$ and the solvent was removed under reduced pressure. The product was obtained as brown crystals (640 mg, 2.6 mmol, 64%).[13] The product was directly used without further purification.

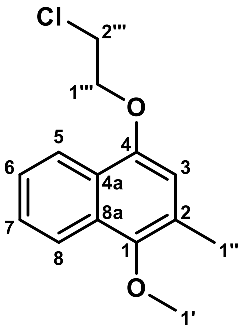

6.1.24 4-(2-Chloroethoxy)-1-methoxy-2-ethylnaphthalene (**56**)

According to procedure 6.1.23 in a 100 mL round-bottom-flask under argon atmosphere, 4-(2-chloroethoxy)-2-ethylnaphthalen-1-ol (3.80 g, 15.2 mmol, 1.0 equiv.) was dissolved in 1,4-dioxane (38 mL). Afterwards 40% aqueous sodium hydroxide solution (38 mL) was added under stirring and the reaction mixture was cooled in an ice bad. Dimethyl sulfate (2.50 mL, 26.4 mmol, 1.7 equiv.) was added via cannula and the reaction mixture. The reaction mixture was poured onto 10% aqueous ammonia solution (500 mL). The product was obtained as brown crystals (3.95 g, 14.9 mmol, 98%).[13]

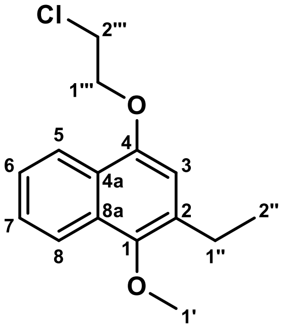

^{1}H NMR (600 MHz, $CDCl_3$) δ (ppm) = 8.30 – 8.26 (m, 1H, 8-*H*), 8.08 – 8.04 (m, 1H, 5-*H*), 7.58 – 7.53 (m, 1H, 6-*H*), 7.49 – 7.45 (m, 1H, 7-*H*), 6.66 (s, 1H, 2-*H*), 4.39 (t, ^3J = 5.8 Hz, 2H, 1'''-*H*), 3.95 (t, ^3J = 5.8 Hz, 2H, 2'''-*H*), 3.91 (s, 3H, 1'-*H*), 2.85 (q, ^3J = 7.6 Hz, 2H, 1''-*H*), 1.33 (t, ^3J = 7.6 Hz, 3H, 2''-*H*).

^{13}C NMR (151 MHz, $CDCl_3$) δ (ppm) = 150.5 (C-1), 147.1 (C-4), 131.9 (C-3), 128.9 (C-4a), 126.7 (C-8), 125.5 (C-8a), 125.0 (C-7), 122.4 (C-6), 121.8 (C-5), 106.9 (C-2), 68.6 (C-1'''), 62.2 (C-1'), 42.3 (C-2'''), 23.1 (C-1''), 15.5 (C-2'').

HRMS for $C_{15}H_{17}O_2Cl$ (ESI+) [M+H$^+$]: *m*/*z* calculated 265.0990, found 265.0987.

6.1.25 4-(2-Chloroethoxy)-1-methoxy-2-methylnaphthalene (**19**)

In a dried Schlenk-flask with argon-atmosphere, 4-(2-chloroethoxy)-2-methyl-1-methoxynaphthalene (481 mg, 2.0 mmol, 1.0 equiv.) was dissolved in dry diethyl ether (25 mL) and was cooled to −78 °C. Afterwards a 2.5 M solution of *n*-BuLi/hexane (3.1 mL, 7.7 mmol, 5.0 equiv.) was added dropwise with continuous stirring at −78 °C and the reaction mixture

was allowed to stir overnight for 19 h while the bath warmed slowly. The reaction mixture was quenched 1 M HCl solution (~ 10 mL) and the organic layer was separated. The aqueous layer was extracted with diethyl ether (3x50 mL) and the combined organic layers were dried over $MgSO_4$ and the solvent was removed under reduced pressure. The crude product was purified by column chromatography (CH:EA 95 → 85% in 100 min). The product was obtained as colorless crystals (368 mg, 1.96 mmol, 98%).[13]

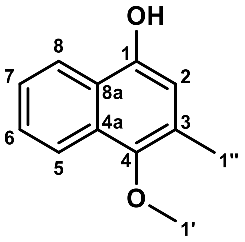

^{1}H NMR (400 MHz, $CDCl_3$) δ (ppm) = 8.13 – 8.10 (m, 1H, 5-*H*), 8.06 – 8.03 (m, 1H, 8-*H*), 7.54 – 7.50 (m, 1H, 7-*H*), 7.46 – 7.42 (m, 1H, 6-*H*), 6.62 (s, 1H, 2-*H*), 6.62 (bs, 1H, 1-O*H*), 3.87 (s, 3H, 1'-*H*), 2.38 (s, 3H, 1''-*H*).

^{13}C NMR (101 MHz, $CDCl_3$) δ (ppm) = 148.0 (C-1), 147.6 (C-4), 129.2 (C-4a), 126.9 (C-7), 126.5 (C-8a), 125.1 (C-6), 124.5 (C-3), 122.4 + 122.13 (C-5 + C-8), 111.9 (C-2), 61.8 (C-1'), 16.3 (C-1'').

HRMS for $C_{12}H_{12}O_2$ (ESI–) [M–H$^+$]: *m/z* calculated 187.0765, found 187.0765.

Spectral data correspond to literature.[7]

6.1.26 4-(2-Chloroethoxy)-1-methoxy-2-ethylnaphthalene (**20**)

According to procedure 6.1.25 in a dried Schlenk-flask with argon-atmosphere, 4-(2-chloroethoxy)-2-ethyl-1-methoxynaphthalene (3.90 g, 14.7 mmol, 1.0 equiv.) was dissolved in diethyl ether (174 mL) and was cooled to −78 °C. A 2.5 M solution of *n*-BuLi/hexane (59.0 mL, 147 mmol, 10.0 equiv.) was added dropwise with continuous stirring at −78 °C. The product was obtained as brown crystals (2.70 g, 13.4 mmol, 91%).[13]

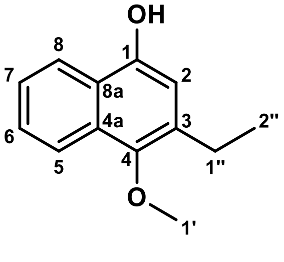

^{1}H NMR (400 MHz, $CDCl_3$) δ (ppm) = 8.10 – 8.09 (m, 1H, 5-*H*), 8.08 – 8.07 (m, 1H, 8-*H*), 7.53 – 7.49 (m, 1H, 7-*H*), 7.45 – 7.42 (m, 1H, 6-*H*), 6.69 (s, 1H, 2-*H*), 5.56 (bs, 1H, 1-O*H*), 3.86 (s, 3H, 1'-*H*), 2.77 (q, ^3J = 7.6 Hz, 2H, 1''-*H*), 1.26 (q, ^3J = 7.6 Hz, 3H, 2''-*H*).

^{13}C NMR (151 MHz, $CDCl_3$) δ (ppm) = 148.1 (C-4), 146.9 (C-1), 132.8 (C-4a), 129.1 (C-8a), 126.7 (C-7), 124.9 (C-6), 124.3 (C-3), 122.2 + 122.17 (C-5 + C-8), 110.1 (C-2), 62.4 (C-1'), 23.0 (C-1''), 15.4 8C-2'').

HRMS for $C_{13}H_{14}O_2$ (ESI+) $[M+H^+]$: *m/z* calculated 201.0921, found 201.0918.

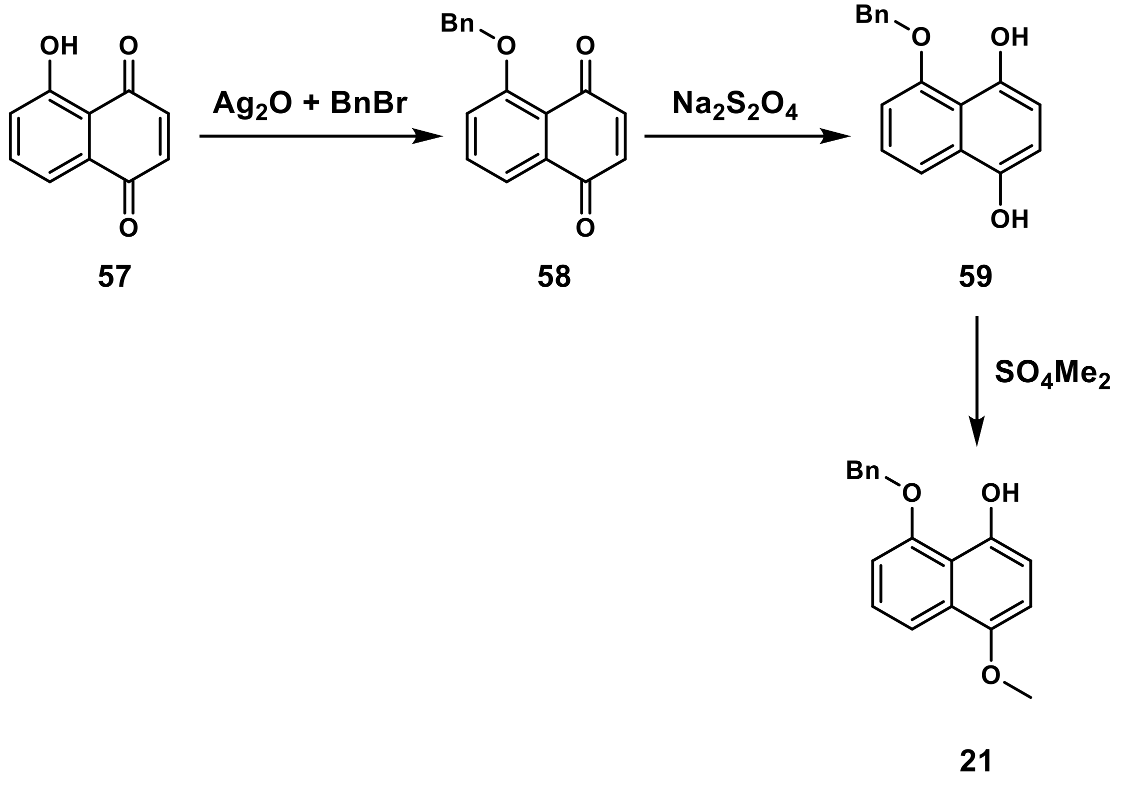

*Figure 36: Overview of the synthetic route of **21**.*

6.1.27 5-(Benzyloxy)naphthalene-1,4-dione (**58**)

In a 100 mL round bottom flask 5-hydroxynaphthalene-1,4-dione (2.00 g, 11.49 mmol, 1 equiv.) was dissolved in DCM (40 mL). Silver(I)oxide (10.59 g, 45.69 mmol, 3.97 eq.) and benzyl bromide (4.09 mL, 34.46 mmol, 3 eq.) was added to the mixture. The reaction was stirred for 7 h. After completion the reaction mixture was filtered through a silica plug to remove silver salts. The plug was washed with DCM (3 x 10 mL). The organic layers were dried over $MgSO_4$ and the solvent was removed under reduced pressure. The crude product was purified via column chromatography (EA:CH 98% → 93% in 90 min). The product was obtained as a yellow solid (2.880 g, 10.90 mmol, 95%) as well as 4,8-Bis(benzyloxy)naphth-1-ol (221 mg, 0.62 mmol, 5%).

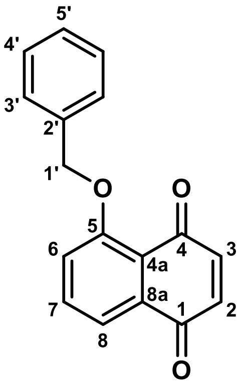

^{1}H-NMR (400 MHz, $CDCl_3$) δ (ppm) = 7.73 (dd, ^{3}J = 7.6, 1.1 Hz, 1H, 8-*H*), 7.63 (dd, ^{3}J = 8.4, 7.6 Hz, 1H, 7-*H*), 7.59 – 7.55 (m, 2H, 6-*H*, 5'-*H*), 7.44 – 7.38 (m, 2H, 3'-*H*), 7.35 – 7.31 (m, 2H, 4'-*H*), 6.87 (s, 2H, 2-*H,* 3-*H*), 5.29 (s, 2H, 1'-*H*).

^{13}C-NMR (101 MHz, $CDCl_3$) δ (ppm) = 185.3 (C-1), 184.2 (C-4), 158.6 (C-5), 141.0 (C-2, C-3), 136.3 (C-6), 136.1 (C-9), 134.9 (C-7), 134.2 (C-8a), 128.8 (C-3'), 128.1 (C-4'), 126.8 (C-5'), 120.4 (C-4a), 119.8 (C-4'), 119.6 (C-8), 71.0 (C-1').

Reaction was adapted from literature.[7]

6.1.28 5-(Benzyloxy)naphthalene-1,4-diol (**59**)

In a 1 L separatory funnel 5-(Benzyloxy)naphthalene-1,4-dione (1.000 g, 3.784 mmol, 1 equiv.) was dissolved in ether (375 mL) and sodium dithionite (3.500 g, 20.00 mmol, 5.28 equiv.) was dissolved in water (250 ml). The two layers were shaken vigorously under argon atmosphere until the organic layer decolorized. After completion of the reaction the organic layer was collected, dried over $MgSO_4$ and the solvent was removed under reduced pressure. After that the flask was immediately flushed with argon. The product was obtained as a colorless amorphous solid (980 mg, 3.680 mmol, 97%). The product was used without further purification.

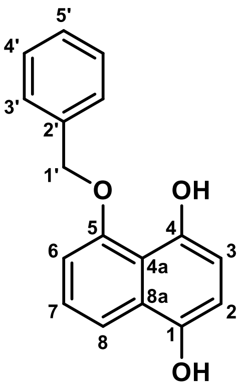

Reaction was adapted from literature.[7]

6.1.29 8-(Benzyloxy)-4-methoxynaphth-1-ol (**21**)

In a 250 mL round bottom flask 5-(Benzyloxy)naphthalene-1,4-diol (980 mg, 3.68 mmol, 1 equiv.) was dissolved in acetone (70 mL) under an argon atmosphere. K_2CO_3 (1.540 g, 11.14 mmol, 3.025 equiv.) was suspended in the mixture and dimethylsulfate (0.86 mL, 9.08 mmol, 2.5 equiv.) was added. The mixture was heated under reflux for 5 h using a heating mantle. After full conversion was detected, the reaction was quenched by the addition of water (50 mL) and was stirred for another 1.5 h to make sure that the dimethylsulfate completely reacted. Afterwards 1 M HCl was added to adjust the pH value to ~4. The acetone was removed under reduced pressure, forcing the product to precipitate out of solution. The precipitated solid was filtered through a glass frit (pore size 4). The product was obtained as reddish crystals (588 mg; 2.07 mmol; 56%). The remaining aqueous washing was extracted with EA (3 x 50 mL). The organic layers were combined, dried over $MgSO_4$ and the solvent was removed under reduced pressure yielding another fraction of product (327 mg, 0.846 mmol, 23%). Combined yield: 79%.

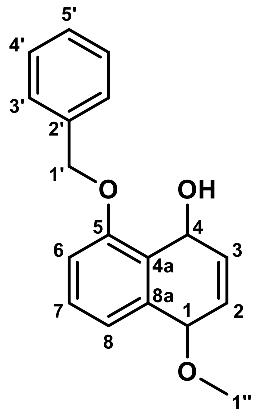

^{1}H-NMR (400 MHz, $CDCl_3$) δ (ppm) = 8.99 (s, 1H, 4-O*H*), 7.87 (dd, ^3J = 8.6, ^4J = 1.4, 1.0 Hz, 1H, 3-*H*), 7.53 – 7.48 (m, 2H, 3'-*H*), 7.46 – 7.38 (m, 3H, 4'-*H*, 5'-*H*), 7.33 (dd, ^3J = 8.6, 7.7 Hz, 1H, 2-*H*), 6.93 (dd, ^3J = 7.7, ^4J = 1.0 Hz, 1H, 2-*H*), 6.76 (s, 2H, 2-*H*, 3-*H*), 5.27 (s, 2H, 1'-*H*), 3.94 (s, 3H, 1''-*H*).

^{13}C-NMR (101 MHz, $CDCl_3$) δ (ppm) = 155.3 (C-1), 148.3 (C-4), 148.0 (C-8a), 135.5 (C-2'), 129.2 (C-4'), 129.0 (C-8), 128.1 (C-3'), 125.3 (C-6), 116.3 (C-5), 115.9 (C-4a), 109.3 (C-2), 106.4 (C-3, C-7), 71.8 (C-1'), 56.1 (C-1'').

Reaction was adapted from literature.[7]

6.1.30 4,4-Dimethoxynaphthalen-1(4*H*)-one (**33**)

In a 50 mL round bottom flask 1-naphthol (835 mg, 5.8 mmol; 1 equiv.) was solved in methanol (25 mL). Afterwards the flask was flushed with argon and cooled to 0°C. To the vigorously stirred solution iodobenzene diacetate (3.731 g; 11.58 mmol; 2 equiv.) was added in small

portions. The reaction was stirred for 3 h and then quenched by purring it in 30 mL saturated $NaHCO_3$ solution. The reaction was extracted with ethyl acetate (3 x 50 mL). The organic layers were dried over $MgSO_4$ and the solvent was removed under reduced pressure. The product was purified by reverse phase column chromatography (MeCN:H_2O 30% → 90% in 60 min). The product was obtained as a brownish oil (256 mg, 1.254 mmol, 22%).

Notably the product is highly prone to hydrolysis in acidic media. The purification by using unmodified silica led to full hydrolysis of the product during the purification.

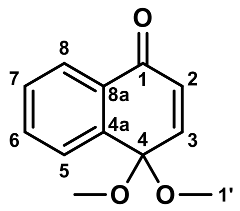

^{1}H-NMR (400 MHz, $CDCl_3$) δ (ppm) = 8.09 (dd, ^{3}J = 7.9, ^{4}J = 1.3 Hz, 1H, 5-*H*), 7.74 (dd, ^{3}J = 7.9, ^{4}J = 1.3 Hz, 1H, 8-*H*), 7.67 (ddd, ^{3}J = 7.9, 7.3 ^{4}J = 1.3 Hz, 1H, 7-*H*), 7.50 (ddd ^{3}J = 7.9, 7.3 ^{4}J = 1.3 Hz, 1H, 6-*H*), 6.93 (d ^{3}J = 10.5 Hz, 1H, 3-*H*), 6.61 (d ^{3}J = 10.5 Hz, 1H, 2-*H*), 3.20 (s, 6H, 1'-*H*).

^{13}C-NMR (101 MHz, $CDCl_3$) δ (ppm) = 184.0, 144.3, 139.7, 133.6, 132.8, 131.7, 129.4, 126.7, 126.4, 95.2, 51.3.

Reaction was adapted from literature.[14]

6.2 Synthesis of polycycles

6.2.1 7*H*,13*H*-5,13-Dimethoxy-7,13-methanobenzo[*f*]naphtho[1,2-*b*]oxocin-8-one (**2**)

According to GP2, 4-methoxy-1-naphthol (131.2 mg, 0.075 mmol, 1 equiv.) and tetraethylammonium hexafluorophosphate (16.6 mg, 0.06 mmol, 0.08 equiv.) were dissolved in HFIP (5 mL). The crude product was purified by column chromatography (silica: 96%→73% CH in 50 min). The product was obtained as a colorless amorphous solid (90 mg, 0.26 mmol, 69%).

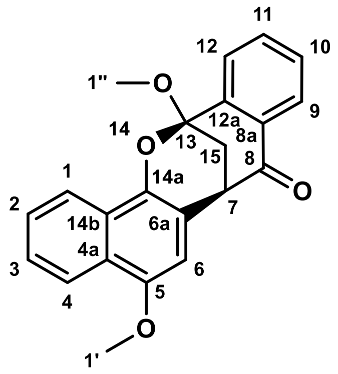

^{1}H NMR (400 MHz, $CDCl_3$) δ [ppm]= 8.17 – 8.10 (m, 2H, 1-*H*, 4-*H*), 8.05 – 8.02 (m, 1H, 12-*H*), 7.97 – 7.94 (m, 1H, 9-*H*), 7.65 – 7.61 (m, 1H, 11-*H*), 7.49 – 7.42 (m, 2H, 2-H, 3-*H*), 7.40 – 7.36 (m, 1H, 10-*H*), 6.64 (s, 1H, 6-*H*), 4.11 (dd, ^{3}J = 3,7 Hz, 2,5 Hz, 1H, 7-*H*), 3.94 (s, 3H, 1'-*H*), 3.75 (s, 1H, 1"-*H*), 2.74 (dd, ^{3}J = 2.5 Hz, ^{2}J = 12.6 Hz, 1H, 15-*H'*), 2.53 (dd, ^{3}J = 3.7 Hz, ^{2}J = 12.6 Hz, 1H, 15-*H"*).

^{13}C NMR (101 MHz, $CDCl_3$) δ [ppm]= 195.2 (C-8), 150.2 (C-5), 143.0 (C-13), 141.8 (C-8a), 134.7 (C-11), 129.9 (C-12a), 129.3 (C-10), 126.7 (C-4a'), 126.4 (C-9), 126.1 (C-12), 125.78 (C-2, C-14b), 125.5 (C-3), 122.0 (C-7), 121.3 (C-1), 110.0 (C-6a), 103.2 (C-6), 97.5 (C-14a), 55.9 (C-1'), 50.3 (C-1"), 48.1 (C-4), 29.2 (C-15).

HRMS for $C_{22}H_{18}O_4$ (ESI+) [M+H^{+}]: *m/z* calculated 347.1278, found 347.1277.

The isolated yield was 68% with 5 mL HFIP substituted by 5 mL DCM:HFIP (1:5).

Scale-Up Experiment:

According to GP2, 4-methoxy-1-naphthol (1.300 g, 7.50 mmol, 1 equiv.) and tetrabutylammonium hexafluorophosphate (165 mg, 0.60 mmol) were dissolved in HFIP (50 mL). The electrolysis was done at 10 °C with 1 *F* and 1.2 A in a 4x12 cm flow cell. The flow rate was 18 mL/min (2.79 mm Ismatec™ tube). The crude product was purified by column chromatography (94% → 73% CH in 45 mins). The product was obtained as a colorless amorphous solid (657 mg, 1.90 mmol, 52%).

For the scale-up reaction the commercially available 4-methoxynaphth-1-ol (97% purity) was used.

Acid stability of the polyclic motif:

To investigate the acid stability of the acetal contained in the product, a drop of trifluoroacetic acid (TFA) was added to a sample polycycle (**2**) in deuterated benzene (0.7 mL) and the course of the reaction was monitored by ^{1}H NMR. A degradation of the *dd*, characteristic for the polycycle, could be observed. In addition, a merging of the aromatic signals into broad multiplets is detectable. This could indicate oligomerization as there is no structural similarity to the homo-coupling product visible. Therefore, the homo-coupling product cannot be a hydrolysis product. However, the acid-catalyzed hydrolysis of the polycycle appears to proceed relatively slowly, since at this high acid concentration, a significant amount of polycycle is still present in solution even after 12 h.

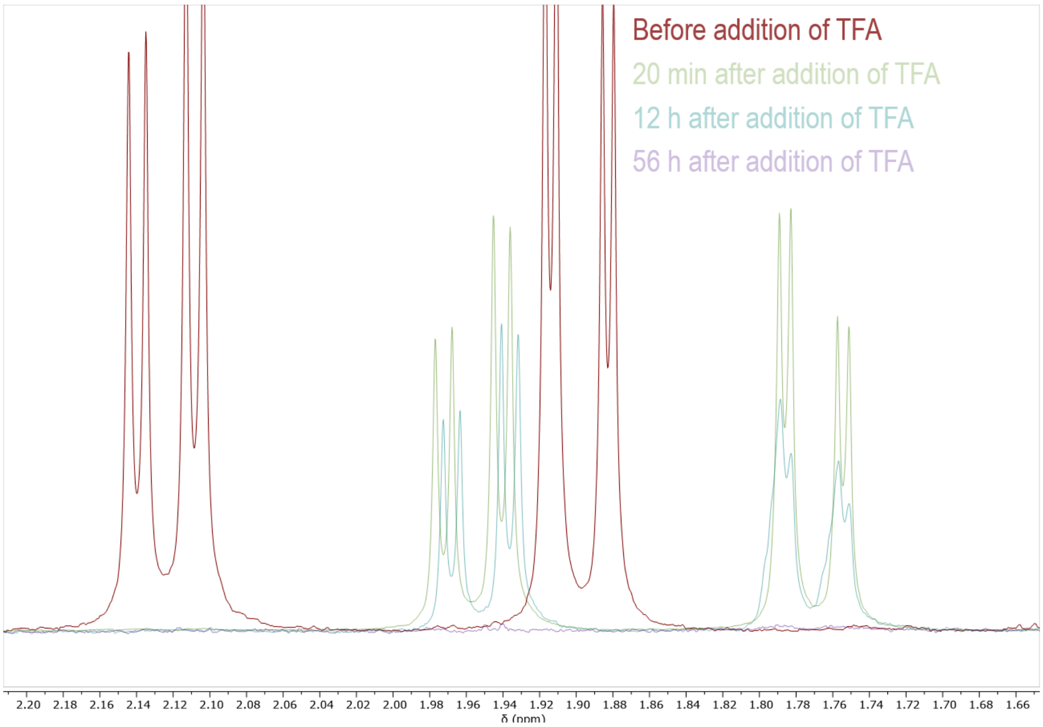

Figure 37: Experiments on the acid stability of **2** shows the change in *dd* over time after the addition of TFA. All spectra were previously normalized to the highest signal. The shift in the signal after the addition of TFA is due to the change in polarity of the solution.

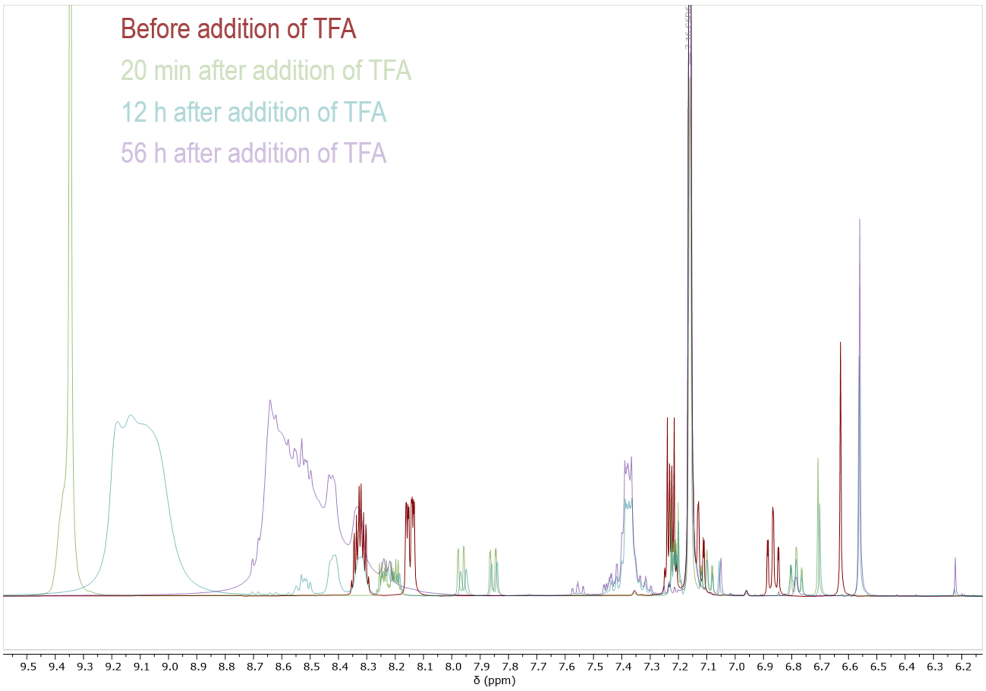

Figure 38: Experiments on the acid stability of **2** shows the change in the aromatic area over time after the addition of TFA. All spectra were previously normalized to the highest signal. The shift in the signal after the addition of TFA is due to the change in polarity of the solution.

6.2.2. 7*H*,13*H*-5,13-Dimethoxy-7,13-ethanobenzo[*f*]naphtho[1,2-*b*]oxocin-8-one (**6**)

According to GP2, 4-ethoxynaphthalen-1-ol (141.2 mg, 0.75 mmol, 1 equiv.) and tetraethylammonium hexafluorophosphate (16.5 mg, 0.06 mmol, 0.08 equiv.) were dissolved in HFIP (5 mL). The crude product was purified by column chromatography (98% → 81% CH in 40 mins) and a reverse phase column chromatography (85% → 100% MeCN in 60 min). The product was obtained as a colorless amorphous solid (99 mg, 0.26 mmol, 71%).

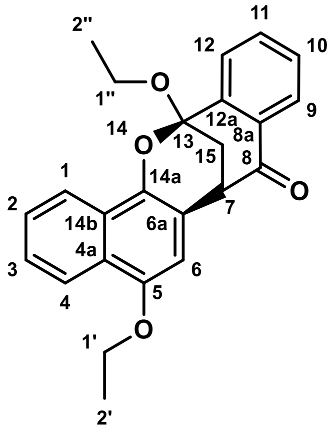

^{1}H NMR (400 MHz, $CDCl_3$) δ [ppm]= 8.20 – 8.11 (m, 2H, 1-*H*, 4-*H*), 8.10 – 8.07 (m, 1H, 12-*H*), 7.98 – 7.92 (m, 1H, 9-*H*), 7.66 – 7.60 (m, 1H, 11-*H*), 7.48 – 7.40 (m, 2H, 2-H, 3-*H*), 7.39 – 7.34 (m, 1H, 10-*H*), 6.64 (s, 1H, 6-*H*), 4.22 – 4.09 (m, 3H, 1'-*H*, 1''(1x)-*H* (geminal coupling constant cannot be detected because of overlap), 4.08 (dd, ^3J = 3.7 Hz, 2.5 Hz, 1H, 7-*H*), 3.88 (dq,

^2J = 9.0 Hz, ^3J = 6.9 Hz 1H, 1'-*H*), 2.72 (dd, ^2J = 12.6 Hz, ^3J = 3.7 Hz, 1H, 15-*H'*), 2.56 (dd, ^2J = 12.6 Hz, ^3J = 2.5 Hz, 1H, 15-*H''*) 1.49 (t, ^3J = 6.9 Hz, 6H, 2'-*H*, 2''-*H*).

^{13}C NMR (101 MHz, $CDCl_3$) δ [ppm]= 195.4 (C-8), 149.4 (C-5), 142.8 (C-4a), 142.0 (C-13), 134.6 (C-11), 129.9 (C-8a), 129.2 (C-10), 126.6 (C-9), 126.5 (C-12a), 126.2 + 126.0 (C-2, C-3), 125.7 (C-14b), 125.6 (C-12), 122.2 + 121.3 (C-1, C-4), 110.2 (C-14a), 104.2 (C-6), 97.4 (C-6a), 64.2 (C-1'), 58.4 (C-1''), 48.3 (C-7), 30.0 (C-15), 16.0 (C-2''), 15.0 (C-2').

HRMS for $C_{24}H_{22}O_4$ (ESI+) [M+H$^+$]: *m/z* calculated 374.1591, found 375.1576.

6.2.3. 7*H*,13*H*-5,13-Dimethoxy-7,13-ethanobenzo[*f*]naphtho[1,2-*b*]oxocin-8-one (**7**)

According to GP2, 4-propoxynaphthalen-1-ol (151.1 mg, 0.75 mmol, 1 equiv.) and tetraethylammonium hexafluorophosphate (16.5 mg, 0.06 mmol, 0.08 equiv.) were dissolved in HFIP (5 mL). The crude product was purified by column chromatography (98% → 67.7% CH in 41 mins). The product was obtained as a yellow amorphous solid (108.9 mg, 0.27 mmol, 72%).

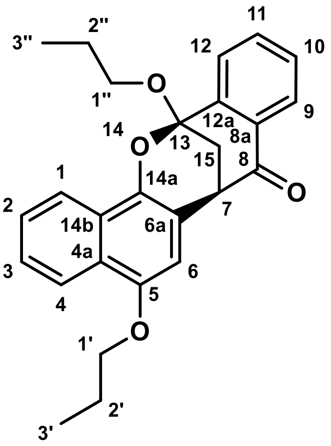

^{1}H NMR (400 MHz, $CDCl_3$) δ [ppm]= 8.22 – 8.17 + 8.17 – 8.12 (m, 2H, 1-*H*, 4-*H*), 8.10 (dd, ^4J = 7.8 Hz, 1.2 Hz, 1H, 12-*H*), 7.97 (dd, ^3J = 7.8 Hz, ^4J = 1.4 Hz, 1H, 9-*H*), 7.63 (ddd, ^3J = 7.9, 7.4 Hz, ^4J = 1.4 Hz, 1H, 11-*H*), 7.50 – 7.42 (m, 2H, 2-H, 3-*H*), 7.37 (ddd, ^3J = 7.9, 7.4 Hz, ^4J = 1.2 Hz, 1H, 10-*H*), 6.66 (s, 1H, 6-*H*), 4.12 – 3.99 (m, 4H, 7-*H,* 1'-*H*, 1''(1x)-*H* (geminal coupling constant cannot be detected because of overlap), 3.78 (dt, ^4J = 6.2 Hz, ^2J = 8.9 Hz 1H, 1'-*H*), 2.71 (dd, ^3J = 2.5 Hz, ^2J = 12.6 Hz, 1H, 15-*H'*), 2.55 (dd, ^3J = 2.5 Hz, ^2J = 12.6 Hz, 1H, 15-*H''*), 1.91 (qt, ^3J = 7.4, 6.2 Hz, 4H, 2'-H, 2''-*H*), 1.19 + 1.12 (t, ^3J = 7.4 Hz, 6H, 3'-*H*, 3''-*H*).

^{13}C NMR (101 MHz, $CDCl_3$) δ [ppm]= 195.4 (C-8), 149.5 (C-5), 142.8 (C-4a), 142.2 (C-13), 134.6 (C-11), 129.9 (C-8a), 129.2 (C-10), 126.6 (C-9), 126.6 (C-12a), 126.3 + 126.0 (C-2, C-3), 125.8 (C-14b), 125.6 (C-12), 122.2 + 121.3 (C-1, C-4), 110.1 (C-14a), 104.1 (C-6), 97.2 (C-

6a), 70.1 + 64.2 (C-1') + (C-1''), 48.3 (C-7), 30.0 (C-15), 23.6 + 22.8 (C-2', C-2''), 11.1 + 10.9 (C-3', C-3'').

HRMS for $C_{26}H_{26}O_4$ (ESI+) [M+H$^+$]: *m/z* calculated 403.1904, found 403.1898.

6.2.4. 7*H*,13*H*-5,13-Di(1-methyleth)oxy-7,13-methanobenzo[*f*]naphtho[1,2-*b*]oxocin-8-one (**8**)

According to GP2, 4-isopropoxynaphthalen-1-ol (152 mg, 0.75 mmol, 1 equiv.) and tetraethylammonium hexafluorophosphate (16.5 mg, 0.06 mmol, 0.08 equiv.) were dissolved in HFIP (5 mL). The crude product was purified by column chromatography (98% → 75% CH in 40 min). The product was obtained as a dark red amorphous solid (95 mg, 0.24 mmol, 63%).

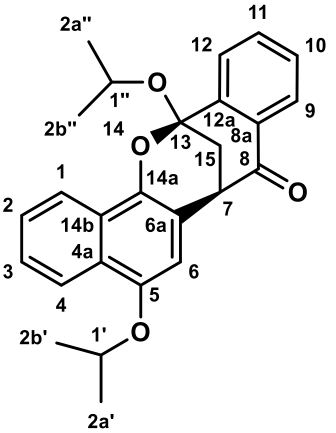

^{1}H NMR (400 MHz, $CDCl_3$) δ [ppm]= 8.16 – 8.12 (m, 1H, 4-*H*), 8.11 – 8.07 (m, 1H, 1-*H*), 8.09 – 8.06 (m, 1H, 12-*H*), 7.95 – 7.93 (m, 1H, 9-*H*), 7.64 – 7.60 (m, 1H, 11-*H*), 7.46 – 7.42 (m, 2H, 3-H, 2-*H*), 7.38 – 7.34 (m, 1H, 10-*H*), 6.69 (s, 1H, 6-*H*), 4.65 (hept, ^3J = 6.2 Hz, 1H, 1'-*H*), 4.52 (hept, ^3J = 6.1 Hz, 1H, 1''-*H*), 4.07 (dd, ^3J = 3.7 Hz, 2.4 Hz, 1H, 7-*H*), 2.71 (dd, ^2J = 12.6 Hz, ^3J = 2.4 Hz,, 1H, 15-*H'*), 2.58 (dd, ^2J = 12.6 Hz, ^3J = 3.7 Hz, 1H, 15-*H''*), 1.62 (d, ^3J = 6.2 Hz, 3H, 2''a-*H*), 1.42 (d, ^3J = 6.1 Hz, 3H, 2'a-*H*), 1.37 (d, ^3J = 6.1 Hz, 3H, 2'b-*H*), 1.32 (d, ^3J = 6.2 Hz, 3H, 2''b-*H*).

^{13}C NMR (101 MHz, $CDCl_3$) δ [ppm]= 148.2 (C-4), 146.8 (C-1), 132.7 (C-3), 129.3 (C-4a), 127.4 (C-6), 125.4 (C-7), 123.8 (C-8a), 123.3 (C-8), 122.0 (C-5), 113.1 (C-2), 62.4 (C-1'), 21.8 (C-1''), 15.0 (C-2'').

HRMS for $C_{26}H_{26}O_4$ (ESI+) [M+H$^+$]: *m/z* calculated 402.1904, found 403.1899.

6.2.5. 7*H*,13*H*-5,13-Di(1-cyclohexyl-7,13-methanobenzo[*f*]naphtho[1,2-*b*]oxocin-8-one (**9**)

According to GP2, 4-hexl-1-naphthol (152.7 mg, 0.75 mmol, 1 equiv.) and tetraethylammonium hexafluorophosphate (16.6 mg, 0.06 mmol, 0.08 equiv.) were dissolved in HFIP (4 mL) and chlorobenzene (1 mL). The electrolysis was performed at 30 °C. The crude

product was purified by column chromatography (5% → 45% dichloromethane in 4 h, TLC in DCM:MeOH beforehand). The product was obtained as a blue amorphous solid (39.7 mg, 0.08 mmol, 22%).

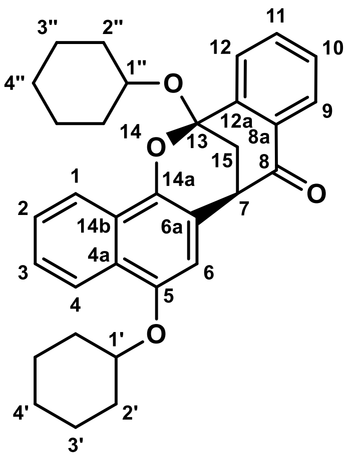

^{1}H NMR (400 MHz, $CDCl_3$) δ [ppm]= 8.20 – 8.15 (m, 1H, 4-*H*), 8.12 – 8.06 (m, 2H, 1-*H,* 12-*H*), 7.96 – 7.92 (m, 1H, 9-*H*), 7.65 – 7.60 (m, 1H, 11-*H*), 7.48 – 7.40 (m, 2H, 2-*H,* 3-*H*), 7.39 – 7.34 (m, 1H, 10-*H*), 6.70 (s, 1H, 6-*H*), 4.40 (tt, ^3J = 8.0, 3.6 Hz, 1H, 1'-*H* or 1''-*H*), 4.18 (tt, ^3J = 9.2, 3.8 Hz, 1H, 1'-*H* or 1''-*H*), 4.07 (dd, ^3J = 3.7, 2.5 Hz, 1H, 7'-*H*), 2.71 (dd, ^2J = 12.7, ^3J = 3.7 Hz, 1H, 15-*H*), 2.57 (dd, ^2J = 12.7, ^3J = 2.5 Hz, 1H, 15'-*H*), 2.30 (m, 1H, cyclohexyl), 2.07 – 1.31 (m, 17H, cyclohexyl), 0.88 – 0.84 (m, 2H, cyclohexyl).

^{13}C NMR (101 MHz, $CDCl_3$) δ [ppm]= 195.5 (C-8), 147.9 (C-5), 143.0 (C-4a), 142.7 (C-13), 134.6 (C-11), 129.8 (C-8a), 129.1 (C-10), 127.6 (C-12a), 126.6 (C-9), 126.2 (C-12), 126.0 (C-14b), 125.9 + 125.9 (C-2, C-3), 122.5 + 121.3 (C-1 + C-4), 110.3 (C-14a), 106.6 (C-6), 97.7 (C-6a), 75.9 + 71.5 (C-1' + C-1''), 48.5 (C-7), 35.1 + 34.6 + 31.8 + 31.6 (C-cyclohexyl), 30.9 (C-8), 29.8, 25.9, 25.7, 24.6, 24.4, 23.7 (C-cyclohexyl).

HRMS for $C_{32}H_{34}O_4$ (ESI+) [M+K$^+$]: *m/z* calculated 521.1723, found 521.1722.

6.2.6. 7*H*,13*H*-5,13-Dibutanoxy-7,13-methanobenzo[*f*]naphtho[1,2-*b*]oxocin-8-one (**10**)

According to GP2, 4-butoxynaphthalen-1-ol (162.6 mg, 0.75 mmol, 1 equiv.) and tetraethylammonium hexafluorophosphate (16.5 mg, 0.06 mmol) were dissolved in HFIP (5 mL). The crude product was purified by column chromatography (98% → 78.5% CH in 43 min). The product was obtained as a colorless amorphous solid (101.7 mg, 0.24 mmol, 63%).

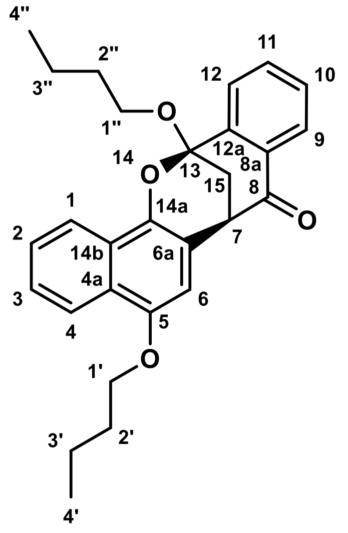

^{1}H NMR (400 MHz, $CDCl_3$) δ [ppm]= 8.20 – 8.12 + 8.12 – 8.09 (m, 2H, 1-*H*, 4-*H*), 8.06 (dd, ^4J = 8.0 Hz, 1.2 Hz, 1H, 12-*H*), 7.94 (dd, ^3J = 8.0 Hz, ^4J = 1.4 Hz, 1H, 9-*H*), 7.62 (ddd, ^3J = 7.9, 7.3 Hz, ^4J = 1.4 Hz, 1H, 11-*H*), 7.48 – 7.41 (m, 2H, 2-H, 3-*H*), 7.37 (ddd, ^3J = 7.6 7.6 Hz, ^4J = 1.4 Hz, 1H, 10-*H*), 6.63 (s, 1H, 6-*H*), 4.16 – 4.02 (m, 4H, 7-*H,* 1'-*H*, 1''(1x)-*H* (geminal coupling constant cannot be detected because of overlap), 3.82 (dt, ^4J = 8.9 Hz, ^2J = 6.0 Hz 1H, 1'-*H*), 2.72 (dd, ^3J = 3.8 Hz, ^2J = 12.6 Hz, 1H, 15-*H'*), 2.55 (dd, ^3J = 2.5 Hz, ^2J = 12.6 Hz, 1H, 15-*H''*), 1.95 – 1.74 (m, 4H, 2'-H, 2''-*H*), 1.70 + 1.51 (m, 3'-*H*, 3''-*H*), 1.08 (t, ^3J = 7.3 Hz 3H, 4'-*H*), 1.01 (t, ^3J = 7.3 Hz 3H, 4'-*H*).

^{13}C NMR (101 MHz, $CDCl_3$) δ [ppm]= 195.4 (C-8), 149.6 (C-5), 142.9 (C-4a), 142.2 (C-13), 134.6 (C-11), 129.2 (C-8a, C-10), 126.7 (C-9), 126.6 (C-12a), 126.4 + 126.3 (C-2, C-3), 125.8 (C-14b), 125.6 (C-12), 122.2 + 121.3 (C-1, C-4), 110.2 (C-14a), 104.1 (C-6), 97.3 (C-6a), 68.3 + 62.3 (C-1') + (C-1'') , 48.3 (C-7), 32.3 (C-15), 31.5 + 30.1 (C-2', C-2''), 19.6 + 19.6 (C-3', C-3'') 14.1 (C-4', C-4'').

HRMS for $C_{28}H_{30}O_4$ (ESI+) [M+H$^+$]: *m/z* calculated 431.2217, found 431.2209.

6.2.7. 7*H*,13*H*-5,13-Dioctanoxy-7,13-methanobenzo[*f*]naphtho[1,2-*b*]oxocin-8-one (**11**)

According to GP2, 4-(octyloxy)naphthalen-1-ol (204.2 mg, 0.75 mmol, 1 equiv.) and tetraethylammonium hexafluorophosphate (16.3 mg, 0.06 mmol) were dissolved in HFIP (4 mL) and dichloromethane (1 mL). The electrolysis was performed at 30 °C. The crude product was purified by column chromatography (98% → 70% CH in 45 mins). The product was obtained as a colorless amorphous solid (108.4 mg, 0.2 mmol, 53%).

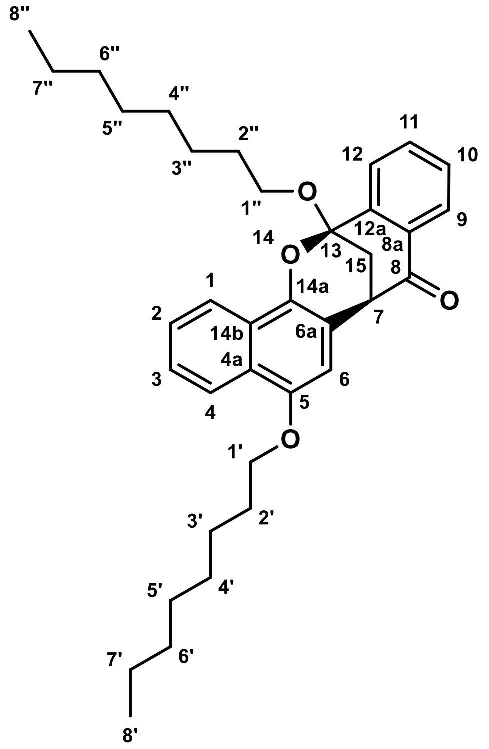

^{1}H NMR (400 MHz, $CDCl_3$) δ [ppm]= 8.20 – 8.10 (m, 2H, 1-*H*, 4-*H*), 8.08 (dd, ^{3}J = 8.0 Hz, 1.2 Hz, 1H, 12-*H*), 7.95 (dd, ^{3}J = 7.8 Hz, ^{4}J = 1.4 Hz, 1H, 9-*H*), 7.63 (ddd, ^{3}J = 8.0, 7.6 Hz, ^{4}J = 1.4 Hz, 1H, 11-*H*), 7.49 – 7.41 (m, 2H, 2-H, 3-*H*), 7.37 (ddd, ^{3}J = 7.8, 7.6 Hz, ^{4}J = 1.2 Hz, 1H, 10-*H*), 6.64 (s, 1H, 6-*H*), 4.16 – 4.02 (m, 4H, 7-*H,* 1'-*H*, 1''(1x)-*H*), 3.84 – 3.77 (m, 1H, 1'-*H*), 2.72 (dd, ^{3}J = 3.8 Hz, ^{2}J = 12.6 Hz, 1H, 15-*H'*), 2.55 (dd, ^{3}J = 2.5 Hz, ^{2}J = 12.6 Hz, 1H, 15-*H''*), 1.96 – 1.76 (m, 4H, 2'-H, 2''-*H*), 1.66 – 1.29 (m, 20H, 2'– 7' -H, 2'' – 7'' -*H*), 0.98 – 0.89 (m, 6H, 8'-*H*, 8''-*H*).

^{13}C NMR (101 MHz, $CDCl_3$) δ [ppm]= 195.4 (C-8), 149.6 (C-5), 142.9 (C-4a), 142.2 (C-13), 134.6 (C-11), 129.9 (C-8a), 129.2 (C-10), 126.6 (C-9), 126.6 (C-12a), 126.2 + 126.0 (C-2, C-3), 125.8 (C-14b), 125.6 (C-12), 122.2 + 121.3 (C-1, C-4), 110.2 (C-14a), 104.1 (C-6), 97.3 (C-6a), 68.6 + 62.57 (C-1') + (C-1'') , 48.3 (C-7), 32.1 + 32.0 (C-3') + (C-3''), 30.2 + 30.1 (C-4' + C-4''), 29.5 + 29.5 + 29.4 + 29.4 (C-2') + (C-2'') +(C-5') + (C-5'') + (C-15), 26.5 + 26.4 (C-6') + (C-6''), 22.8 + 22.8 (C-7', C-7''), 14.3 + 14.3 (C-8', C-8'').

HRMS for $C_{36}H_{46}O_4$ (ESI+) [M+H^{+}]: *m*/*z* calculated 543.3469, found 543.3449.

6.2.8. 7*H*,13*H*-5,13-Di(pent-4-yn-1yloxy)-7,13-methanobenzo[*f*]naphtho[1,2-*b*]oxocin-8-one (**12**)

According to GP2, 4-(Pent-4-yn-1-yl)oxy-1-naphthol (169.6 mg, 0.75 mmol, 1 equiv.) and tetraethylammonium hexafluorophosphate (16.4 mg, 0.06 mmol, 0.08 equiv.) were dissolved in HFIP (5 mL). The crude product was purified by column chromatography (98% → 92% CH in 1 h). The product was obtained as a colorless amorphous solid (148.6 mg, 0.35 mmol, 88%).

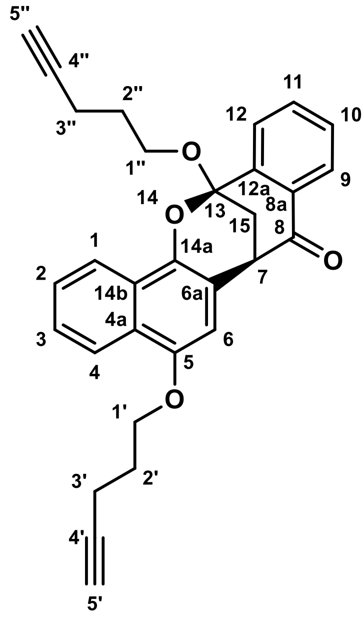

^{1}H NMR (400 MHz, $CDCl_3$) δ [ppm]= 8.18 – 8.10 (m, 2H, 1-*H*, 4-*H*), 8.04 (dd, ^{4}J = 8.0 Hz, 1.2 Hz, 1H, 12-*H*), 7.95 (dd, ^{3}J = 7.9 Hz, ^{4}J = 1.4 Hz, 1H, 9-*H*), 7.63 (ddd, ^{3}J = 7.9, 7.5 Hz, ^{4}J = 1.4 Hz, 1H, 11-*H*), 7.50 – 7.40 (m, 2H, 2-H, 3-*H*), 7.38 (ddd, ^{3}J = 8.0, 7.5 Hz, ^{4}J = 1.2 Hz, 1H, 10-*H*), 6.65 (s, 1H, 6-*H*), 4.27 – 4.12 (m, 3H, 1'-*H*, 1''(1x)-*H*), 4.90 (dd, ^{3}J = 3.7, 2.5 Hz, 1H, 7-*H*) 3.96 – 3.91 (m, 1H, 1'-*H*), 2.73 (dd, ^{3}J = 3.7 Hz, ^{2}J = 12.6 Hz, 1H, 15-*H'*), 2.63 – 2.53 (m, 3H, 15-*H"*, 2''-*H* (coupling constants cannot be detected because of overlap)), 2.51 – 2.44 (m, 4H, 2'-H, 2''-*H*), 2.15 – 2.04 (m, 4H, 3'-H, 3''-*H*), 2.02 + 2.00 (t, ^{3}J = 2.6 Hz, 1H, 5'-*H*, 5''-H).

^{13}C NMR (101 MHz, $CDCl_3$) δ [ppm]= 195.2 (C-8), 149.3 (C-5), 143.0 (C-4a), 141.9 (C-13), 134.6 (C-11), 129.9 (C-8a), 129.3 (C-10), 126.7 (C-9), 126.5 (C-12a), 126.4 + 126.1 (C-2, C-3), 125.8 (C-14b), 125.6 (C-12), 122.1 + 121.5 (C-1, C-4), 110.1 (C-14a), 104.2 (C-6), 97.3 (C-6a), 83.9 + 83.7 (C-4' + C-4''), 69.1 + 69.0 (C-3') + (C-3''), 66.8 (C-1'), 60.9 (C-1''), 48.2 (C-7), 30.0 (C-15), 29.8 (C-4' + C-4'') 29.1 + 29.5 (C-5') + (C-5''), 15.6 + 15.6 (C-2' + C-2'').

HRMS for $C_{30}H_{26}O_4$ (ESI+) $[M+H^+]$: *m/z* calculated 451.1904, found 451.1891.

6.2.9. 7*H*,13*H*-5,13-Di(benzyloxy)-7,13-methanobenzo[*f*]naphtho[1,2-*b*]oxocin-8-one (**13**)

According to GP2, 4-(benzyl)oxy-1-naphthol (187.7 mg, 0.75 mmol, 1 equiv.) and tetraethylammonium hexafluorophosphate (16.4 mg, 0.06 mmol, 0.08 equiv.) were dissolved in HFIP (5 mL). The crude product was purified by column chromatography (97% → 78% CH in 1 h) followed by a reverse column chromatography (85% → 100% MeCN in 1 h). The product was obtained as a blue amorphous solid (75.4 mg, 0.15 mmol, 40%).

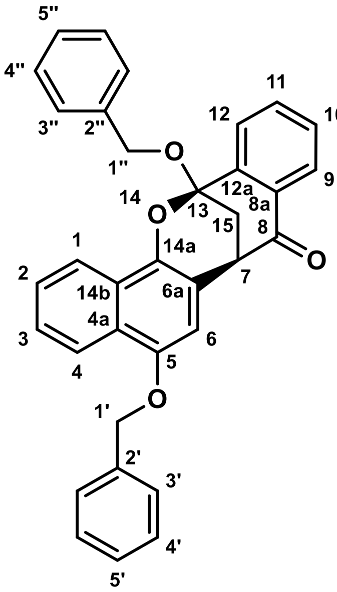

^{1}H NMR (400 MHz, $CDCl_3$) δ [ppm]= 8.25 – 8.20 (m, 1H, 1-*H*/4-*H*), 8.15 – 8.10 (m, 2H, 1-*H*/4-*H* and 12-*H*), 8.00 – 7.95 (m, 1H, 9-*H*), 7.67 – 7.61 (m, 1H, 11-*H*), 7.61 – 7.57 (m, 2H, 3'-*H*), 7.54 – 7.50 (m, 2H, 3''-*H*), 7.49 – 7.35 (m, 9H, 2-*H,* 3-*H*, 10-*H*, 4'-*H*, 4''-*H*, 5'-*H*, 5''-*H*), 6.77 (s, 1H, 6-*H*), 5.25 (d, ^{2}J = 11.5 Hz, 1H, 1''a-*H*), 6.77 (s, 1H, 1'-*H*), 4.92 (d, ^{2}J = 11.5 Hz, 1H, 1''b-*H*), 4.12 (dd, ^{3}J = 3.7, 2.5 Hz, 1H, 7-*H*), 2.81 (dd, ^{3}J = 3.7 Hz, ^{2}J = 12.6 Hz, 1H, 15-*H'*), 2.66 (dd, ^{3}J = 2.5 Hz, ^{2}J = 12.6 Hz, 1H, 15-*H''*).

^{13}C NMR (101 MHz, $CDCl_3$) δ [ppm]= 195.1 (C-8), 149.4 (C-5), 143.2 (C-4a), 141.8 (C-13), 138.4 (C-2''), 137.8 (C-2'), 134.8, 130.0, 129.4, 128.7, 128.7, 128.1, 127.9 (cannot be assigned because of strong overlap in ^{1}H NMR and 2D spectra), 127.8 (C-3''), 127.7 (C-3'), 126.8 (C-12), 126.7, 126.5, 126.3, 125.9 (cannot be assigned because of strong overlap in ^{1}H NMR and 2D spectra), 125.8 (C-9), 122.4 (C-4), 121.4 (C-1), 110.2 (C-14a), 104.7 (C-6), 97.8 (C-6a), 70.6 (C-1'), 64.9 (C-1''), 48.3 (C-7), 30.4 (C-15).

HRMS for $C_{34}H_{26}O_4$ (ESI+) [M+Na$^+$]: *m*/*z* calculated 521.1723, found 521.1722.

6.2.10. 7H,13H-5,13-Difluoro-7,13-methanobenzo[*f*]naphtho[1,2-b]oxocin-8-one (**14**)

For the electrolysis of 4-fluoro-1-naphthol (121.6 mg, 0.75 mmol, 1 equiv.) and tetraethylammonium hexafluorophosphate (16.2 mg, 0.06 mmol, 0.08 equiv.) were dissolved in HFIP (4 mL) and chlorobenzene (1 mL) due to poor solubility in only HFIP. The crude product was purified by column chromatography (99% → 96% CH in 1 h). Traces (16 mg, 0.05 mmol, 7%) of the product were obtained as a colorless amorphous solid. The product does not have the required purity. The mass corresponds to a coupling product (polycycles or homo-coupling product) (321.0734 in ESI–) and the H NMR gives clear indications of the polycycle through the *dd* of the methylene bridge instead of a homo-coupling product.

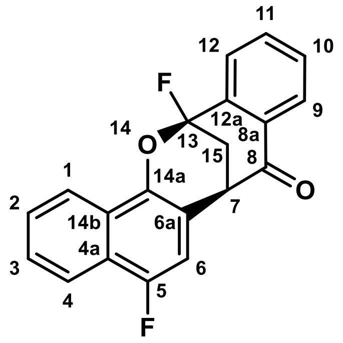

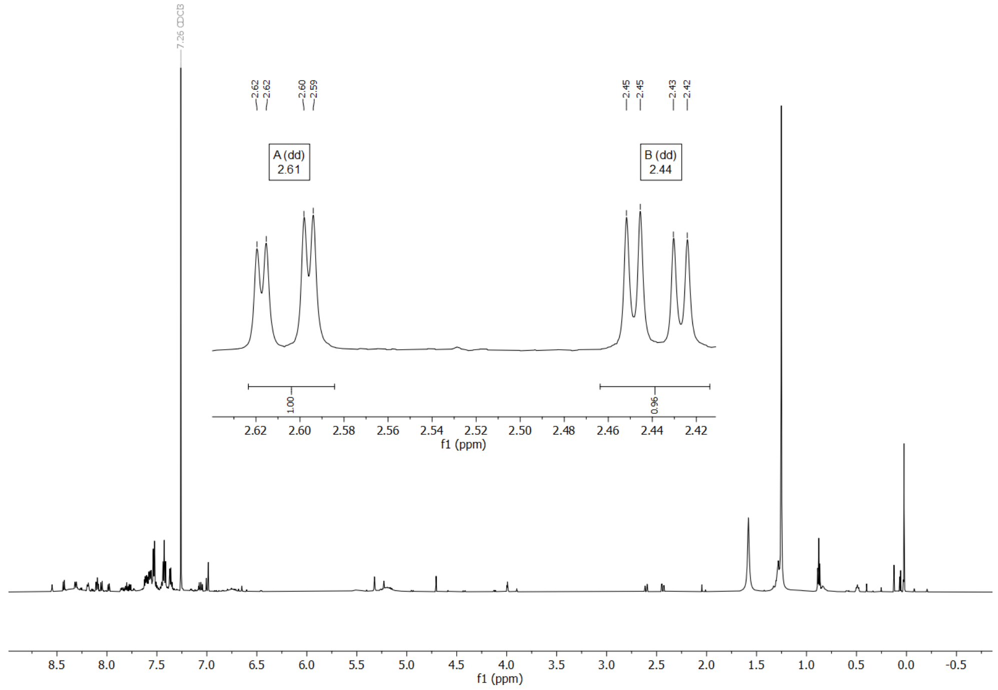

Figure 39: ^{1}H NMR (400 MHz, $CDCl_3$) of **15** showing the *dd* of the methylene bridge motif, indicating a polycyclic formation.

6.2.11. Limitations in the electrolysis of 5-((tri-(1-methylethyl)silyl)oxy)naphthalen-1-ol (**15**)

According to GP2, 5-((tri-(1-methylethyl)silyl)oxy)naphthalen-1-ol (**15**) (237.4 mg, 0.75 mmol, 1 equiv.) and tetraethylammonium hexafluorophosphate (16.3 mg, 0.06 mmol, 0.08 equiv.) were dissolved in HFIP (5 mL). The crude product was purified by column chromatography (99.5% → 80% CH in 32 min). This product was obtained as a colorless amorphous solid (10 mg, 0.015 mmol, 4%). The product was identified as a homo-coupling product (**60**) instead of a polycyclic motif by NMR and HRMS. However, the exact structure cannot be assigned by the gained data.

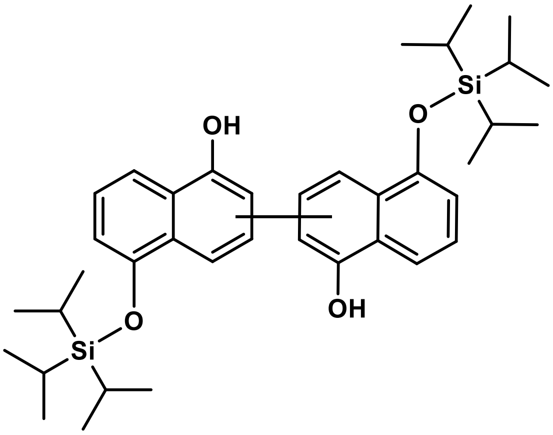

HRMS for $C_{38}H_{54}O_4Si_2$ (ESI+) [M+H$^+$]: *m/z* calculated 630.3633, found 631.3642.

6.2.12. Limitations in the electrolysis of 4-hydroxynaphthalen-1-yl (**16**)

According to GP2, 4-acetoxy-1-naphthol (151.2 mg, 0.75 mmol, 1 equiv.) and tetraethylammonium hexafluorophosphate (16.8 mg, 0.06 mmol, 0.08 equiv.) were dissolved in HFIP (5 mL). The crude product was purified by column chromatography (98% → 70% CH in 1 h) leading to 41.8 mg of **17** with impurities. The crude product was purified by reverse column chromatography (55% → 100% MeCN in 1 h 10 mins). Traces (7 mg, 0.02 mmol, 2%) of 1,4-naphthoquinone **29** was obtained as a yellow amorphous solid. One identified side product was 1-hydroxy-1',4'-dioxo-1',4'-dihydro-[2,2'-binaphthalen]-4-yl acetate (**61**) (10 mg).

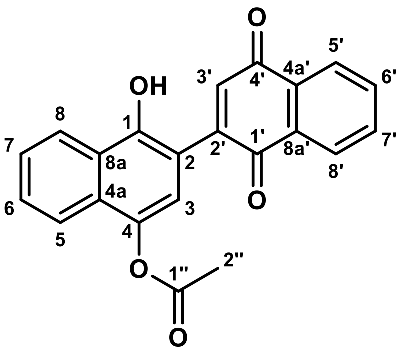

^{1}H NMR (600 MHz, $CDCl_3$) δ [ppm]= 9.28 (s, 1H, 1-O*H*), 8.49 – 8.45 (m, 1H, 5'-*H*), 8.27 – 8.23 (m, 1H, 8'-*H*), 8.14 – 8.10 (m, 1H, 5'-*H*), 7.86 – 7.77 (m, 4H, 8-*H*, 6'-*H*, 7'-*H*), 7.64 – 7.56 (m, 2H, 6-*H*, 7-*H*), 7.11 (s, 2H, 3-*H*, 3'-*H*), 2.46 (s, 3H, 2''-*H*).

^{13}C NMR (151 MHz, $CDCl_3$) δ [ppm]= 189.0 (C-8'), 184.2 (C-9'), 169.8 (C-1''), 150.0 (C-4a), 148.7 (C-1), 140.4 (C-2'), 139.3 (C-3'), 135.0 + 134.1 (C-6') + (C-7'), 132.4 + 131.6 (C-4a' + C-8a'), 129.0 (C-4a), 128.6 (C-7), 127.8 (C-8'), 127.3 (C-8a), 126.6 (C-6), 126.4 (C-5'), 124.5 (C-5), 120.9 (C-8), 119.3 (C-3), 114.3 (C-2), 21.0 (C-2'').

HRMS for $C_{22}H_{14}O_5$ (ESI+) [M–H$^-$]: *m/z* calculated 357.0768, found 357.0770.

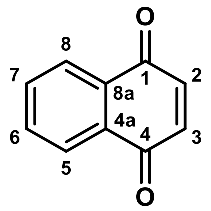

^{1}H NMR (400 MHz, $CDCl_3$) *δ* [ppm] = δ 8.12 – 8.03 (m, 2H, 6-*H*, 7-*H*), 7.80 – 7.69 (m, 2H, 5-*H*, 8-*H*), 6.98 (s, 2H, 2-*H*, 3-*H*).

^{13}C NMR (151 MHz, $CDCl_3$) *δ* [ppm]= 185.2 (C-1, C-4), 138.8 (C-2, C-3), 134.1 (C-5, C-8), 132.0 (C-4a, C-8a), 126.5 (C-6, C-7).

6.2.13. Further investigated limitations

Beside the products that were acquired in traces, following GP2, 4-bromo-1-naphthol (**17**) (143.6 mg, 0.64 mmol, 1 equiv.) and tetraethylammonium hexafluorophosphate (13.1 mg, 0.05 mmol, 0.07 equiv.) were dissolved in HFIP (5 mL). The electrolysis was carried out at rt in a batch-type Teflon® cell due limited solubility (electrochemical parameter: 10 mA/cm^2 (15.3 mA), 1 *F* (72.4 C)). After the electrolysis the crude project was analysed with LCMS and TLC. Due to the poor solubility most of the starting material was not converted. Furthermore, no products with the molecular weight or corresponsive NMR signals for the homo-coupling or polycyclic products were observed.

Following GP2, 3-chloro-4-methoxynaphthalen-1-ol, 3-methyl-4-methoxynaphthalen-1-ol and 3-ethyl-4-methoxynaphthalen-1-ol did not lead to the expected polycyclic motif. Instead, the homo-coupling **62** product was obtained as main product. Further, a dimeric product with loss of the methoxy groups, as well as the formation of two carbons with a shift of >180 ppm can be observed. These groups can be assumed to be two non-identical carbonyl groups. Therefore, we assume that structure **63** is the main product of those reactions.

According to GP2, 4-methoxy-3-methyl-1-naphthol (94.1 mg, 0.5 mmol, 1 equiv.) and tetraethylammonium hexafluorophosphate (17.2 mg, 0.06 mmol, 0.12 equiv.) were dissolved in HFIP (5 mL). The crude product was purified by column chromatography (Silica: 96%→70% CH in 50 min).

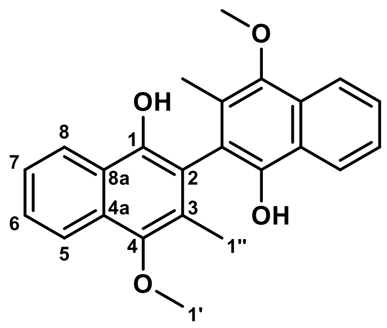

Figure S40: Proposed homo-coupling product (**62**).

^{1}H NMR (400 MHz, $CDCl_3$) δ [ppm]= 8.19 (dt, ^3J = 8.3 Hz, ^4J = 1.1 Hz, 1H, 5-*H*), 8.00 (dt, ^3J = 8.3 Hz, ^4J = 1.1 Hz, 1H, 8-*H*), 7.53 (td, ^3J = 6.7 Hz, ^4J = 1.3 Hz, 1H, 7-*H*), 7.44 (td, ^3J = 6.7 Hz, ^4J = 1.3 Hz, 1H, 6-*H*), 3.81 (s, 3H, 1'-*H*), 1.93 (s, 3H, 1''-*H*).

^{13}C NMR (101 MHz, $CDCl_3$) δ [ppm]= 146.4 (C-1, C-4), 128.0 (C-4a), 126.5 (C-3), 126.1 (C-7), 124.7 (C-8a), 124.2 (C-6), 122.9 (C-5), 121.4 (C-8), 118.87 (C-2), 61.0 (C-1'), 13.2 (C-1'').

HRMS for $C_{24}H_{22}O_4$ (ESI–) [M–H$^+$]: *m*/*z* calculated 373.1445, found 373.1433.

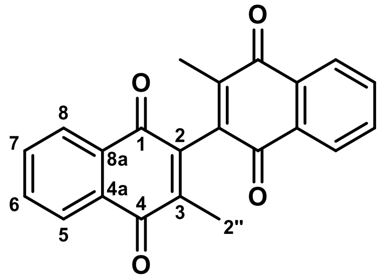

Figure S41: Proposed diketone-product (**63**).

^{1}H NMR (400 MHz, $CDCl_3$) δ [ppm]= 8.18 – 8.10 (m, 1H, 8-*H*), 8.12 – 8.06 (m, 1H, 5-*H*), 7.82 – 7.72 (m, 2H, 6-*H*, 7-*H*), 2.06 (s, 3H, 1''-*H*).

^{13}C NMR (101 MHz, $CDCl_3$) δ [ppm]= 184.6 (C-4), 182.8 (C-1), 145.9 (C-2), 140.7 (C-3), 134.1 + 134.0 (C-6 + C-7), 132.3 + 132.0 (C-4a + C-8a), 126.8 + 126.8 (C-5 + C-8), 14.52 (C-1').

HRMS for $C_{24}H_{14}O_4$ (ESI–) [M–H$^-$]: *m*/*z* calculated 341.1445, found 341.1437.

According to GP2, the corresponding phenol (0.75 mmol, 1 equiv.) and tetraethylammonium hexafluorophosphate (17.2 mg, 0.06 mmol, 0.12 equiv.) were dissolved in HFIP (5 mL). The crude mixture was investigated by GC-MS. Notably the anode showed a way more president layer could just be removed by extensive sanding. Since the mass of the coupling product was just observed using phenol **25**, only this reaction was purified by column chromatography (Silica: 99%→98% CH in 50 min). Hereby one fraction (6 mg) containing the characteristic signal of the bridge protons, but mainly the starting material can be isolated (Figure S46). The conversion of the reaction was 96%. Isolation of the polycyclic product out of this fraction was

not possible. Scaling up this reaction 10 times: 2-*tert*-butyl-4-methoxyphenol (1.352 g; 7.5 mmol, 1 equiv.) and tetraethylammonium hexafluorophosphate (172 mg, 0.6 mmol, 0.12 equiv.) in HFIP (50 mL), no accumulation of the polycyclic product was observed. We assume this is due to passivation of the electrode by the formed polymers. Therefore, the formation of the product can just be assumed by the given NMR and GC-MS.

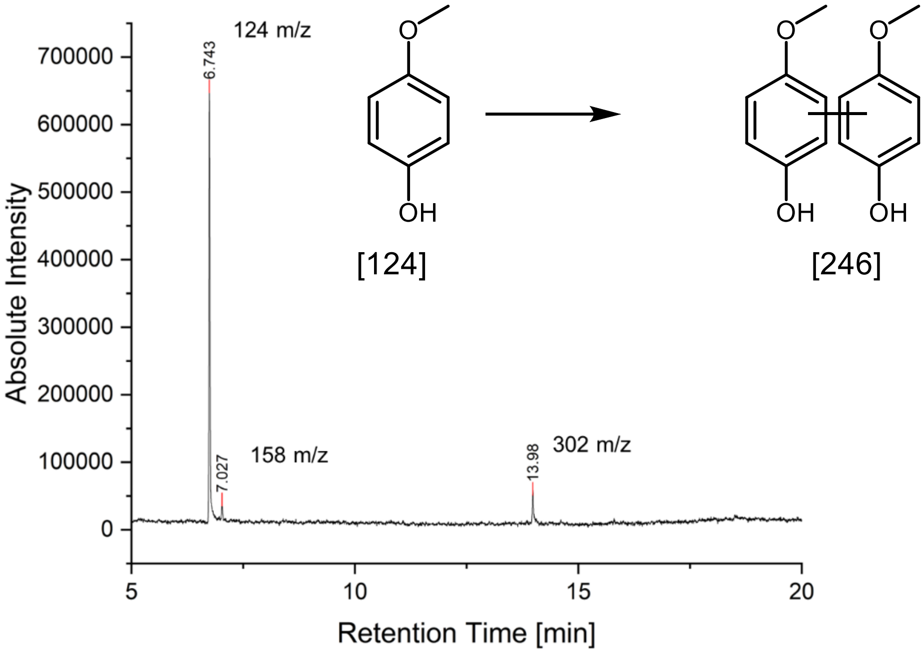

Figure S42: GC-MS Spectrum of the crude mixture after the electrolysis of **22**. M/z ratio of the molecule peak is indicated.

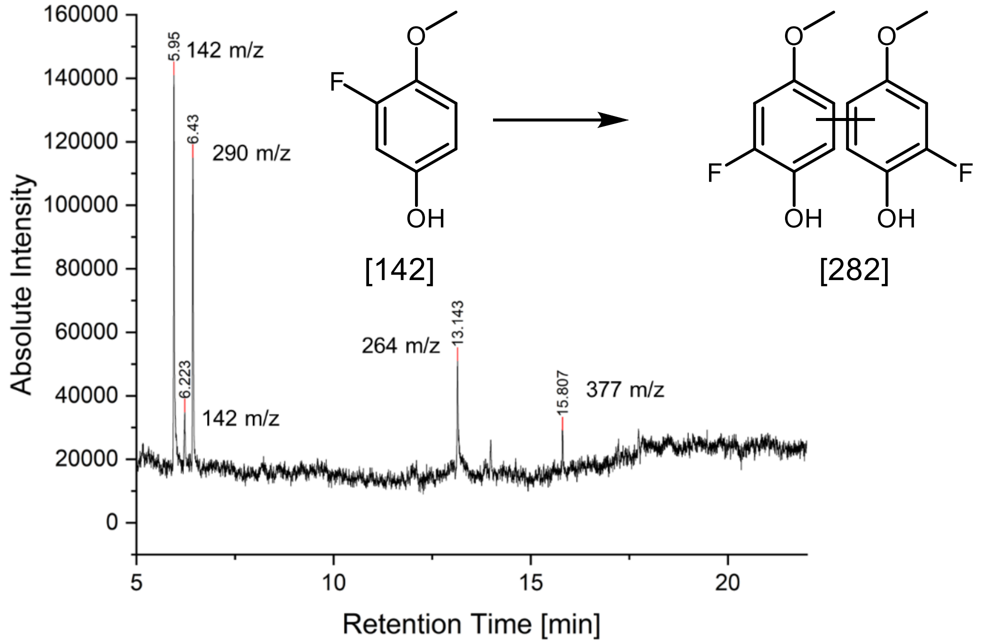

Figure S43: GC-MS Spectrum of the crude mixture after the electrolysis of **23**. M/z ratio of the molecule peak is indicated.

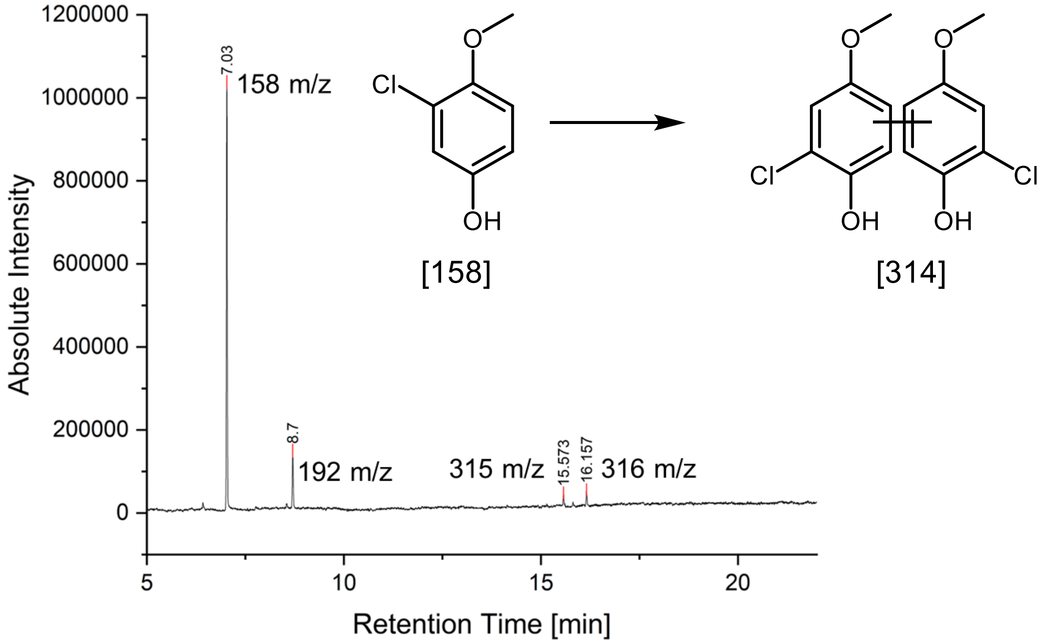

Figure S44: GC-MS Spectrum of the crude mixture after the electrolysis of **24**. M/z ratio of the molecule peak is indicated.

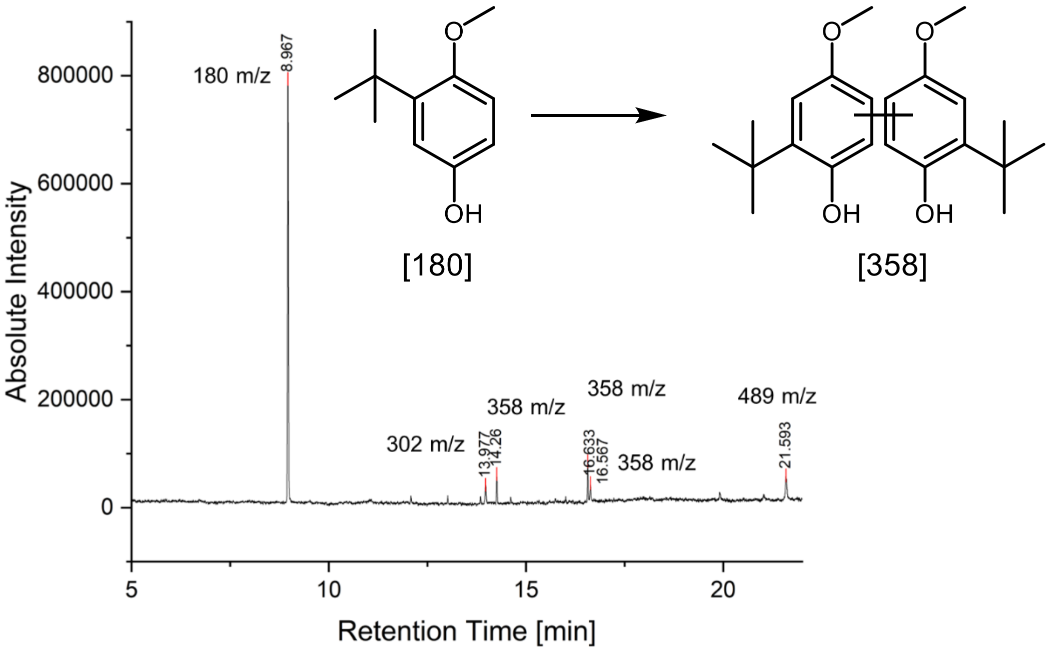

Figure S45: GC-MS Spectrum of the crude mixture after the electrolysis of **25**. M/z ratio of the molecule peak is indicated.

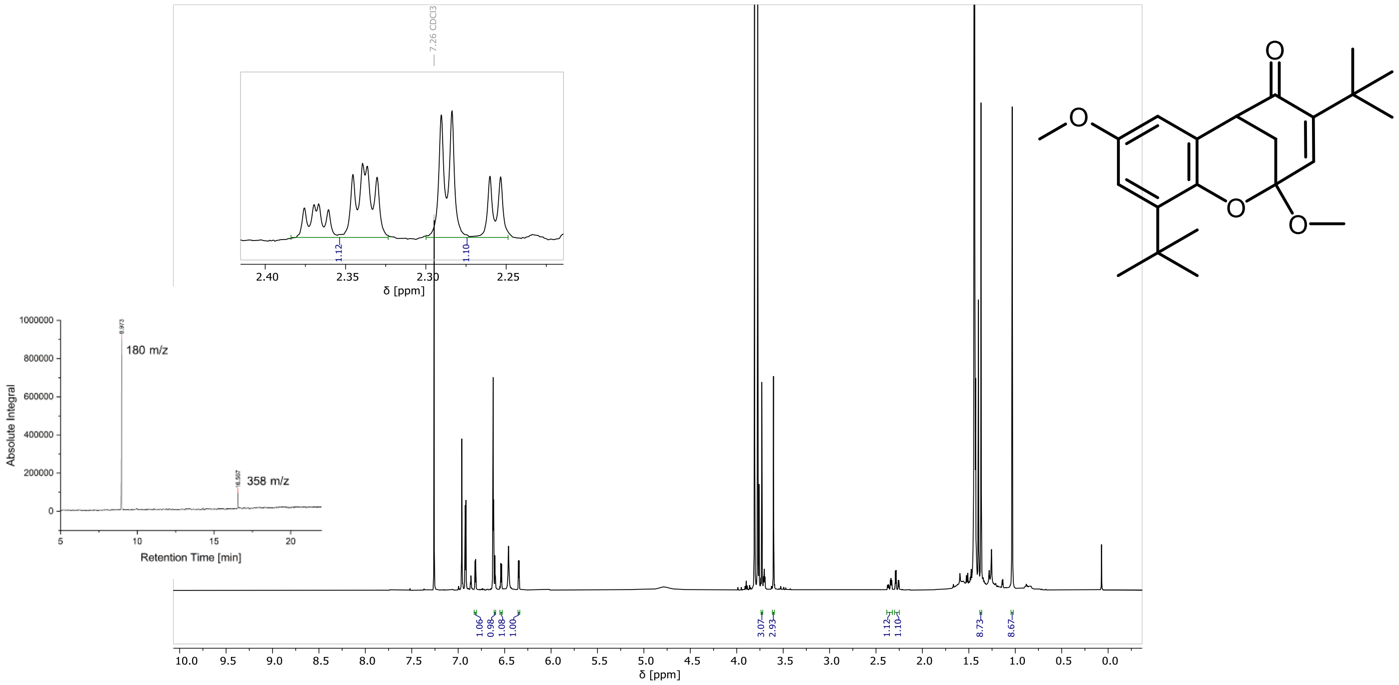

Figure 46: ^{1}H NMR ($CDCl_3$, 400 MHz) of the isolated fraction, containing mostly starting material. Signals that correspond to the polycycle are integrated. The peaks, corresponding to the bridge protons have been highlighted in the zoom window. The new coupling visible in the signal at 2.35 ppm corresponds to the interaction with the proton at the double bound of the newly formed Michael-system. Furthermore, GC-MS of this fraction is shown.

7 Crystallographic data

7.1 7*H*,13*H*-5,13-Dimethoxy-7,13-methanobenzo[*f*]naphtho[1,2-*b*]oxocin-8-one (**2**)

Table 15: Summary of the crystallographic data for **2**.

Molecular structure:	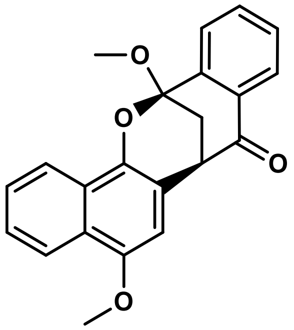
Molecular structure:	Thermal ellipsoids are depicted at 50% probability

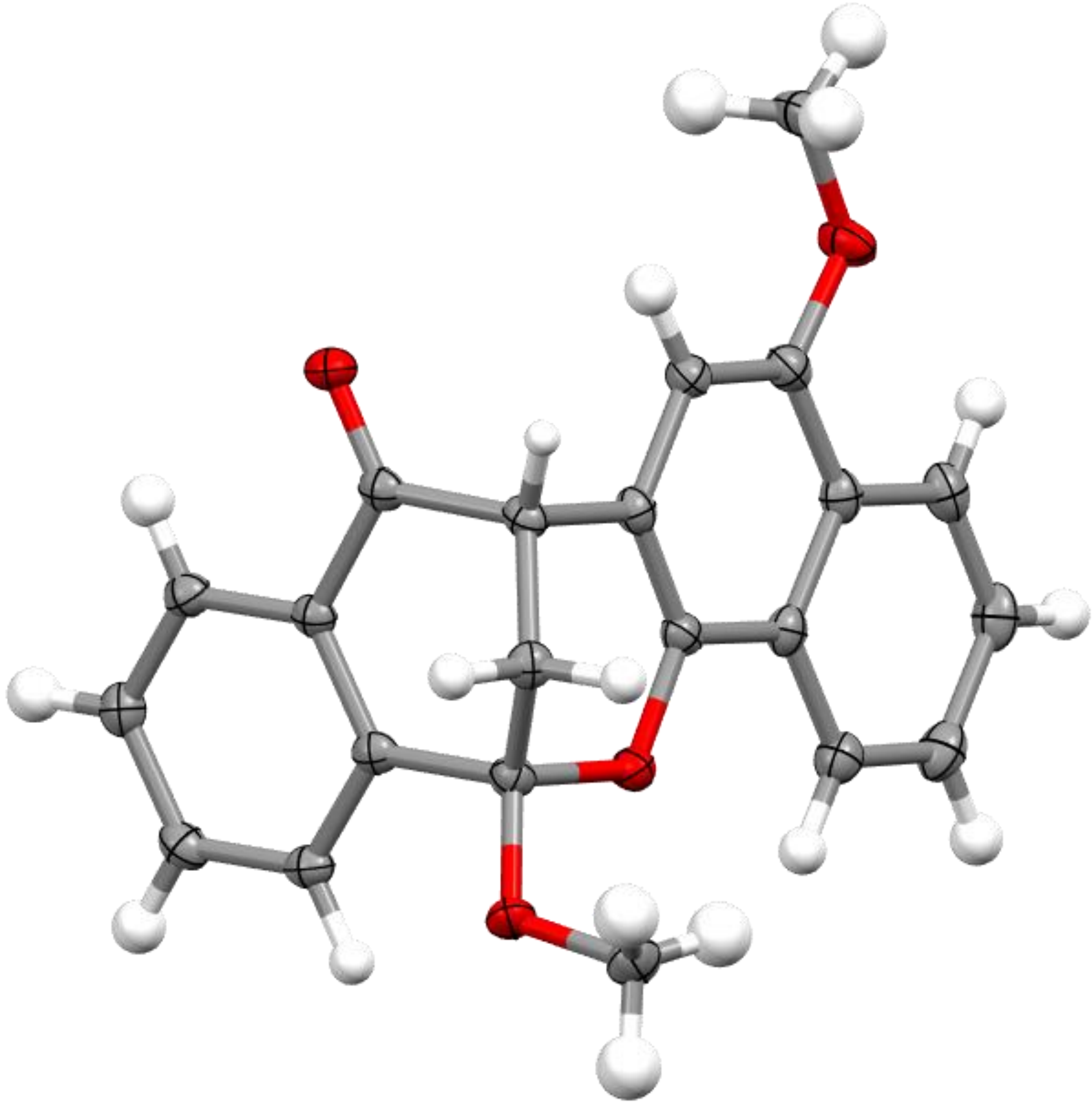

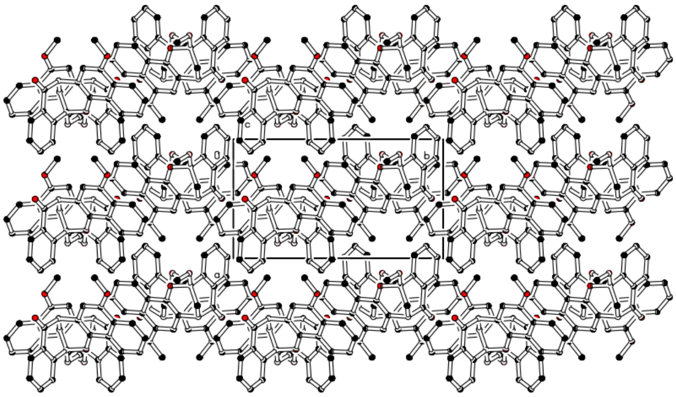

CCDC Number	2377041
Empirical formular	$C_{22}H_{18}O_4$
Moiety formular	$C_{22}H_{18}O_4$
Formular weight	346.36 g·mol^{-1}
Temperature	120(2) K
Wavelength, radiation type	0.71073Å, MoKα
Diffractometer	STOE IPDS 2T
Crystal system	monoclinic
Space group name, number	P 2_1/c, (14)
Unit cell dimensions	a = 8.3250(5) Å
	b = 14.4876(6) Å, β = 98.928(5) °
	c = 13.7446(8) Å
Volume	1637.64(15) Å^3
Number of reflections	9188
And range used for lattice parameters	2.81° ≤ Θ ≤ 28.36°
Z	4
Density (calculated)	1.405 Mg/m^3
Absorption coefficient	0.096 mm^{-1}
Absorption correction	none
F(000)	728
Crystal size, color and form	0.180 · 0.230 · 0.290 mm^3, colorless block
Theta range for data collection	2.812 to 27.903°.
Index ranges	-10 ≤ h ≤ 10, -16 ≤ k ≤ 18, -18 ≤ l ≤ 13
Number of reflections:	

collected	8075
independent	3861 [R_{int} = 0.0230]
observed [I>2sigma(I)]	3171
Completeness to theta = 25.2°	99.3%
Refinement method	Full-matrix least-squares on F^2
Data / restraints / parameters	3861 / 0 / 302
Goodness-of-fit on F^2	1.059
Final R indices [I>2sigma(I)]	R1 = 0.0486, wR2 = 0.1054
R indices (all data)	R1 = 0.0630, wR2 = 0.1148
Largest diff. peak and hole	0.399 und -0.221 eÅ^{-3}

7.2 7*H*,13*H*-5,13-Dimethoxy-7,13-ethanobenzo[*f*]naphtho[1,2-*b*]oxocin-8-one (**6**)

Table 16: Summary of the crystallographic data for **6**.

Molecular structure:

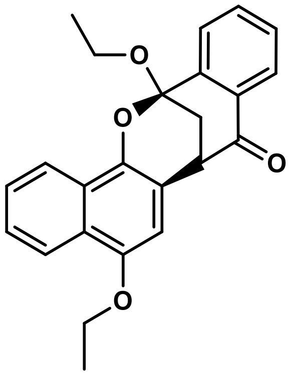

Molecular structure: Thermal ellipsoids are depicted at 50% probability

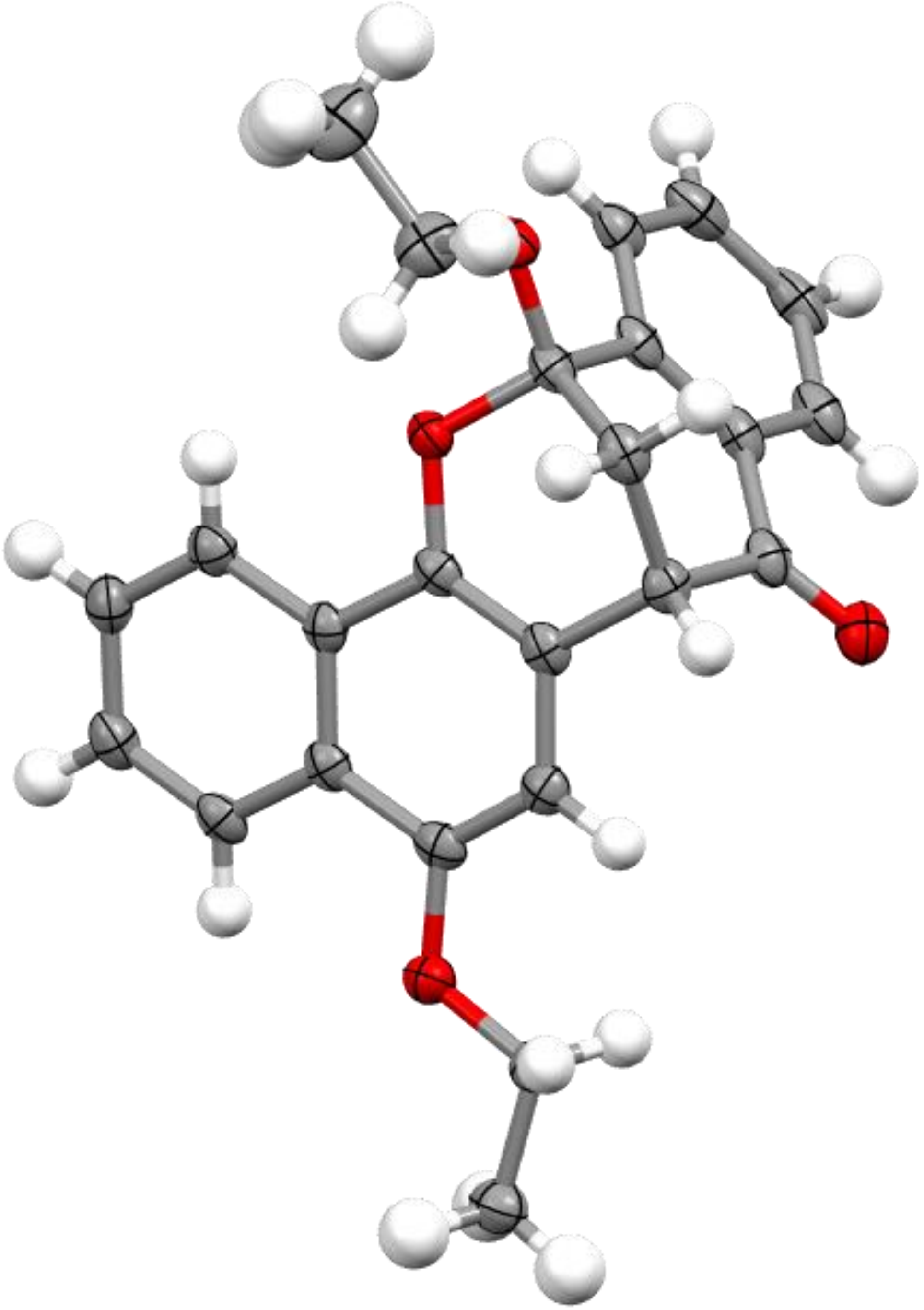

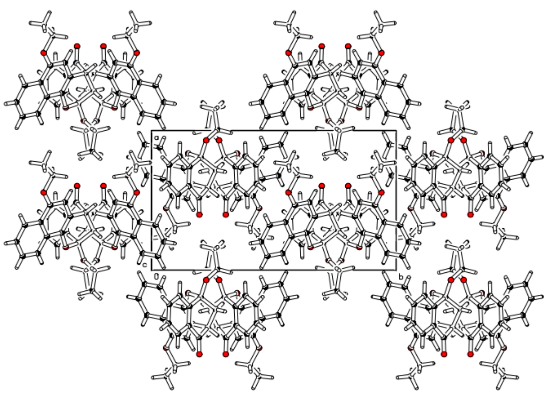

CCDC Number	2377042
Empirical formular	$C_{24}H_{22}O_4$
Moiety formular	$C_{24}H_{22}O_4$
Formular weight	374.41 g·mol^{-1}
Temperature	120(2) K
Wavelength, radiation type	1.54178 Å, CuKα
Diffractometer	STOE STADIVARI
Crystal system	monoclinic
Space group name, number	P 2_1/c, (14)
Unit cell dimensions	a = 10.3738(4) Å, α = 90°
	b = 17.8252(6) Å, β = 101.542(3)°
	c = 10.6640(4) Å, γ = 90°
Volume	1932.06(12) Å^3
Number of reflections	8865
And range used for lattice parameters	4.35° ≤ Θ ≤ 68.46°
Z	4
Density (calculated)	1.287 Mg/m^3
Absorption coefficient	0.701 mm^{-1}
Absorption correction	none
F(000)	792
Crystal size, color and form	4.350 · 0.130 · 0.110 mm^3, colorless block
Theta range for data collection	4.350 to 67.906 °.
Index ranges	-12 ≤ h ≤ 12, -21 ≤ k ≤ 21, -12 ≤ l ≤ 12
Number of reflections:	
collected	13858
independent	3480 [R_{int} = 0.0438]
observed [I>2sigma(I)]	2272
Completeness to theta = 25.2°	99.3%

Refinement method	Full-matrix least-squares on F^2
Data / restraints / parameters	3480 / 0 / 255
Goodness-of-fit on F^2	0.905
Final R indices [I>2sigma(I)]	R1 = 0.0434, wR2 = 0.1002
R indices (all data)	R1 = 0.0735, wR2 = 0.1096
Largest diff. peak and hole	0.333 und -0.256 eÅ^{-3}

7.3 7*H*,13*H*-5,13-Dimethoxy-(7,13- pent-4-yn-1yloxy)benzo[*f*]naphtho[1,2-*b*]oxocin-8-one (**12**)

Table 17: Table 16: Summary of the crystallographic data for **12**.

Molecular structure:

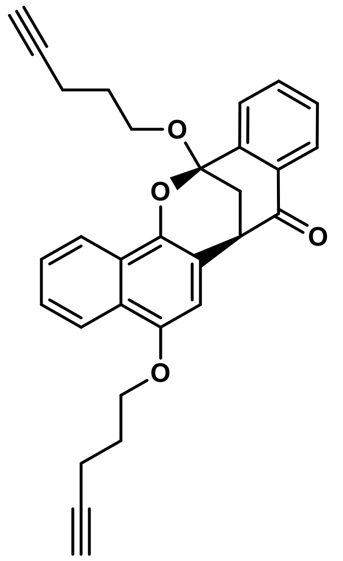

Molecular structure: Thermal ellipsoids are depicted at 50% probability

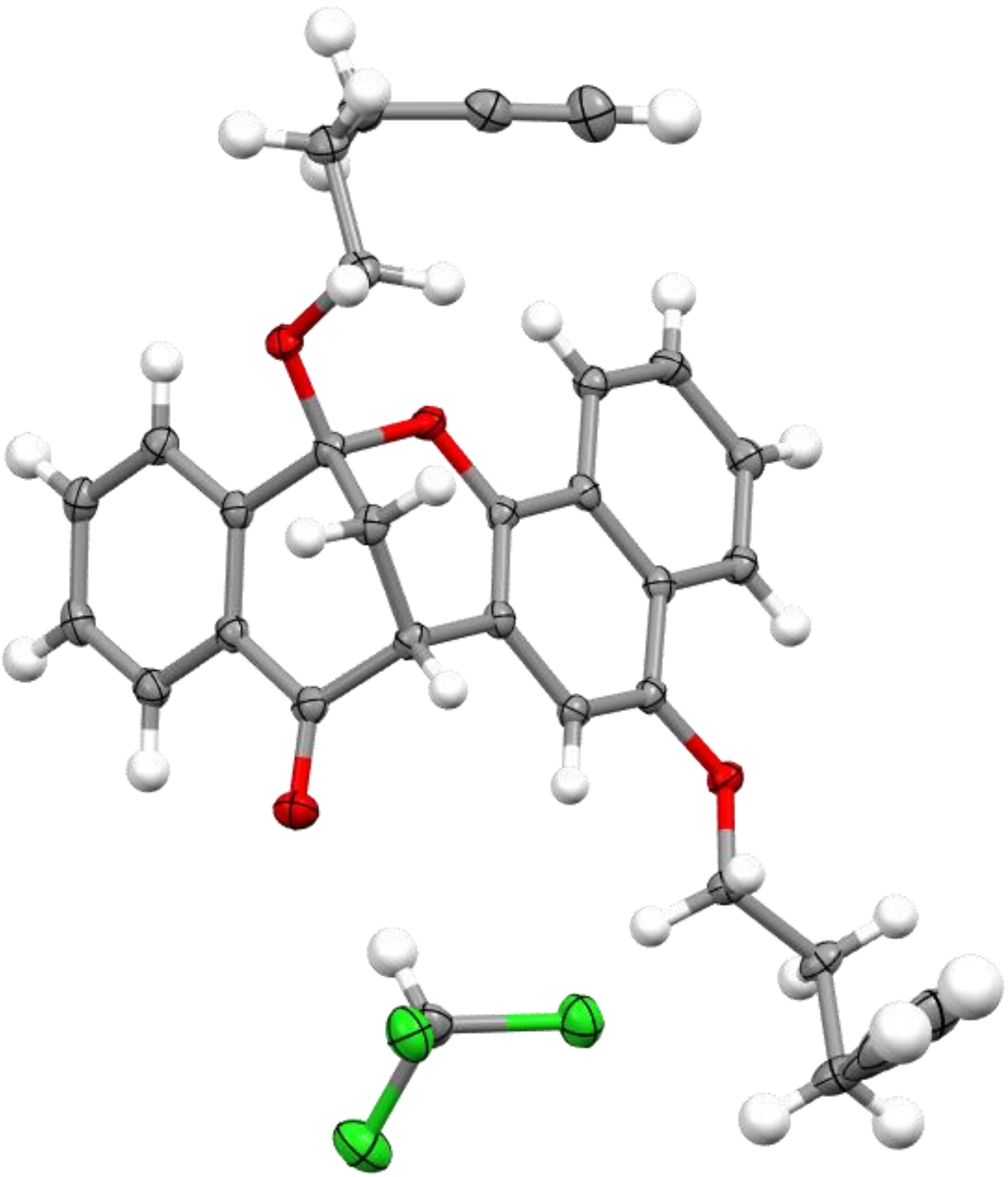

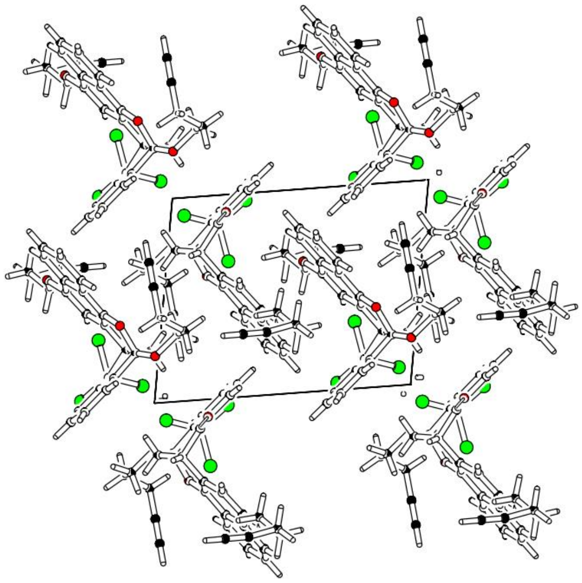

CCDC Number	2377043
Empirical formular	$C_{31}H_{27}Cl_3O_4$
Moiety formular	$C_{30}H_{26}O_4$, $CHCl_3$
Formular weight	569.87 g·mol^{-1}
Temperature	120(2) K
Wavelength, radiation type	0.71073 Å, MoKα
Diffractometer	STOE IPDS 2T
Crystal system	triclinic
Space group name, number	P -1, (2)
Unit cell dimensions	a = 10.0901(5) Å, α = 100.262(4)°
	b = 11.8574(7) Å, β = 112.300(4)°
	c = 12.7877(7) Å, γ = 94.444(4)°
Volume	1375.08(14) Å^3
Number of reflections	13849
And range used for lattice parameters	2.60° ≤ Θ ≤ 28.66°
Z	2
Density (calculated)	1.376 Mg/m^3
Absorption coefficient	0.369 mm^{-1}
Absorption correction	Integration
Max. and min. transmission	0.9446 and 0.8744
F(000)	592
Crystal size, color and form	0.170 · 0.270 · 0.350 mm^3, colorless block
Theta range for data collection	2.601 to 28.255 °.
Index ranges	-13 ≤ h ≤ 13, -14 ≤ k ≤ 15, -17 ≤ l ≤ 16

Number of reflections:	
collected	12490
independent	6742 [R_{int} = 0.0251]
observed [I>2sigma(I)]	5434
Completeness to theta = 25.2°	99.5%
Refinement method	Full-matrix least-squares on F^2
Data / restraints / parameters	6742 / 15 / 352
Goodness-of-fit on F^2	1.031
Final R indices [I>2sigma(I)]	R1 = 0.0428, wR2 = 0.0979
R indices (all data)	R1 = 0.0582, wR2 = 0.1068
Largest diff. peak and hole	0.412 und -0.299 eÅ^{-3}
Additional information	One *C≡CH* group slightly misaligned

8 NMR spectra

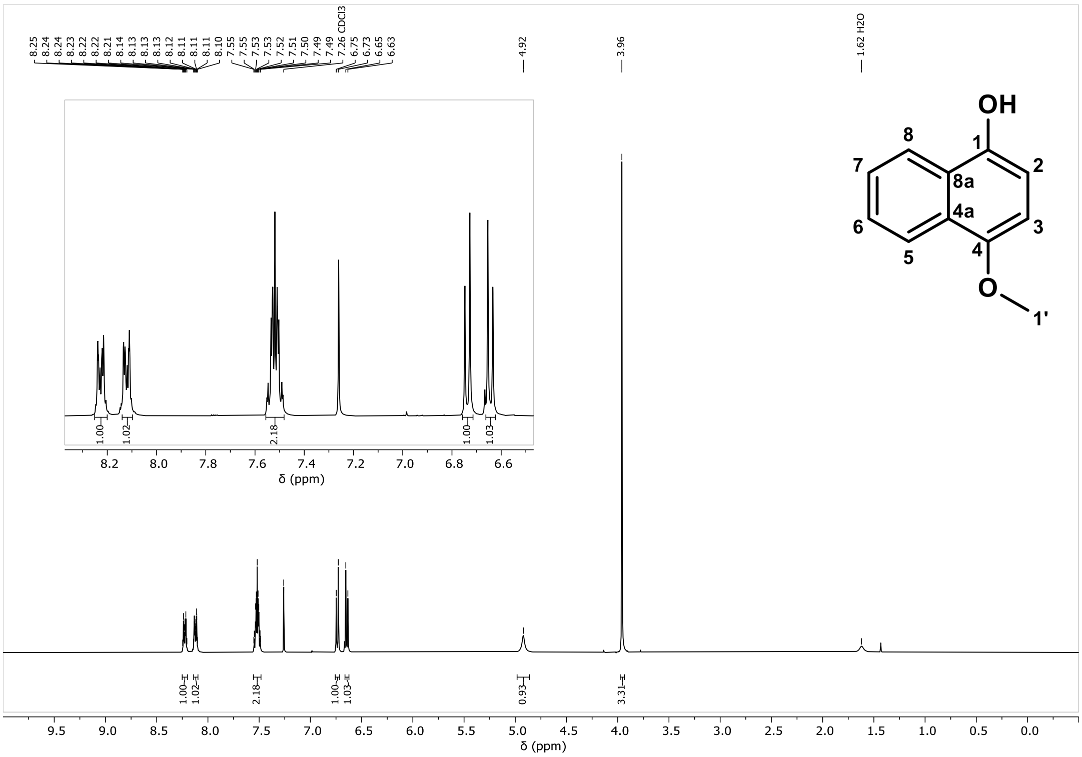

^{1}H NMR spectrum (400 MHz, $CDCl_3$) of **1**.

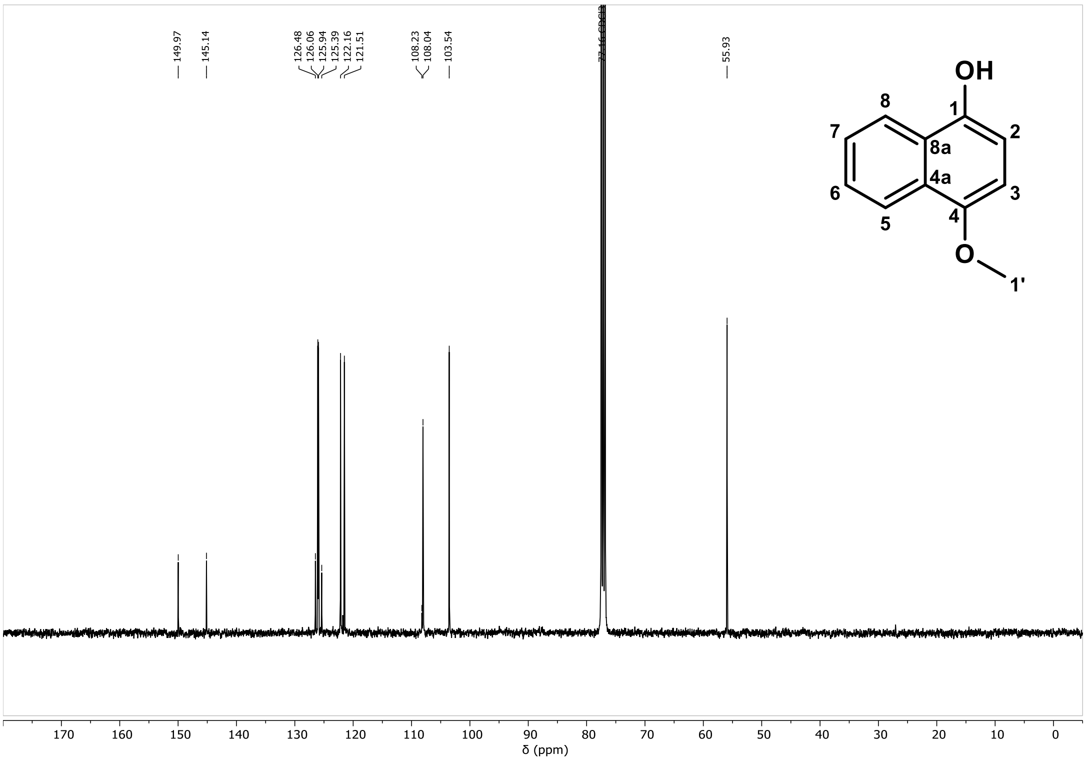

^{13}C NMR spectrum (101 MHz, $CDCl_3$) of **1**.

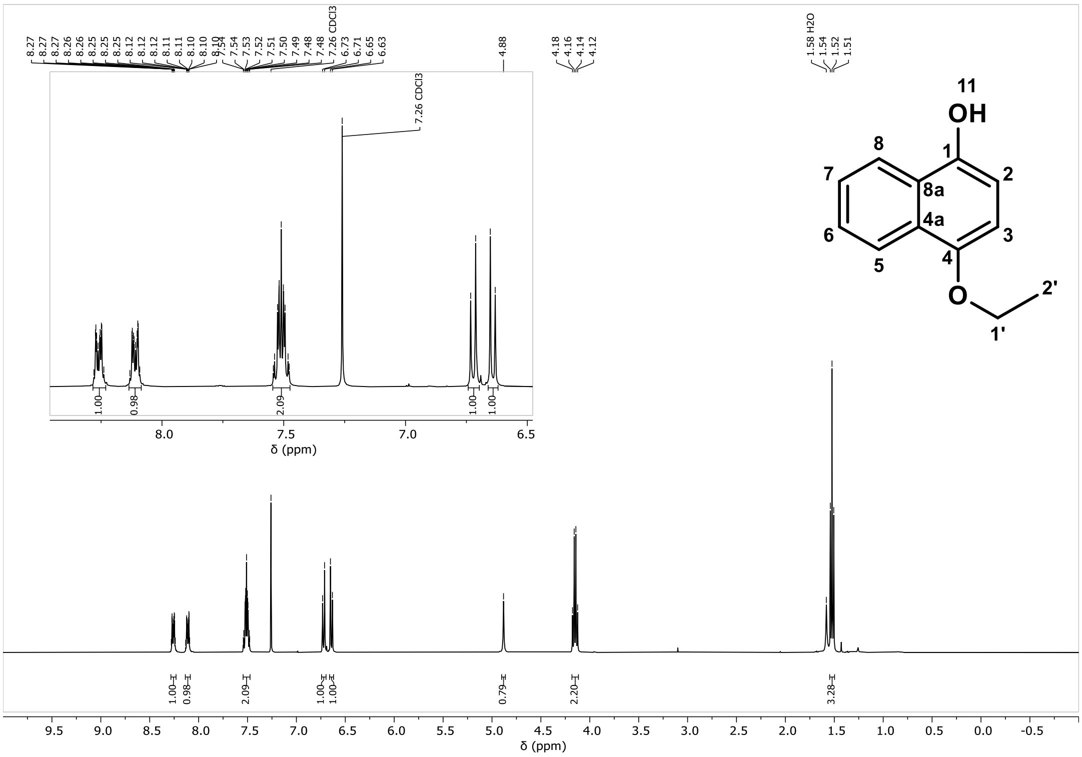

^{1}H NMR spectrum (400 MHz, $CDCl_3$) of **37**.

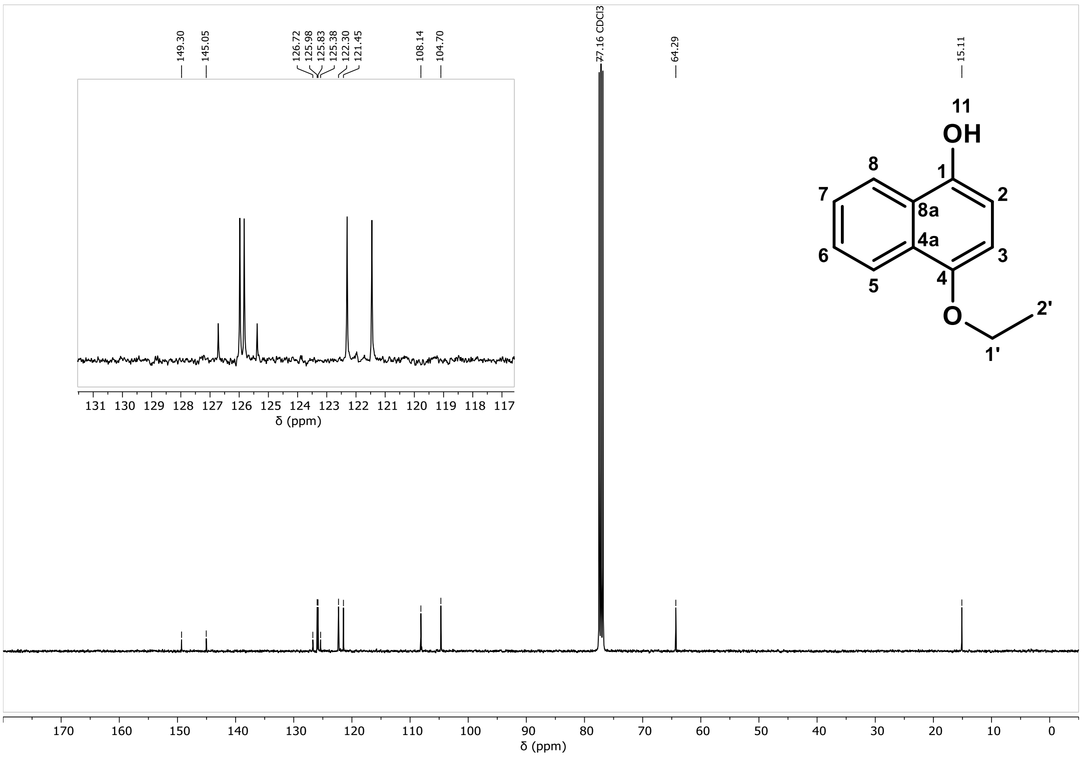

^{13}C NMR spectrum (101 MHz, $CDCl_3$) of **37**.

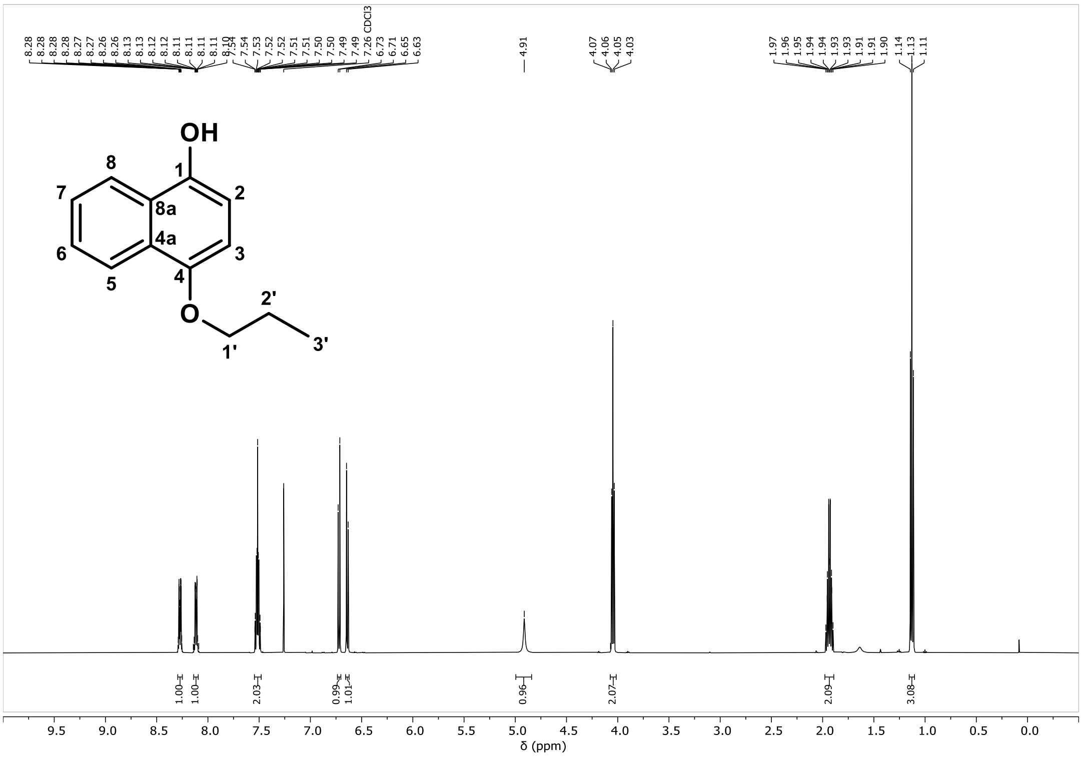

^{1}H NMR spectrum (500 MHz, $CDCl_3$) of **36**.

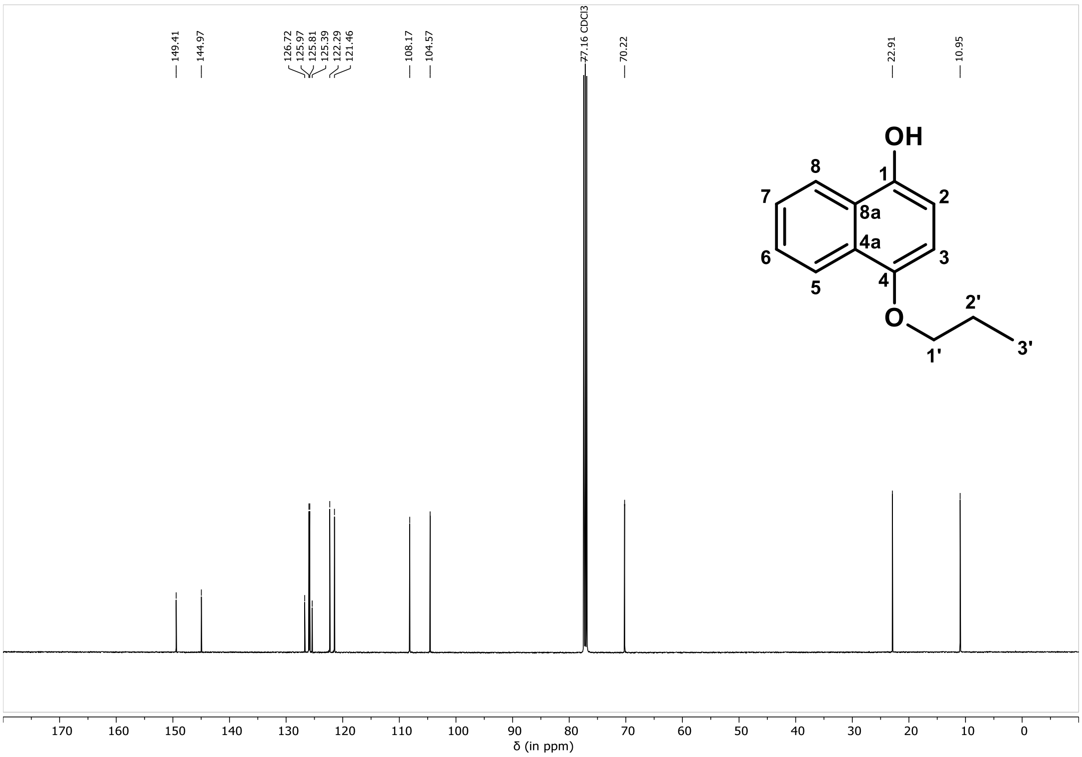

^{13}C NMR spectrum (126 MHz, $CDCl_3$) of **36**.

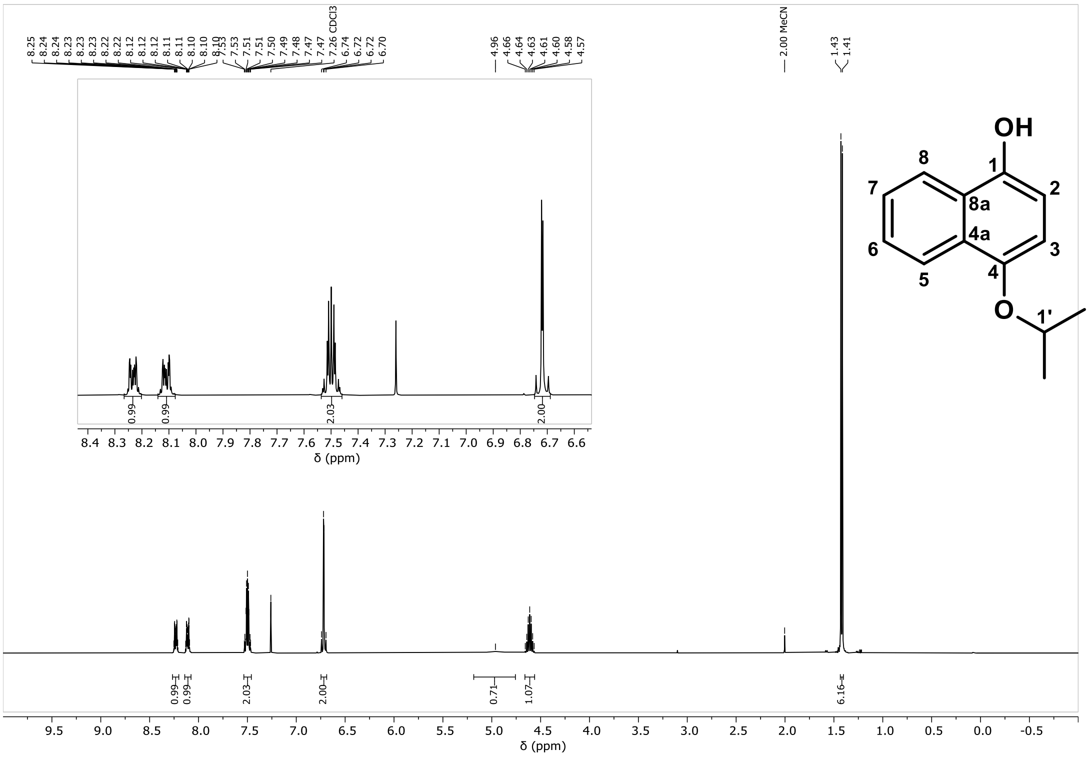

^{1}H NMR spectrum (400 MHz, $CDCl_3$) of **38**.

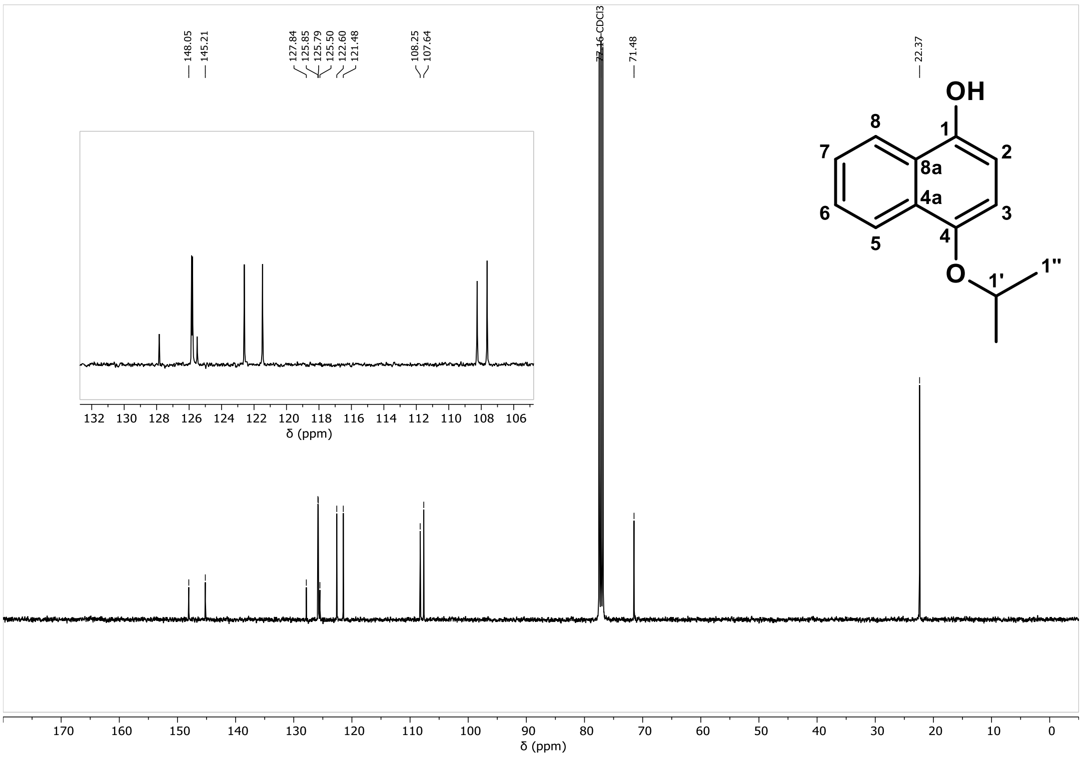

^{13}C NMR spectrum (101 MHz, $CDCl_3$) of **38**.

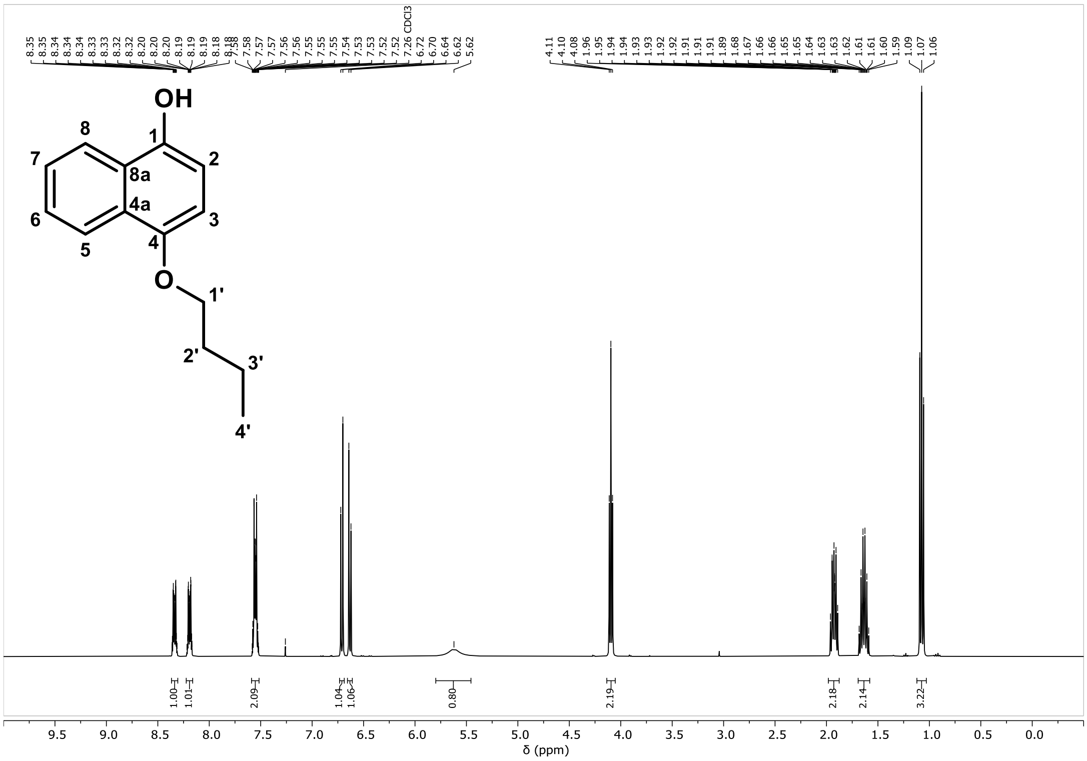

^{1}H NMR spectrum (500 MHz, $CDCl_3$) of **39**.

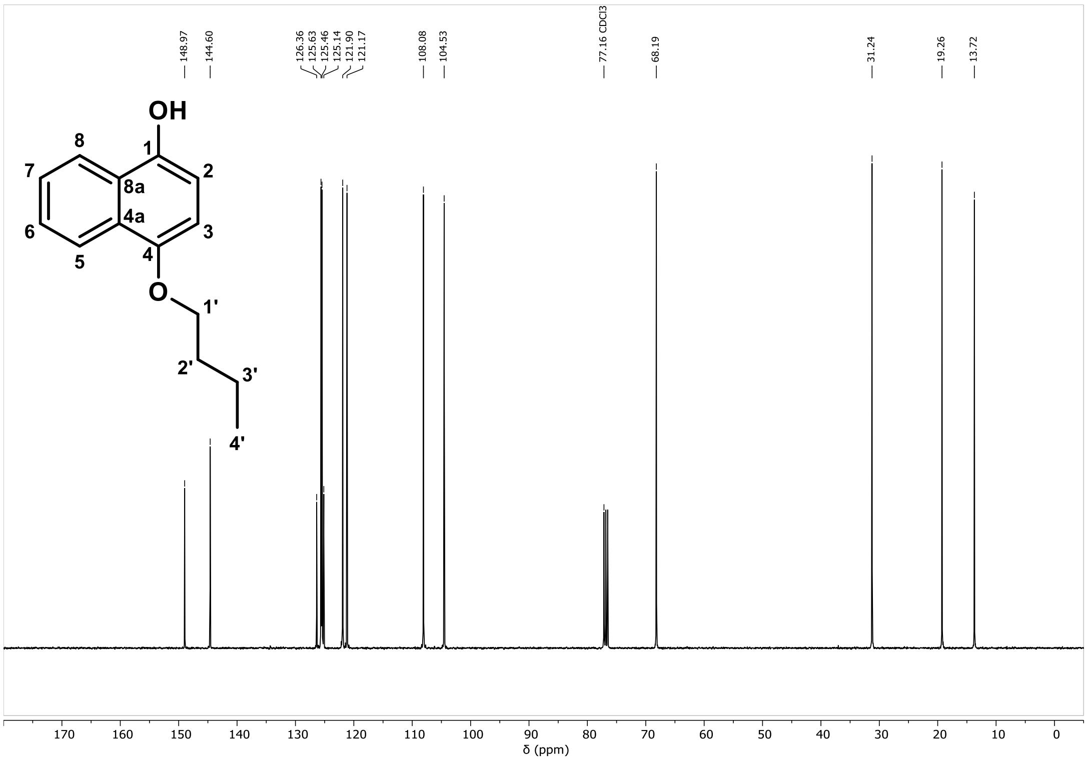

^{13}C NMR spectrum (126 MHz, $CDCl_3$) of **39**.

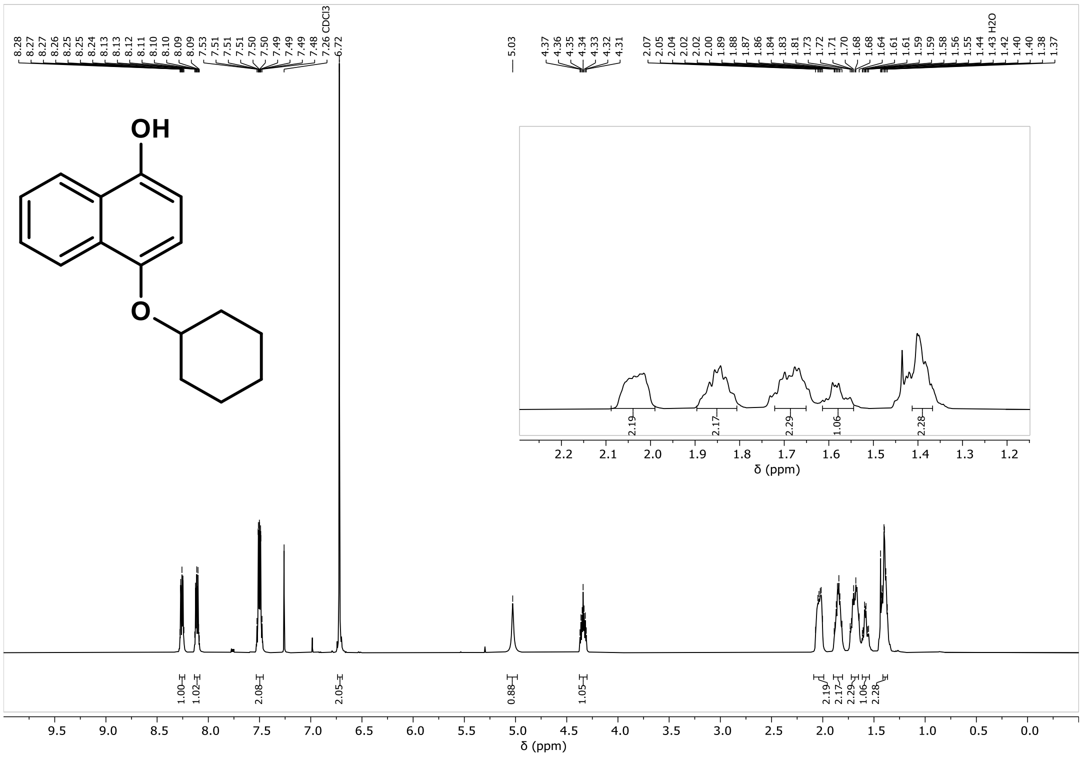

^{1}H NMR spectrum (400 MHz, $CDCl_3$) of **40**.

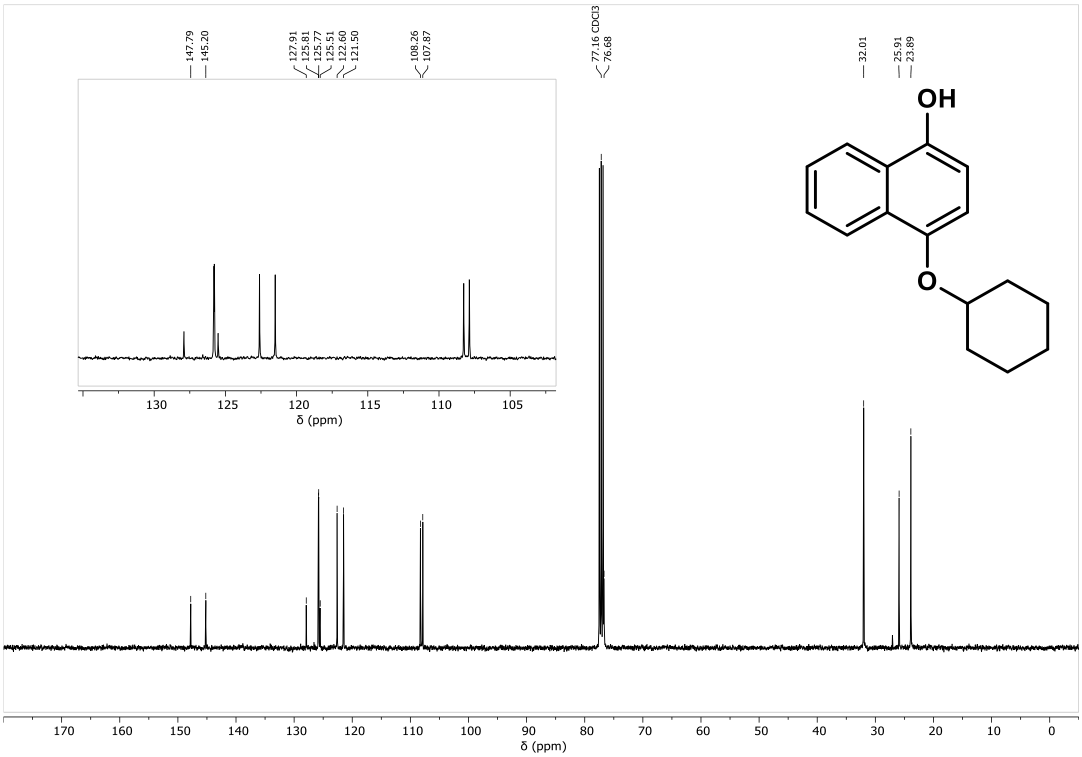

^{13}C NMR spectrum (101 MHz, $CDCl_3$) of **40**.

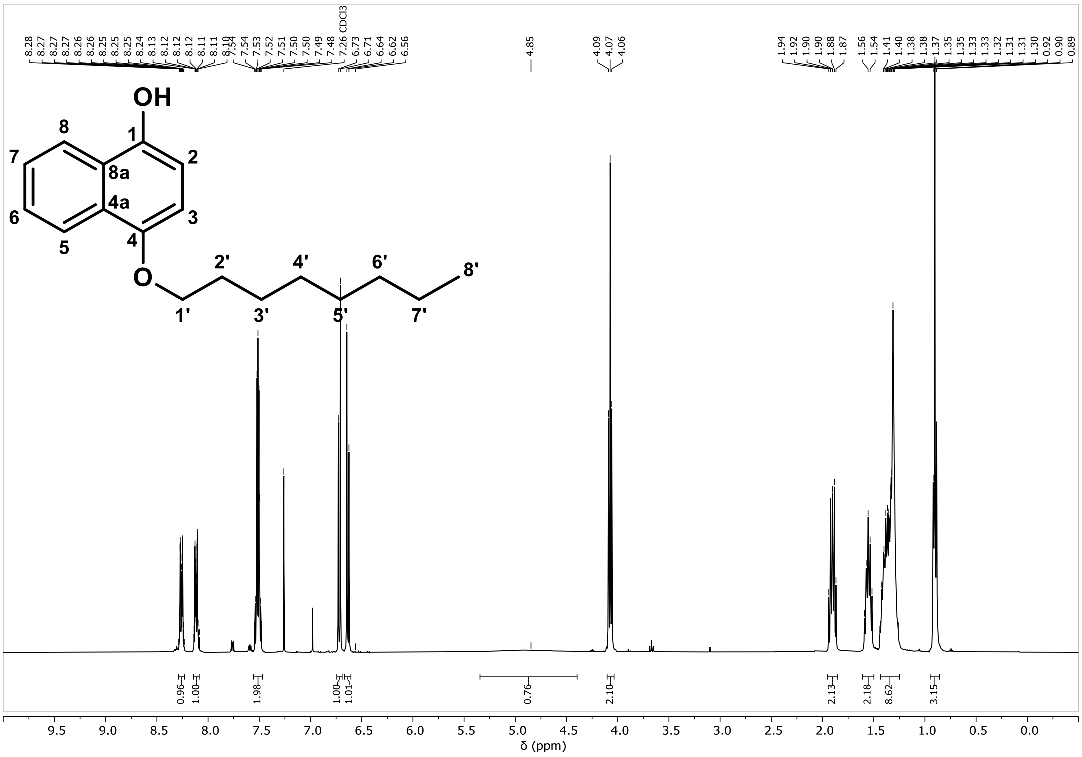

^{1}H NMR spectrum (400 MHz, CDCl$_3$) of **41**.

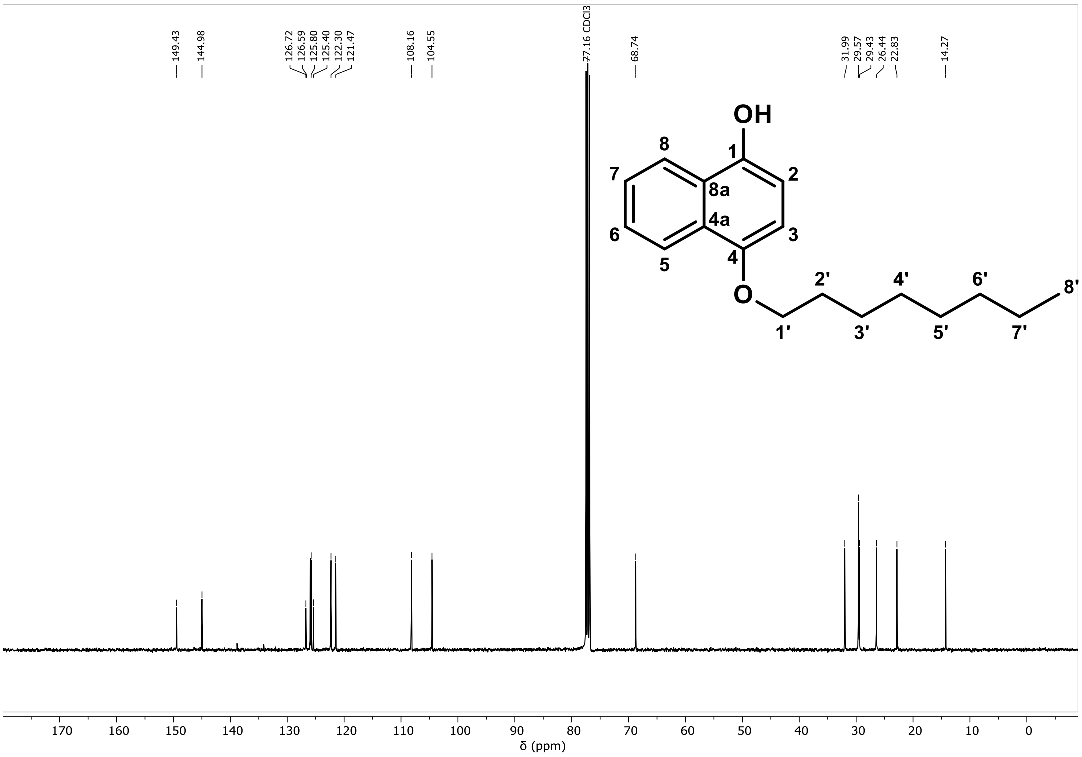

^{13}C NMR spectrum (101 MHz, CDCl$_3$) of **41**.

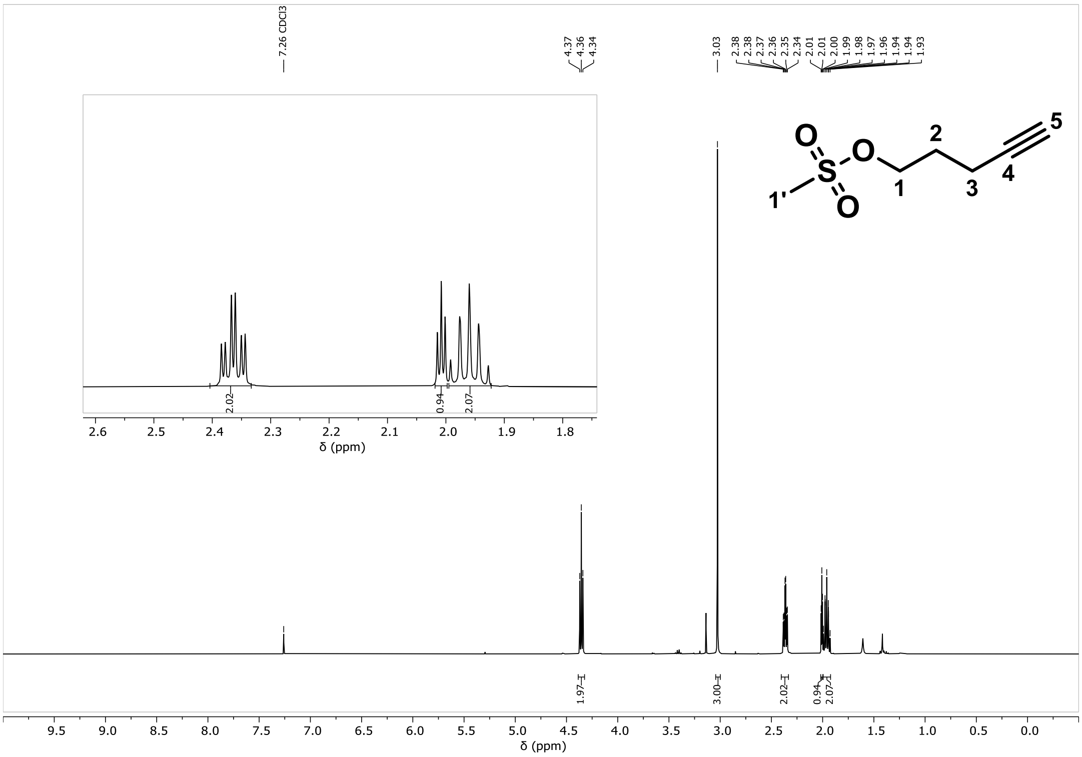

^{1}H NMR spectrum (400 MHz, $CDCl_3$) of **42**.

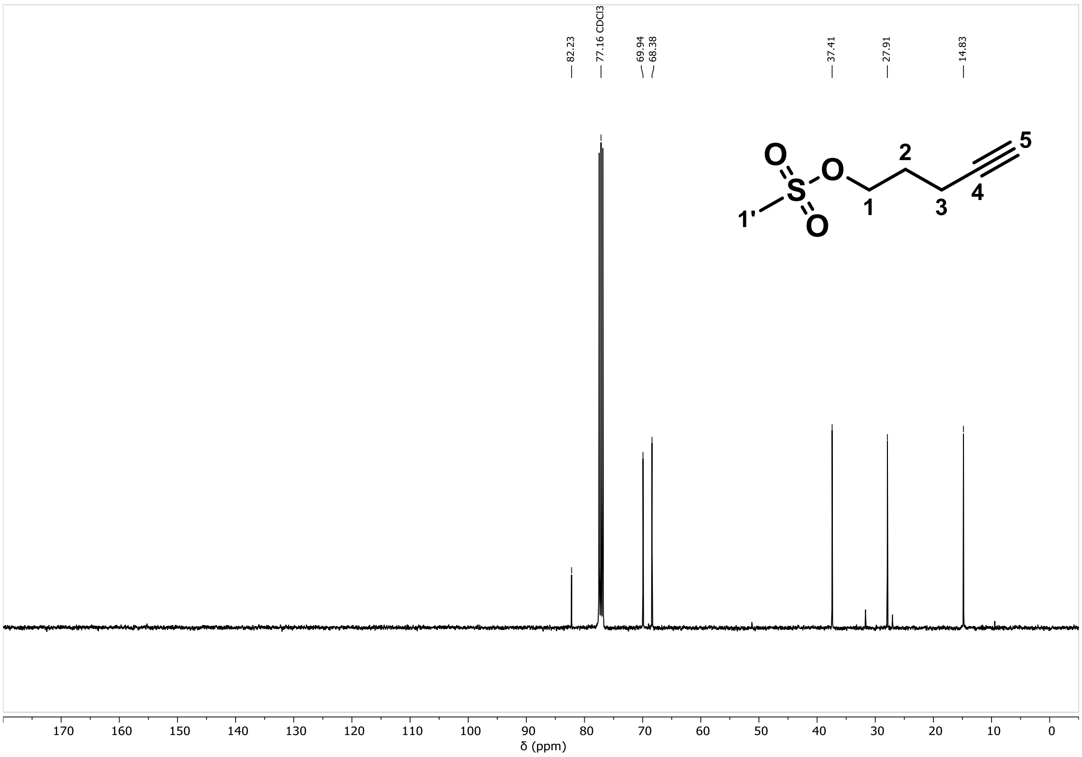

^{13}C NMR spectrum (101 MHz, $CDCl_3$) of **42**.

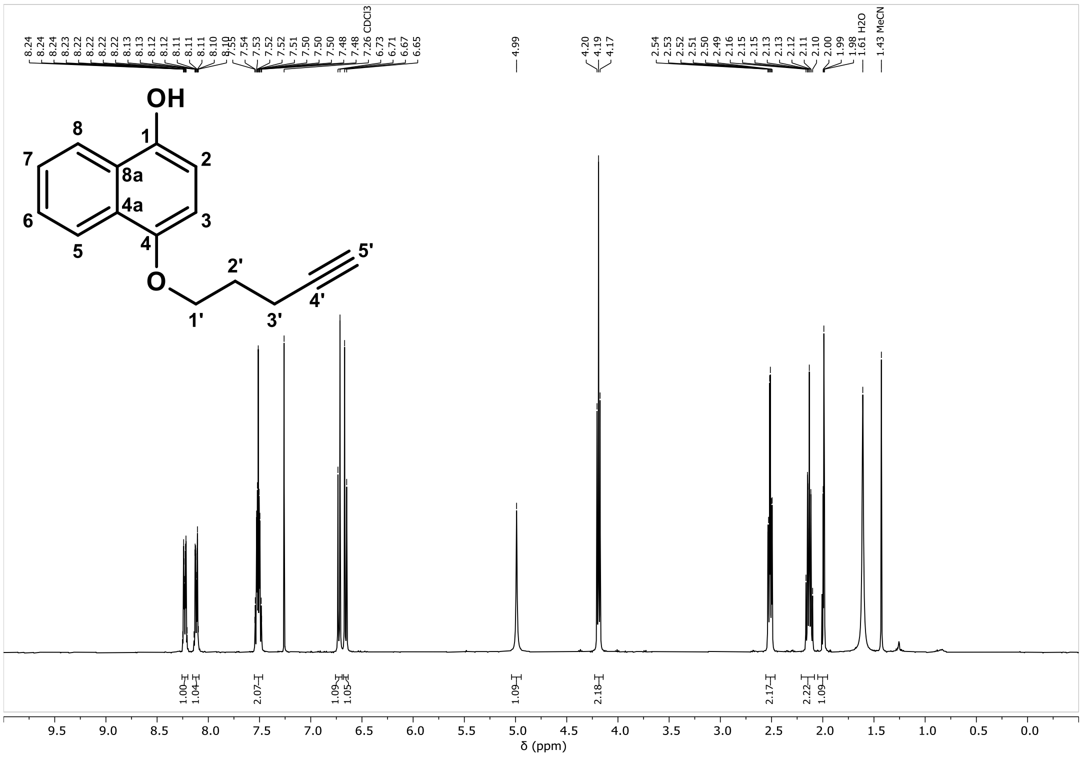

^{1}H NMR spectrum (400 MHz, $CDCl_3$) of **43**.

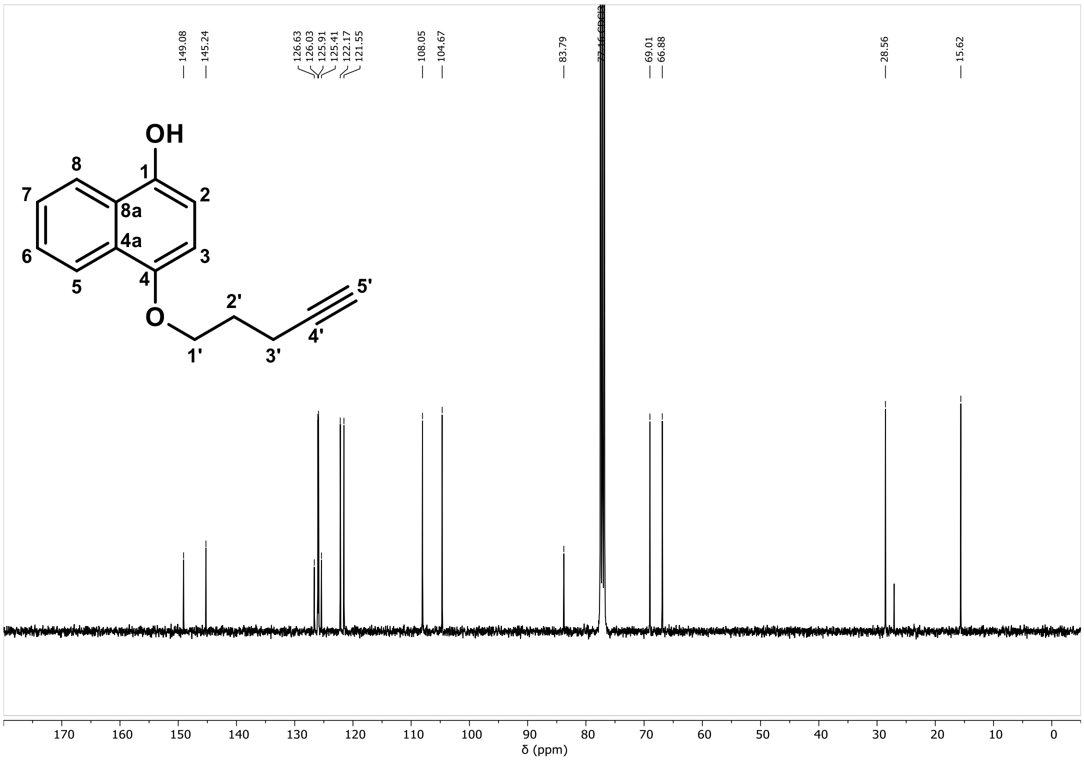

^{13}C NMR spectrum (101 MHz, $CDCl_3$) of **43**.

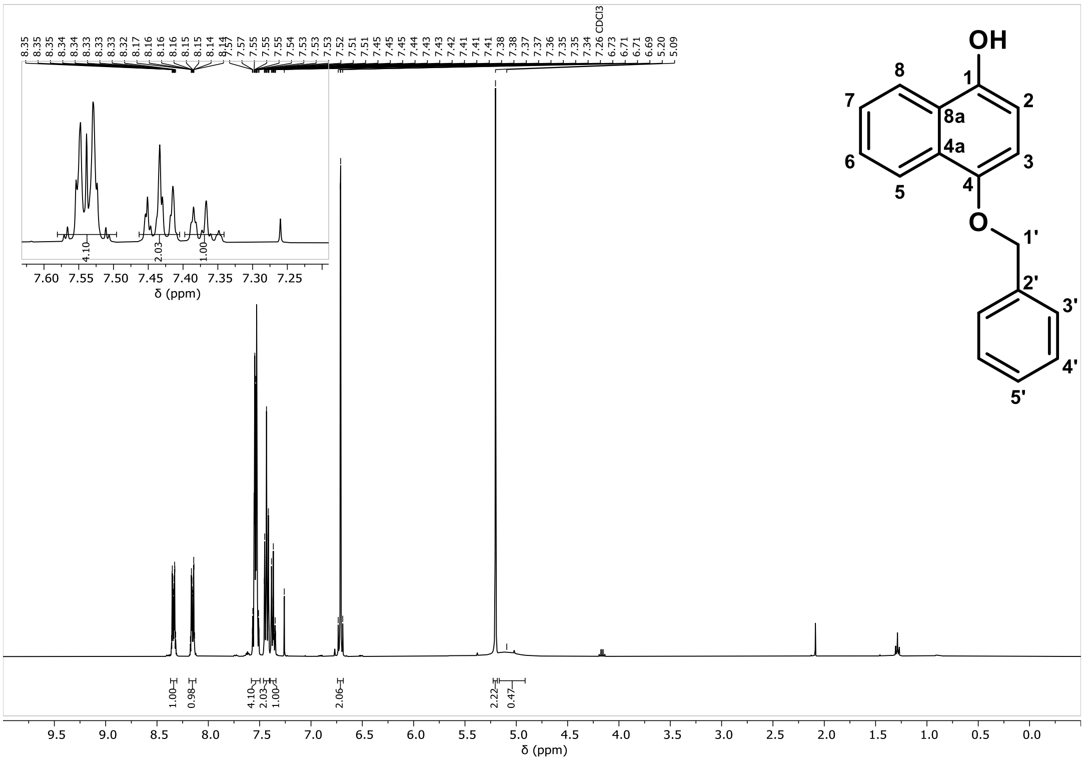

^{1}H NMR spectrum (400 MHz, $CDCl_3$) of **44**.

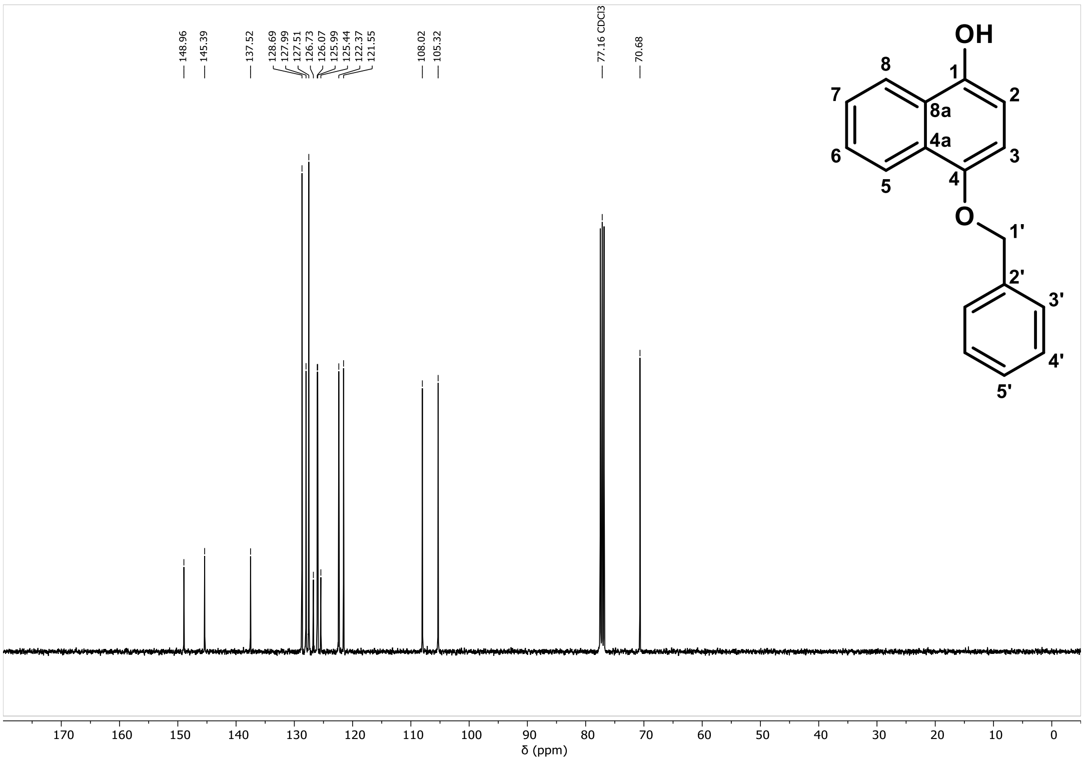

^{13}C NMR spectrum (101 MHz, $CDCl_3$) of **44**.

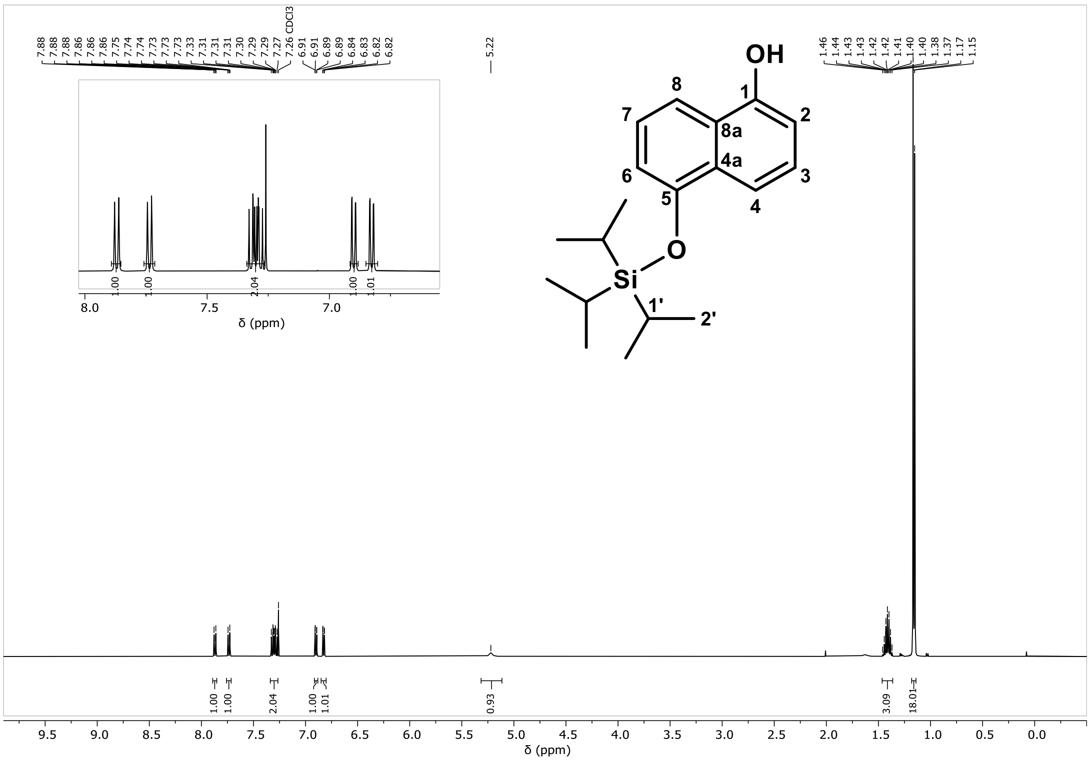

^{1}H NMR spectrum (600 MHz, $CDCl_3$) of **15**.

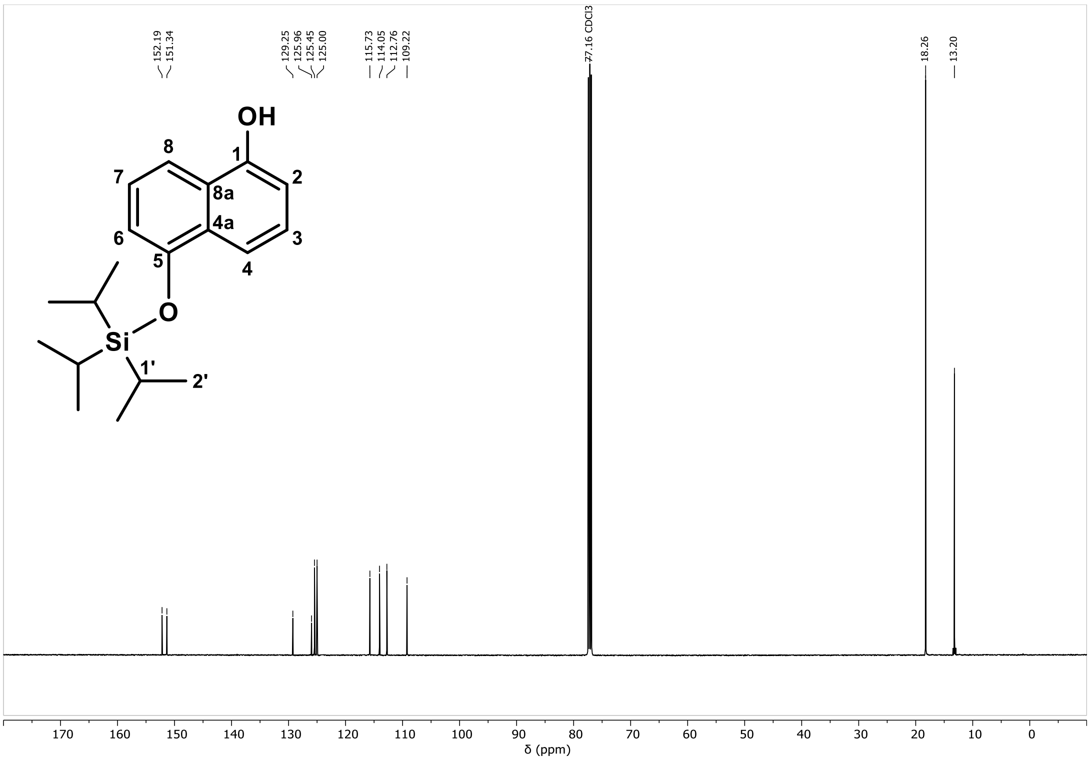

^{13}C NMR spectrum (126 MHz, $CDCl_3$) of **15**.

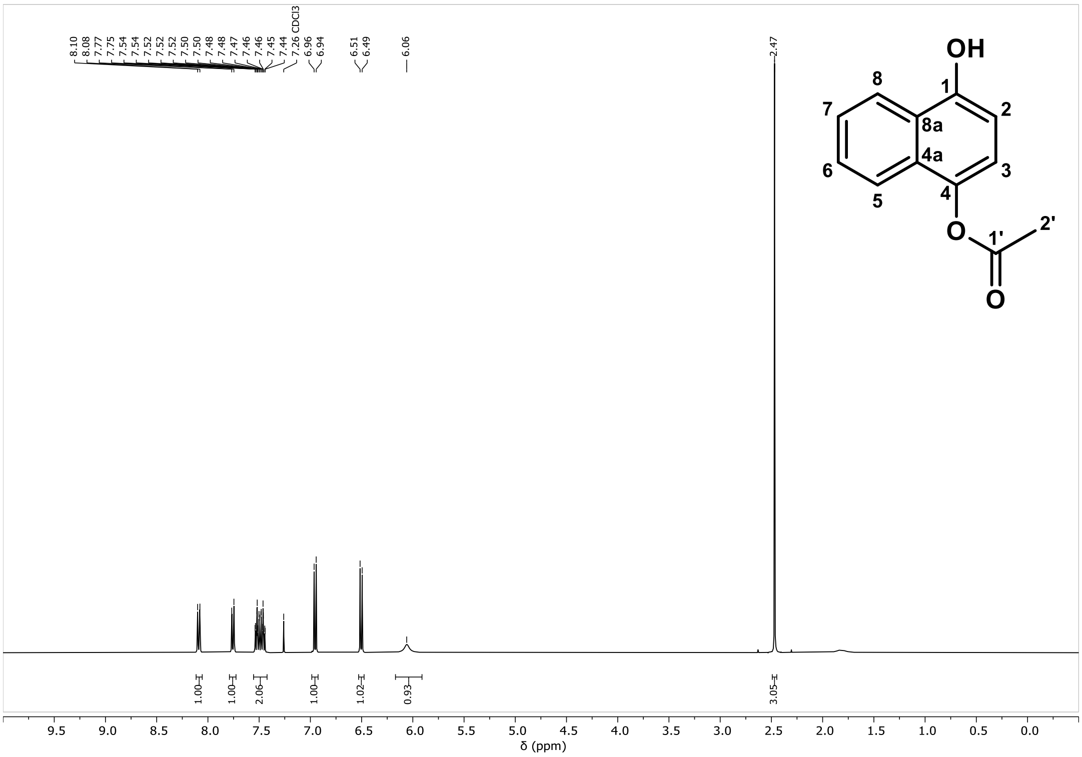

^{1}H NMR spectrum (400 MHz, $CDCl_3$) of **16**.

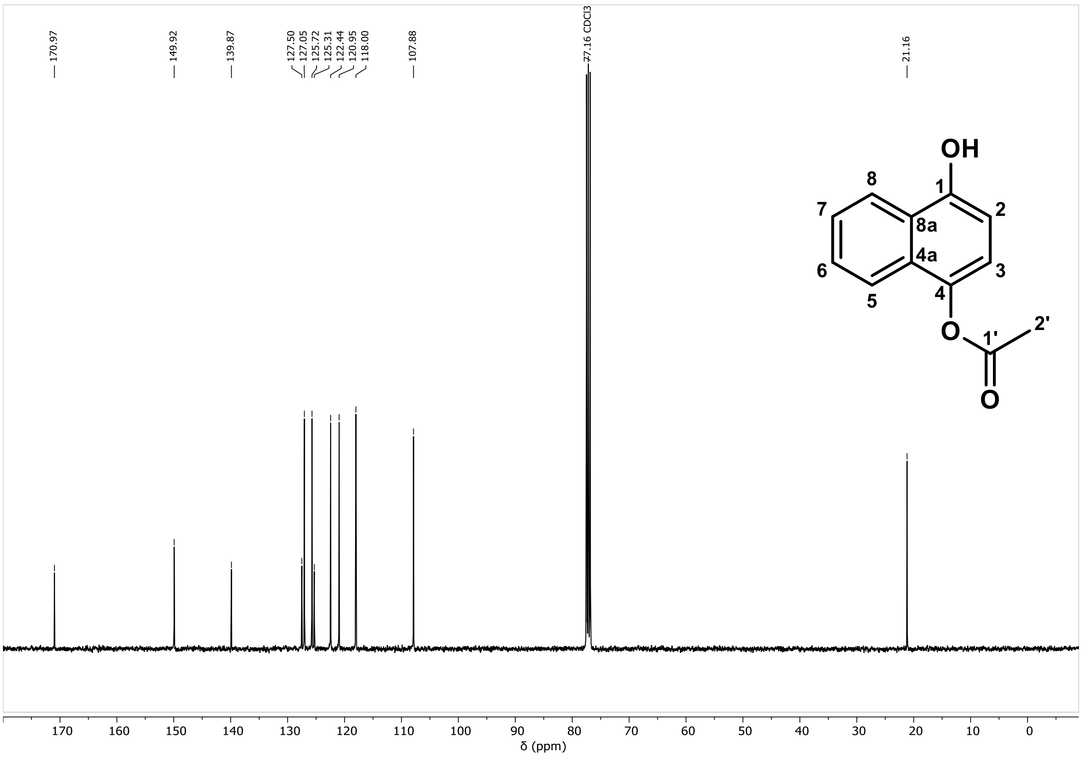

^{13}C NMR spectrum (101 MHz, $CDCl_3$) of **16**.

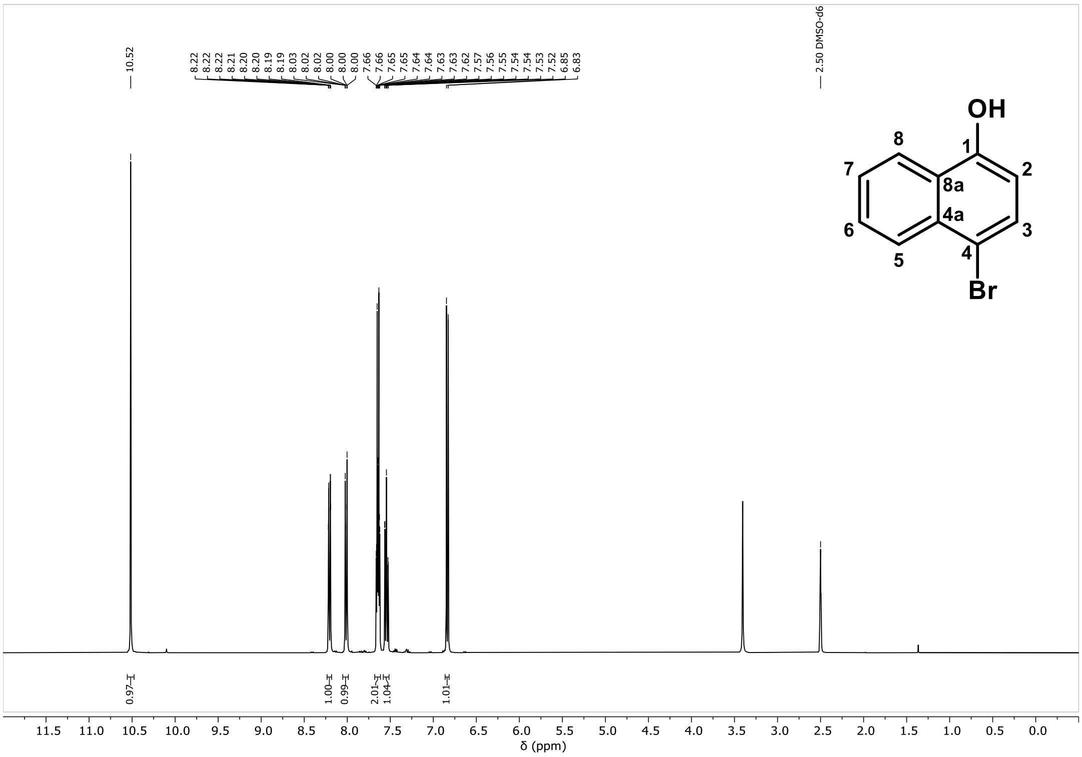

^{1}H NMR spectrum (400 MHz, DMSO-d_6) of **17**.

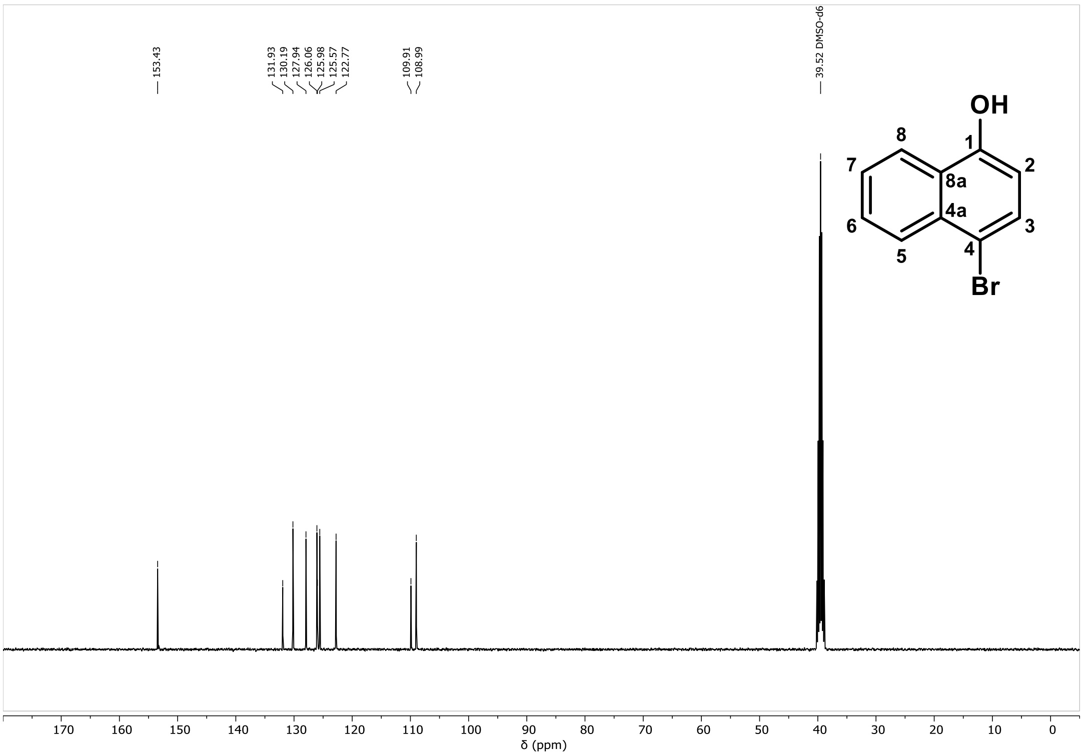

^{13}C NMR spectrum (101 MHz, DMSO-d_6) of **17**.

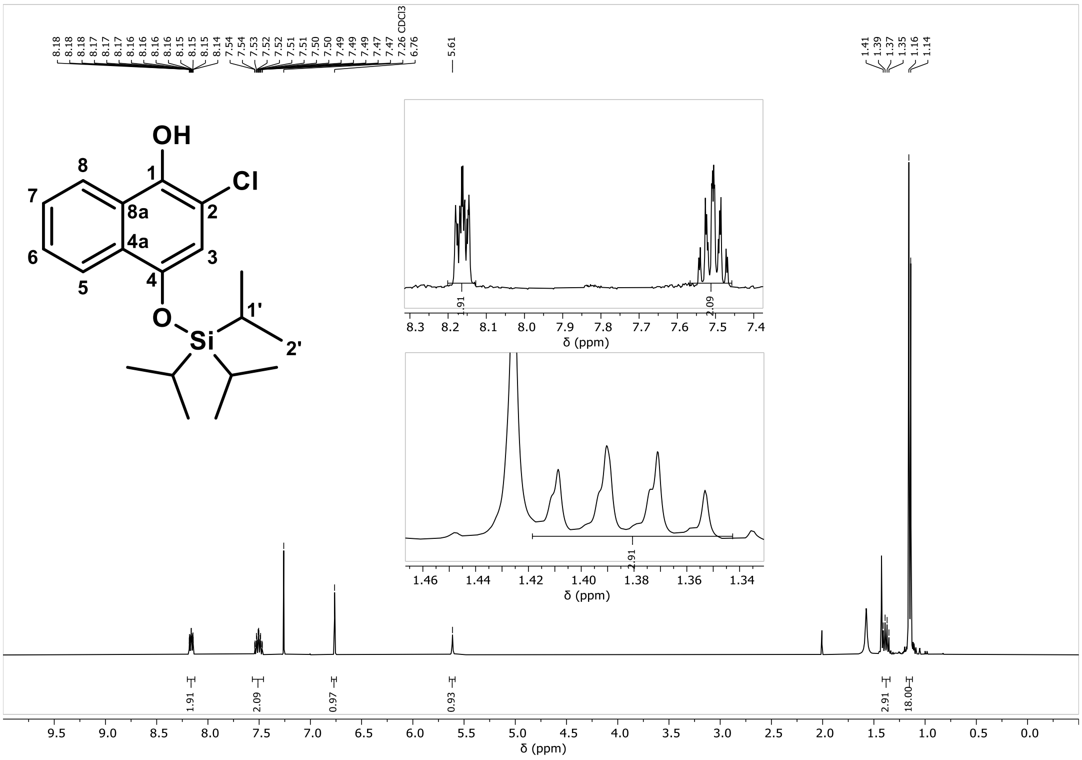

^{1}H NMR spectrum (400 MHz, $CDCl_3$) of **47**.

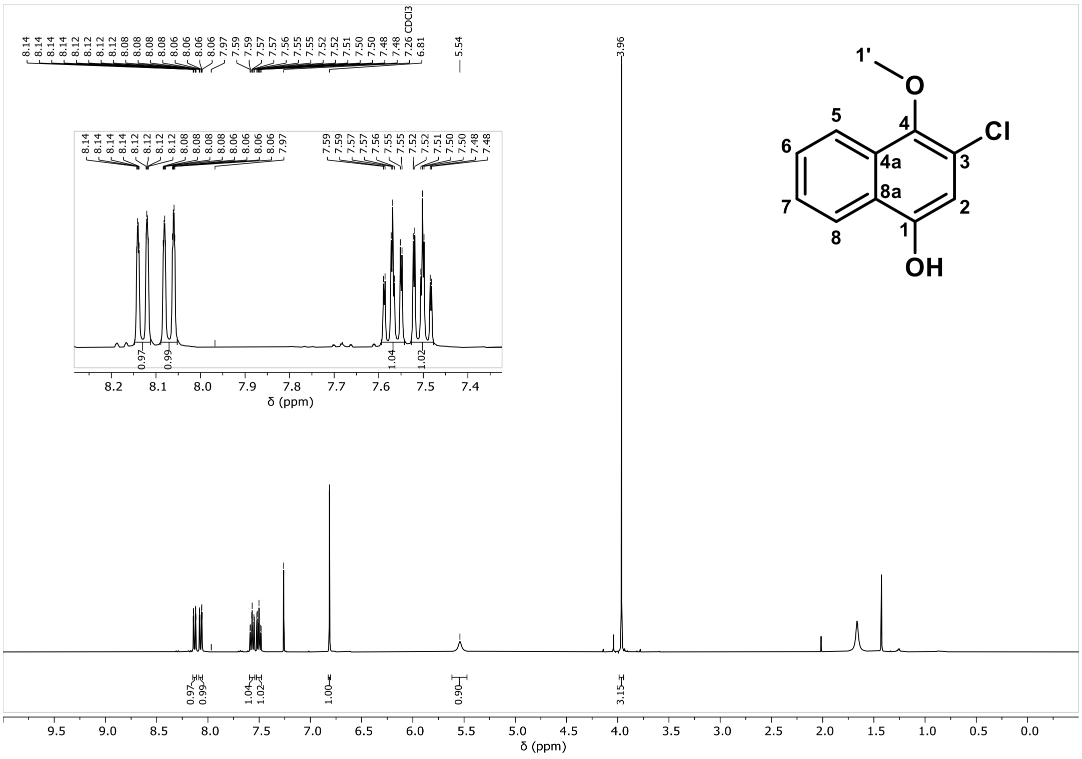

^{1}H NMR spectrum (400 MHz, $CDCl_3$) of **18**.

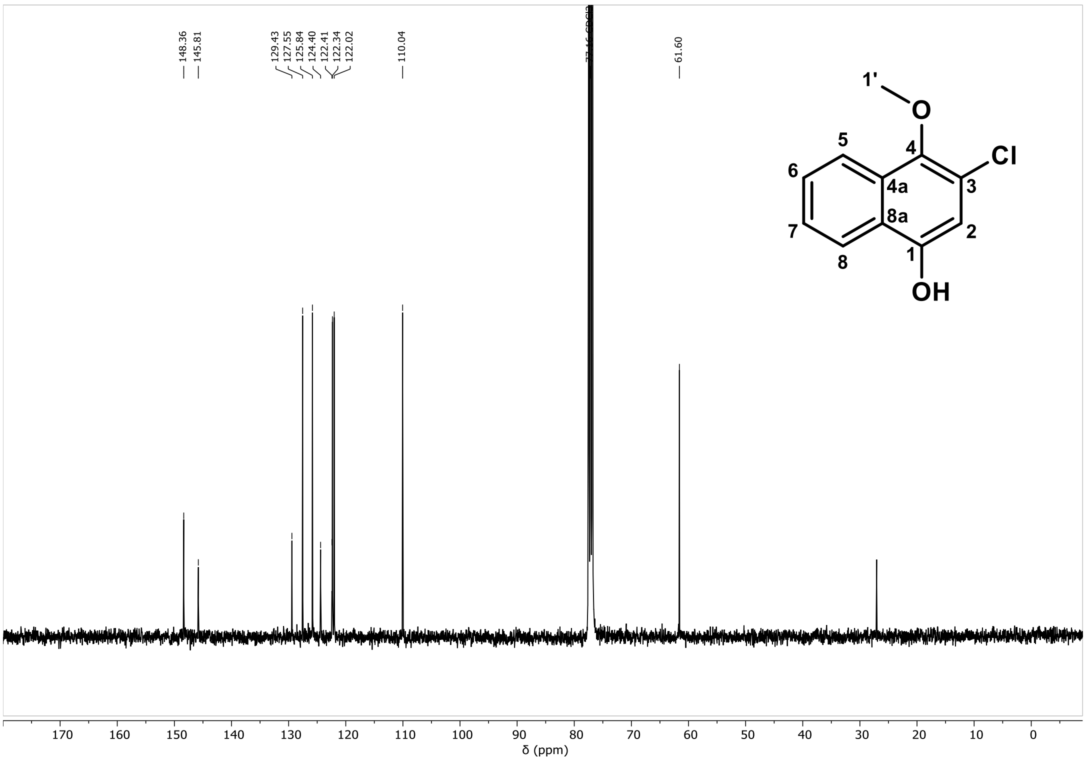

^{13}C NMR spectrum (101 MHz, $CDCl_3$) of **18**.

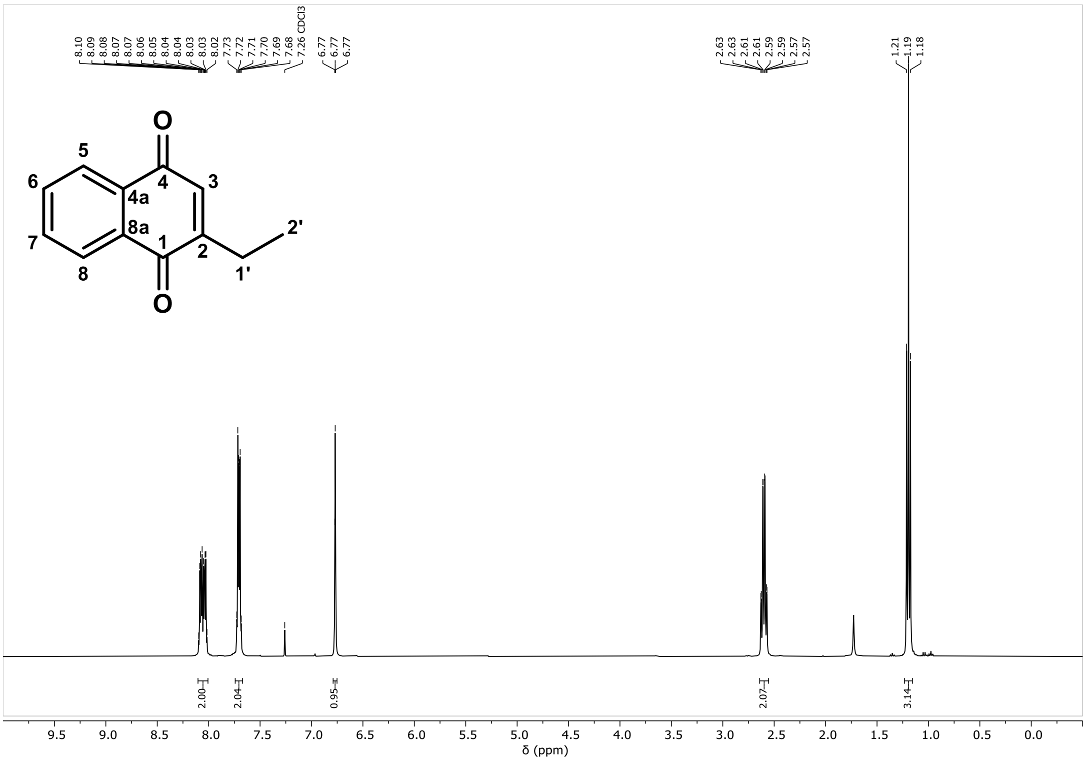

^{1}H NMR spectrum (400 MHz, $CDCl_3$) of **50**.

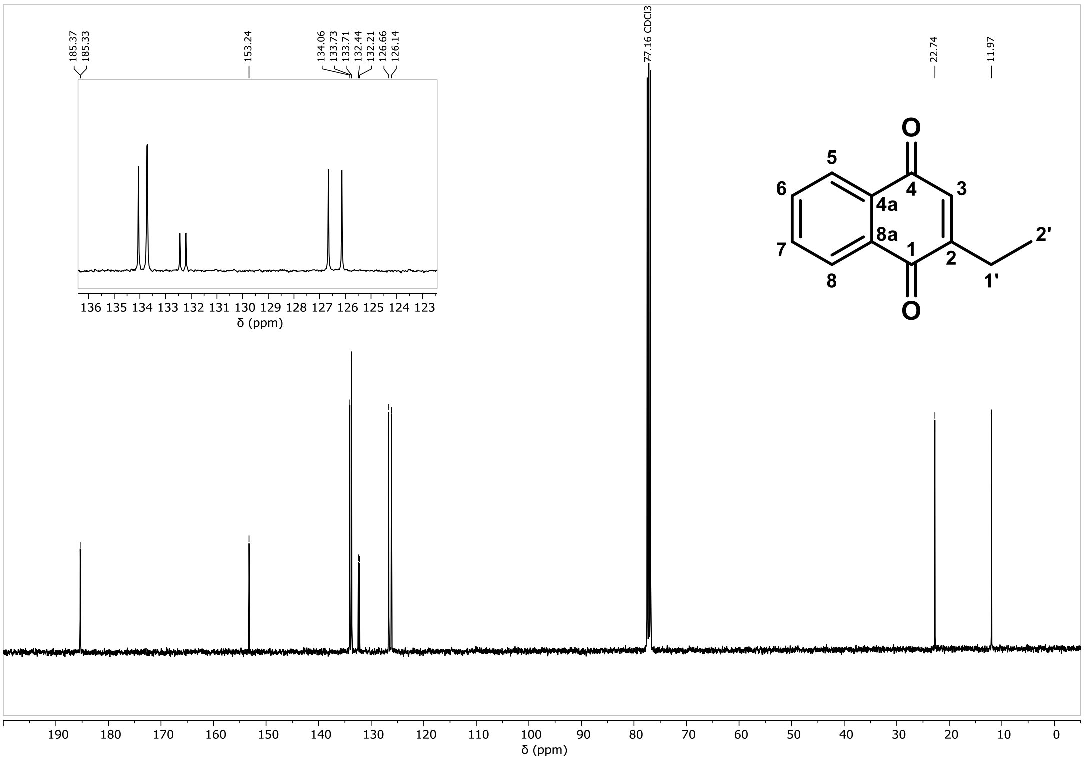

^{13}C NMR spectrum (101 MHz, $CDCl_3$) of **50**.

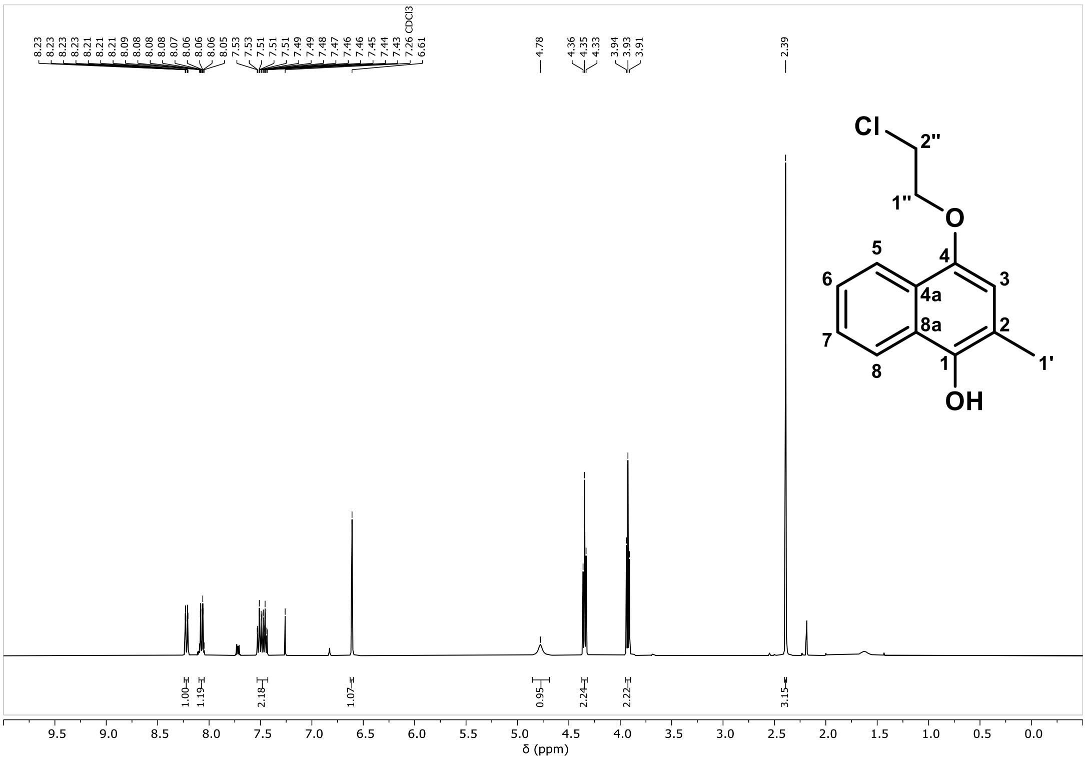

^{1}H NMR spectrum (400 MHz, $CDCl_3$) of **53**.

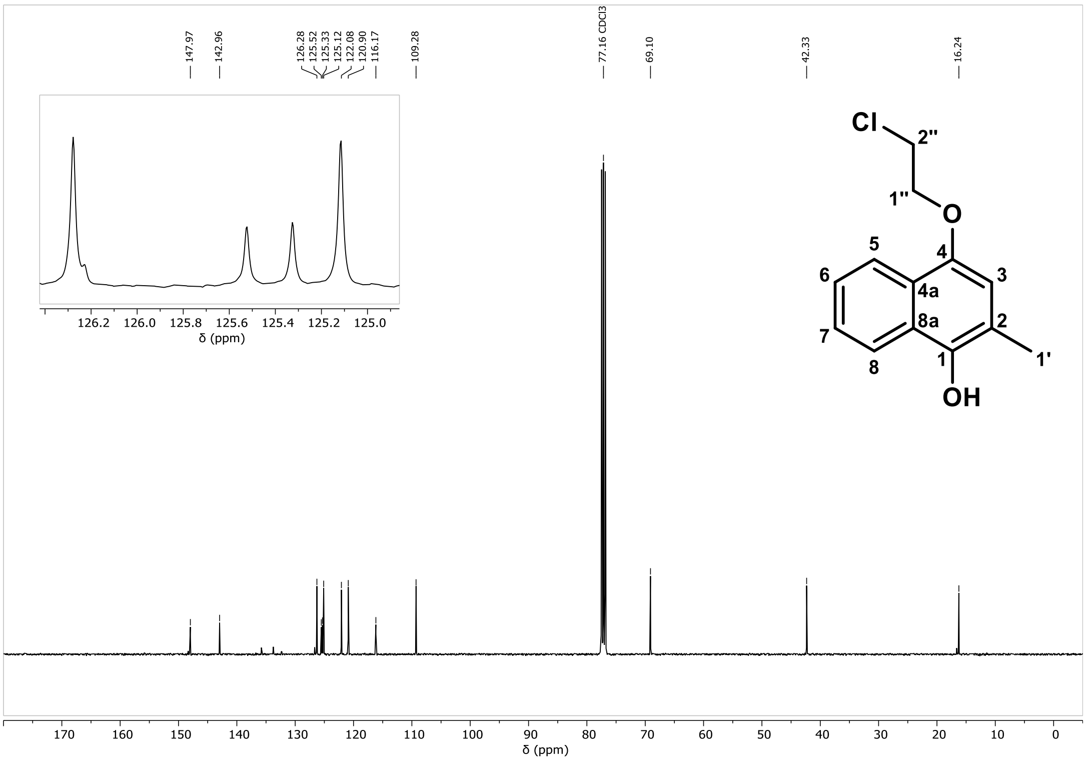

^{13}C NMR spectrum (101 MHz, $CDCl_3$) of **53**.

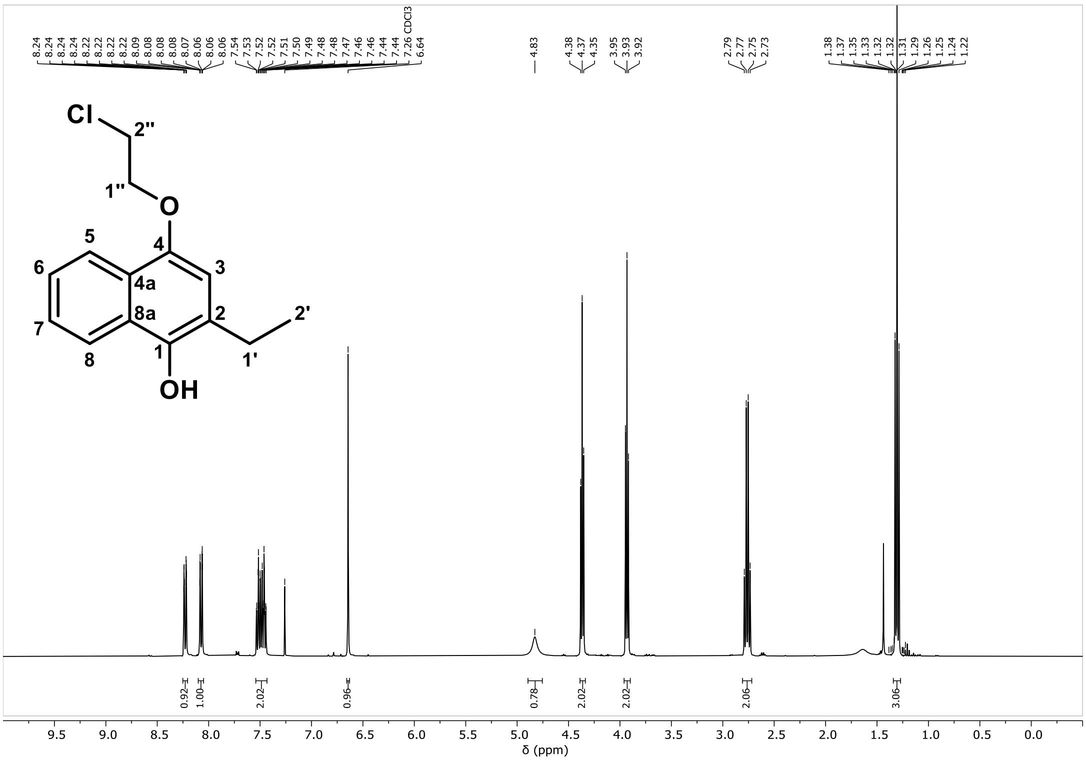

^{1}H NMR spectrum (400 MHz, $CDCl_3$) of **54**.

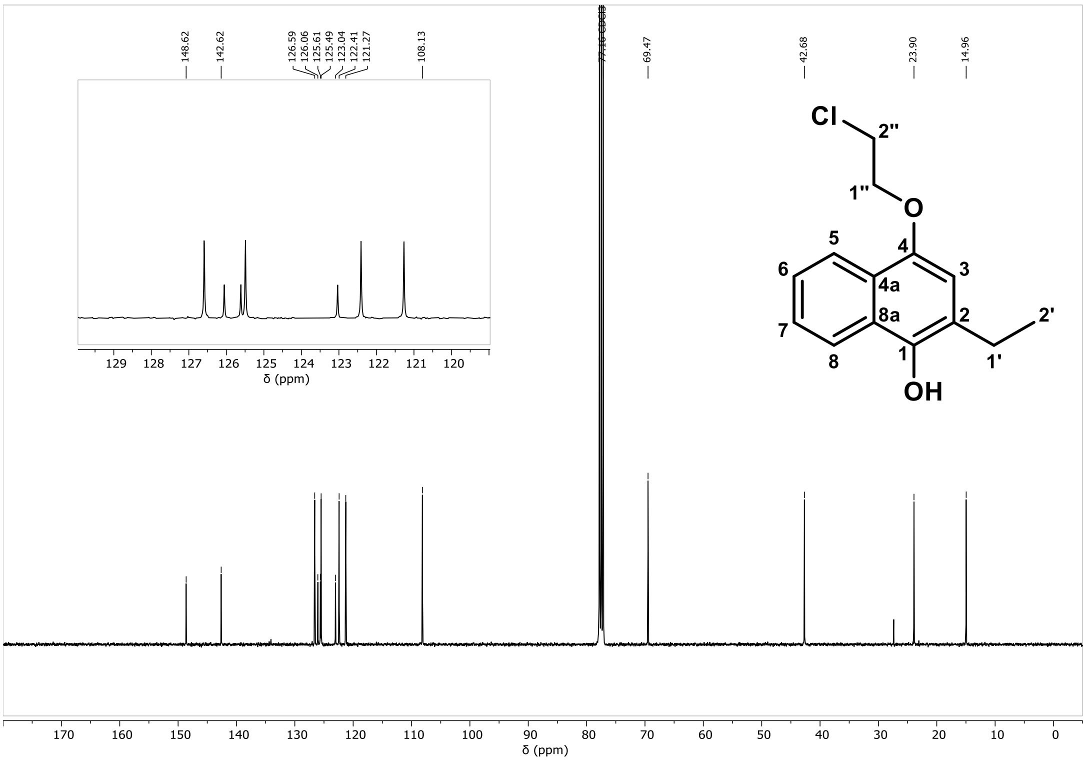

^{13}C NMR spectrum (101 MHz, $CDCl_3$) of **54**.

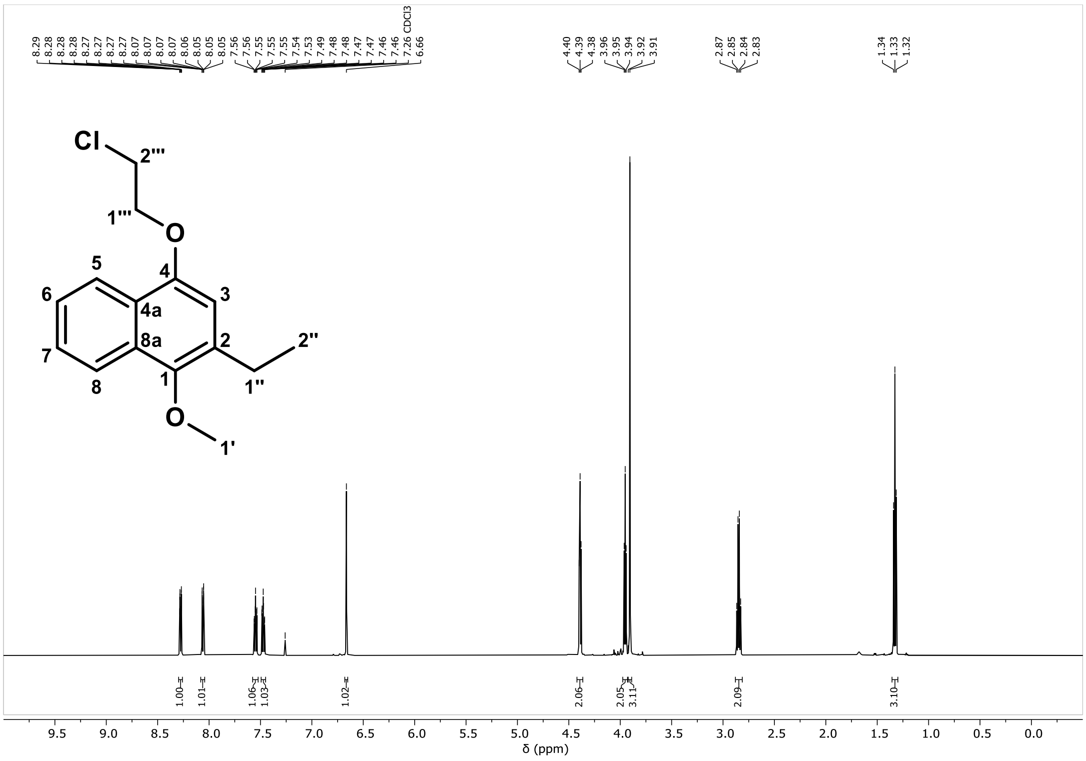

^{1}H NMR spectrum (400 MHz, $CDCl_3$) of **56**.

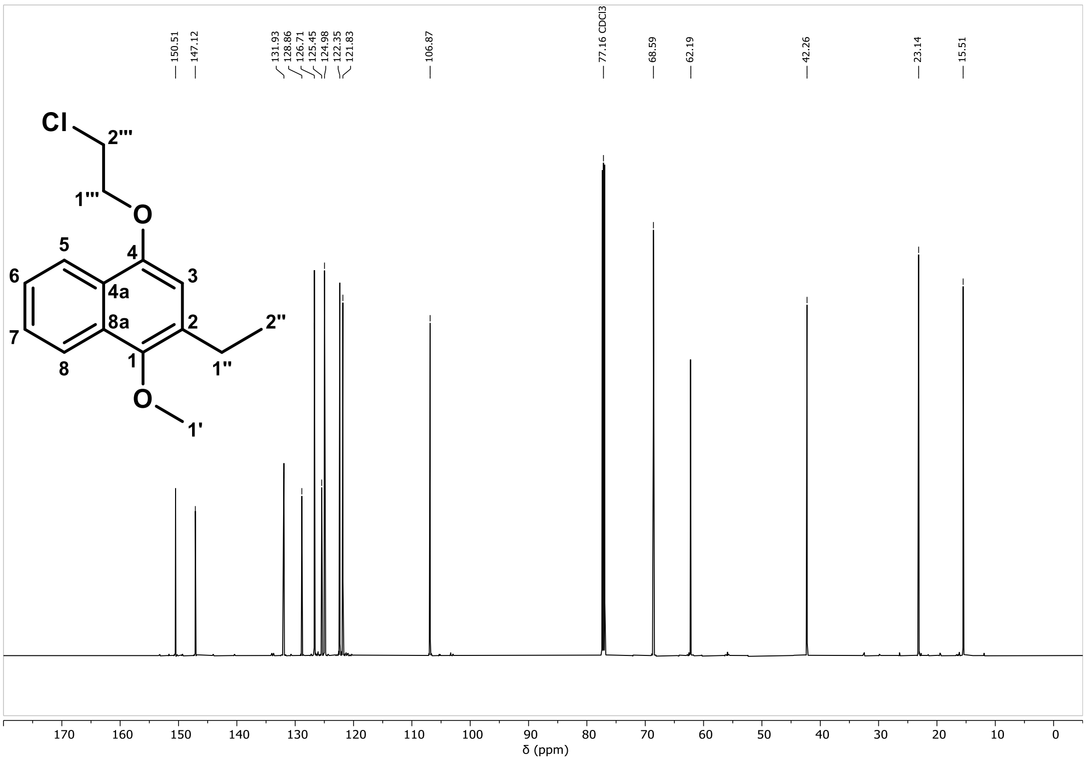

^{13}C NMR spectrum (101 MHz, $CDCl_3$) of **56**.

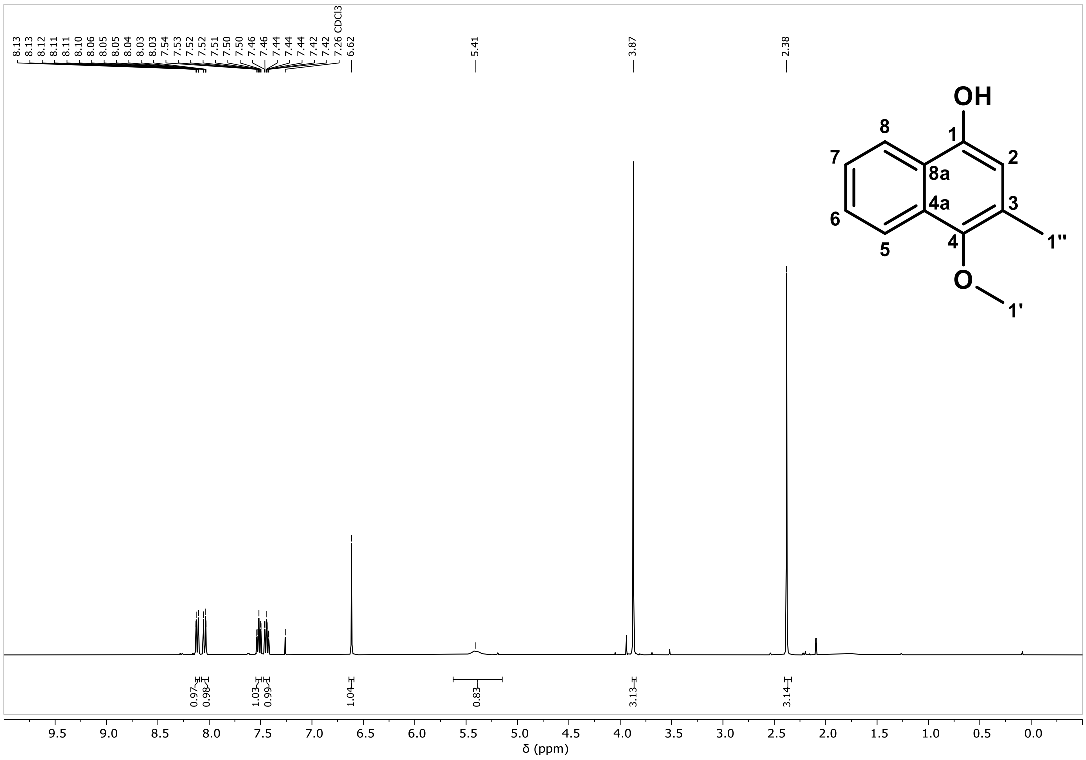

^{1}H NMR spectrum (400 MHz, $CDCl_3$) of **19**.

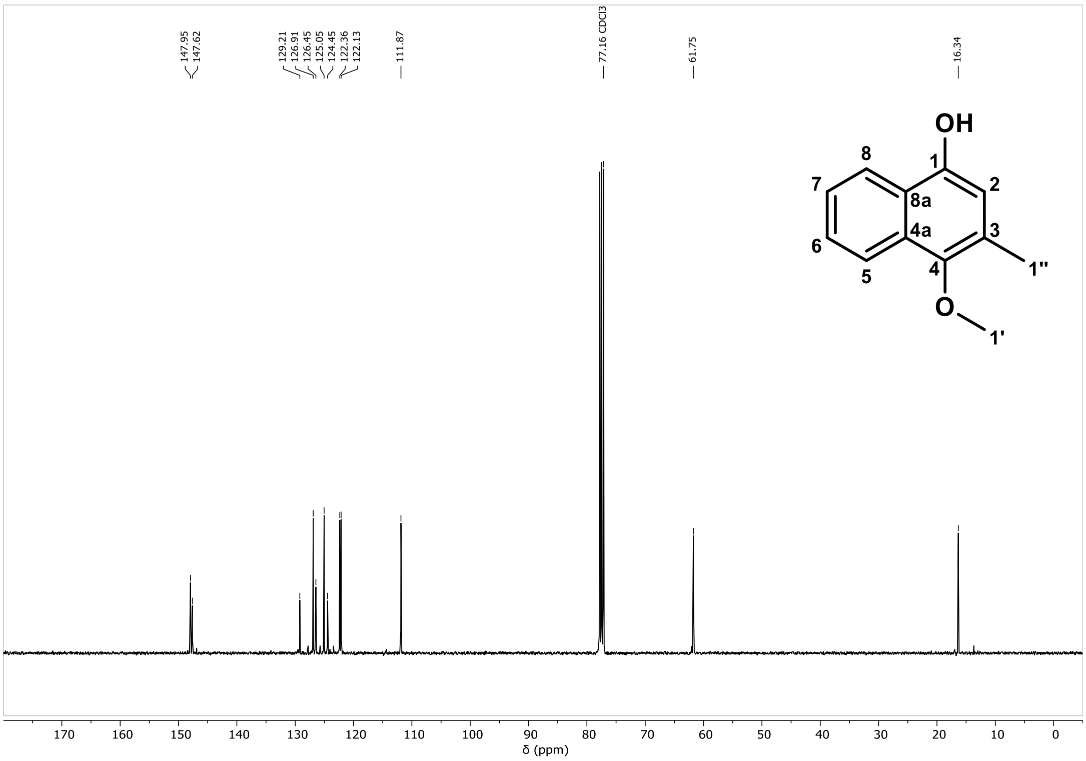

^{1}H NMR spectrum (400 MHz, $CDCl_3$) of **19**.

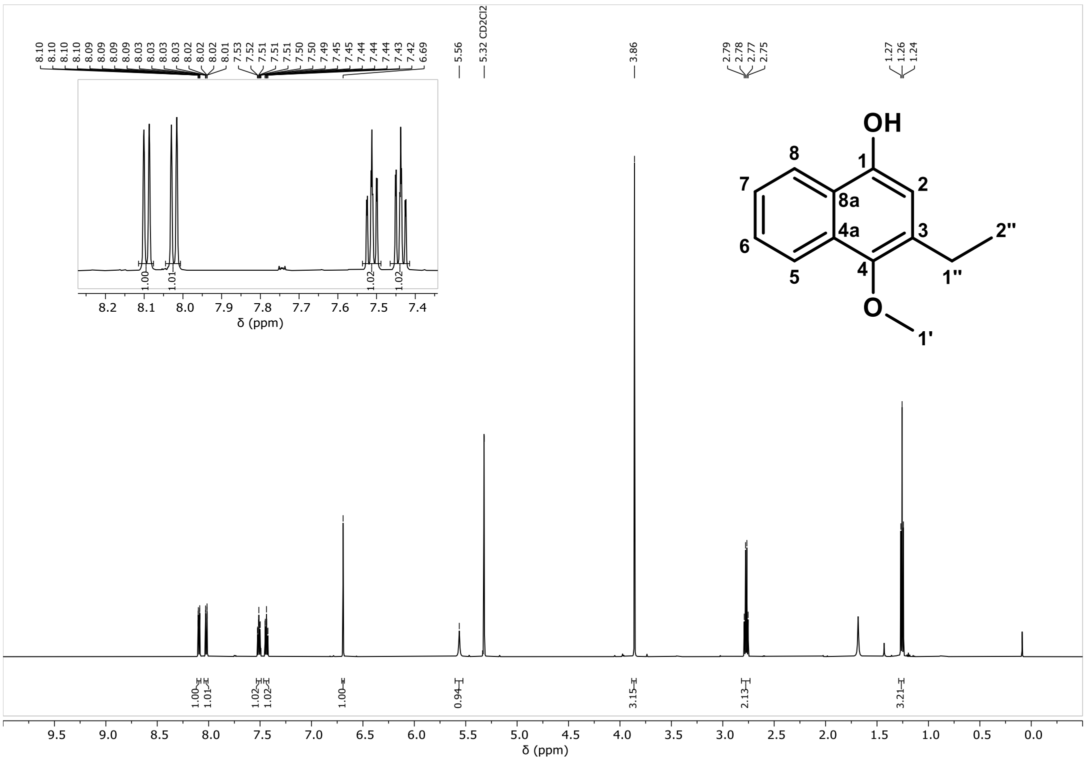

^{1}H NMR spectrum (400 MHz, CD_2Cl_2) of **20**.

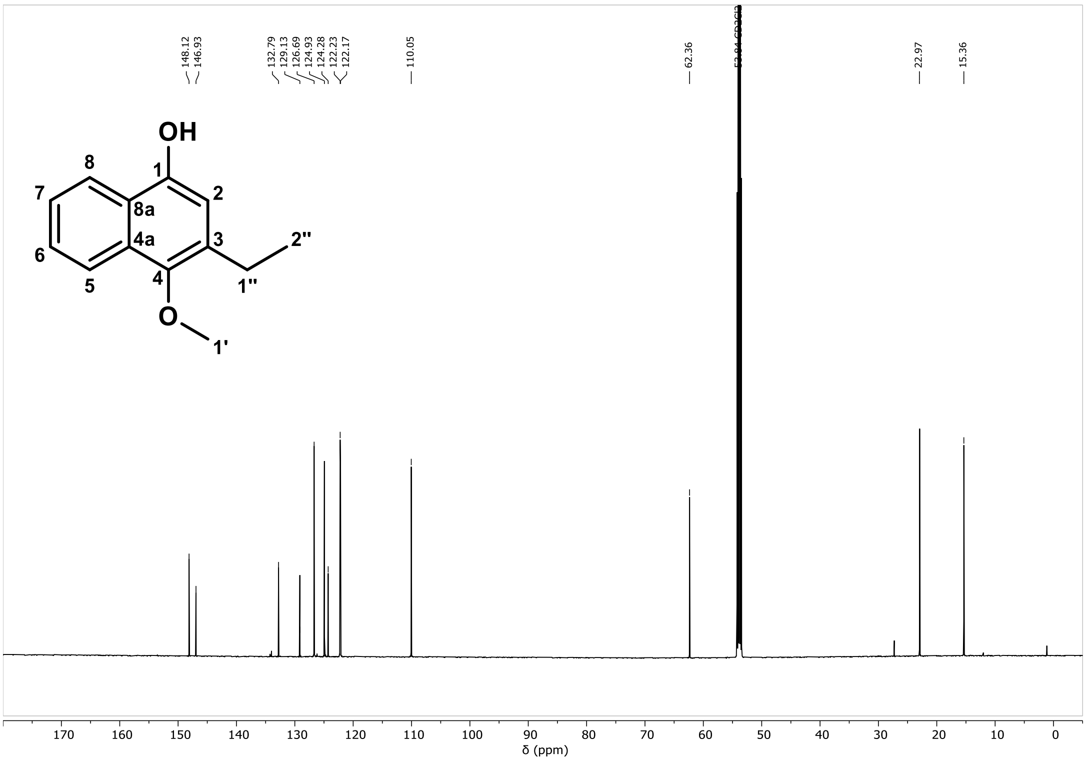

^{13}C NMR spectrum (151 MHz, CD_2Cl_2) of **20**.

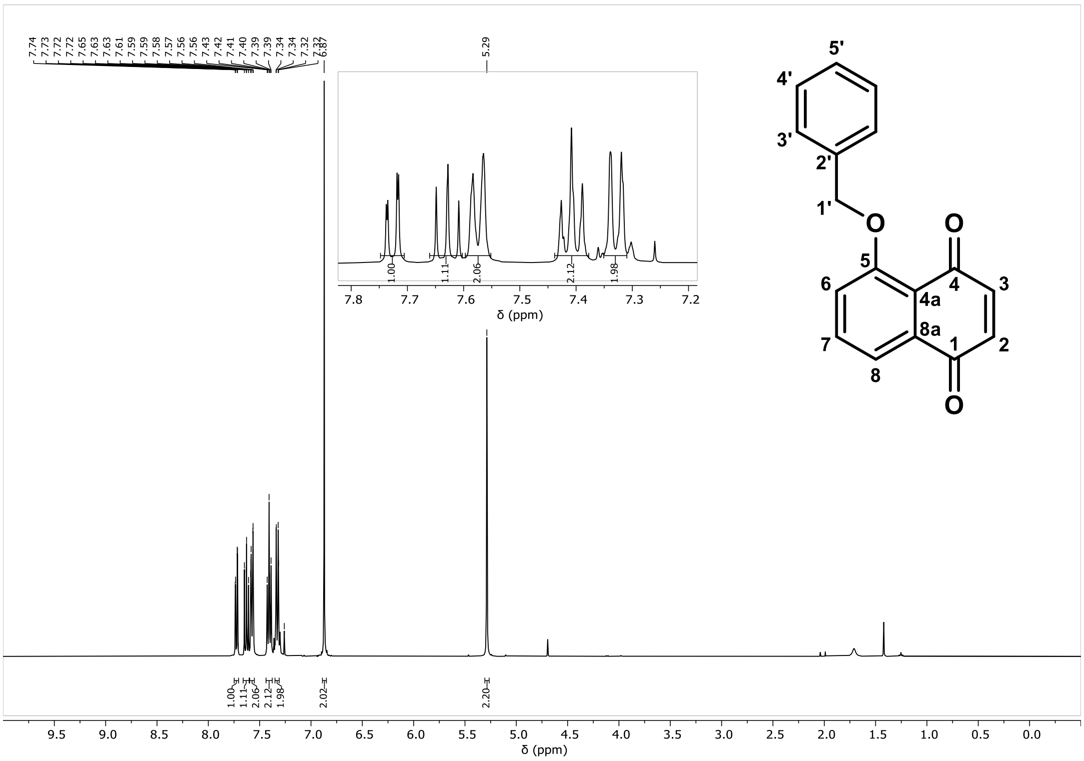

^{1}H NMR spectrum (400 MHz, $CDCl_3$) of **58**.

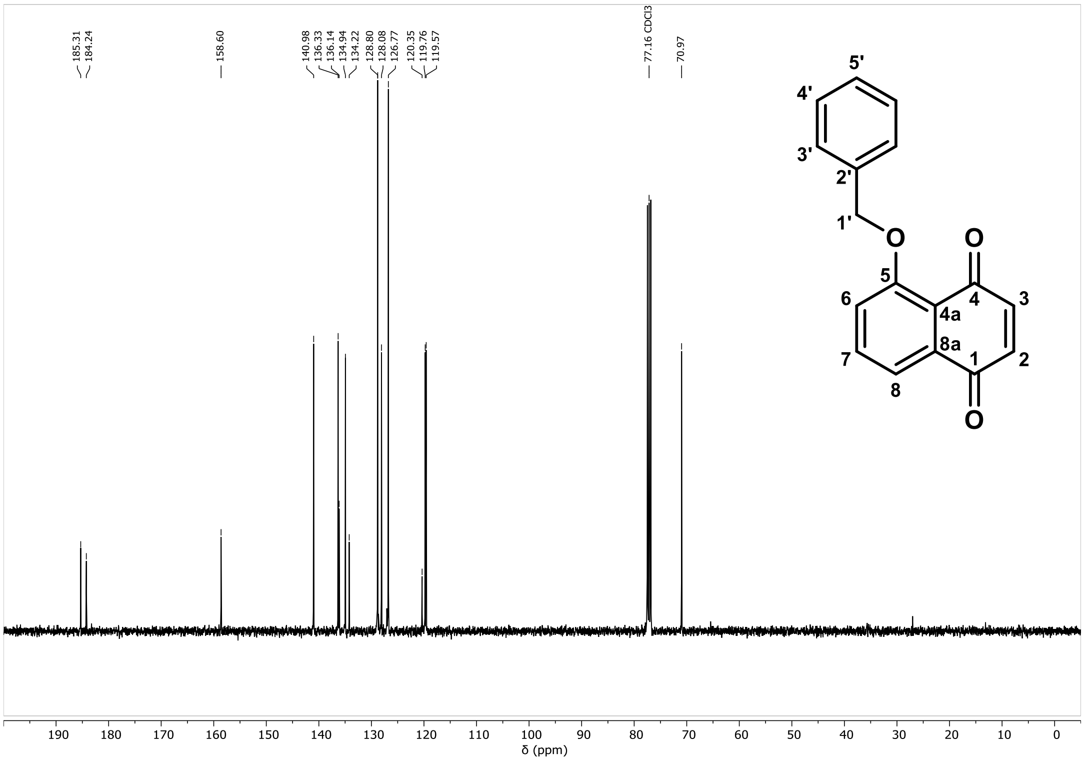

^{13}C NMR spectrum (151 MHz, $CDCl_3$) of **58**.

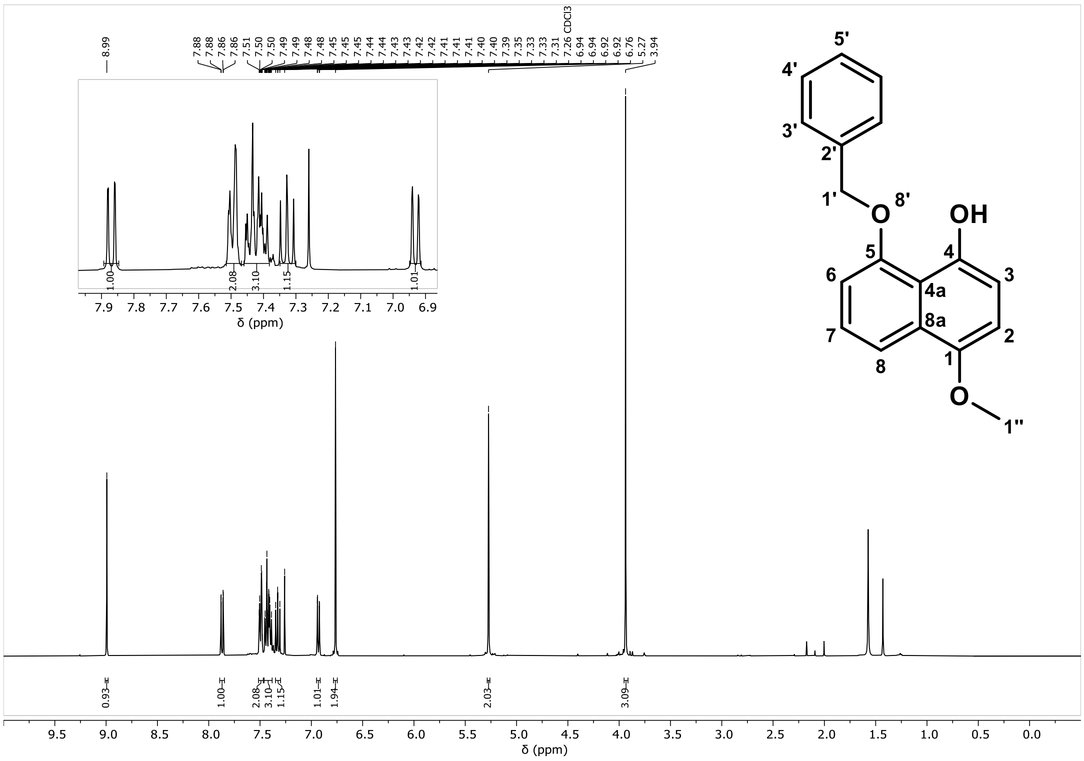

^{1}H NMR spectrum (400 MHz, $CDCl_3$) of **21**.

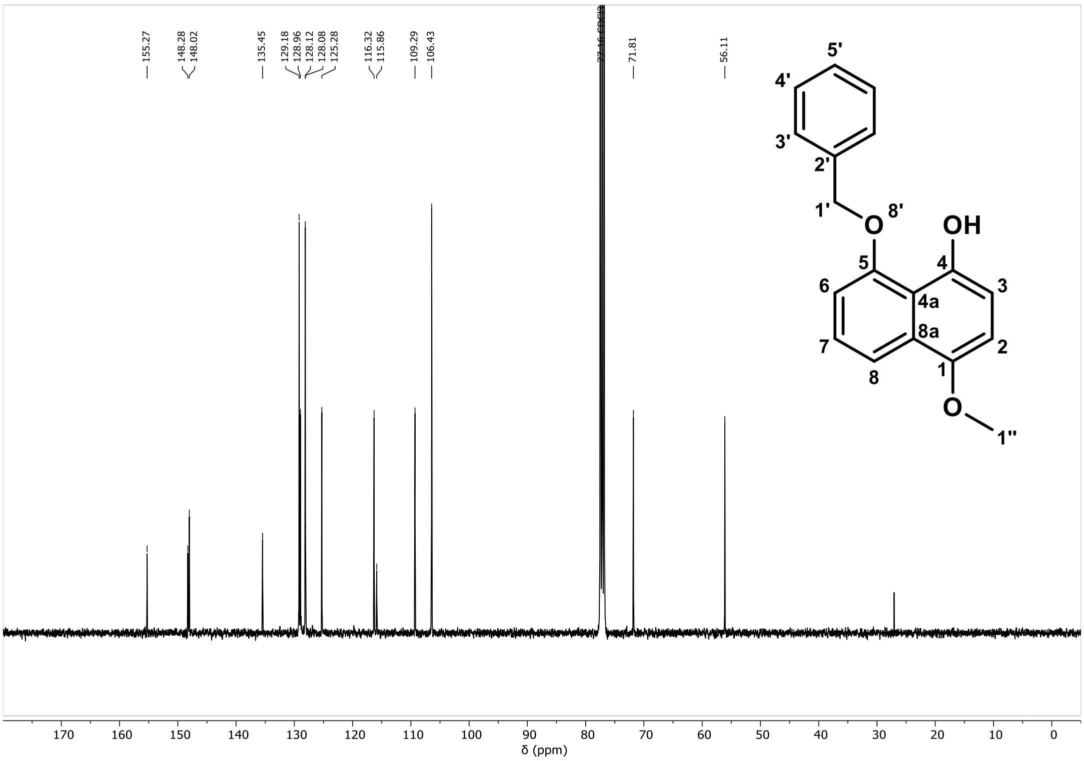

^{13}C NMR spectrum (151 MHz, $CDCl_3$) of **21**.

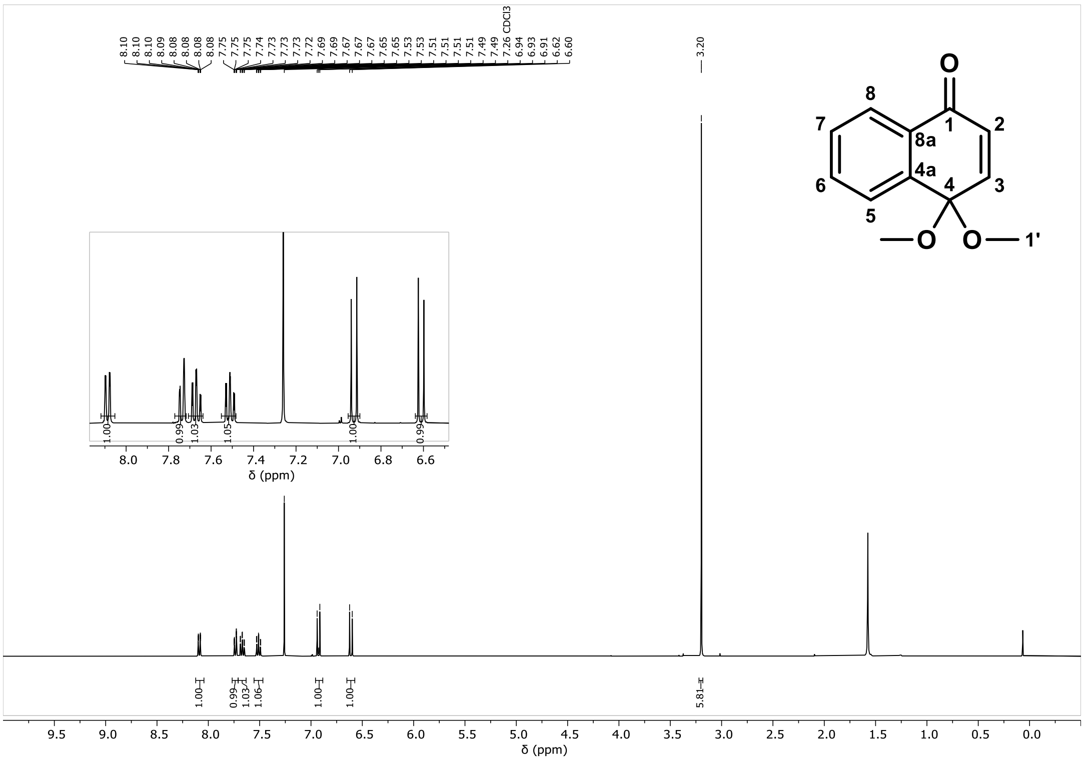

^{1}H NMR spectrum (400 MHz, $CDCl_3$) of **33**.

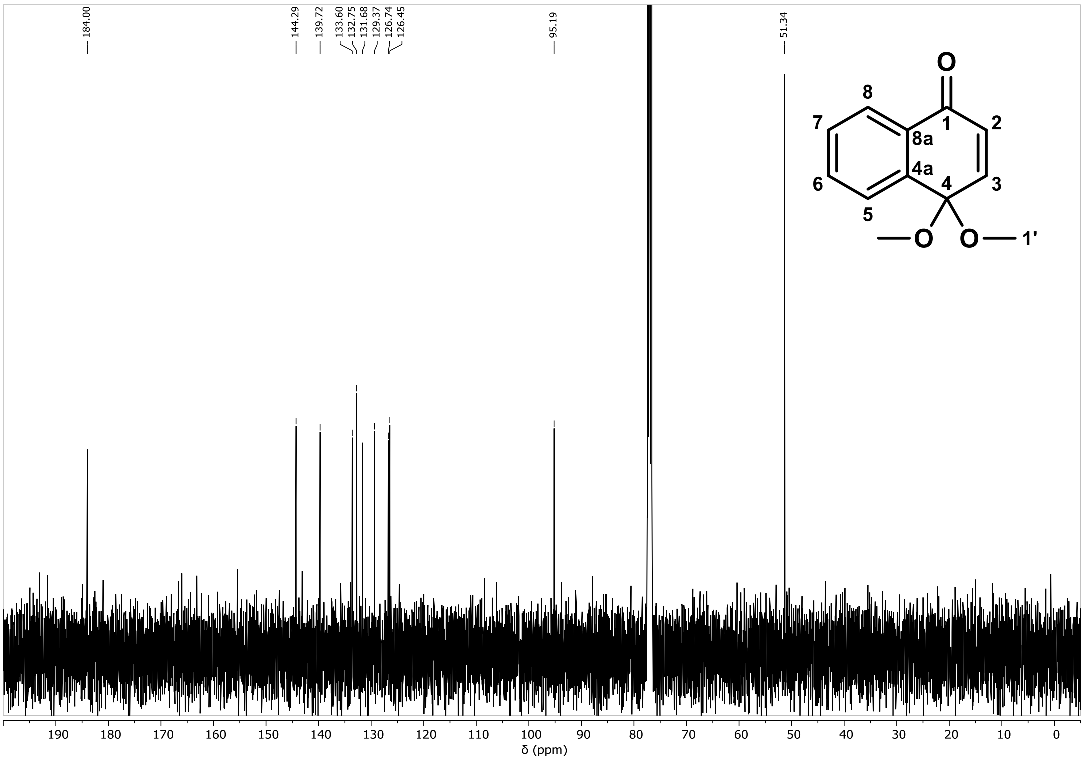

^{13}C NMR spectrum (151 MHz, $CDCl_3$) of **33**.

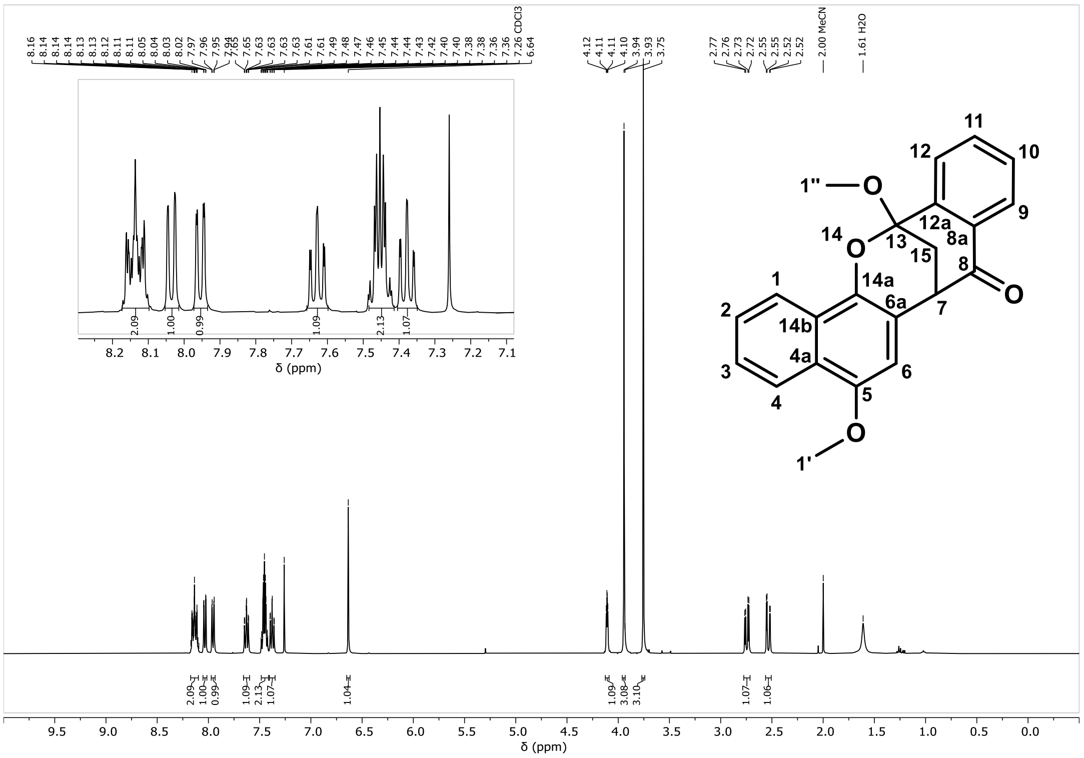

^{1}H NMR spectrum (400 MHz, $CDCl_3$) of **2**.

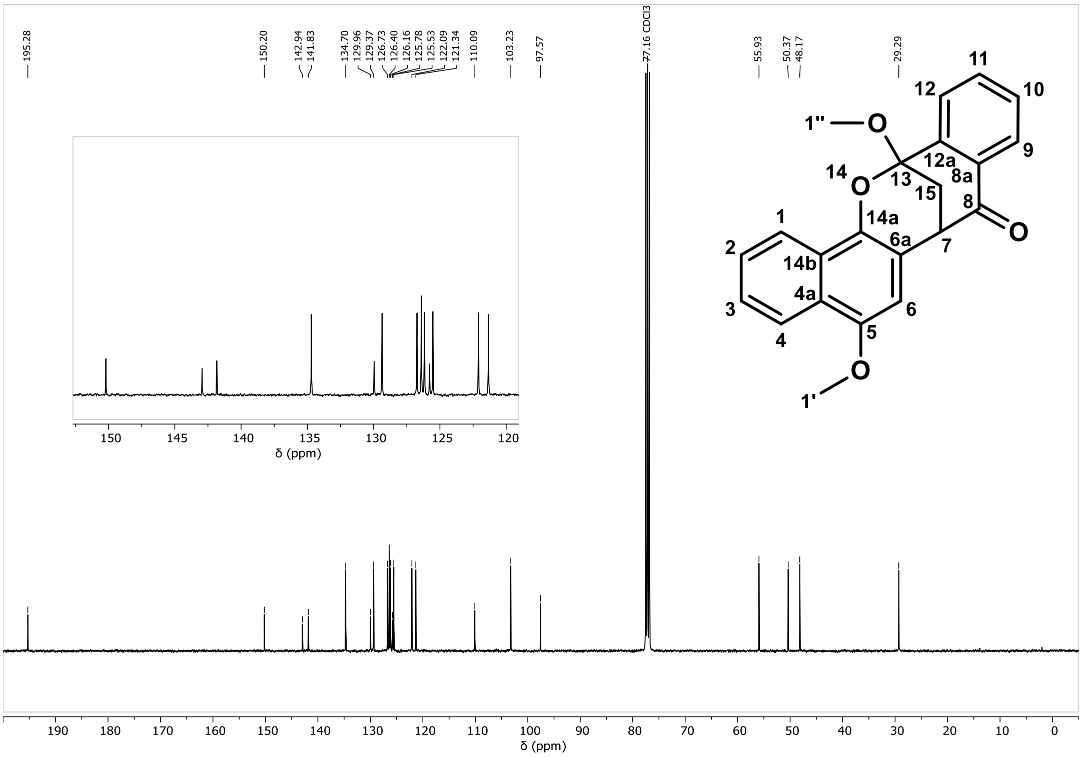

^{13}C NMR spectrum (101 MHz, $CDCl_3$) of **2**.

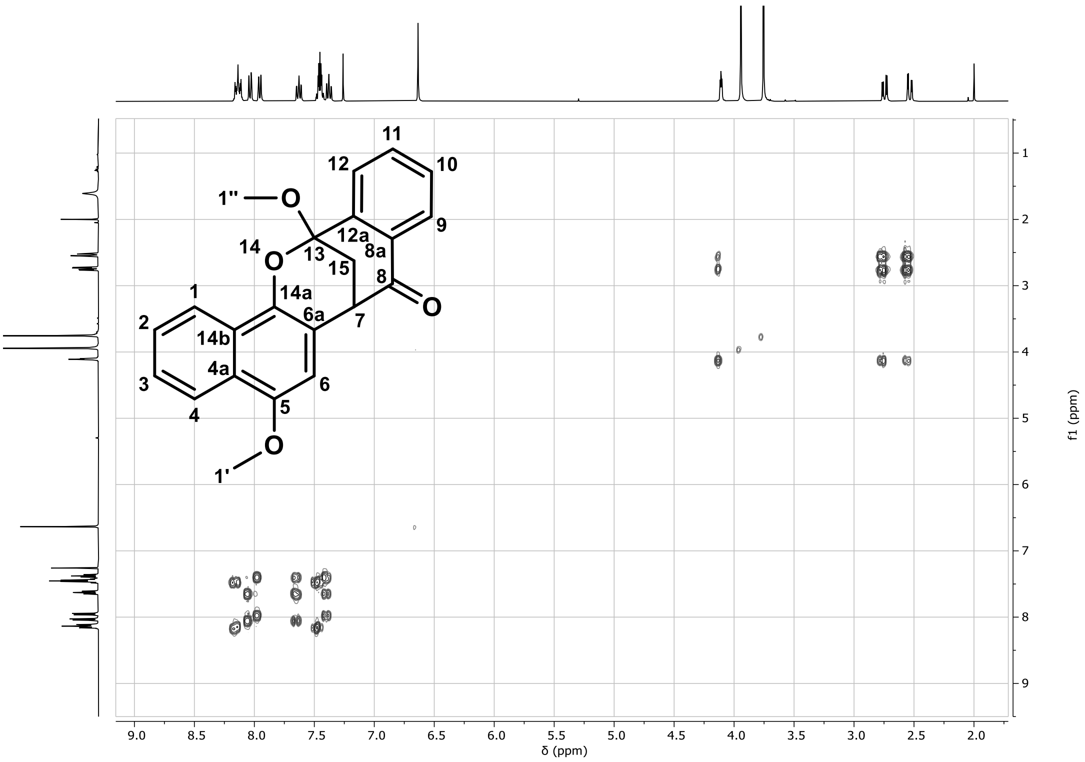

^{1}H,^{1}H COSY NMR ($CDCl_3$) of **2**.

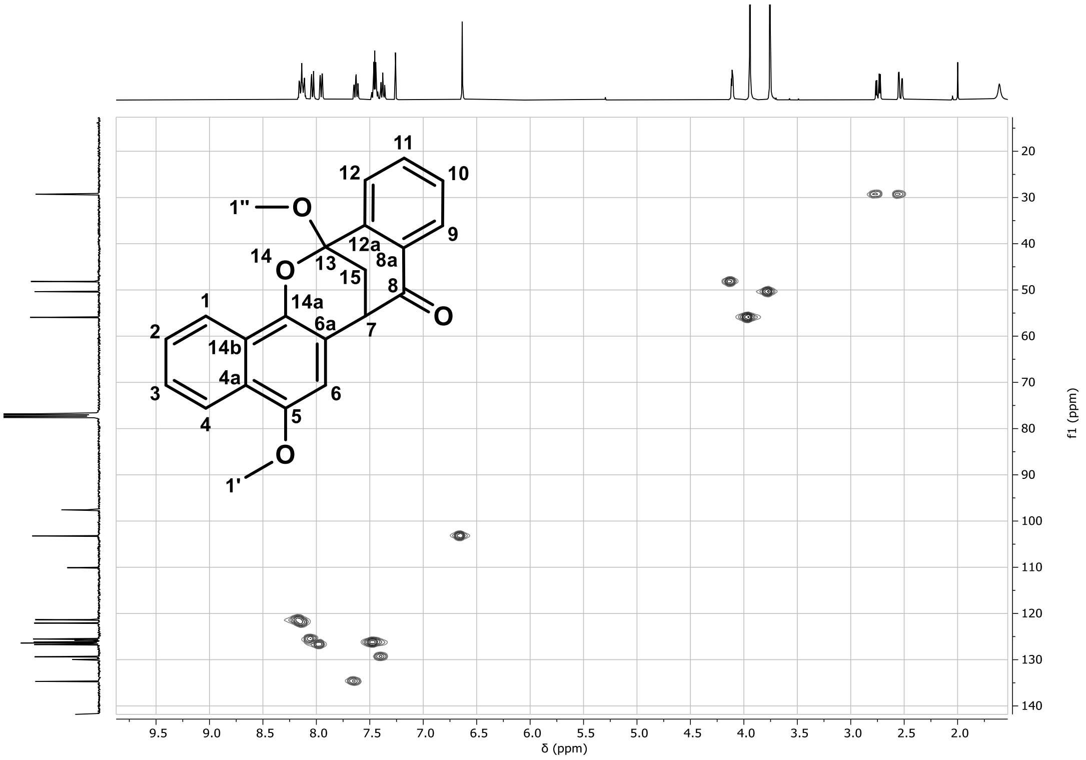

$^{1}H,^{13}C$ HSQC NMR ($CDCl_3$) of **2**.

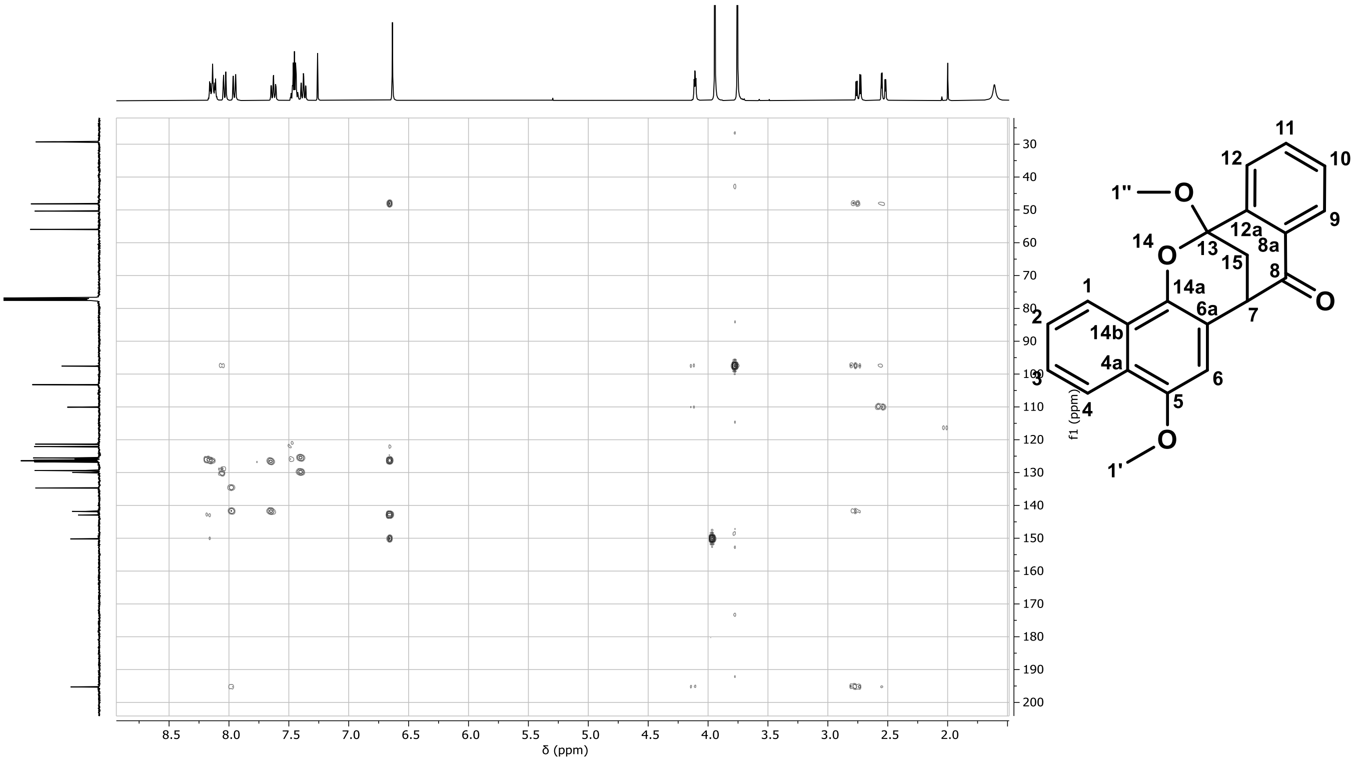

$^{1}H,^{13}C$ HMBC NMR ($CDCl_3$) of **2**.

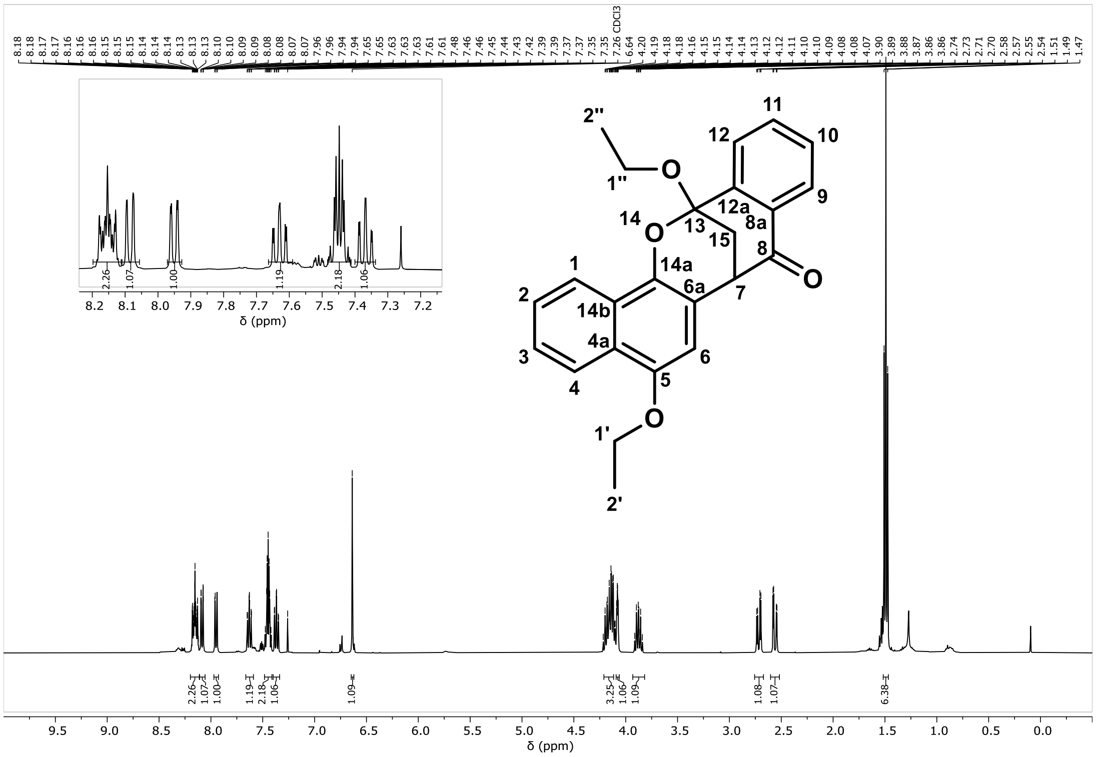

^{1}H NMR spectrum (400 MHz, $CDCl_3$) of **6**.

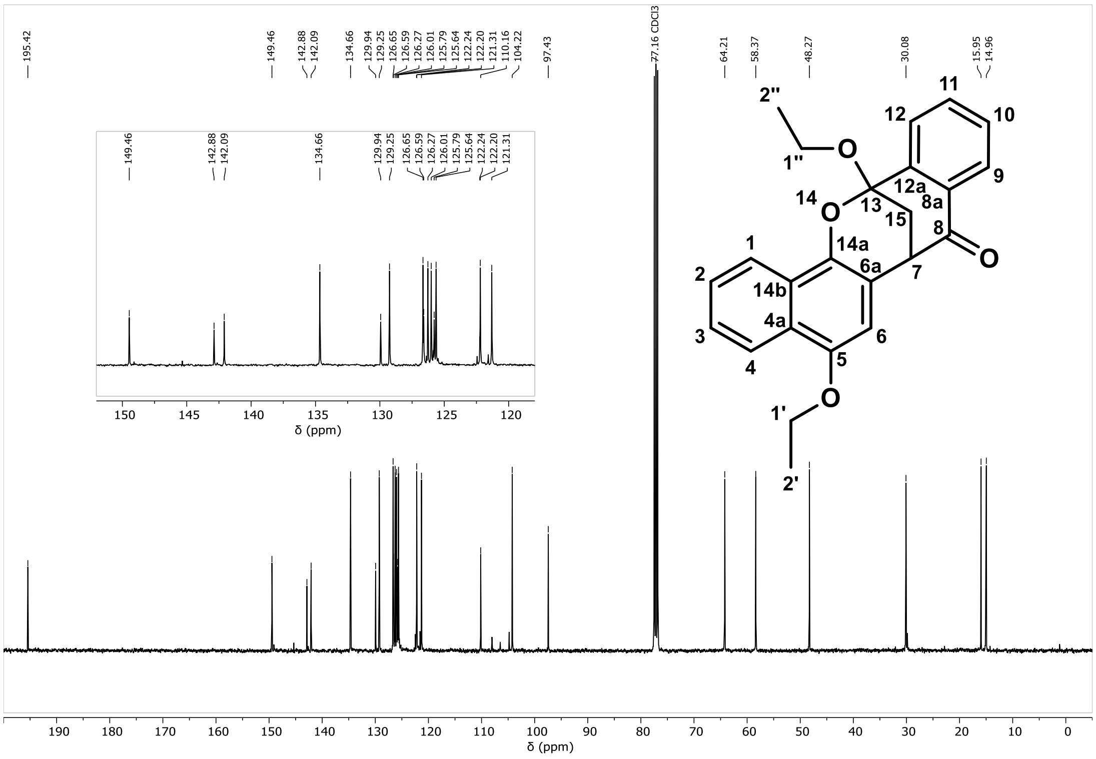

^{13}C NMR spectrum (101 MHz, $CDCl_3$) of **6**.

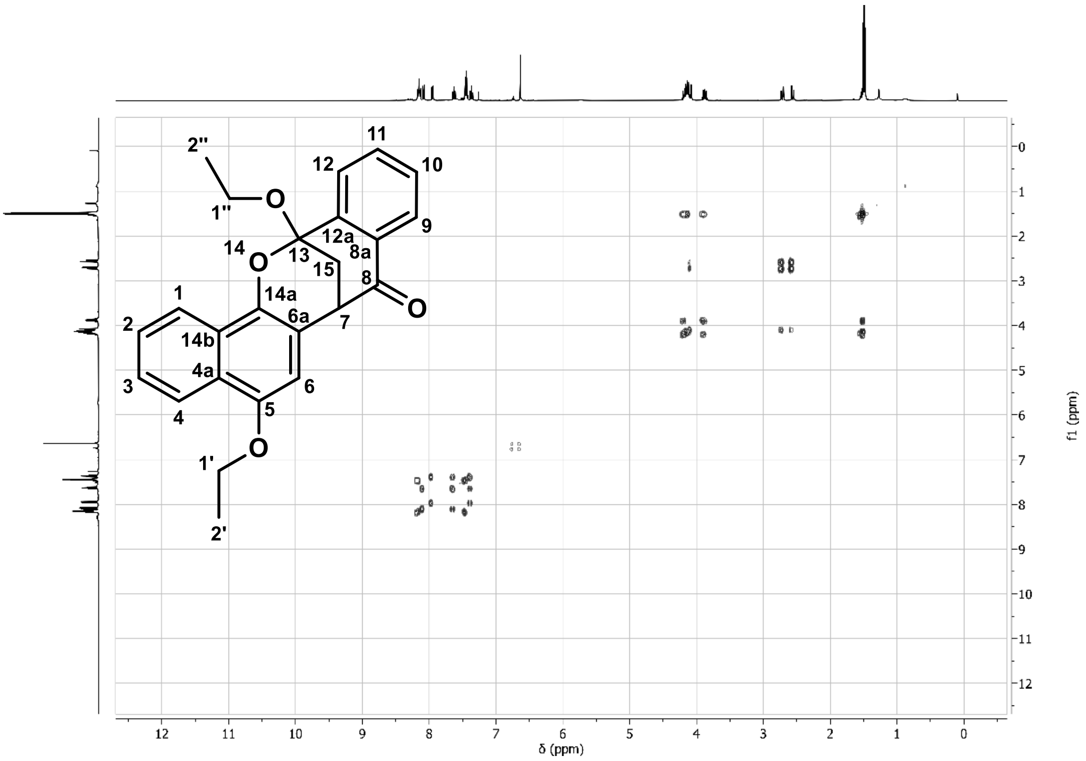

$^{1}H,^{1}H$ COSY NMR ($CDCl_3$) of **6**.

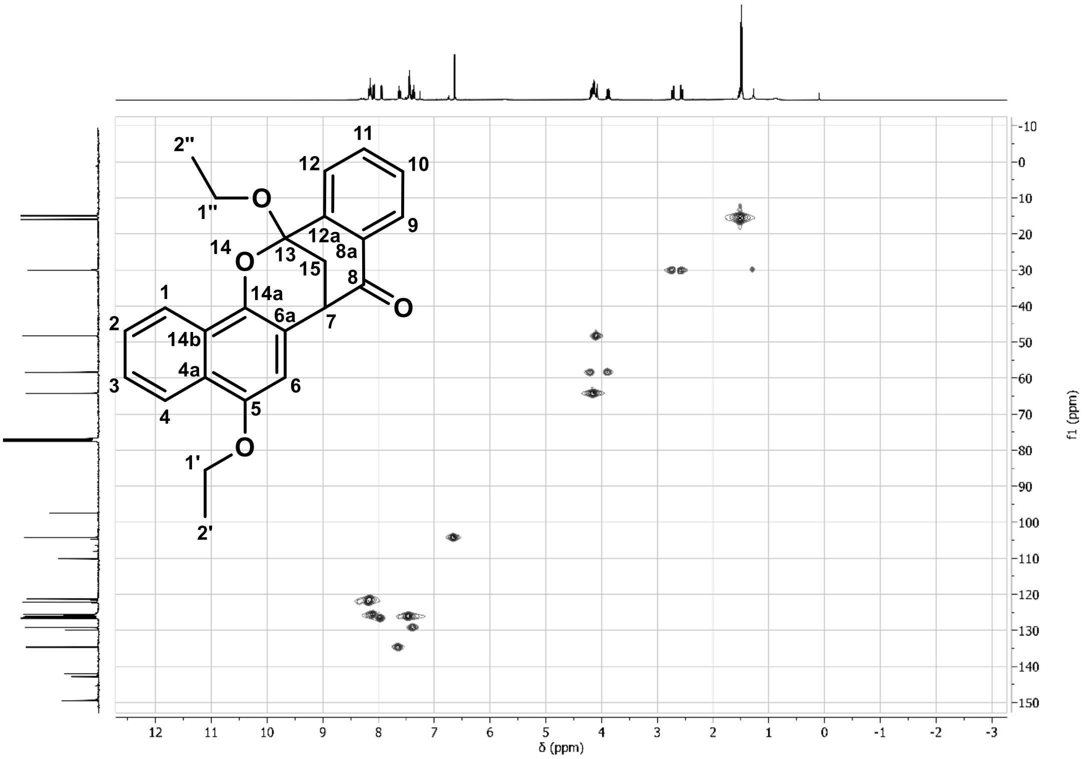

$^{1}H,^{13}C$ HSQC NMR ($CDCl_3$) of **6**.

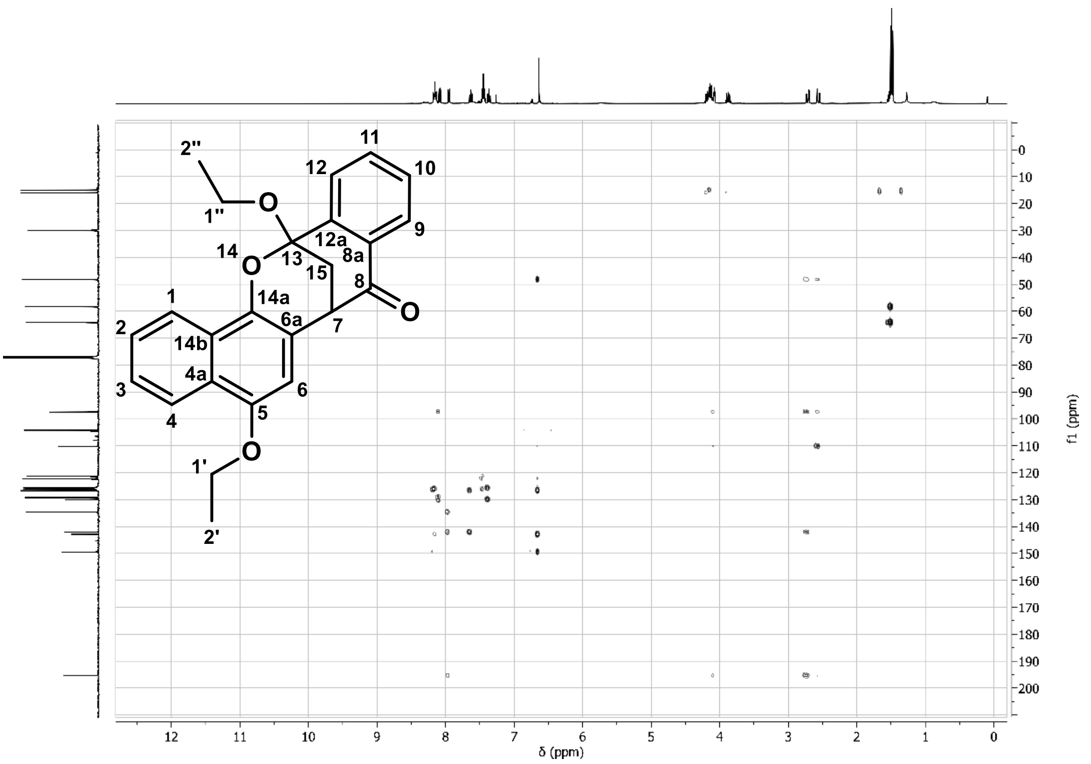

^{1}H, ^{13}C HMBC NMR ($CDCl_3$) of **6**.

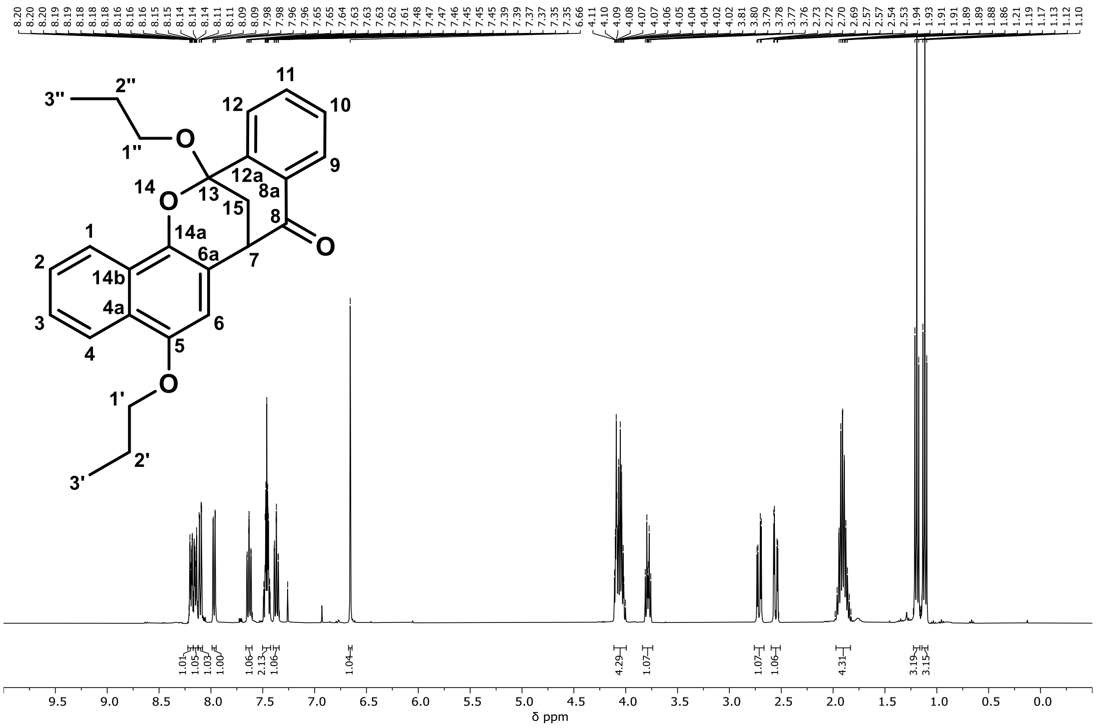

^{1}H NMR spectrum (400 MHz, $CDCl_3$) of **7**.

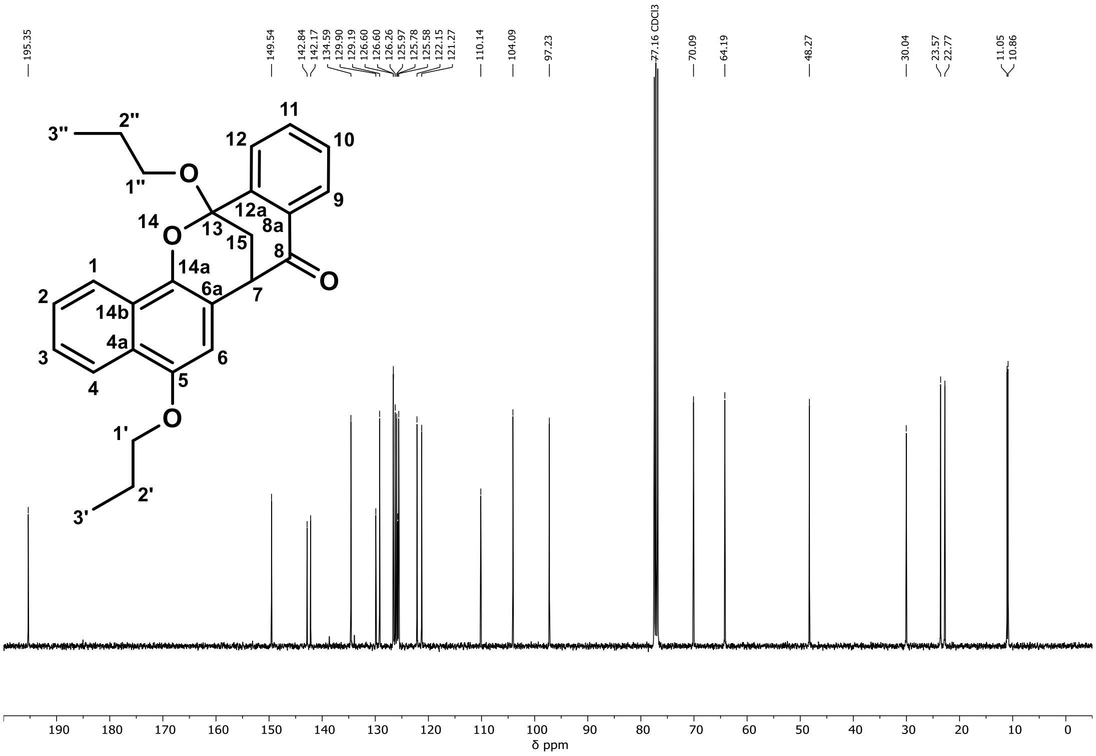

^{13}C NMR spectrum (101 MHz, CDCl$_3$) of **7**.

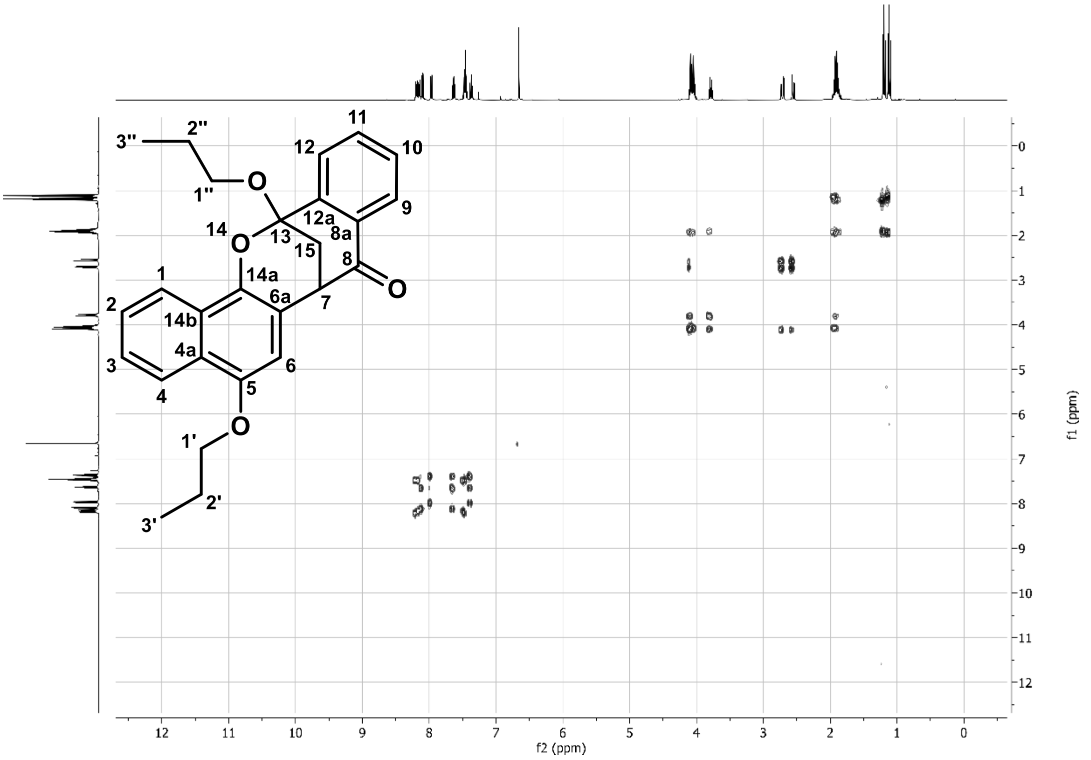

^{1}H,^{1}H COSY NMR (CDCl$_3$) of **7**.

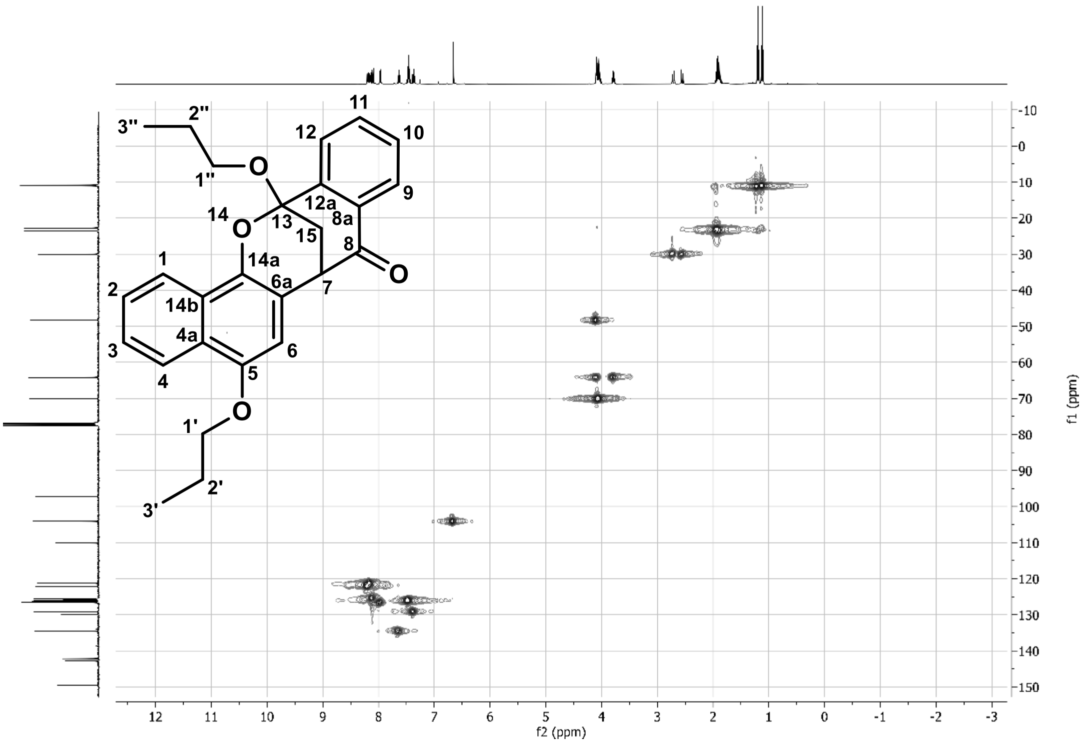

^{1}H,^{13}C HSQC NMR ($CDCl_3$) of **7**.

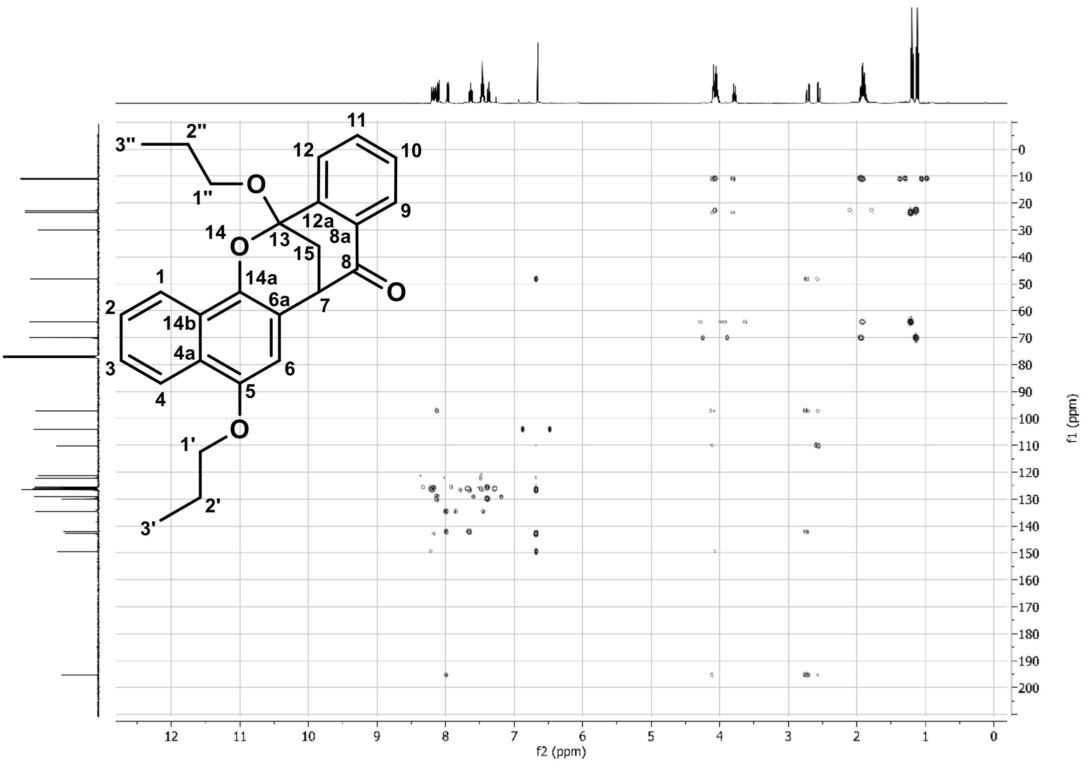

^{1}H, ^{13}C HMBC NMR ($CDCl_3$) of **7**.

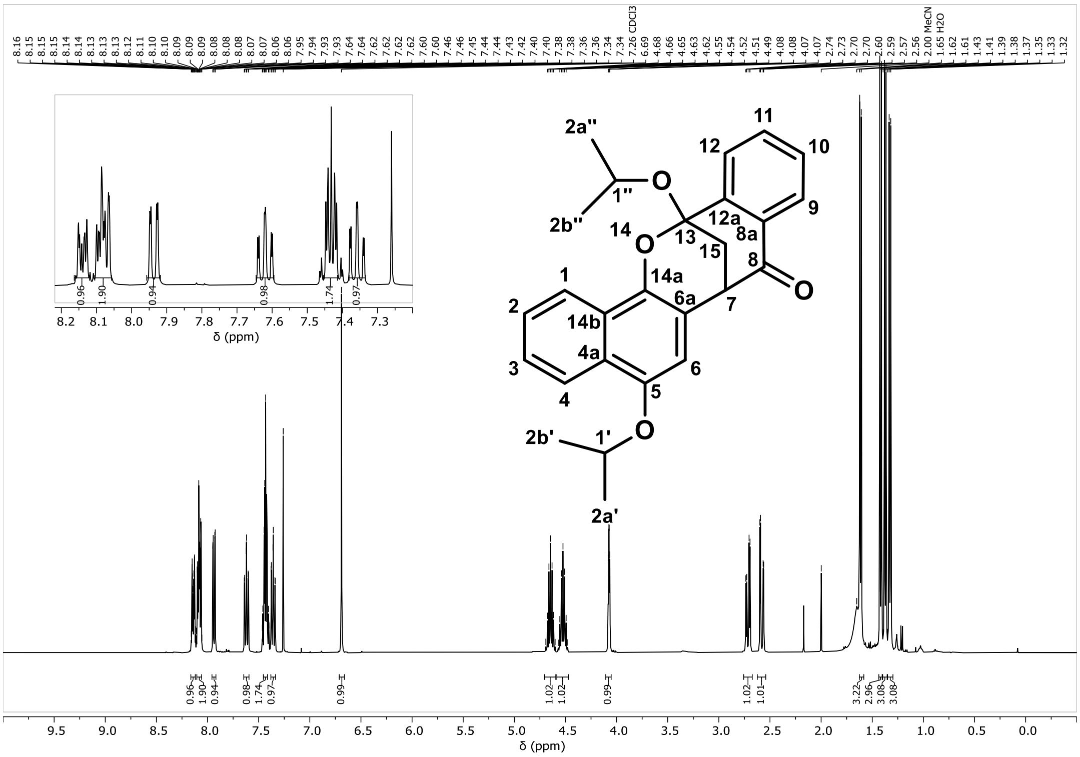

^{1}H NMR spectrum (400 MHz, $CDCl_3$) of **8**.

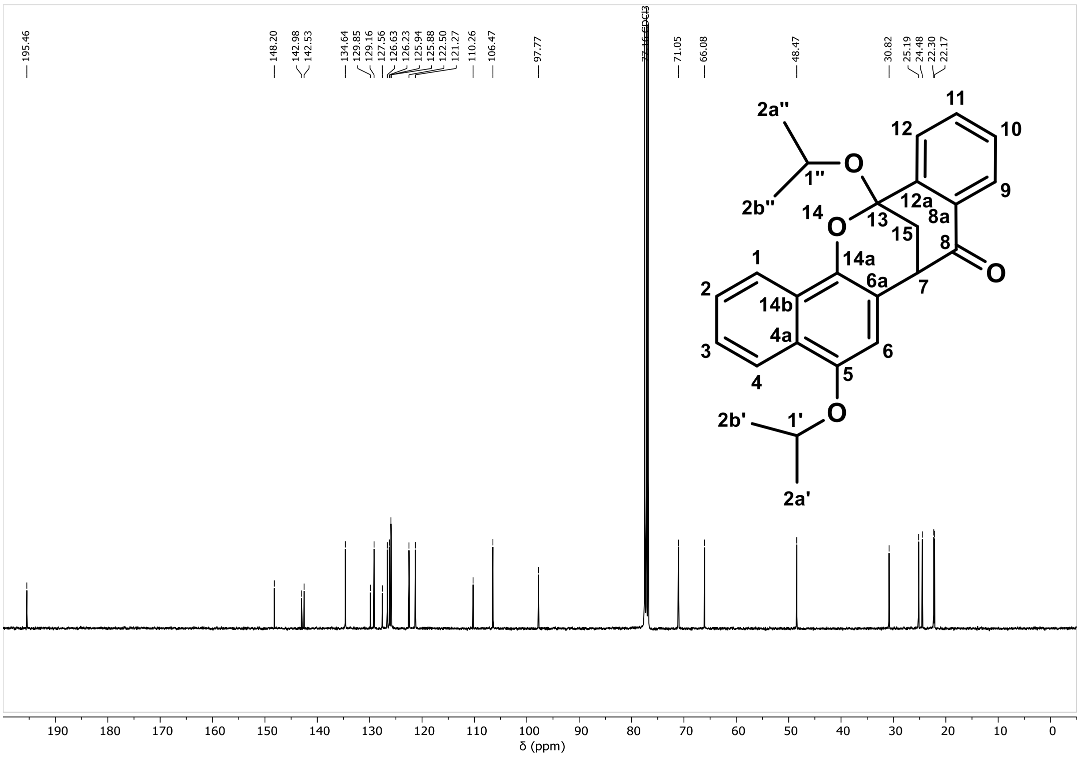

^{13}C NMR spectrum (101 MHz, $CDCl_3$) of **8**.

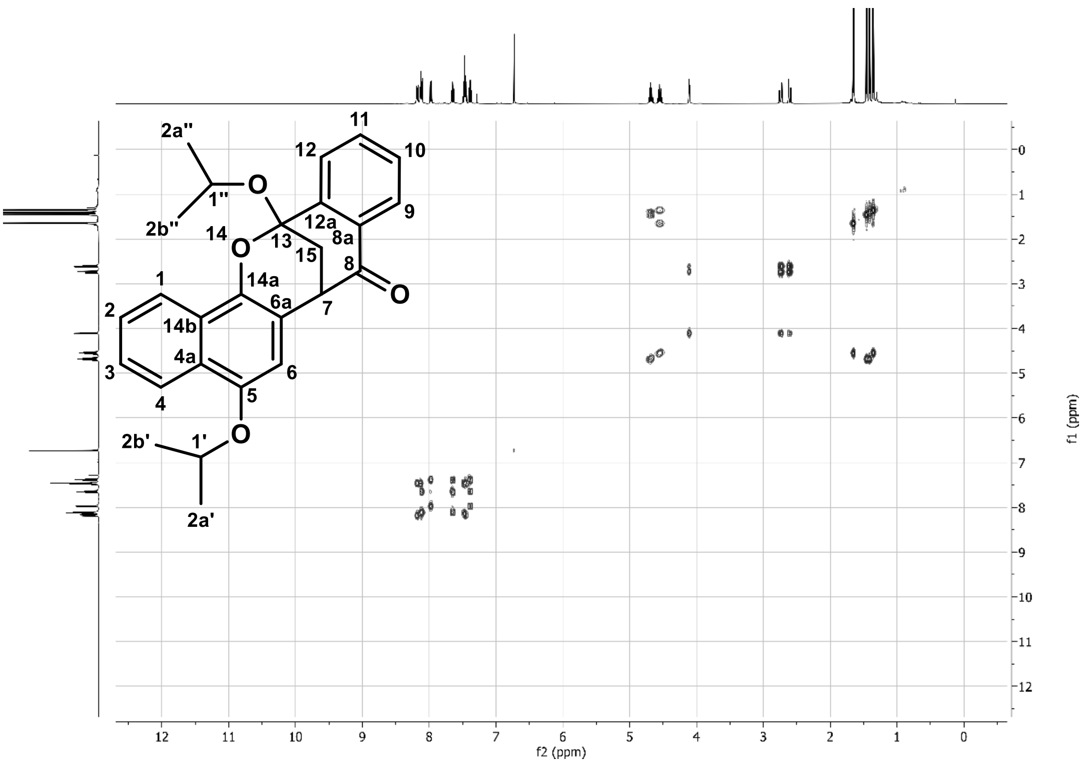

$^{1}H,^{1}H$ COSY NMR ($CDCl_3$) of **8**.

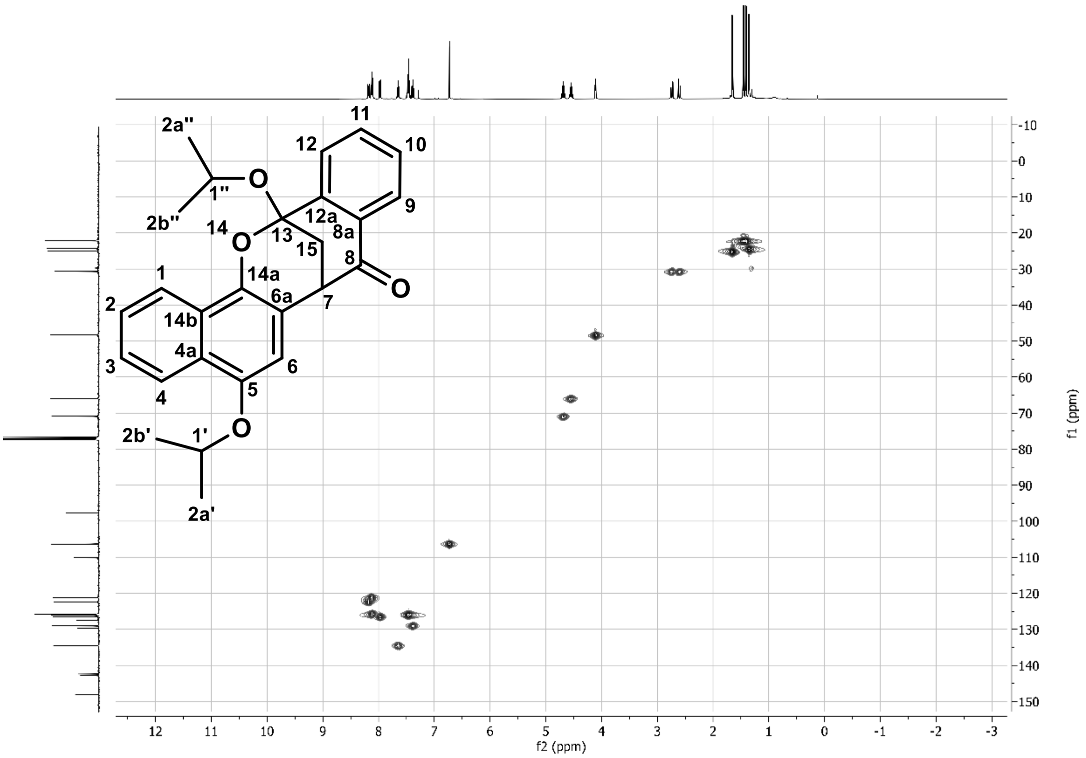

$^{1}H,^{13}C$ HSQC NMR ($CDCl_3$) of **8**.

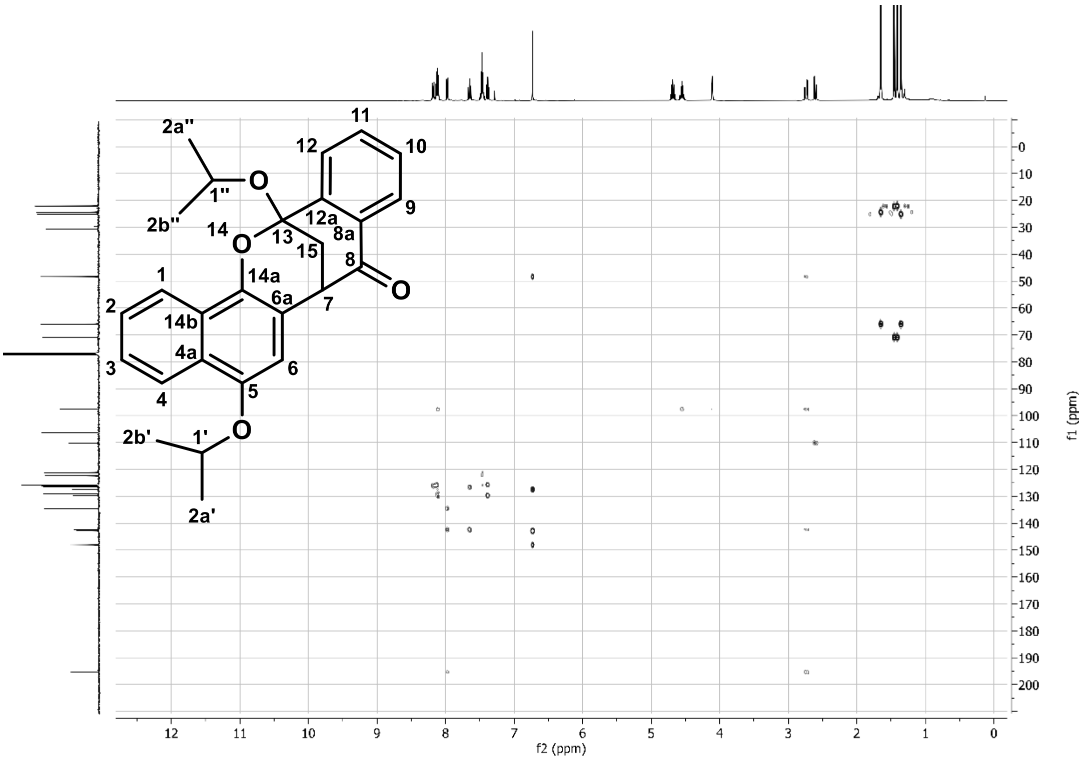

^1H, ^{13}C HMBC NMR ($CDCl_3$) of **8**.

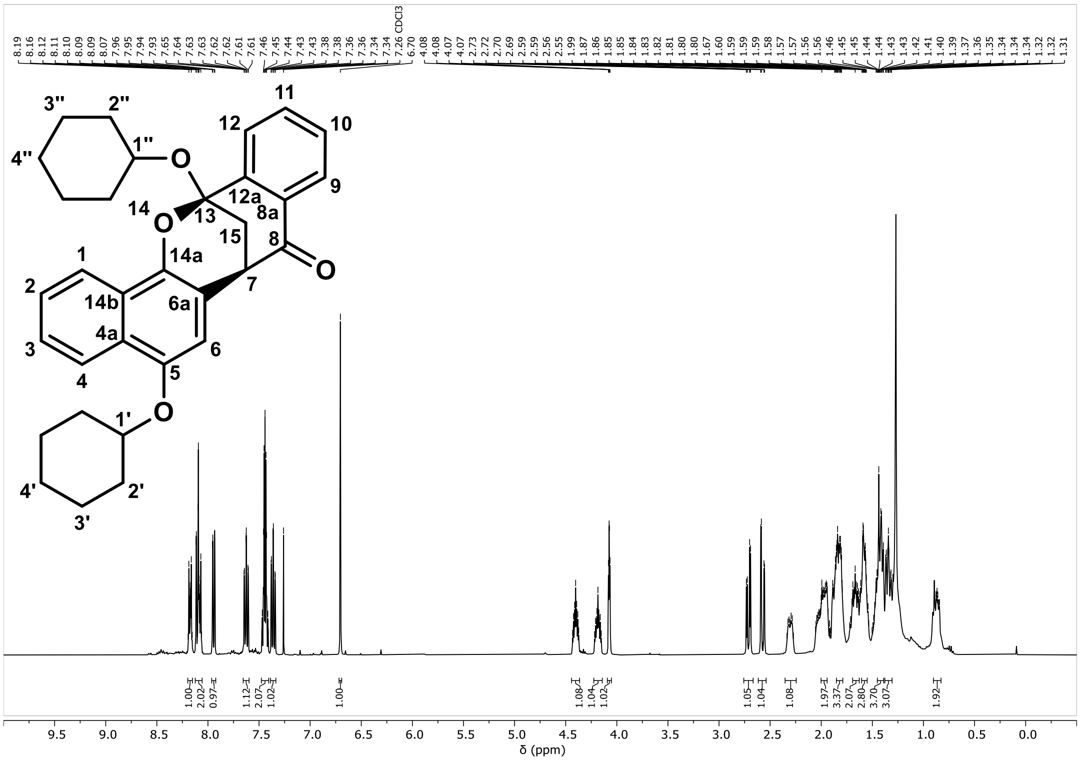

^1H NMR spectrum (500 MHz, $CDCl_3$) of **9**.

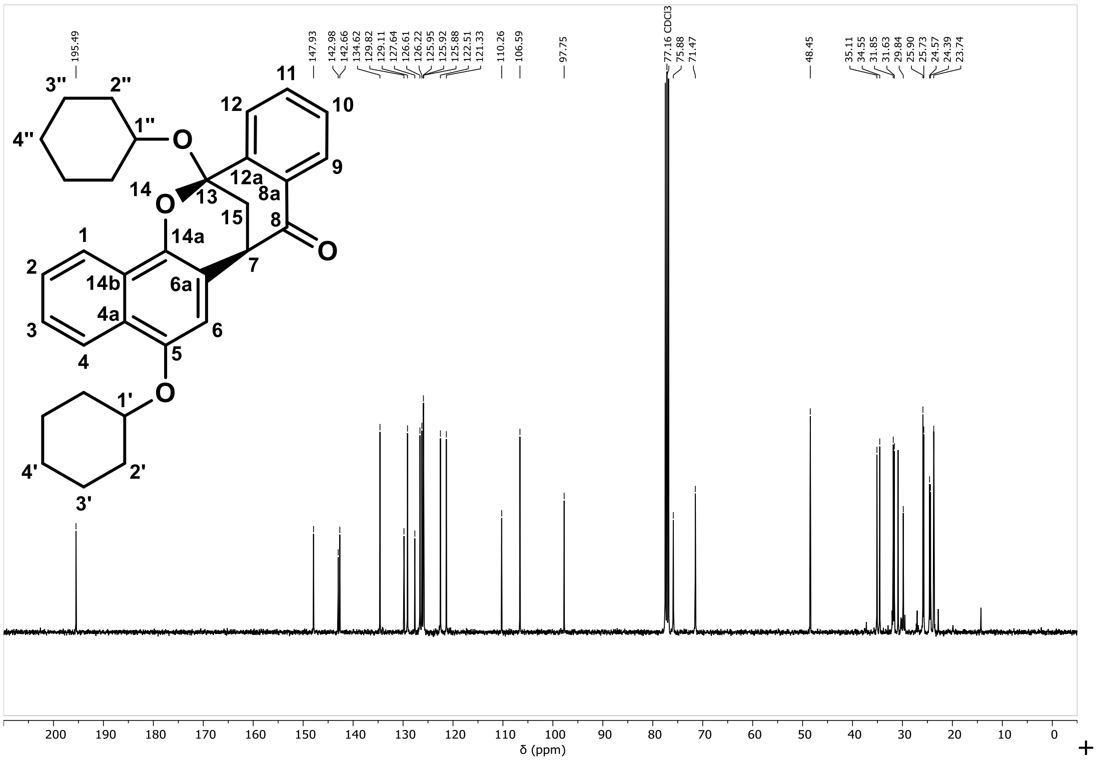

^{13}C NMR spectrum (151 MHz, $CDCl_3$) of **9**.

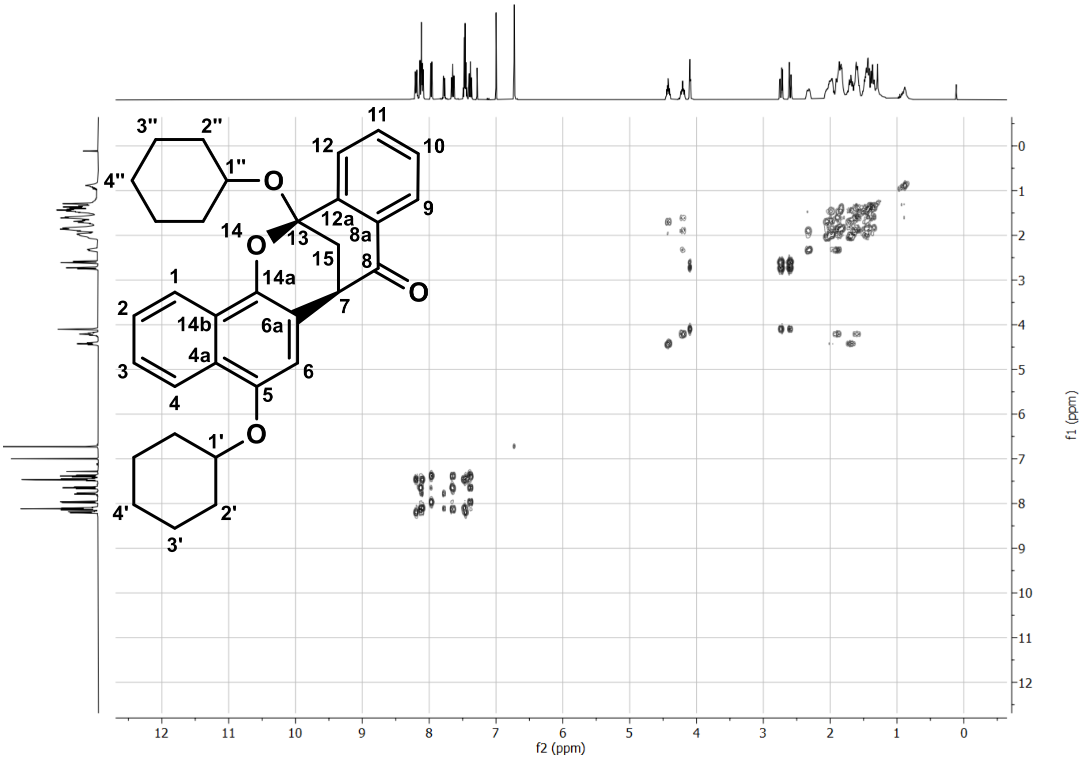

$^{1}H,^{1}H$ COSY NMR ($CDCl_3$) of **9**.

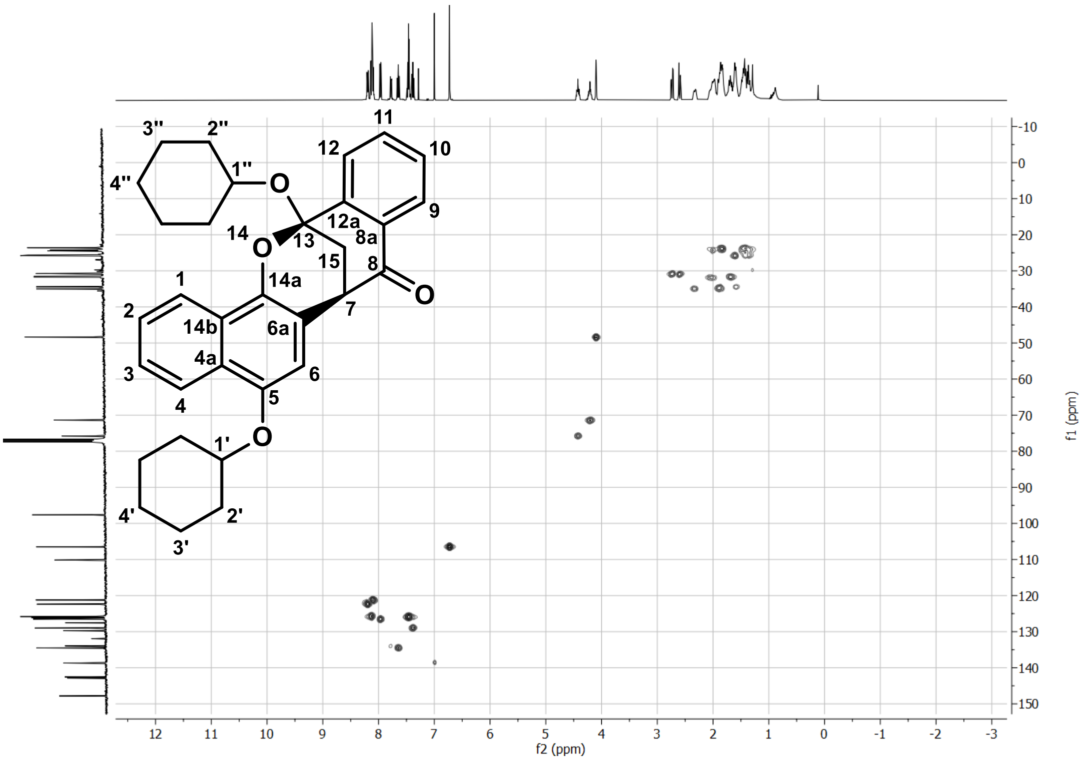

$^{1}H,^{13}C$ HSQC NMR ($CDCl_3$) of **9**.

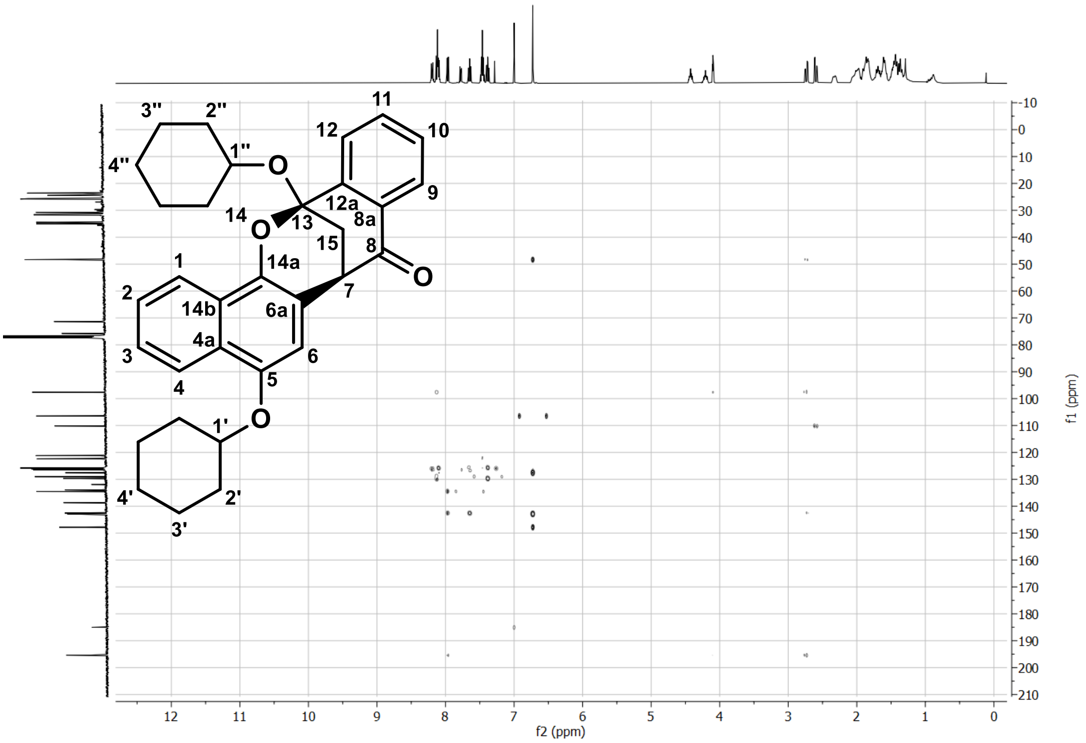

$^{1}H, ^{13}C$ HMBC NMR ($CDCl_3$) of **9**.

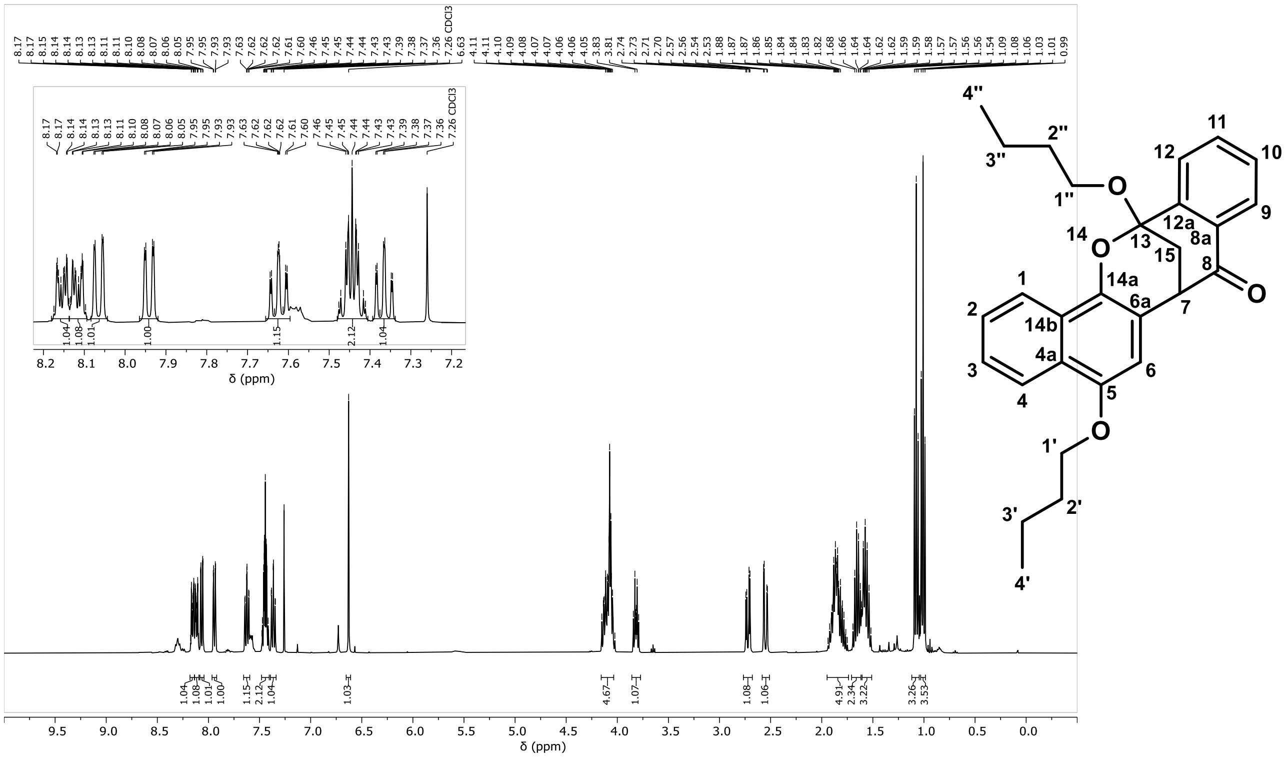

^{1}H NMR spectrum (400 MHz, $CDCl_3$) of **10**.

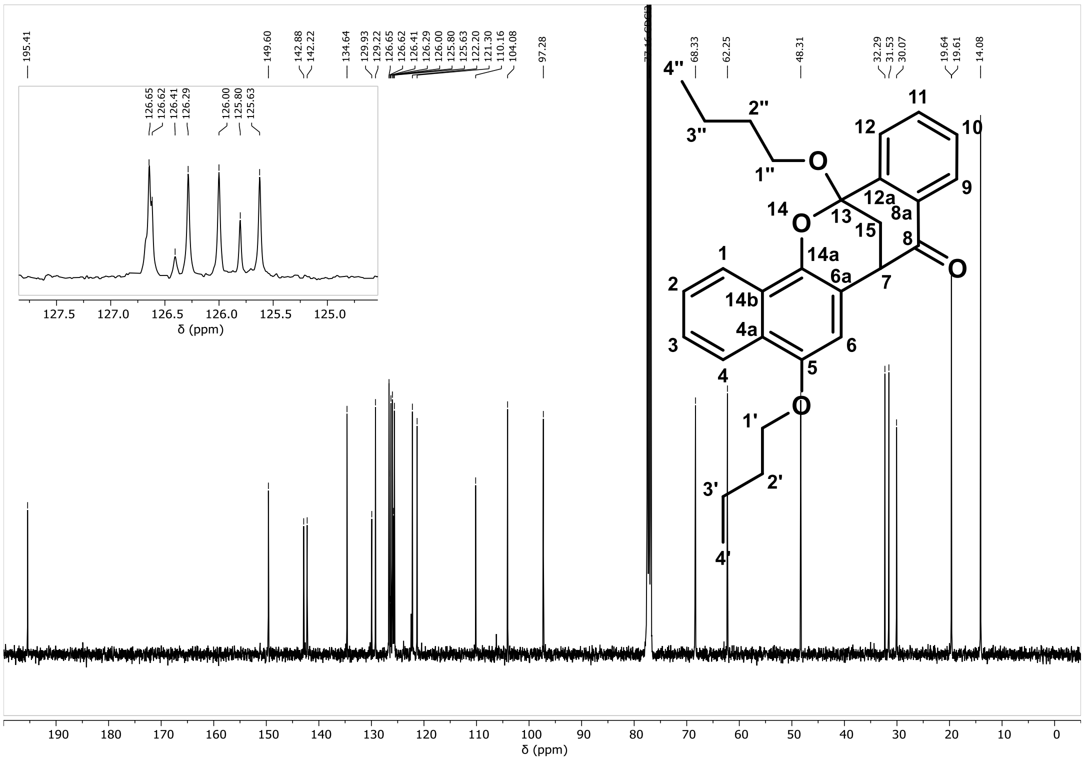

^{13}C NMR spectrum (101 MHz, $CDCl_3$) of **10**.

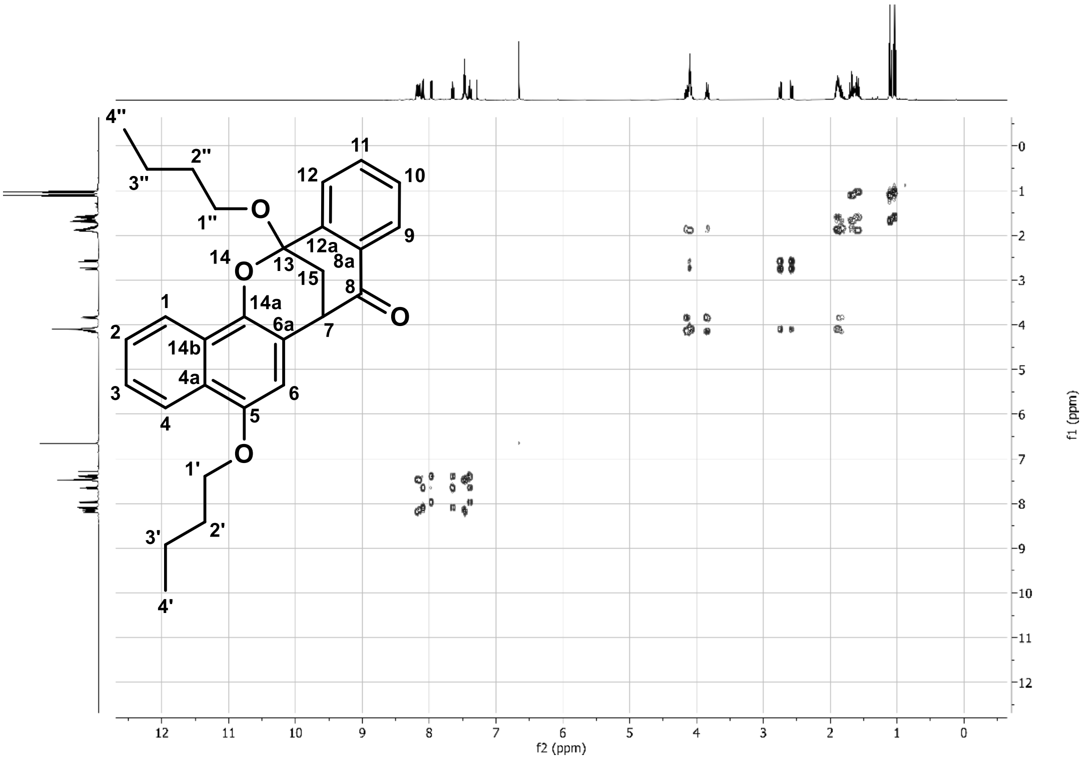

$^1H,^1H$ COSY NMR ($CDCl_3$) of **10**.

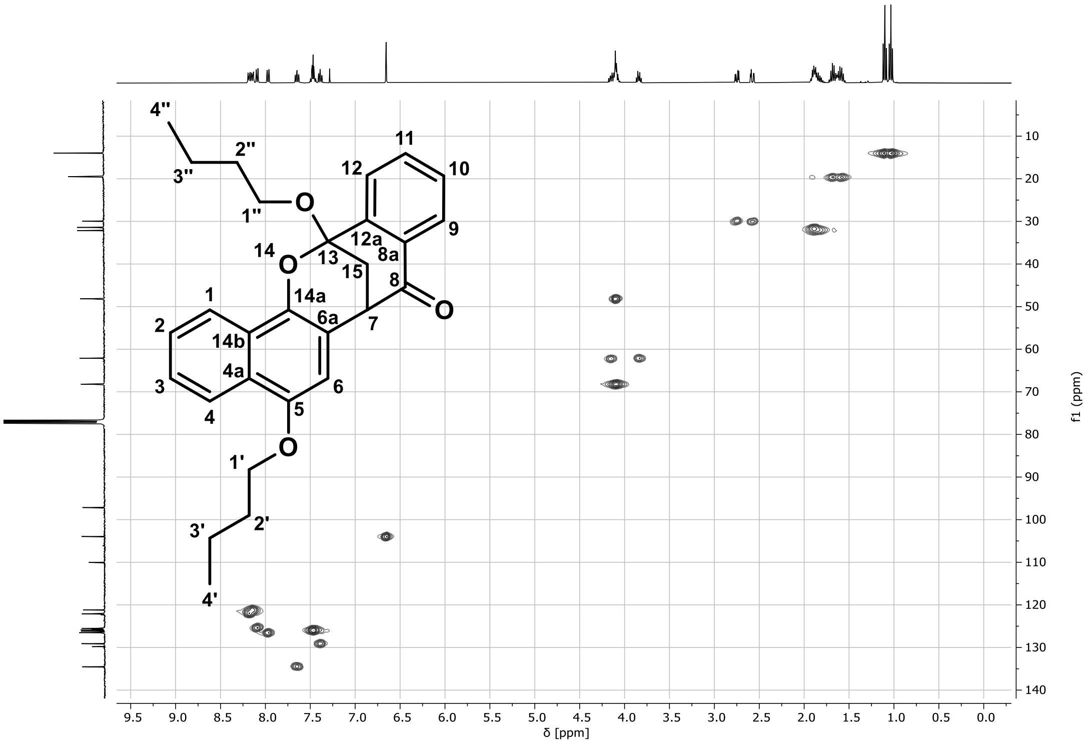

$^1H,^{13}C$ HSQC NMR ($CDCl_3$) of **10**.

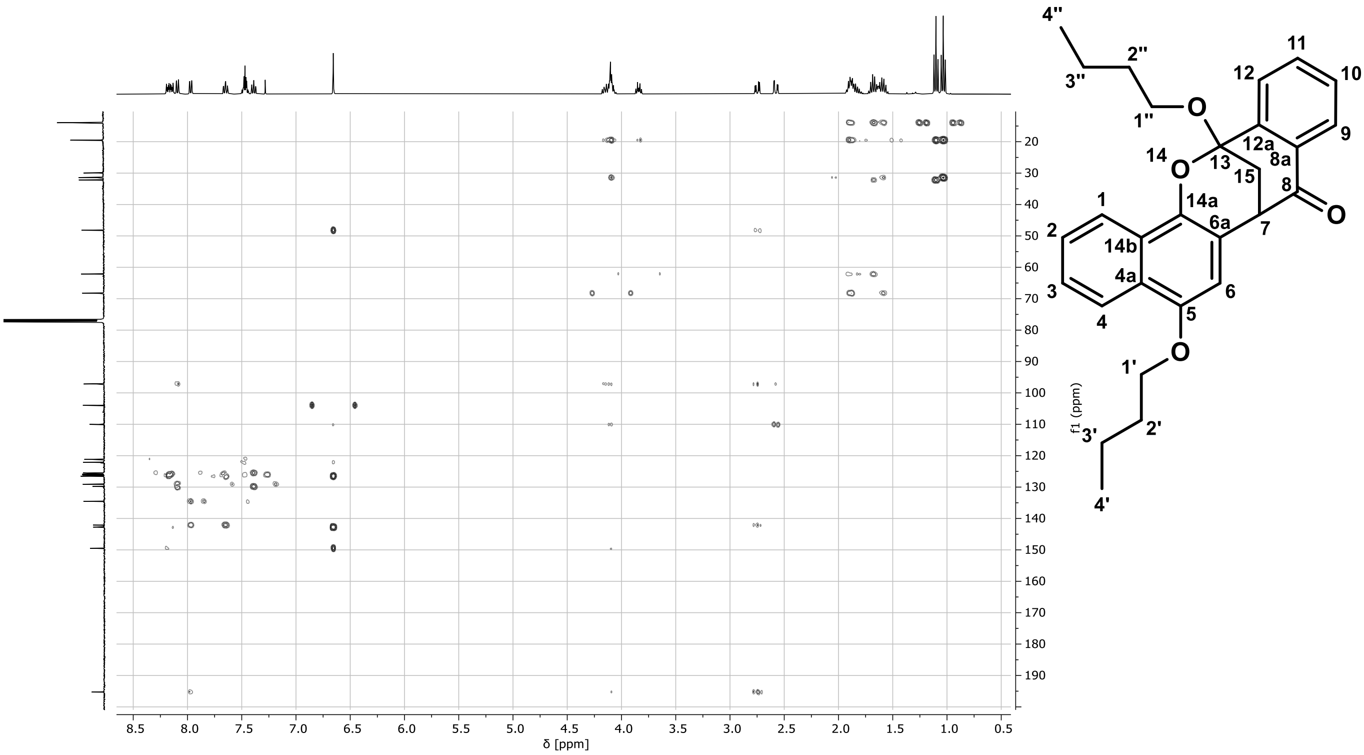

$^{1}H,^{13}C$ HMBC NMR ($CDCl_3$) of **10**.

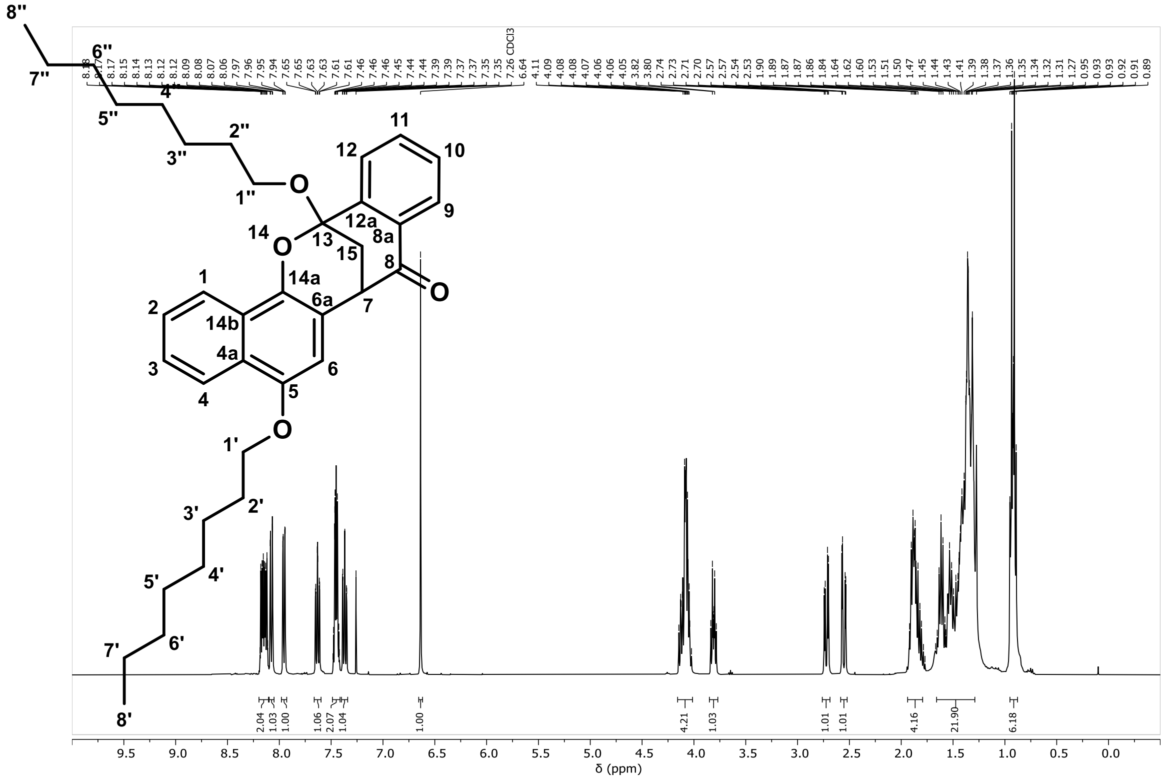

^{1}H NMR spectrum (400 MHz, $CDCl_3$) of **11**.

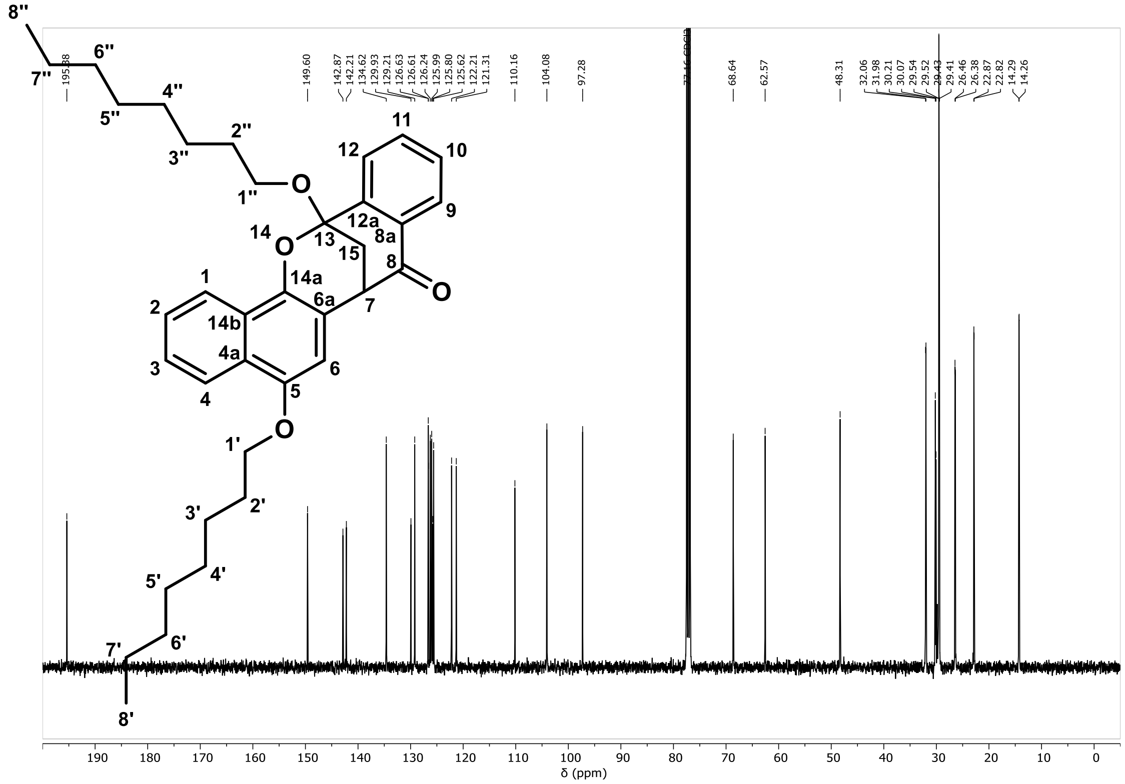

^{13}C NMR spectrum (101 MHz, $CDCl_3$) of **11**.

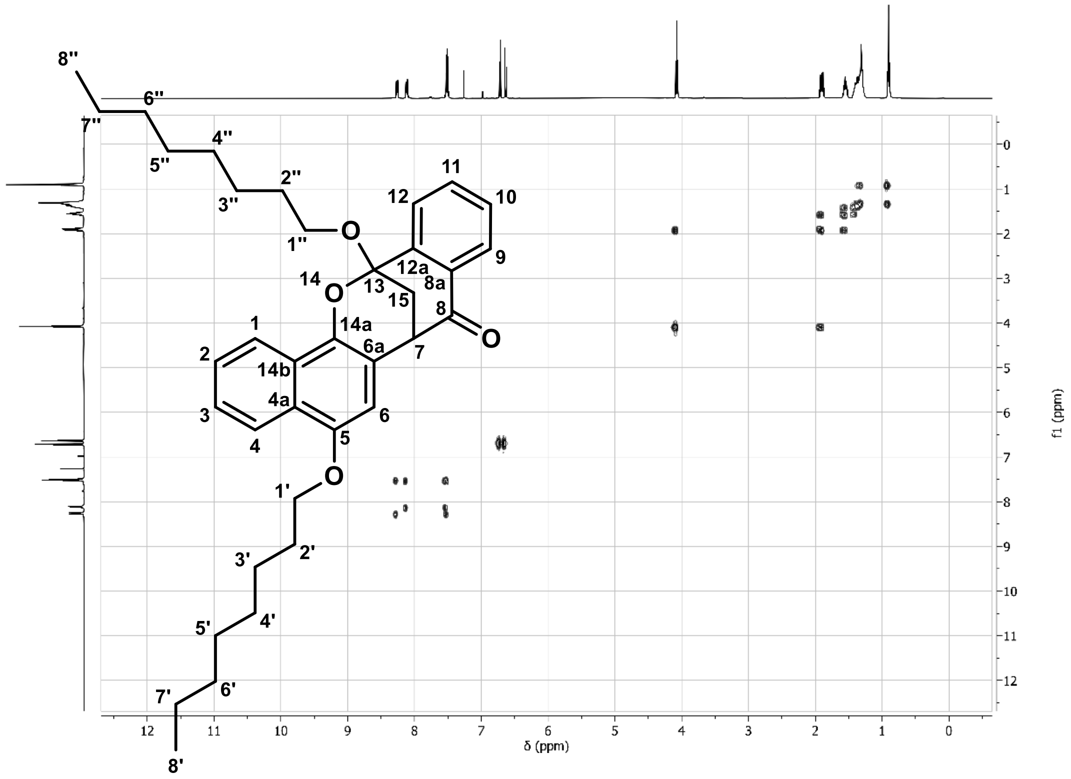

^{1}H,^{1}H COSY NMR ($CDCl_3$) of **11**.

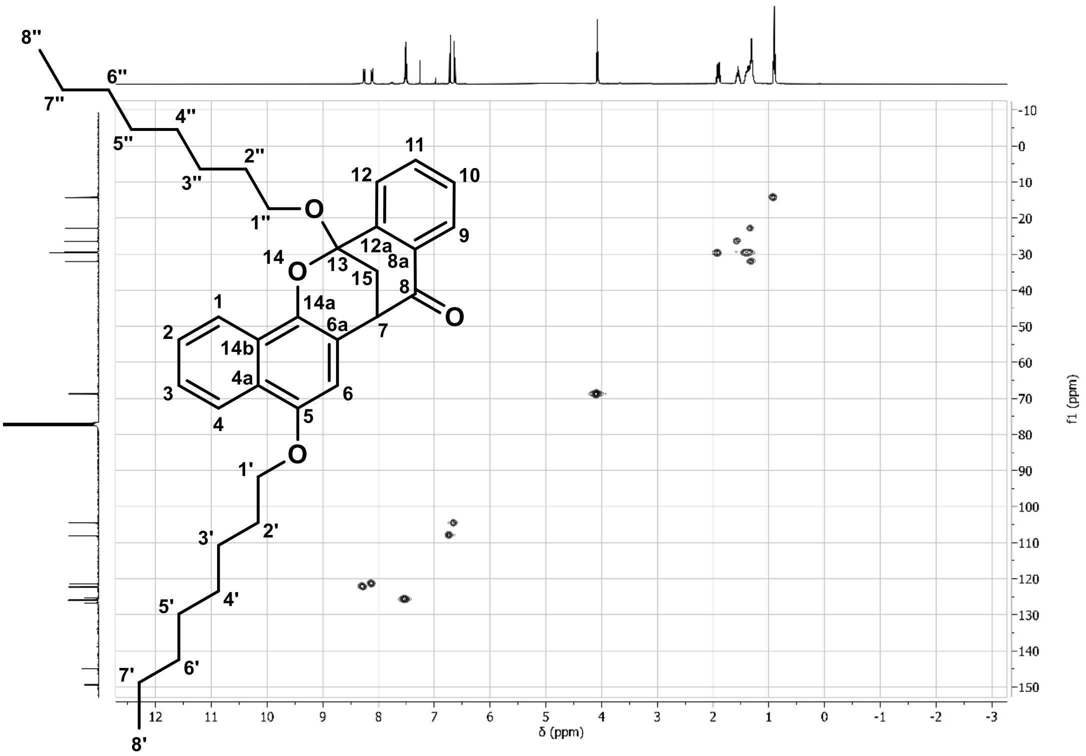

$^1H,^{13}C$ HSQC NMR ($CDCl_3$) of **11**.

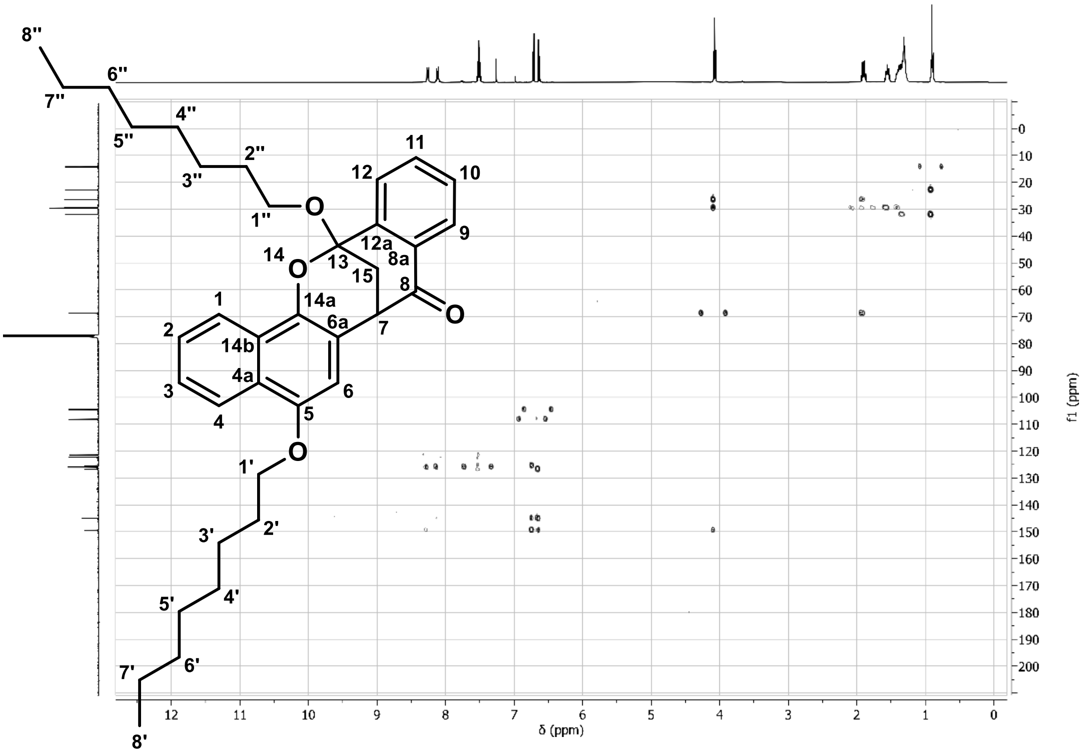

$^1H, ^{13}C$ HMBC NMR ($CDCl_3$) of **11**.

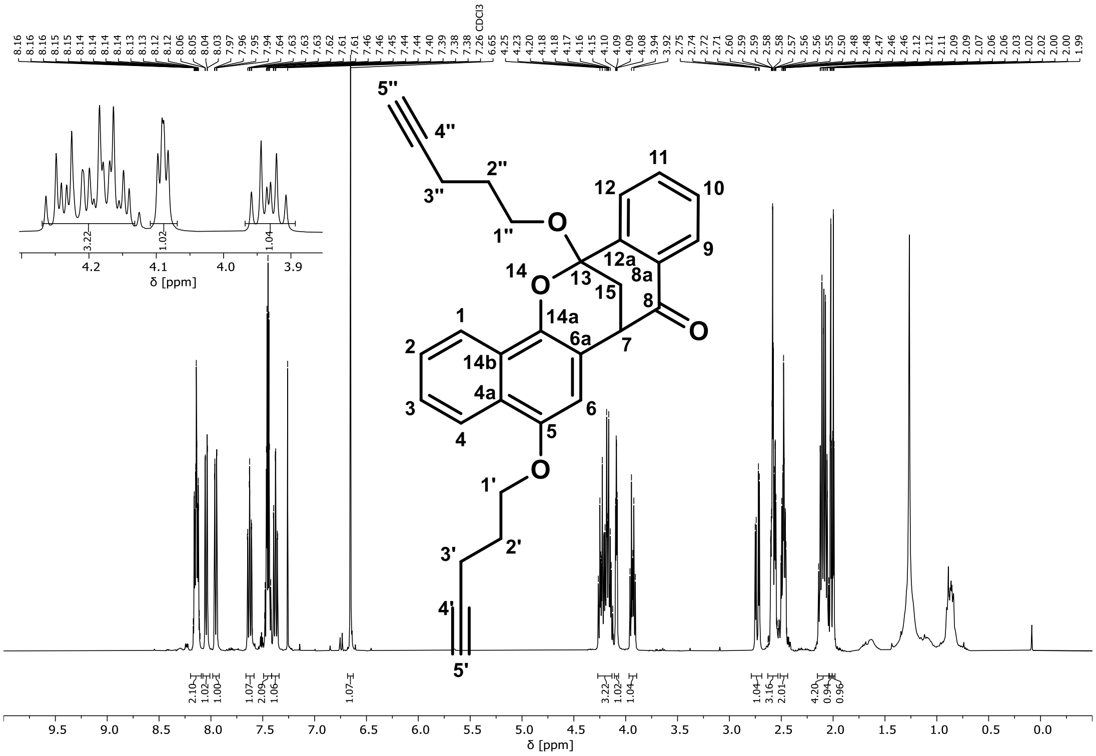

^{1}H NMR spectrum (400 MHz, $CDCl_3$) of **12**.

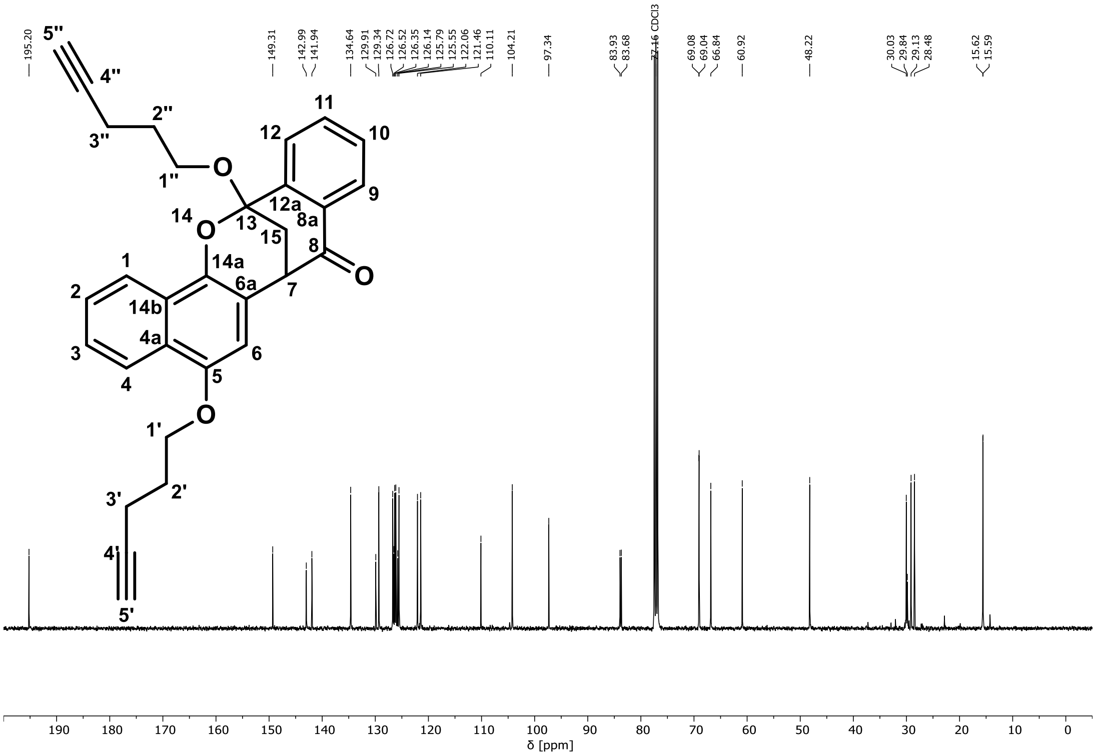

^{13}C NMR spectrum (101 MHz, $CDCl_3$) of **12**.

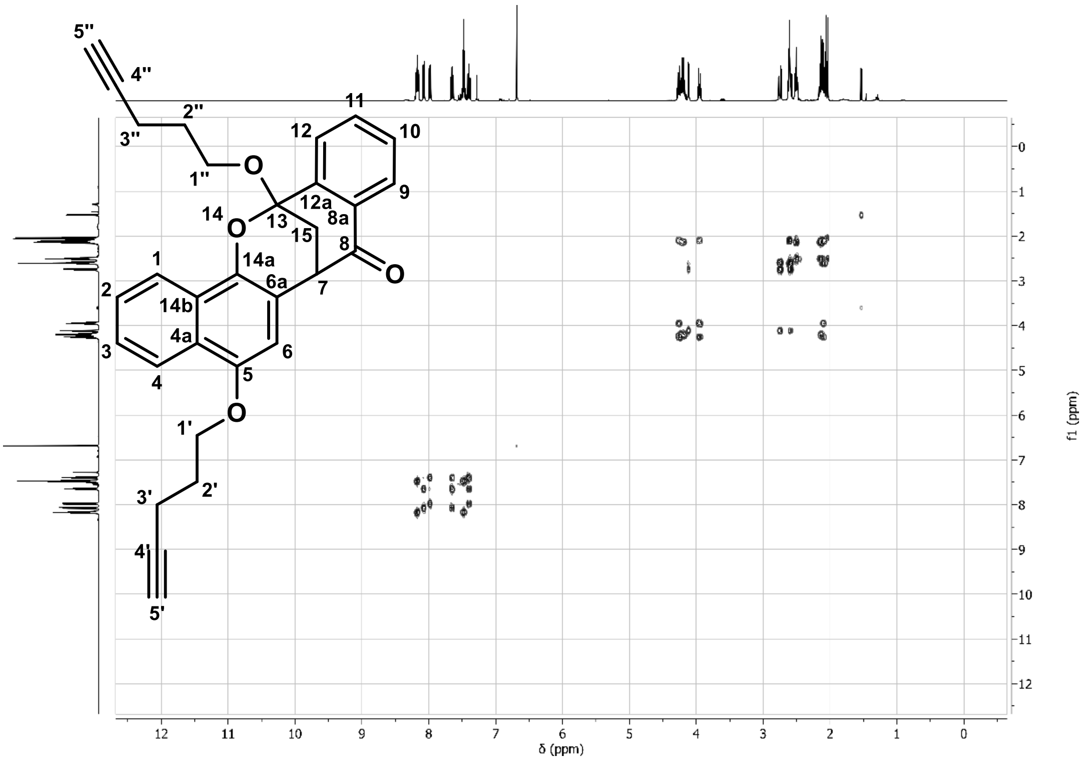

^{1}H,^{1}H COSY NMR ($CDCl_3$) of **12**.

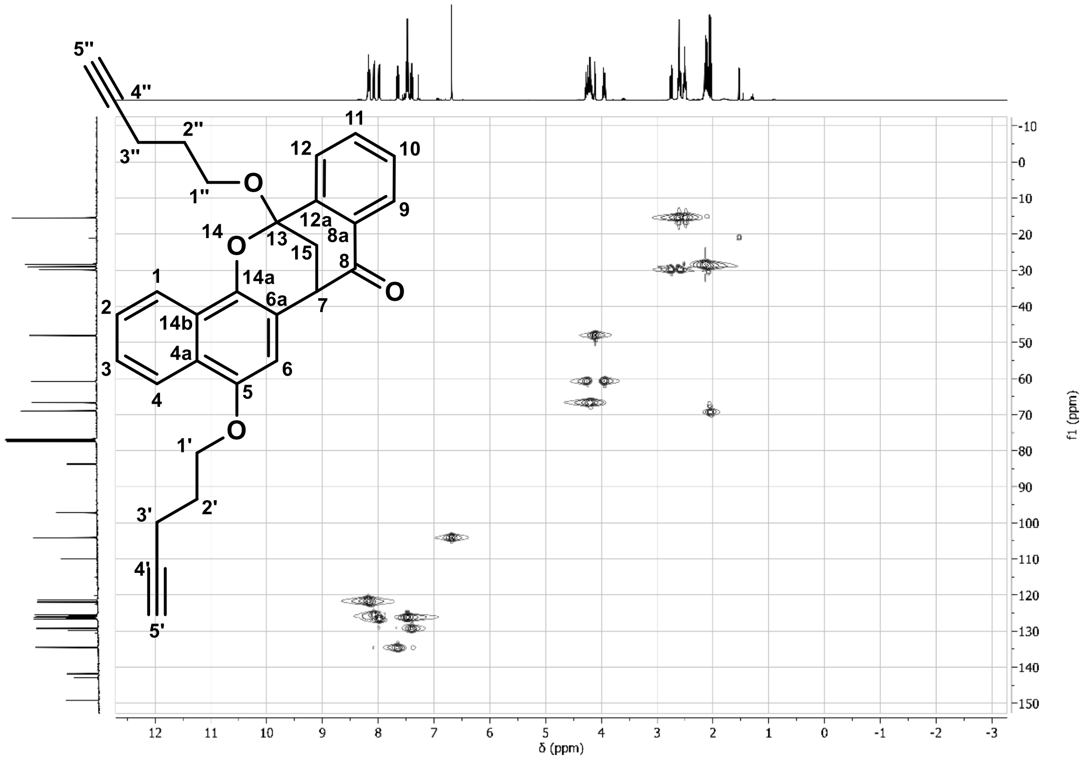

^{1}H,^{13}C HSQC NMR ($CDCl_3$) of **12**.

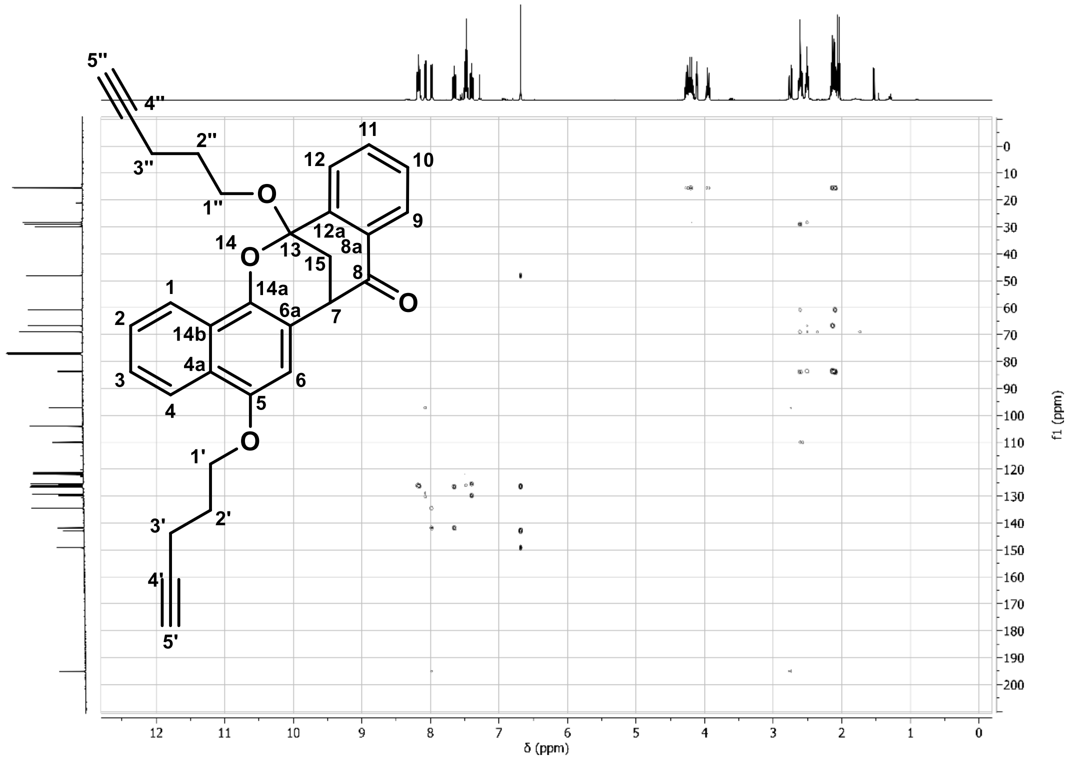

^{1}H, ^{13}C HMBC NMR ($CDCl_3$) of **12**.

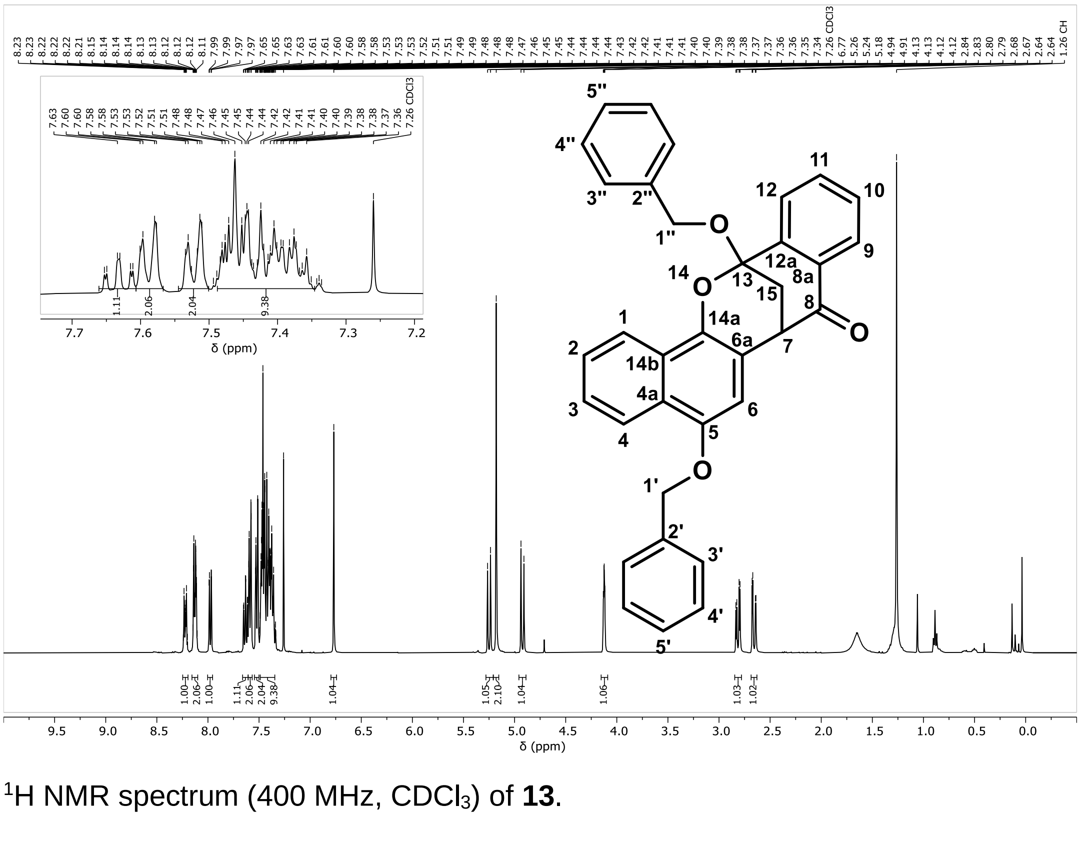

^{1}H NMR spectrum (400 MHz, $CDCl_3$) of **13**.

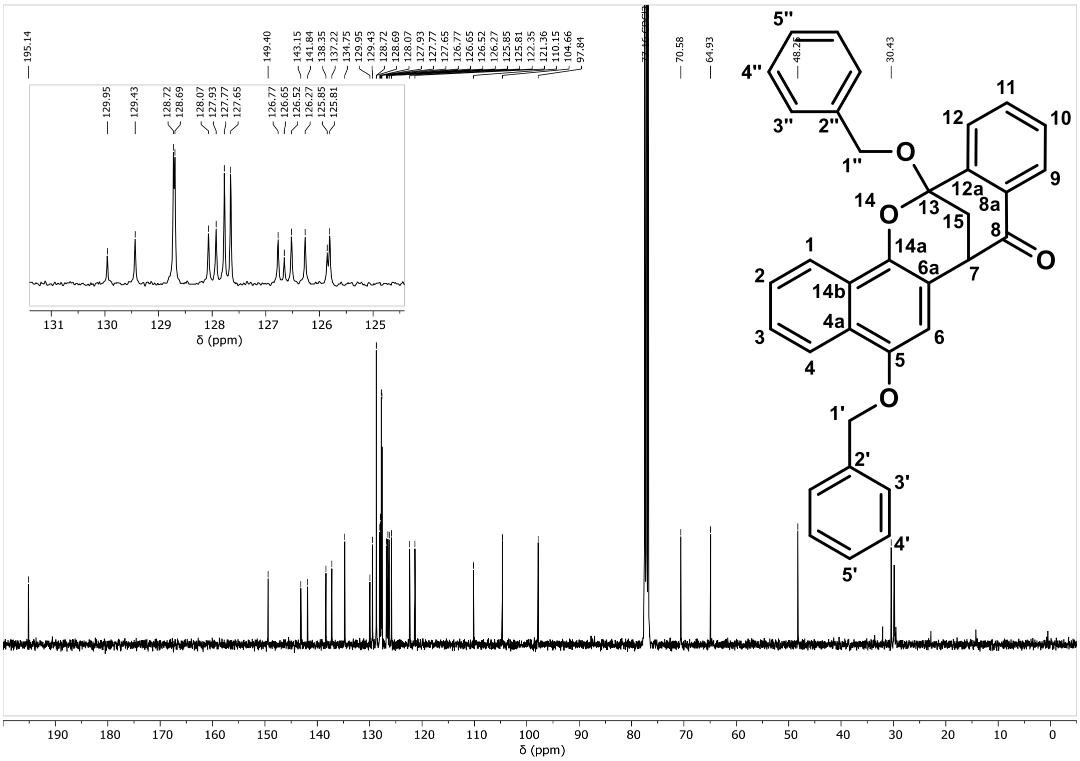

^{13}C NMR spectrum (101 MHz, $CDCl_3$) of **13**.

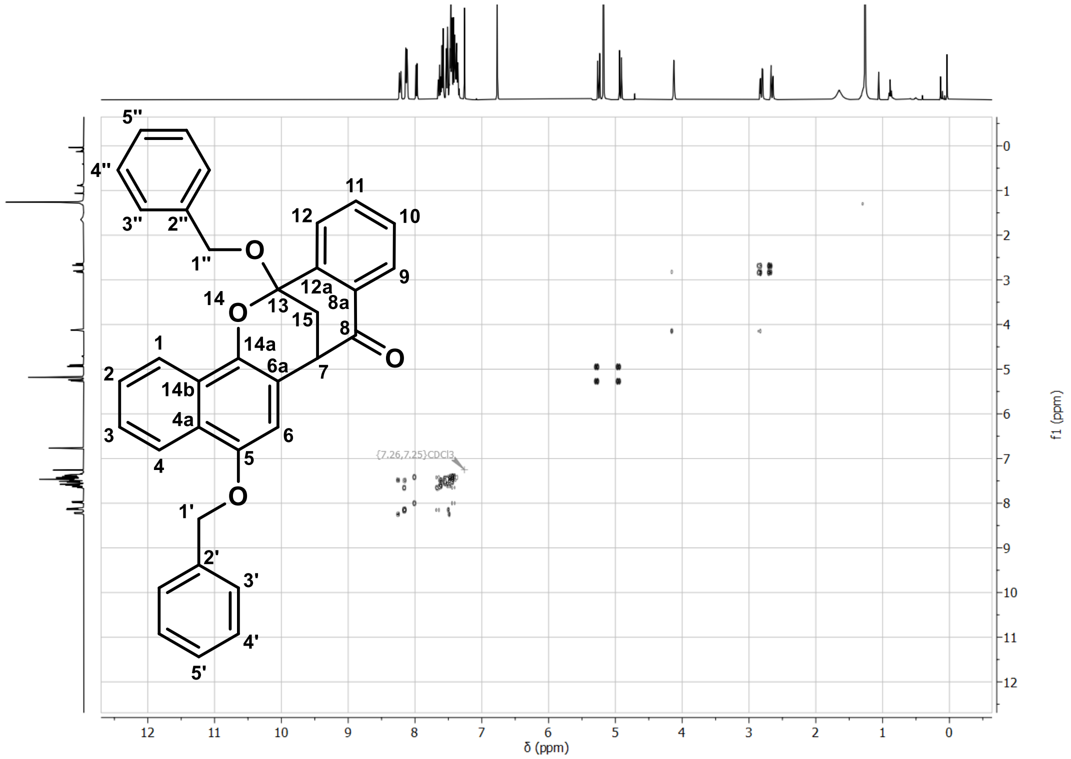

^{1}H,^{1}H COSY NMR ($CDCl_3$) of **13**.

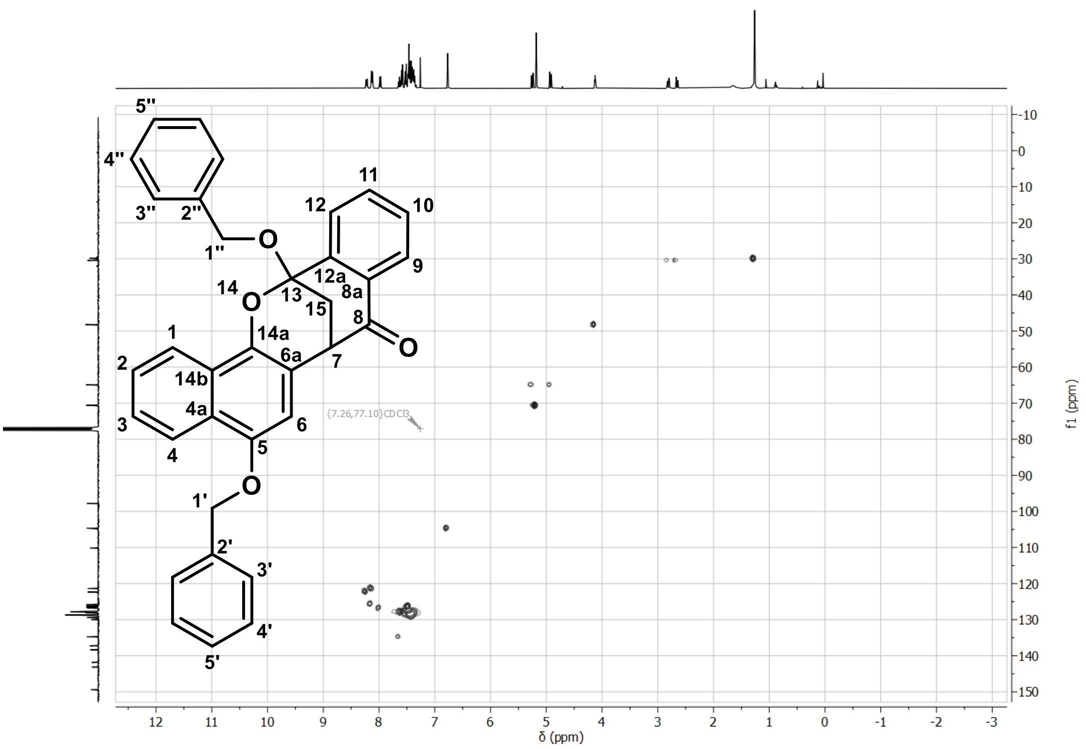

^{1}H,^{13}C HSQC NMR (CDCl$_3$) of **13**.

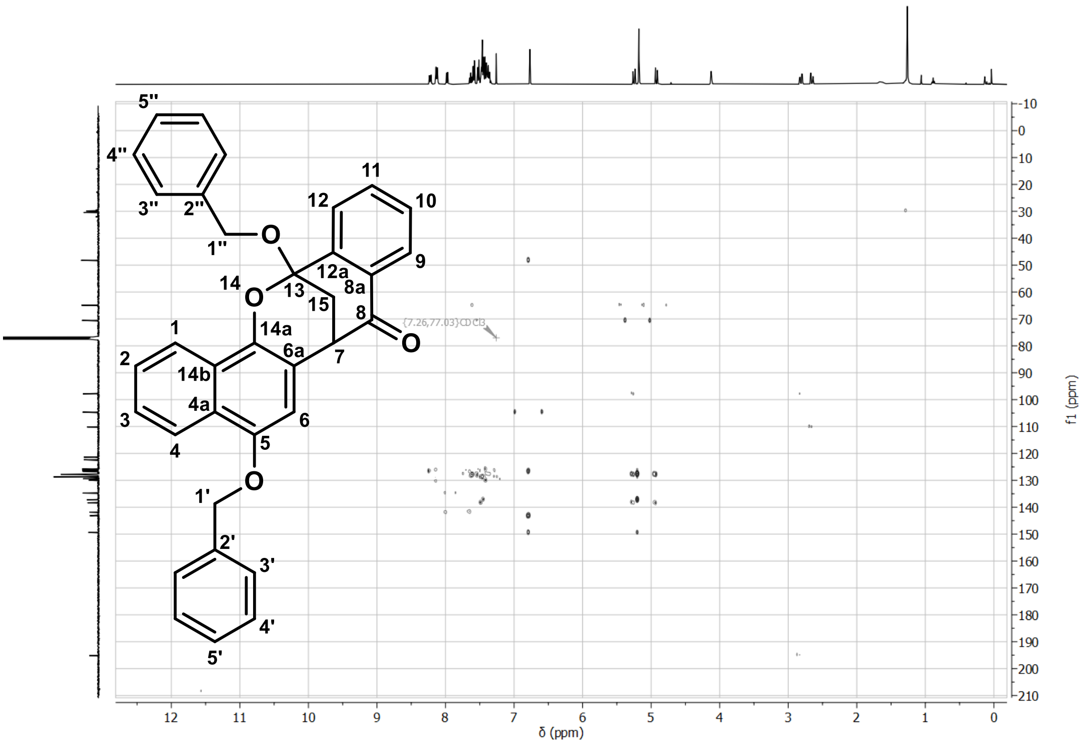

^{1}H, ^{13}C HMBC NMR (CDCl$_3$) of **13**.

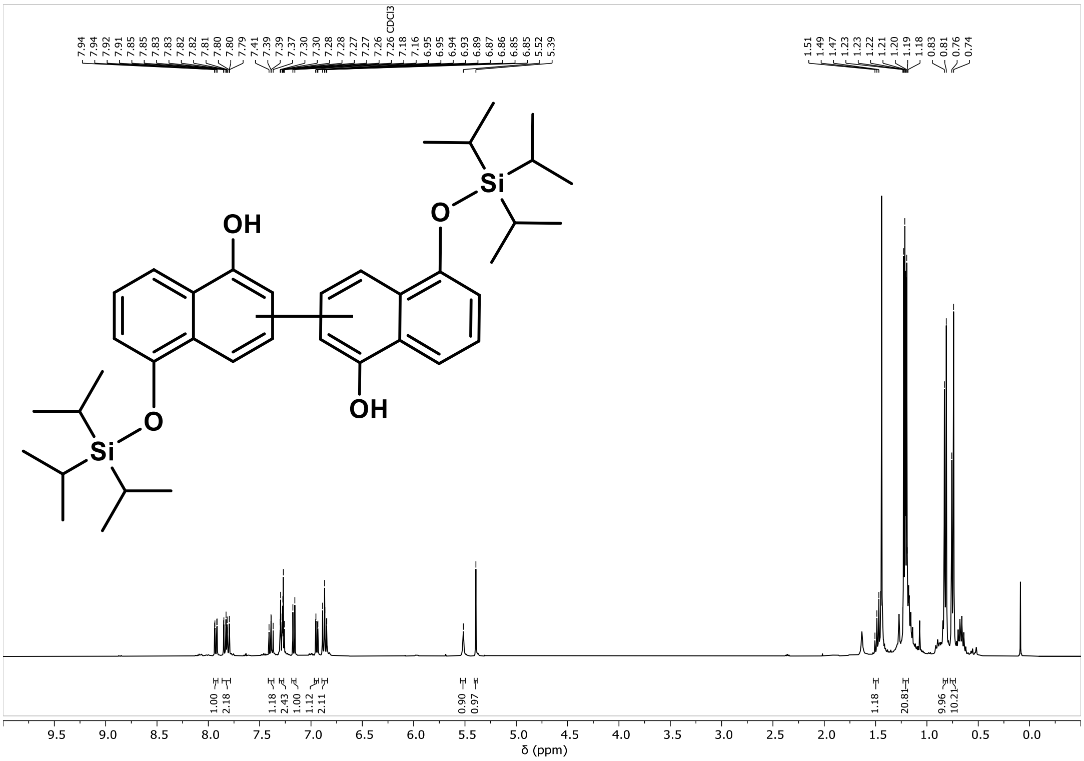

^{1}H NMR spectrum (400 MHz, $CDCl_3$) of **60**.

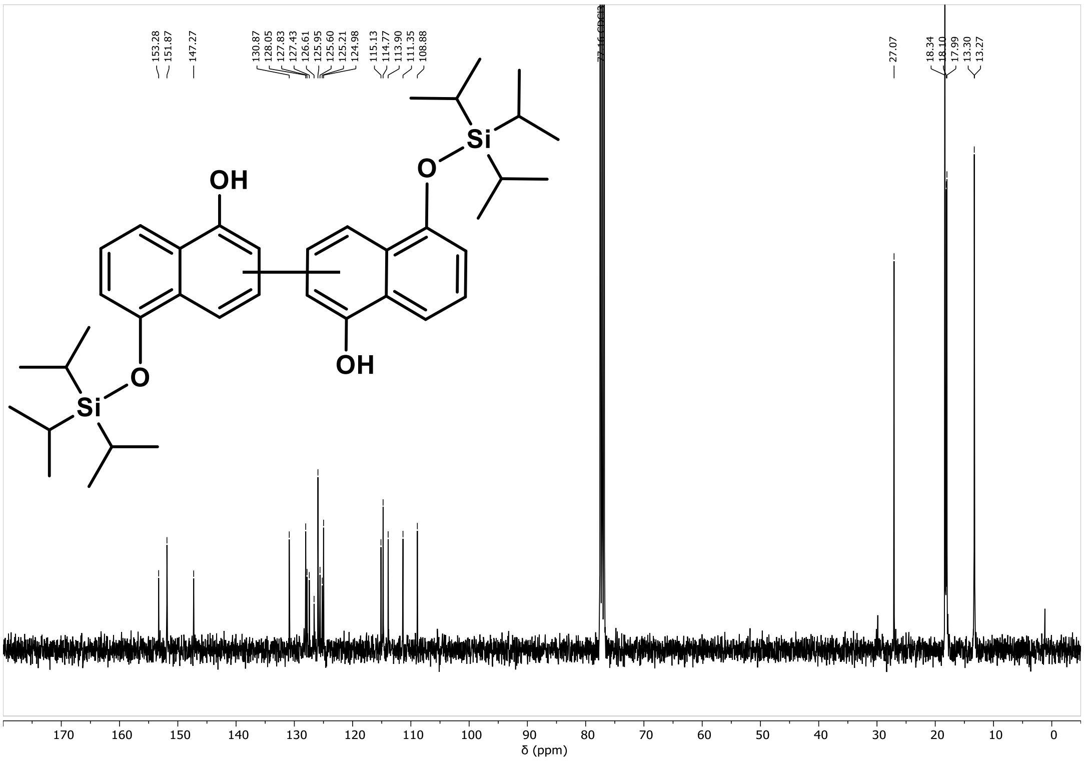

^{13}C NMR spectrum (101 MHz, $CDCl_3$) of **60**.

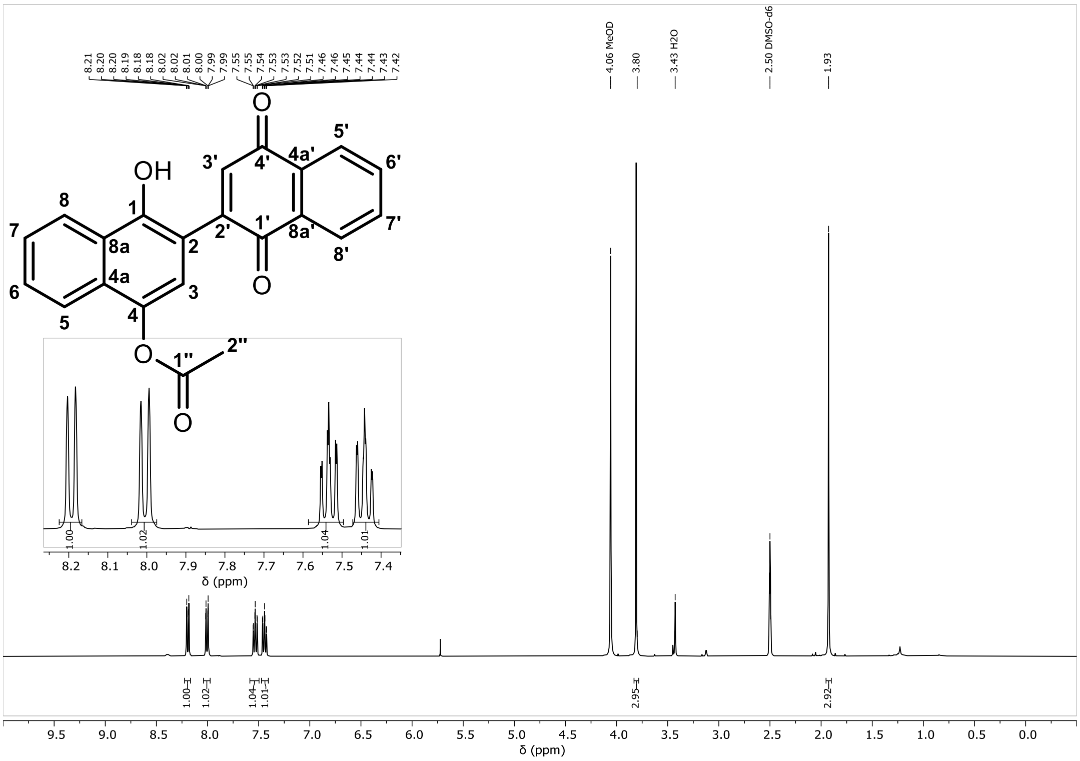

^{1}H NMR spectrum (400 MHz, $CDCl_3$) of **61**.

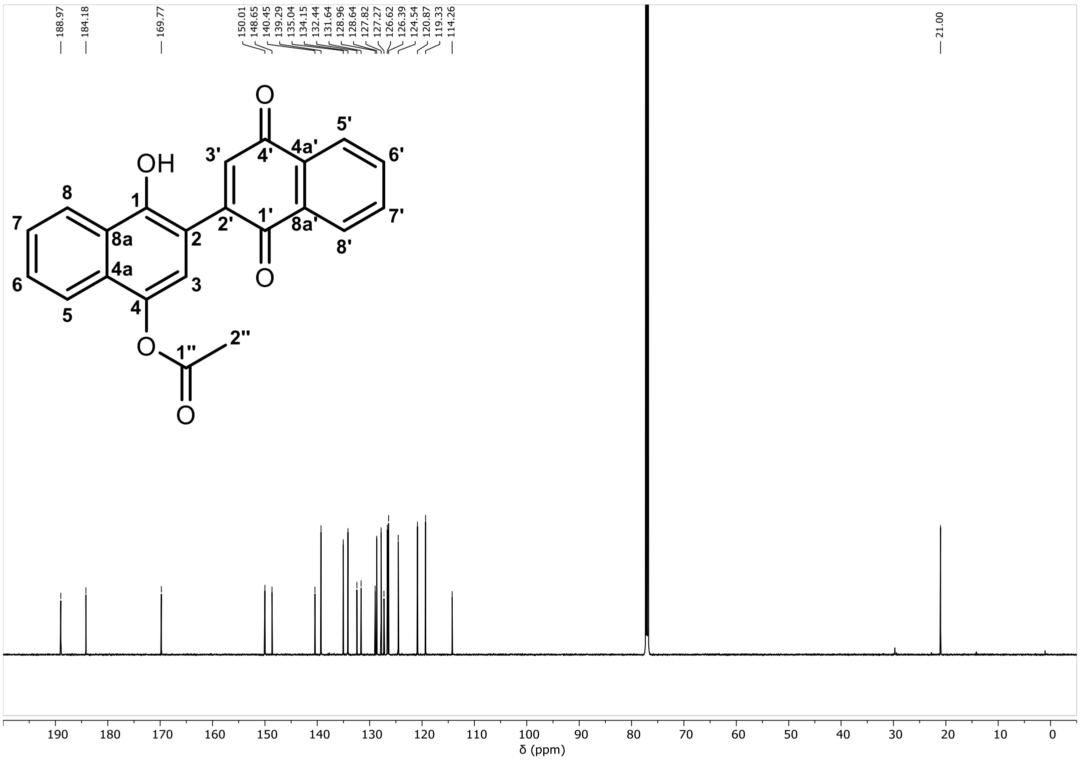

^{13}C NMR spectrum (101 MHz, $CDCl_3$) of **61**.

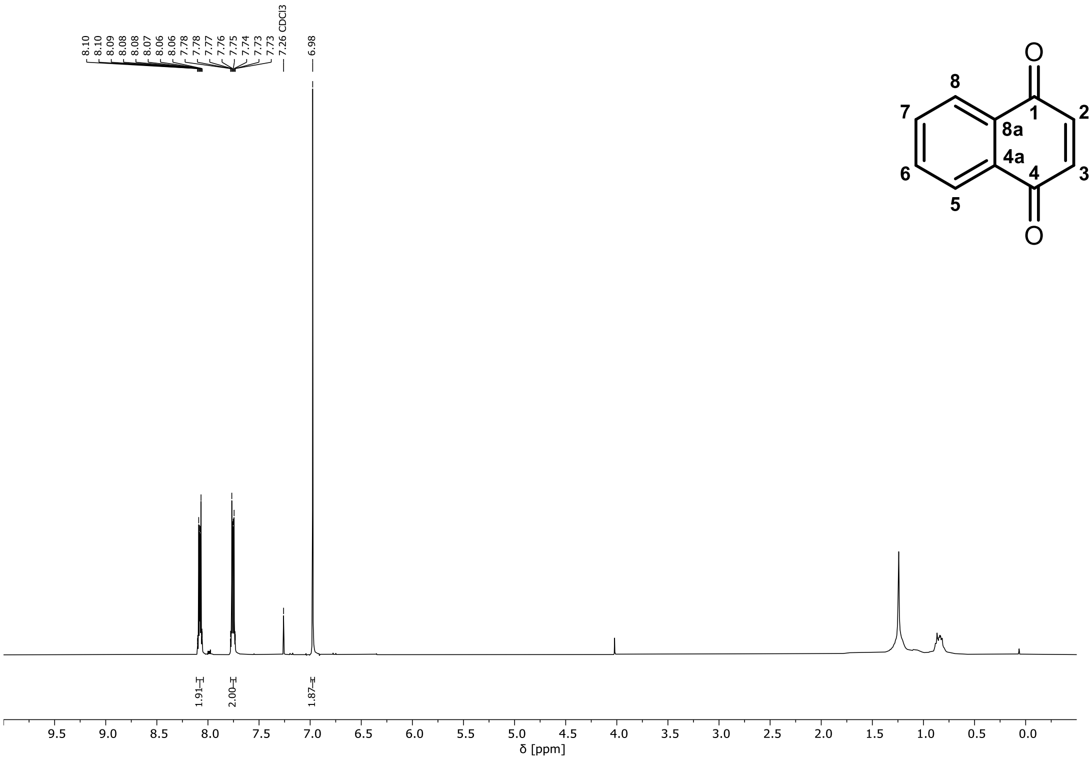

^{1}H NMR spectrum (400 MHz, $CDCl_3$) of **34**.

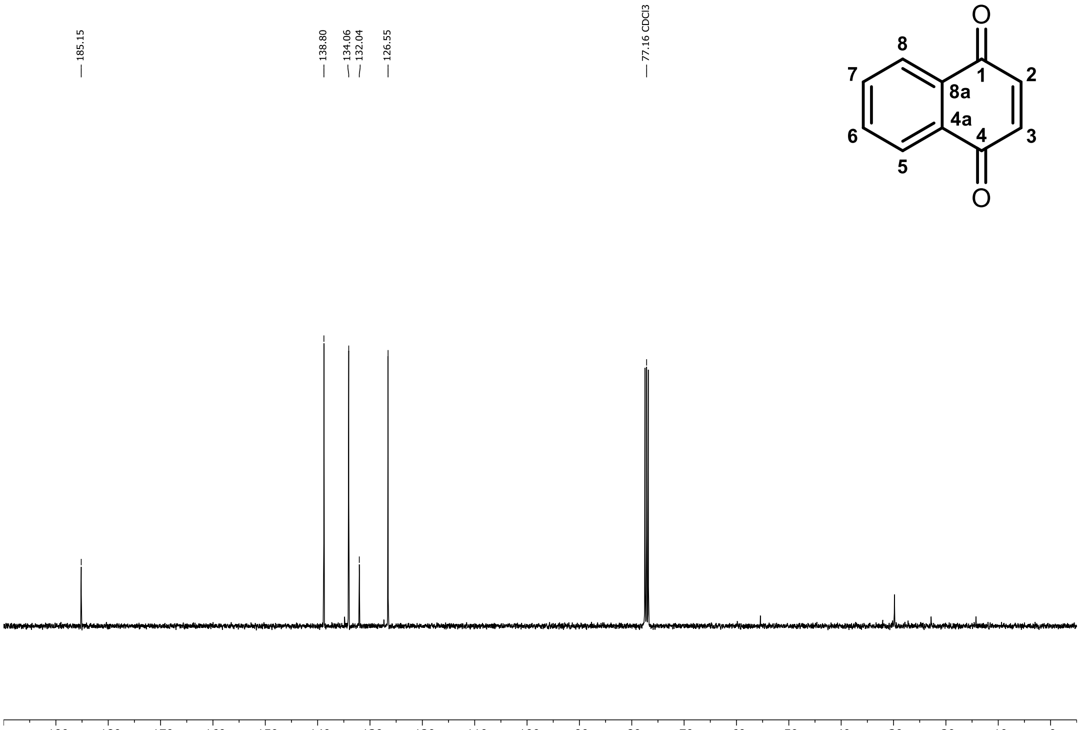

^{13}C NMR spectrum (101 MHz, $CDCl_3$) of **34**.

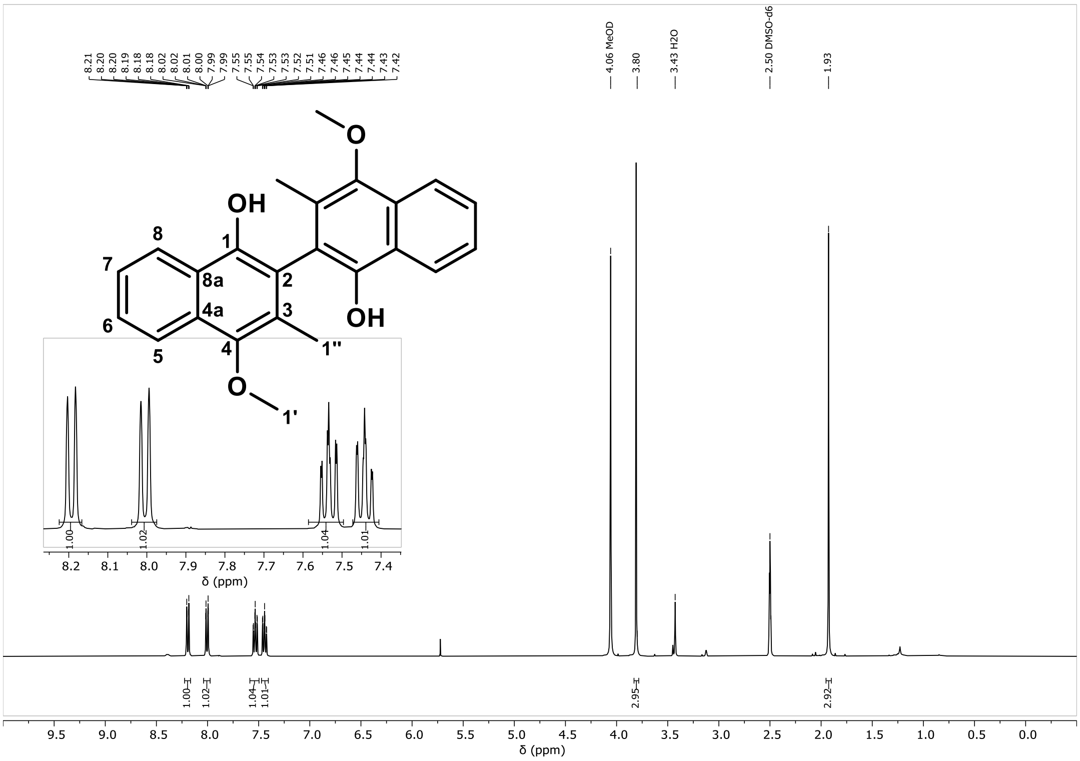

^{1}H NMR spectrum (400 MHz, DMSO-d_6) of **62**.

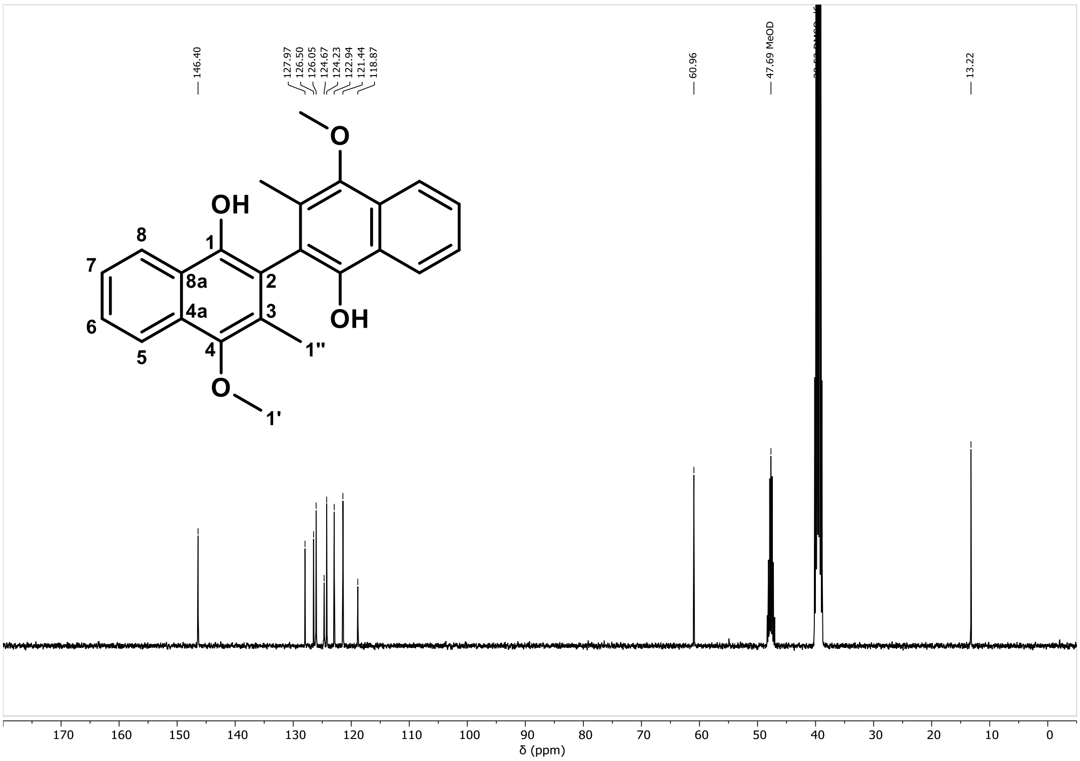

^{13}C NMR spectrum (101 MHz, DMSO-d_6) of **62**.

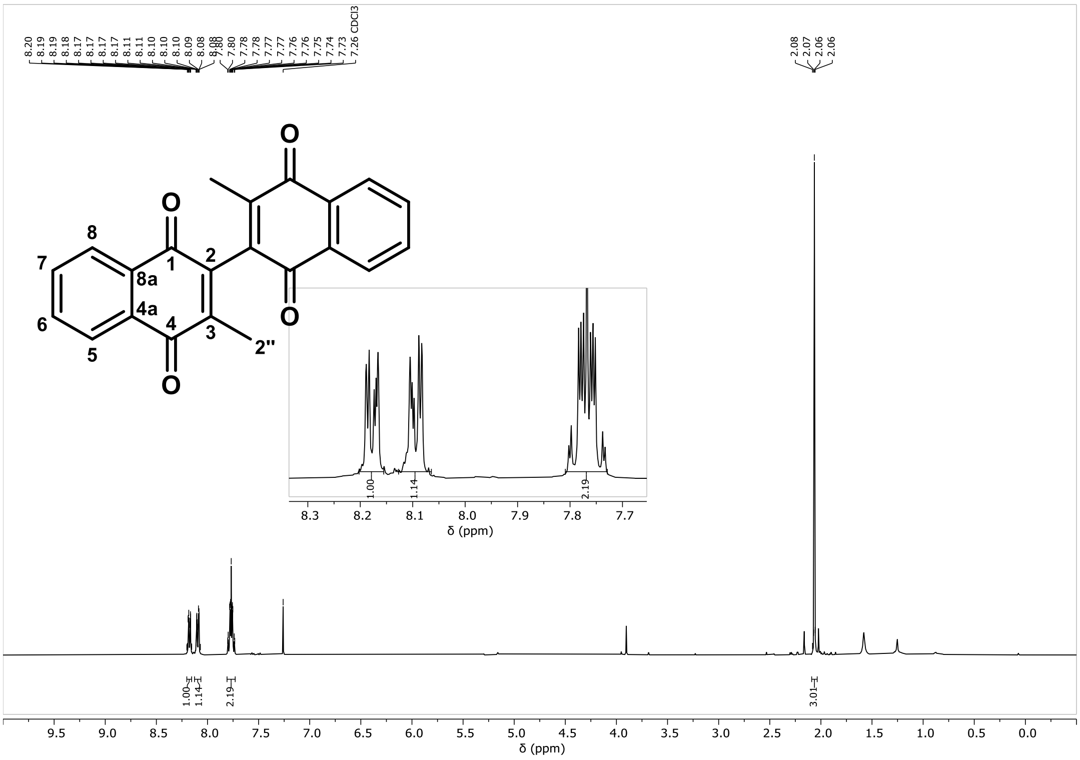

^{1}H NMR spectrum (400 MHz, $CDCl_3$) of **63**.

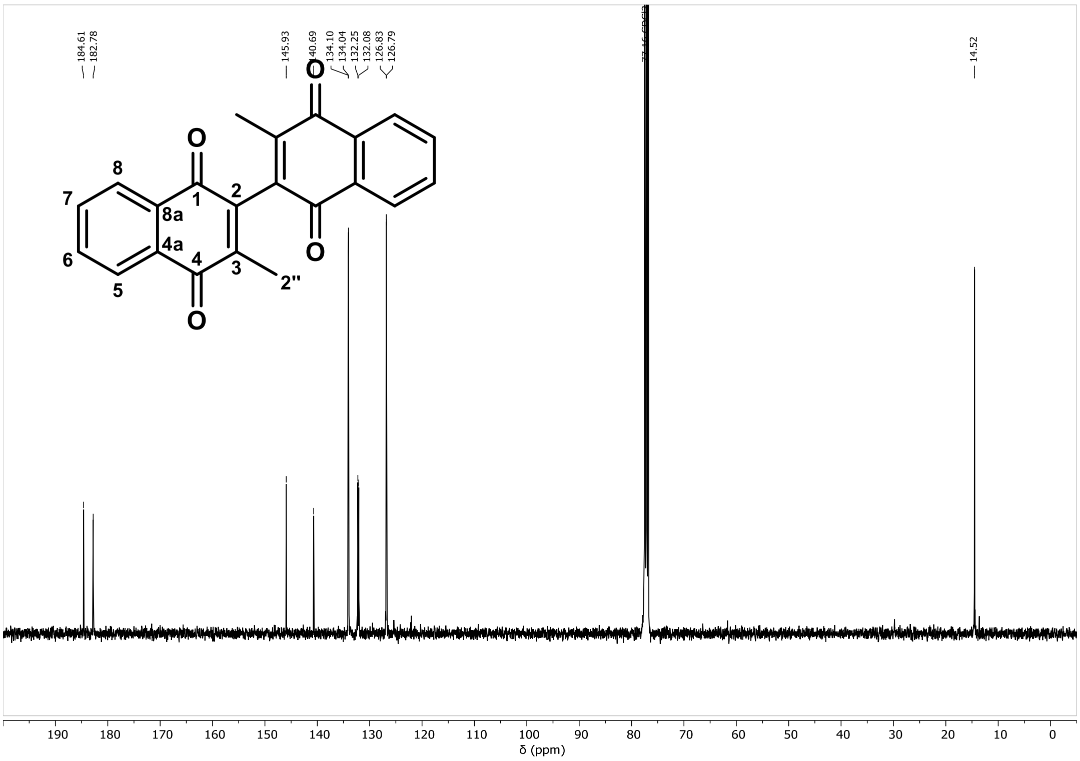

^{13}C NMR spectrum (101 MHz, $CDCl_3$) of **63**.

9 References

(1) Fulmer, G. R.; Miller, A. J. M.; Sherden, N. H.; Gottlieb, H. E.; Nudelman, A.; Stoltz, B. M.; Bercaw, J. E.; Goldberg, K. I. NMR Chemical Shifts of Trace Impurities: Common Laboratory Solvents, Organics, and Gases in Deuterated Solvents Relevant to the Organometallic Chemist. *Organometallics* **2010**, *29*, 2176–2179.

(2) Gütz, C.; Stenglein, A.; Waldvogel, S. R. Highly Modular Flow Cell for Electroorganic Synthesis. *Org. Process Res. Dev.* **2017**, *21*, 771–778.

(3) Zirbes, M.; Graßl, T.; Neuber, R.; Waldvogel, S. R. Peroxodicarbonate as a Green Oxidizer for the Selective Degradation of Kraft Lignin into Vanillin. *Angewandte Chemie (International ed. in English)* **2023**, *62*, e202219217.

(4) Gruber, C. C.; Oberdorfer, G.; Voss, C. V.; Kremsner, J. M.; Kappe, C. O.; Kroutil, W. An algorithm for the deconvolution of mass spectroscopic patterns in isotope labeling studies. Evaluation for the hydrogen-deuterium exchange reaction in ketones. *J. Org. Chem.* **2007**, *72*, 5778–5783.

(5) Nguyen Van, T.; Kesteleyn, B.; Kimpe, N. de. Synthesis of 1,3-disubstituted naphtho[2,3-c]pyran-5,10-diones. *Tetrahedron* **2001**, *57*, 4213–4219.

(6) Ferreira, V. F.; Schmitz, F. J. Reductive-alkylation and aromatic coupling reactions of 1,4-benzoquinone derivatives promoted by ethylaluminum dichloride. *J. Organomet. Chem.* **1998**, *571*, 1–6.

(7) Laatsch, H. Dimere Naphthochinone, II. Einfache und regioselektive Synthese von Naphthohydrochinon-monoalkylethern über 2,3-Dihydronaphthochinone. *Liebigs Ann. Chem.* **1980**, *1980*, 140–157.

(8) Liu, X.; Liu, B.; Liu, Q. Migratory Hydrogenation of Terminal Alkynes by Base/Cobalt Relay Catalysis. *Angew. Chem., Int. Ed. Engl.* **2020**, *59*, 6750–6755.

(9) Kumamoto, T.; Aoyama, N.; Nakano, S.; Ishikawa, T.; Narimatsu, S. Synthesis of enantiomeric 4-hydroxypropranolols from 1,4-dihydroxynaphthalene. *Tetrahedron: Asymmetry* **2001**, *12*, 791–795.

(10) Weimar, M.; Dürner, G.; Bats, J. W.; Göbel, M. W. Enantioselective synthesis of (+)-estrone exploiting a hydrogen bond-promoted Diels-Alder reaction. *J. Org. Chem.* **2010**, *75*, 2718–2721.

(11) Pluim, H.; Wynberg, H. Catalytic asymmetric induction in oxidation reactions. Synthesis of optically active epoxynaphthoquinones. *J. Org. Chem.* **1980**, *45*, 2498–2502.

(12) Marchand, A. P.; Reddy, G. M. Mild and Highly Selective Ultrasound-Promoted Zinc/Acetic Acid Reduction of C=C Bonds in α,β-Unsaturated γ-Dicarbonyl Compounds. *Synthesis* **1991**, *1991*, 198–200.

(13) Laatsch, H. Dimere Naphthochinone, 13. Synthese m-substituierter 4-Methoxy-1-naphthole – β-Halogenalkylether als Schutzgruppen für Phenole / Dimeric Naphthoquinones, 13. Synthesis of m-Substituted 4-Methoxy-1-naphthols – β-Haloalkyl Ethers as Protective Groups in Phenols. *Z. für Naturforschung B* **1985**, *40*, 534–542.

(14) Hammill, J. T.; Contreras-García, J.; Virshup, A. M.; Beratan, D.; Yang, W.; Wipf, P. Synthesis and chemical diversity analysis of bicyclo3.3.1non-3-en-2-ones. *Tetrahedron* **2010**, *66*, 5852–5862.
